# Direct Identification of the *Meloidogyne incognita* Secretome Reveals Proteins with Host Cell Reprogramming Potential

**DOI:** 10.1371/journal.ppat.1000192

**Published:** 2008-10-31

**Authors:** Stéphane Bellafiore, Zhouxin Shen, Marie-Noelle Rosso, Pierre Abad, Patrick Shih, Steven P. Briggs

**Affiliations:** 1 Division of Biological Sciences, University of California, San Diego, La Jolla, California, United States of America; 2 INRA, Unité Interactions Plantes-Microorganismes et Santé Végétale, Antibes, France; The University of North Carolina at Chapel Hill, United States of America

## Abstract

The root knot nematode, *Meloidogyne incognita*, is an obligate parasite that causes significant damage to a broad range of host plants. Infection is associated with secretion of proteins surrounded by proliferating cells. Many parasites are known to secrete effectors that interfere with plant innate immunity, enabling infection to occur; they can also release pathogen-associated molecular patterns (PAMPs, e.g., flagellin) that trigger basal immunity through the nematode stylet into the plant cell. This leads to suppression of innate immunity and reprogramming of plant cells to form a feeding structure containing multinucleate giant cells. Effectors have generally been discovered using genetics or bioinformatics, but *M. incognita* is non-sexual and its genome sequence has not yet been reported. To partially overcome these limitations, we have used mass spectrometry to directly identify 486 proteins secreted by *M. incognita*. These proteins contain at least segmental sequence identity to those found in our 3 reference databases (published nematode proteins; unpublished *M. incognita* ESTs; published plant proteins). Several secreted proteins are homologous to plant proteins, which they may mimic, and they contain domains that suggest known effector functions (e.g., regulating the plant cell cycle or growth). Others have regulatory domains that could reprogram cells. Using *in situ* hybridization we observed that most secreted proteins were produced by the subventral glands, but we found that phasmids also secreted proteins. We annotated the functions of the secreted proteins and classified them according to roles they may play in the development of root knot disease. Our results show that parasite secretomes can be partially characterized without cognate genomic DNA sequence. We observed that the *M. incognita* secretome overlaps the reported secretome of mammalian parasitic nematodes (e.g., *Brugia malayi*), suggesting a common parasitic behavior and a possible conservation of function between metazoan parasites of plants and animals.

## Author Summary

Parasitic nematodes are microscopic worms that cause major diseases of plants, animals, and humans. Infection is associated with secretion of proteins by the parasite; these proteins suppress the immune system and cause other changes to host cells that are required for infection. Identification of secreted proteins has been difficult because they are released only in trace amounts. We have developed very sensitive methods that enabled the discovery of 486 proteins secreted by the root knot nematode, *Meloidogyne incognita*; prior to this, only a handful of secreted proteins were known. Several secreted proteins appear to mimic normal plant proteins, and they may participate in the process by which the nematode hijacks the plant cell for its own purposes. *Meloidogyne* species infect many crops, including corn, soybean, cotton, rice, tomato, carrots, alfalfa, and tobacco. The discovery of these secreted proteins could lead to new methods for protecting these important crops from nematode damage. We observed that the secretome of the human pathogen, *Brugia malayi*, overlaps that of *M. incognita*, suggesting a common parasitic behavior between pathogens of plants and animals.

## Introduction

*M. incognita* can infect 1,700 plant species [1]. At the infective juvenile (J2) stage of development, *M. incognita* enters the elongation zone of the root and burrows through the apoplast to the root tip where it enters the vascular cylinder, moving up to the zone of root differentiation. The nematode then inserts its stylet into the plant cell cytoplasm and induces nuclear division without cytokinesis, creating multinucleate giant cells that nurture the developing worm. Infection is associated with the reprogramming of plant cell development rather than host cell death [2]. *M. incognit*a infection causes plant defense genes to become either promptly suppressed or transiently induced, in contrast to incompatible interactions, which immediately induce and sustain expression of defense genes [3].

The proteins and metabolites secreted from the esophageal glands (subventral and dorsal glands) of plant-parasitic nematodes are thought to be responsible for compatibility [4]. The two subventral gland (SvG) cells are biologically active during the J2 stage, while the dorsal gland cell is predominantly active on the second day post-infection through to the end of the nematode's life. *In vivo* observations of the root cyst nematode, *Heterodera schachtii*, revealed that the dorsal gland secretions are released through the stylet into the plant cell [5]. Other nematode tissues also secrete proteins that may be important for plant-pathogen interaction: two amphids localized in the anterior part of the worm, around the lip region, and two phasmids at the posterior part could be receptors for chemotaxis [6]. These two kind of organs contain socket cells that are highly secretory but functions of these secretions remain mostly unknown [6],[7].

Following the establishment of compatibility, pathogen-produced effector molecules are the key to infection. These molecules have been found in well-characterized pathosystems where they modulate host signaling pathways to prevent defense responses [8], but little is known about effectors that mediate plant-metazoan pathogenesis. Bird and Saurer (1967) characterized secreted molecules from the esophageal gland cells of *Meloidogyne javanica* [9]. They showed that the secretions were mainly proteins; no nucleic acids were detected. Antibodies have been used to monitor the expression of esophageal antigens from several plant nematode species [10]. In *Globodera rostochiensis*, antibodies recognized proteins present both in the subventral gland cells and on the surface of the nematode [11]. Other studies of *Meloidogyne spp.* showed that silencing of genes expressed in the SvG reduced pathogenicity [12],[13]. Secretions of the animal-parasitic nematode, *Trichinella spiralis*, appear to reprogram the host cell into a nurse cell, and *in vitro* injection of collected secretions from *T. spriralis* into rat muscles mimicked cellular changes that occur *in vivo* [14].

Pathogen associated molecular patterns (PAMPs) are typically proteins or nucleic acids that are wide-spread in microbes and are shed during infection. Host receptors are activated by PAMPs. For example, flagellin from bacteria stimulates innate immunity from both plant and mammalian cells [15],[16]. No PAMPs from metazoans have been reported.

The identification of secreted proteins from *M. incognita* may facilitate the discovery of effectors and PAMPs. Effectors might account for how root knot nematodes reprogram plant cells to become giant cells and to form root knots. Several hypotheses have been proposed to explain how *M. incognita* establishes compatibility with its plant hosts. It may invade root tissues by first producing cell wall-degrading enzymes. Once established in the root it could produce detoxifying enzymes, followed by additional effectors that induce giant cell formation [2].

Discovery of secreted proteins by bioinformatics is possible for organisms with known genomic DNA sequences. Recently a secretome of *Plasmodium falciparum* comprising 200 proteins was predicted using bioinformatics [17]. A similar approach cannot be applied to *M. incognita* since its genomic sequence is not yet known. Instead, experimental approaches have been used. A transcript profile of the esophageal gland cells of *M. incognita* has been reported [18]. Based on bioinformatic analyses of cDNA sequences, secreted proteins were predicted to include cellulases, chitinases, extensins, proteases, and a superoxide dismutase (SOD). In a recent study, Roze *et al.* (2008) analyzed the cDNA sequences of proteins putatively secreted by *Meloidogyne chitwoodi* [19]. They identified cDNAs corresponding to 398 putative proteins and confirmed by *in situ* hybridization seven that are specifically transcribed in the SvG, one in the dorsal gland, and one in the phasmids.

We chose to directly identify secreted proteins based on the pioneering work of Jaubert *et al.* (2002) who used resorcinol to induce esophageal gland secretion by *M. incognita*. It was clear from their work that many more proteins were secreted than were identified [20]. To explore the *M. incognita* secretome in greater depth, we developed sensitive methods for high-throughput proteomics based liquid chromatography, nano-electrospray ionization and tandem mass spectrometry (nanoLC ESI MS/MS) [21]. This method requires both a protein database as well as algorithms to assign peptide sequences to mass spectra. The conservation of protein sequences between species enables a protein database from heterologous species to partially substitute for a database from the cognate species. To control for false positive identifications we reversed the amino acid sequence of the protein databases and filtered the search result so that our protein false discovery rate (FDR) was 0.4%. While use of heterologous databases precludes discovery of peptides that are unique to the organism, thereby reducing the number of proteins identified, it nevertheless opens a window on the proteome. In this study, we identified 486 proteins from the *M. incognita* secretome, including proteins that could play a role in root knot formation by regulating the plant cell cycle and plant growth.

## Results

### Extraction and identification of secreted proteins

**Figure 1:**
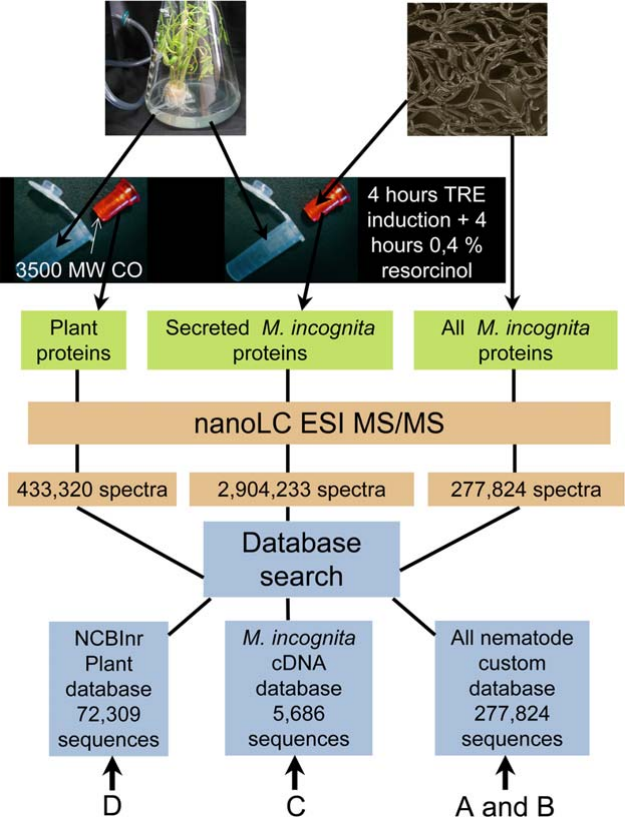
Sample process flow chart. *M. incognita* proteins were collected from the aqueous medium (the secretome) and from extracts of worms. Proteins in the tomato root exudates (TRE) that diffused across a 3,500Da filter membrane were also collected. Proteins were identified by nanoLC ESI MS/MS. A, B, C and D are Protein databases used for protein identification, complementary information for each database are available on Table S3.

We induced protein secretion by J2 stage *M. incognita* nematodes by treating first with filtered, low-molecular weight (<3,500 Da) tomato root exudates followed with resorcinol. A sample of nematodes was removed and stained with Coomassie Blue, which confirmed that treatment caused proteins to be secreted from the stylet region. Secreted proteins were extracted from the solution bathing unstained nematodes and were identified by nanoLC ESI MS/MS (**Figure 1**). To ensure the accuracy of protein identifications, the threshold for mass spectral quality was set at high stringency using very low peptide and protein false discovery rates (FDRs). FDRs were determined by searching the MS/MS spectra against a concatenated 1∶1 forward-reverse database [22]. MS/MS spectra with peptide FDR less than 0.1% were considered valid. We set the protein FDR at 0.4%. Due to the multiple protein databases used in this study and the natural sequence redundancy in the protein databases, the same peptide sometimes appeared in multiple protein sequences. In order to address this protein redundancy issue, protein sequences containing the same set or subset of valid peptides were grouped together into protein groups with the best match listed first [23]. The numbers of proteins we report in this paper are protein group numbers. This is a conservative measure because more than one protein within a group may actually be detected. Only proteins with at least 2 valid MS/MS spectra were reported. Proteins with a single unique peptide but multiple spectra were manually validated.

Observations of *M. incognita* and *Heterodera glycines* stylet activity with and without stimulation by neuroregulators has been extensively studied [24]. The authors reported that neuroregulators induce a dramatic stimulation of stylet pulsing frequency but they pointed out that even without stimulation, stylet pumping occurred. We observed by both mass spectrometry and silver stained gels that J2 nematodes secrete low but detectable levels of proteins (less than 1% as much as after stimulation). Proteins identified in the absence of stimulation included 14-3-3b [listed as protein (4) in **Table S1**]; Hsp90 (9); SEC-2 (11); aldolase (20); glyceraldehyde-3-phosphate-dehydrogenase (14); protein with thioredoxin domain (52); and protein with glutathione S-transferase domain (43).

**Figure 2:**
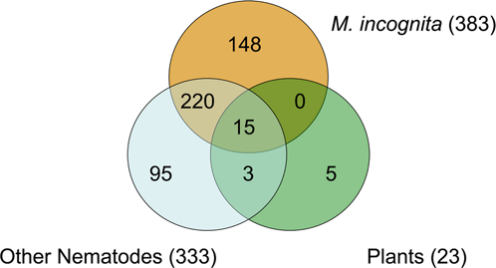
Distribution of secreted proteins identified in the protein databases. Venn diagram showing the distribution of secreted proteins identified using the *M. incognita*, Other Nematode, or Plant protein databases. A complete description of all databases is shown in Table S3.

We identified 486 proteins from the *M. incognita* secretome after treatment using a protein FDR of 0.4% (**Figure 2**). These include all seven proteins reported by Jaubert *et al.* (2002), indicating that our results both confirm and extend previous studies. The majority of the proteins (311; 64%) were identified by the detection of 2 or more peptides. Of the 175 proteins identified by only one peptide, some were previously shown to be secreted. Proteins identified by several MS/MS spectra but only one peptide have been manually validated and the spectra are summarized in **Table S2**.

**Figure 3:**
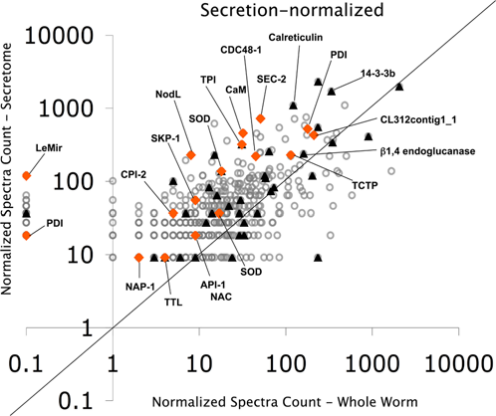
Relative abundance of secreted proteins compared to extracts from intact nematodes. Spectrum counting was used for relative protein quantification compating the secretome to the intact nematode proteome. The number of valid MS/MS spectra from each protein was normalized to the total MS/MS spectra number of each dataset. The normalized spectrum count ratio of each protein (secretome/whole worm proteome) was used to evaluate if the protein was enriched in the secretome. Circle represents all secreted proteins identified in this study; Triangle represents proteins previously reported to be in the secretome (A complete description of these proteins is shown in Table S5); Diamond represents proteins that we found in *M. incognita* secretome and that could play a crucial role in the establishment of the host-pathogen compatibility. These proteins are discussed in detail.

To serve as a control for potential contamination of the secretome by cellular debris, we examined proteins extracted from intact nematodes. Visual inspection of nematode preparations did not reveal any signs of damage or debris. We compared the relative abundance of proteins in the secretome to their abundance in extracts from intact nematodes. This revealed that many (19%) of the secreted proteins are highly abundant in intact nematodes; these were removed from consideration out of concern that they may be contaminants, even though they were not observed in the water control. The normalized spectrum count ratio of each protein (secretome/whole nematode proteome) was used to calculate secretome enrichment. Most of the proteins identified in the solution bathing treated nematodes (i.e., the secretome) were significantly less abundant or absent in the proteome of whole nematodes, providing further evidence that they are indeed secreted (**Table S1**, column 5). Approximately 81% (394) of the secreted proteins are enriched and 60% (288) are at least 2-fold more abundant in the *M. incognita* secretome (**Figure 3** and **Table S1**, column 5). Due to the relatively large size of the SvGs and the number of dense granules in them, it would not be surprising to find secreted proteins in the whole nematode extract. The remaining 19% (92) of non-enriched proteins (e.g., actin) may in fact be secreted, but to be conservative we do not consider them further.

High-throughput nano-LC ESI MS/MS depends upon protein databases and is most useful when the entire annotated genome sequence of an organism is available. However, with the proliferation of genome projects, adequate sequence information has become available to enable protein identification using databases from other species. We used two *M. incognita* cDNA sequence databases with sequence databases from all nematodes and plants (**Figure 1** and **Table S3**). Nearly all of the secreted proteins (481; 99%) were identified by reference to the nematode protein sequences (**Figure 2**). Approximately half of the proteins (235; 48%) were identified both by *M. incognita* sequence and by sequence from other nematodes. Only 20% (95) were identified by orthologous nematode sequence alone and 31% (151) from *M. incognita* sequence alone. A total 69% of the *M. incognita* secretome could have been identified without reference to the *M. incognita* DNA sequences (**Figure 2**). **Table S4** shows full-length proteins with identified peptides derived from searching the *M. incognita* sequence database.

### Our observations encompass previously reported components of secretomes

Comparison of our observations with published reports of proteins secreted by *M. incognita* revealed extensive overlap and, in addition, we identified orthologs of proteins that are secreted by other parasitic nematodes (**Table S5**). Among the 10 most abundant proteins in our data (**Table S1**), 14-3-3b protein and calreticulin were previously shown to be produced and secreted by the SvG of *M. incognita* [25],[26].

Comparison of our *M. incognita* secretome with that from the parasitic helminth, *Brugia malayi*, reveals significant overlap [27]. Of the 80 proteins known to be secreted by *B. malayi*, 26 are also secreted by *M. incognita* (**Table S5**). This conserved group includes proteins involved in detoxification (e.g. SODs), cytosolic stress response (e.g. 14-3-3-like proteins), cytosolic energy metabolism (e.g. a triose phosphate isomerase), structure (e.g. actin), protein turnover or folding (e.g. ubiquitin-like proteins SMT3 and protein disulfide isomerases PDI), protease inhibitors (e.g. Cystatin-type Cysteine Protease Inhibitor CPI-2), and two transthyretin-like family proteins (TTLs).

The discovery of effectors from nematodes has lagged behind progress made with bacterial and oomycete pathogens, but recently phytopathogenic nematode effectors have been reported. We re-examined our mass spectra using sequence from members of the SPRYSEC protein family, which includes effectors from *G. rostochiensis* [28],[29]. We also searched for Cg-1, an *M. incognita* candidate effector gene acting in the Mi1.2 resistance pathway [30], and for MAP-1, a putative avirulence protein produced by amphids [31]. We did not identify peptides corresponding to any of these proteins in the *M. incognita* secretome nor in the extract of intact nematodes.

We searched our mass spectra for peptides from proteins secreted by *M. chitwoodi* but could find none [19]. However, by doing a BLASTP search using proteins identified in our study, we were able to show that 4 proteins secreted by *M. chitwoodi* are also in the *M. incognita* secretome (cysteine protease, beta-1,4-endoglucanase, VAP-1 and pectate lyase). The reason we initially missed them is because our search algorithms require exact amino acid sequence matches but the peptides identified in the *M. incognita* secretome have at least one amino acid difference compared to those deduced from *M. chitwoodi* ESTs.

### Annotation of secreted proteins using KOGs and BLAST

Using the euKaryotic Orthologous Groups (KOGs) classification scheme to annotate the secreted proteins [32] we found that 103 proteins catalyze post-translational modifications, protein turn-over or chaperone functions; 93 participate in protein synthesis or secretion; 88 trigger metabolic reactions; 48 interact with nucleic acid (DNA or RNA); 25 are involved in signal transduction and 33 interact with actin or microtubules. We performed a BLASTP search for each protein to refine their annotations (**Table S1**, column 8 and 9). We combined the KOGs and BLASTP results to classify the *M. incognita* secretome into 9 subfamilies (**Tables S1** and **S6**): Proteins interacting with actin or microtubules (33 proteins, family 1); Proteins interacting with nucleic acids (48 proteins, family 2); Post-translational modification, protein turnover, and chaperone functions (103 proteins, family 3); Metabolism (88 proteins, family 4); Signal transduction (25 proteins, family 5); Protein synthesis and secretion (93 proteins, family 6); Detoxification (17 proteins, family 7); Cell wall modification enzymes (8 proteins, family 8); and Other (94 proteins, family 9).

Nematode infection causes gene expression changes in the plant cell [33]. These changes could be due to indirect effects, but there is evidence for secreted nematode proteins interacting directly with plant transcription factors (reviewed in references [2],[34]). This was first suggested when putative secreted factors were observed to have nuclear localization signals (NLSs) [18]. Later, an mRNA was identified from the esophageal gland of *H. schachtii* and the capacity of its expressed protein to interact *in planta* with two putative plant SCARECROW-like transcription factors was reported [35]. To determine whether the secreted proteins we observed could be targeted to the plant nucleus and could potentially modify plant gene expression, we searched for NLSs and DNA or chromatin interaction motifs. We found 66 proteins that meet one or both criteria: 26 proteins with an NLS motif and 40 additional proteins with putative nucleotide binding activity. Of these, 8 proteins are predicted to have both an NLS and a nucleotide binding activity (**Table S7**).

### The *M. incognita* secretome includes mimetics that may have arisen by horizontal gene transfer

We identified 5 secreted proteins present only in the plant protein sequence database (**Table S1**). Among them was LeMir, a protease inhibitor known to be upregulated in plants during nematode infection. Low molecular weight tomato root exudates were used to induce nematode secretion so we examined as a control the water medium without nematodes for proteins and peptides that could potentially diffuse across the membrane that separated root exudates from nematodes. No proteins were detected in gels by silver staining (data not show) but, using mass spectrometry, we identified 4 peptides derived from 3 plant proteins (**Table S8**). Only one protein overlapped with the nematode secretome (remorin 1); we could not detect LeMir or any of the other plant homologs in the nematode secretome indicating that they were not contaminants. Earlier reports identified other proteins with putative horizontal gene transfer (HGT) origins. We confirmed that several of these are in the secretome, including two pectate lyases [36], a cellulose binding protein [37], and two beta-1,4-endoglucanases [38],[39]. McCarter *et al.*, (2003) reported cases of potential HGT from microbes; we confirmed their existence in the secretome, including a Rhizobacterial homolog of nodL (CL221Contig1_1) and a polygalacturonase (221104r1.1_1) [40]. Two other putative HGT candidates were identified: a conserved hypothetical protein from *Trichomonas vaginalis* (MI00116) and a putative Type IV secretory pathway VirB6 component from *Ehrlichia canis str. Jake* (CL1842Contig1_1) (**Table 1**).

**Table 1:** Selected proteins from the *M. incognita* secretome.

Protein number	Organism	Corresponding *M. incognita* accession nb.	Number unique peptides	Secretion/Whole Mi - normalized	KOGs	Protein class	Protein family homology
18	*M. incognita*	CL321Contig1_1_AA	10	2.01	DZ	1	TCTP
364	*M. incognita*	MI02098	1	4.58	BD	2	NAP-1
453	Other nematodes	MP01475	1	2.04	K	2	NAC
4	*M. incognita*	CL2470Contig1_1_AA	17	5.07	O	3	14-3-3b
16	*M. incognita*	CL1191Contig1_1_AA	10	4.37	O	3	CDC48-1
138	*M. incognita*	CL210Contig1_1_AA	3	4.17	O	3	ubiquitin carboxyl-terminal hydrolase
185	*M. incognita*	CL781Contig1_1_AA	2	7.33	?	3	Protease inhibitor
202	*M. incognita*	210k21r1.1_1_AA	2	4.07	O	3	ubiquitin carboxyl-terminal hydrolase
235	*M. incognita*	MI01032	2	6.11	O	3	SKP1
331	*M. incognita*	217d04r1.1_1_AA	1	1.83	O	3	ubiquitin-activating enzyme
362	*M. incognita*	MI06174	1	2.04	?	3	Protease inhibitor
376	*M. incognita*	MI09158	1	3.05	O	3	Ubiquitin-like
380	*M. incognita*	MI00316	1	9.16	?	3	metalloproteinase
407	Other nematodes	PP00571	2	36669.00	O	3	14-3-3b
4982	*L. esculentum*	Unknown	3	119172.00	?	3	LeMir Protease inhibitor
17	*M. incognita*	CL480Contig2_1_AA	10	10.35	G	4	Triosephosphate isomerase
170	*M. incognita*	CL221Contig1_1_AA	2	28.64	E	4	NodL
261	*M. incognita*	MI06202	2	1.41	E	4	metalloproteinase
21	*M. incognita*	CL12Contig1_1_AA	9	14.32	T	5	CaM and related proteins
118	*M. incognita*	CL109Contig1_1_AA	3	4.01	T	5	CaM and related proteins
312	*M. incognita*	CL3006Contig1_1_AA	1	0.73	T	5	CaM and related proteins
378	*M. incognita*	MI01602	1	1.31	T	5	CPK31
472	Other nematodes	MJ05164	1	1.31	R	7	thioredoxin
291	*M. incognita*	221l04r1.1_1_AA	1	0.56	?	8	polygalacturonase
11	*M. incognita*	CL5Contig2_1_AA	14	14.20	?	9	SEC2
13	*M. incognita*	CL312Contig1_1_AA	13	2.04	?	9	No putative function
165	*M. incognita*	MI00116	3	2.04	R	9	No putative function
194	*M. incognita*	CL1842Contig1_1_AA	2	1.25	?	9	Type IV secretory pathway VirB6 components
320	*M. incognita*	CL2552Contig1_1_AA	1	2.29	?	9	Transthyretin-like family
7	*M. incognita*	CL673Contig1_1_AA	16	2.92	O	3;7	PDI
40	*M. incognita*	CL1Contig68_1_AA	6	15.28	O	3;7	glutathione S-transferase-1
67	*M. incognita*	206d07c1.1_1_AA	5	4.01	O	3;7	glutathione S-transferase-1
242	*M. incognita*	MI03461	2	1.26	O	3;7	glutathione peroxidase
454	Other nematodes	cr01.Contig0.wum.390.1	1	18335.00	O	3;7	PDI
28	*M. incognita*	MI02513	8	7.64	P	4;7	SOD
278	*M. incognita*	CL129Contig1_1_AA	1	2.16	P	4;7	SOD
424	Other nematodes	MH02402	2	4.58	Q	4;7	glutathione synthetase

We assigned a protein number to index proteins across tables presented in this study. Proteins are ranked first according to their “protein class” and listed from highest to lowest confidence levels based on the number of unique peptides identified per protein. A description of each “protein class” is available on **Table S6** and accession numbers for all proteins are available on **Table S1**.

### Transcripts corresponding to secreted proteins are enriched in the SvG

**Figure 4:**
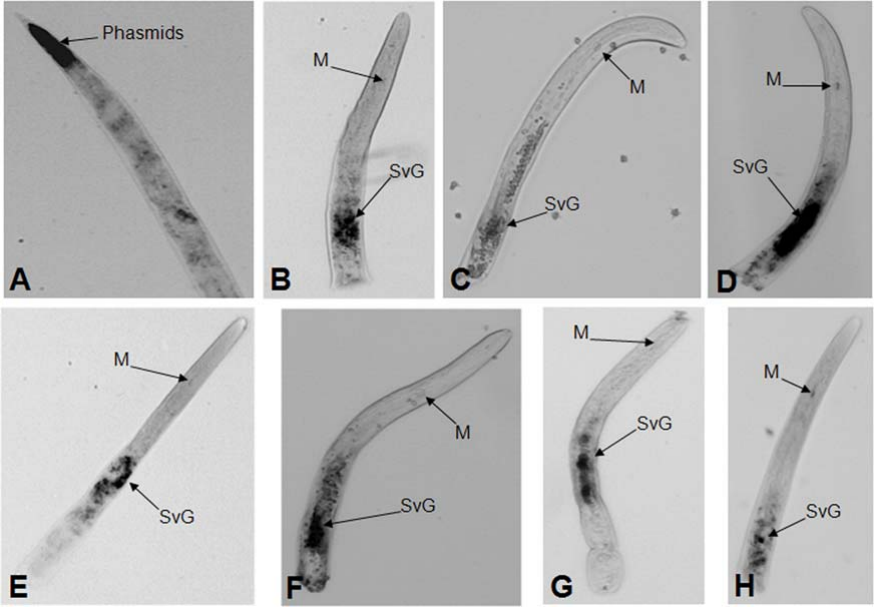
Localization of gene expression by *in situ* hybridization. Digoxigenin-labeled antisense cDNA probes of selected gene clones were hybridized to transcripts expressed within cells of J2 stage *Meloidogyne incognita*. Sections of the nematode were incubated with antisense probes designed based on DNA sequence of the following contigs: *A*, CL1191Contig1_1; *B*, CL312Contig1_1; *C*, CL5Contig2_1; D AF100549 (β-1,4-endoglucanase), E, CL2552Contig1_1; *F*, CL321Contig1_1; G, AF402771 (calreticulin); H, CL480Contig2_1. M = metacorpus, SvG = subventral glands.

We localized mRNA corresponding to a subset of secreted proteins using *in situ* hybridization to J2 stage nematodes (**Figure 4**). As a positive control, we localized transcripts for two previously characterized secreted proteins from the SvG: beta-1,4 endoglucanase and calreticulin (**Figure 4D **and** G** respectively). We tested and confirmed that the following members of the *M. incognita* secretome are also expressed specifically in the SvG: CL312Contig1_1 (protein with unknown function); CL5Contig2_1 (SEC2); CL2552Contig1_1 (Transthyretin-like family protein homolog); CL321Contig1_1 (Translationally-controlled tumor protein homolog); CL480Contig2_1 (triosephosphate isomerase homolog). A BLASTX search revealed that CL312Contig1_1 encodes for a *C. elegans* homolog (E value 9E-06) that is predicted to be a membrane protein with unknown function. We also found a transcript that encodes a putative CDC48 protein (contig CL1191Contig1_1) that is enriched in phasmid organs.

## Discussion

We identified several proteins at low levels in the water control (untreated nematodes). This is not surprising since, even in the absence of stimulation, *M. incognita* could secrete proteins [24]. The identified proteins could play a role in plant ROS signaling or in suppression of plant cell death. Protection from ROS could be provided by glyceraldehyde-3-phosphate-dehydrogenase [41], the transcript product from CL2662Contig1_1_AA (a protein with thioredoxin domain) or from CL2084Contig1_1_AA (a protein with a glutathione S-transferase domain). Additional proteins found in the water control include SEC-2 and 14-3-3b. We showed in this study an enrichment of SEC-2 transcript in the SvG and it was previously reported that 14-3-3b transcript is enriched in the dorsal oesophageal gland and in an undetermined tissue anterior to the metacarpus of *M. incognita* [25].

Following stimulation, we identified 486 proteins in the *M. incognita* secretome, representing functions that are potentially required for invasion, immune suppression, and host cell reprogramming. A published scheme classified most secreted proteins into four categories: cell wall degrading enzymes; detoxification enzymes; plant nuclear localized proteins; and giant cell formation [2]. To accommodate for the large increase in protein diversity reported here, we propose expanding the *M. incognita* secretome classification into 8 categories plus some proteins that were not classified (**Table 1** and **Table S6**); selected examples are described below.

### Protein synthesis and secretion

We identified several chaperones that may be involved in protein secretion: thioredoxin, glutathione peroxidases, cyclophilins, and protein disulfide isomerases (PDIs). PDIs have also been found in the secretion of the nematode, *Ostertagia ostertagi*, where their overexpression increases the yield of secreted proteins [42]. PDIs participate in actin filament polymerization, gene expression, cell-to-cell interactions and in the regulation of receptor functions [43],[44]. Cyclophilins are associated with protein trafficking, protein folding, chromatin remodeling, and chaperone activity [45]. Coaker *et al.* (2005) showed that the *Pseudomonas syringae* cysteine protease, AvrRpt2, requires activation by a plant cyclophilin before it can cleave RIN4 [46]. It is possible that *M. incognita* secretes cyclophilins to activate its effectors.

The correct folding of secreted nematode proteins may be necessary for infection. It has been shown previously that the AVR9 peptide elicitor of *Cladosporium fulvum* contains three disulfide bridges and that its correct folding depends on the redox state of the environment, with folding rates greatly increased in the presence of PDI [47]. If AVR9 is even partially reduced, it loses all activity, illustrating the importance of disulfide bridges.

### Cell wall modification enzymes

SvGs are known to secrete a beta-1, 4-endoglucanase *in planta* [48],[38],[49], as well as a pectinase [50] and an expansin [51]. We observed these and other cell wall degrading enzymes in the *M. incognita* secretome indicating that the nematode may use these enzymes for moving through the root or for assisting with plant cell wall remodeling during root knot formation.

### Detoxification

One of the earliest plant responses to infection is the production of reactive oxygen species (ROS) [52]. Based on our study, the *M. incognita* secretome contains detoxification enzymes that may be able to degrade ROSs. This could assist the nematode to establish a successful feeding site. It was previously reported that *M. incognita* secretes proteins which protect it from ROSs [53]. In plant tissues, SODs exist in three main families containing Cu and Zn, Mn, or Fe in their active site. We found two putative cytosolic CuZnSODs in the *M. incognita* secretome. A CuZnSOD was highly expressed and active in emergent symbiotic Rhizobium nodules of *Lotus japonicus* suggesting that this enzyme could play an important role in the early stages of symbiosis [54]. Taking this into consideration it is possible that the nematode enzyme may play a role in establishing compatibility with the plant cell.

ROSs are also scavenged by ascorbate peroxidases, cytochrome C-peroxidases, catalases, thioredoxins and glutathione peroxidases [55]. Two glutathione peroxidases and one thioredoxin were observed in the *M. incognita* secretome, as were several glutathione S-transferases. Normally these enzymes are induced in plants by H_2_O_2_, where they act as calcium-dependent cellular protectants [56], so perhaps the nematode enzymes also provide protection from ROS-catalyzed damage. A similar mechanism has been observed in the maize pathogen, *Ustilago maydis*, which overcomes host redox defenses by sensing peroxide with Yap1. Once activated, Yap1 induces *U. maydis* peroxidase gene expression, leading to the successful establishment of infection [57]. Therefore, it is possible that *M. incognita* may have evolved enzymes to control the global oxidative status of the plant cell as a way to increase its virulence.

### Proteins in the plant cell nucleus

Two of the most obvious consequences of nematode infection are distortion of the plant cell-cycle and cytoskeleton, leading to the formation of giant cells and the characteristic root knot [58],[59]. We identified several secreted proteins that could be targeted to the plant cell nucleus, where they could regulate gene expression resulting in some of the morphological changes observed. The target of nematode effectors to the plant nucleus was first suggested by the presence of putative secreted proteins with nuclear localization signals [18]. Later, a small, secreted peptide was identified that interacts *in planta* with two plant SCARECROW-like transcription factors [35]. We identified 66 secreted proteins with putative nuclear localization, DNA binding, or chromatin modification domains. These include two helicases, several RNA and DNA binding proteins, histones and the Nucleosome Assembly Protein, NAP-1 (**Table S7**). NAP proteins move histones into the nucleus, assist with nucleosome assembly, and modulate transcription [60].

### Giant cell formation

Several secreted proteins were identified that could potentially regulate plant cell proliferation including a CDC48-like protein (VCP/CDC48), SKP1, TCTP, NAC protein, and a CDPK. We confirmed by *in situ* hybridization that the corresponding mRNA of the CDC48-like protein is specifically expressed in the nematode phasmid (**Figure 4A**). A previous study using Coomassie Brillant Blue G-250 revealed that phasmids secrete proteins that take up the stain [61]. Phasmids are specialized pairs of sensory organs found in the posterior lateral field of most nematodes. The function of phasmids remains unclear although a role as receptors for female sex pheromone was proposed for *Scutellonema brachyurum* [62]. Most plant parasitic nematodes have phasmids [6]. Baldwin (1985) identified two types of phasmids in the J2 stage of *H. schachtii*: a larger type that secretes and a smaller one that does not [63]. In proliferating cells of Arabidopsis, AtCDC48 is highly expressed, but it's down-regulated in most differentiated cell types [64]. CDC48/VCP/p97 in Zebrafish has been shown to induce cell proliferation [65]. Based on this discovered we can add phasmids to the set of organs that may play a role in nematode parasitism.

S-phase kinase-associated protein 1 (SKP1) is a key component of the SCF complex that provides ubiquitin-protein ligase activity required for cell cycle progression. Gao *et al.*, (2003) identified a SKP1 homolog in the dorsal gland of *Heterodera glycines* [66]. The SKP1 homologue identified in our study has a nuclear localization signal, and therefore could be potentially targeted to plant nuclei. Translationally-controlled tumor proteins (TCTPs) are highly conserved and are implicated in several different cellular processes including growth, cell cycle progression, malignant transformation, and protection of cells against stress and apoptosis [67]. TCTP proteins are expressed in rapidly growing plant organs, such as the apical meristem, suggesting a role in cell proliferation [68]. Overexpression of TCTP in cultured tobacco cells resulted in faster regeneration and the induction of more calli following *Agrobacterium* infection [69]. We found that the mRNA for secreted TCTP is enriched in the SvG of *M. incognita* (**Figure 4F**) suggesting that TCTP could be actively secreted into the host plant cell.

We observed one Calcium-Dependent Protein Kinase (CDPK) and several CaM proteins in the *M. incognita* secretome. Using RNA interference, Ivashuta *et al.* (2005) showed that in *Medicago truncatula*, CDPK1 is essential for root hair formation and cell elongation [70]. Inactivation of CDPK1 results in significant diminution of Rhizobial and mycorrhizal symbiotic colonization [70]. The CDPK family and signaling pathways are conserved across the plant kingdom [71], so nematodes may have developed the ability to control this central and ubiquitous element of plant development.

We identified several secreted proteins with established or suggested roles in the virulence of parasites. Anand *et al.* (2007) [72] used virus-induced gene silencing and an *in planta* tumorigenesis assay to identify plant genes involved in *Agrobacterium*-mediated plant transformation. They identified several genes that were required to produce the crown gall phenotype; we identified homologs in the *M. incognita* secretome. Among them were SKP1, actin or actin-binding proteins, and histones H3, H2a, and H2b. Histone H2a is required for T-DNA integration [73] and histone H3 has also been implicated [72]. We found a homolog of the Nodulin protein, NodL, in the *M. incognita* secretome, which is similar to the nodulin-like proteins (NLP) required for *Agrobacterium*-mediated transformation [73]. Root knot nematodes induce cytoskeletal changes that closely resemble those induced by Nod proteins [74]. *MtENOD11* is expressed early following both arbuscular mycorrhizal infection and *Meloidogyne* infection of *Medicago* [75]. Therefore, it is possible that root knot nematodes use a Nod-like pathway to initiate giant cell formation.

We were surprised to observe that plant and animal metazoan parasites secrete a common set of proteins. For example, *B. malayi* and *M. incognita* both secrete transthyretin-like protein (TLP or TTL), which is a member of a growing family of transthyretin (TTR)-related proteins (TRPs). TRPs are related to the vertebrate transthyretin, an extracellular thyroid hormone carrier protein [76]. TRPs may represent the ancestor of the vertebrate thyroid hormone carriers [77]. We found in the *M. incognita* secretome a TTL and confirmed that its corresponding transcript is specifically expressed in the SvG of J2 stage nematodes (**Figure 4E**). Therefore, we reason that this TTL homolog is secreted into the plant cell where it regulates growth. A plant TTL is known to interact with the brassinosteroid receptor kinase to control plant growth [78].

### Protein modifications and turn over

Both cysteine (CPI-2) and aspartyl (API-2) protease inhibitor (PI) family members were observed in the *M. incognita* secretome. The function of PIs in nematodes is to protect their intestine from dietary proteases [79]. In plants, endogenous PIs are active against all four classes of proteinase (cysteine-, serine-, aspartyl-, and metallo-). PIs accumulate following wounding or herbivory and they may provide protection [80]. PIs have also been shown to regulate programmed cell death (PCD). For example, synthetic peptide inhibitors of caspases could suppress PCD induced by a *Ps. syringae* infection of tobacco [81]. Recently a cystatin CPI-2 protease inhibitor was identified in *B. malayi* secretions and it was proposed to inhibit host proteases required for antigen processing and presentation [27].

The *M. incognita* secretome contained metallopeptidases, aminopeptidases, a cysteine proteinase, proteasome components, and proteins involved in ubiquitination. Secreted proteases could have two obvious functions: either the destruction of plant defense proteins or nutritional pre-digestion. Cysteine proteinases are involved in both the initiation and execution of the cell death program [82] and intriguingly we found two kinds of cysteine proteinase inhibitors.

*G. rostochiensis* has been shown to secrete metalloproteases [11], as have other phytopathogenic nematodes and free living nematodes; a role in the hatching process was proposed for the latter [83],[11]. Nematode metalloproteases could catalyze protein degradation *in planta* to enable uptake of proteins that are otherwise too large [84].

We identified several ubiquitin proteins in the *M. incognita* secretome. Tytgat *et al.*, (2004) [85] identified a ubiquitin extension protein secreted from the dorsal pharyngeal gland of root cyst nematodes. The ability of pathogens to manipulate the ubiquitination-proteasome system of animal immune systems is known (for a review see Loureiro and Ploegh., 2006) [86]. The ubiquitin pathway is required for innate immunity in *Arabidopsis* [87].

### Proteins with unknown roles

We identified 94 proteins that we were unable to classify (**Table S6**). Among them we had shown that the transcripts of two genes, SEC2 and CL312Contig1_1, are enriched in the SvG of J2 stage *M. incognita* (**Figure 4C** and **4B** respectively). We identified several proteins with a putative function but we were unable to discern a role for them in pathogenicity. One example is a triosephosphate isomerase (TPI) homolog that is highly secreted (ratio secreted/whole = 10.35); the corresponding transcript (CL480Contig2_1; **Figure 4H**) is enriched in the SvG of J2 stage *M. incognita*. BLASP and KOG annotation revealed a putative function of this protein in metabolism. However, a similar TPI was also found in the fungal mammalian pathogen *Paracoccidioides brasiliensis* where TPI localized to the cell wall and cytoplasmic compartments [88]. The authors suggested that TPI is required for interaction between *P. brasiliensis* and the extracellular matrix and could be important for fungal adherence to and invasion of host cells. A similar function could be postulated for the *M. incognita* TPI since after the mobile J2 stage, the parasitic nematode is sedentary and is in close contact with plant tissue.

### Summary

The development of sensitive proteomics methods has allowed us to significantly expand the known secretome of *M. incognita*. A rich set of candidates has been found that can now be functionally evaluated. Conservation of protein sequence allowed us to search our mass spectra using sequence databases from other nematode species and plants. Nearly half (48%) of our identifications from heterologous sequence databases were confirmed by matches to the limited *M. incognita* sequence that is publicly available, suggesting that proteomics can be useful even with nematodes for which no sequence information is available. As more *M. incognita* DNA sequence becomes available, we can probably identify additional proteins by re-searching our mass spectra. We confirmed that most secreted proteins are produced by esophageal glands and we found direct evidence for one secreted by phasmids [2],[34]. Twenty-six proteins overlap between the *M. incognita* and *B. malayi* secretomes (**Table S5**). These include proteins with potential functions in parasitic behavior (e.g., TCTP; Cystatin CPI-2). This remarkable conservation of sequence raises the possibility that plant and animal parasitic nematodes share conserved mechanisms of infection.

## Materials and Methods

### Biological material

*Meloidogyne incognita* was propagated from greenhouse-grown tomato plants (*Solanum esculentum* cv. Rutgers). After 8 weeks of infection, eggs were recovered from tomato plants by shaking *M. incognita*-infected roots in 1∶9 dilution of bleach for 3 min in a flask. Eggs were collected onto a 25 µm mesh and were then bleached twice for 10 minutes with a 1∶5.7 dilution of bleach supplemented with 0.02% Tween 20. Eggs were rinsed four times with sterile ddH_2_O. Twenty million eggs were hatched at room temperature for 3 days in 10 mM Tris pH 7.0 with 300 mg/l carbenicillin (hatching buffer), and juvenile 2 stage (J2) worms were allowed to crawl though five Kimwipe tissue layers into the same hatching buffer. Freshly hatched J2s were washed several times in sterile water and then collected on 8 µm sieves.

Tomato seeds (*Solanum esculentum* cv. Rutgers) were placed above a plastic cylinder filled with cotton fiber and placed into an aerated hydroponics vessel constructed from a 2-liter flask. Hydroponic vessels were supplied with 250 ml sterilized solution of 0.5× Gamborg media basal salts medium complemented with 1× Gamborg vitamins, 0.5% sucrose and 200 mg/l carbenicillin. Tomato plants were maintained under a 16-h photoperiod for 6 weeks and root media was collected and filtered through a 0.22 µm syringe filter to give the “hydroponic tomato root culture solution”.

### Stylet secretion production

Hatched J2s were stimulated for 4 hours by hydroponic tomato root culture solution separated from the nematodes by a 3,500 MW cutoff mini dialysis membrane (Pierce, Rockford, USA). Then they were treated for 4 h with 0.4% resorcinol (Sigma-Aldrich Chimie, St Quentin, France). Stylet secretions were filtered through a 0.22 µm syringe filter to remove nematodes.

### Sample preparation

Secreted proteins were concentrated to ∼1 ml in a vacuum centrifuge at room temperature. Tris buffer was added to a final concentration of 20 mM (pH 7.2). Proteins were reduced and alkylated using 1 mM Tris (2-carboxyethyl) phosphine (Fisher, AC36383) at 65°C for 30 minutes and 2.5 mM iodoacetamide (Fisher, AC12227) at 37°C in dark for 30 minutes, respectively. Proteins were then digested with 1 µg trypsin (Roche, 03 708 969 001) at 37°C overnight.

Whole *M. incognita* worms were lysed in 100 µL 2% (w/v) RapiGest (Waters) by sonicating in a Branson Sonifier 450 fitted with a high intensity cup horn (Part No. 101-147-046, Branson) at 4°C for 2 minutes. Crude lysate was spun down at 16,100 g at 4°C for 5 min. Supernatant was collected and the pellet was discarded. RapiGest was diluted to 0.5% (w/v) by adding 300 µL of 20 mM Tris. Proteins were reduced and alkylated as described above. Protein concentration was measured using a Bradford assay. Protein (400 µg) was digested with 10 µg trypsin (Roche, 03 708 969 001) at 37°C overnight.

TFA (0.5% v/v) was added to each sample to a final pH of 1.8 to precipitate RapiGest after digestion. Samples were incubated at 4°C overnight and then centrifuged at 16,100 g at 4°C for 15 minutes. Supernatants were collected and centrifuged through a 0.22 µM filter to clear any solid particles.

### nanoLC ESI MS/MS analysis

An Agilent 1100 HPLC system (Agilent Technologies, Wilmington, DE) delivered a flow rate of 300 nL min^−1^ to a 3-phase capillary chromatography column through a splitter. Using a custom pressure cell, 5 µm Zorbax SB-C18 (Agilent) was packed into fused silica capillary tubing (200 µm ID, 360 µm OD, 20 cm long) to form the first dimension reverse phase column (RP1). A 5 cm-long strong cation exchange (SCX) column packed with 5 µm PolySulfoethyl (PolyLC) was connected to RP1 using a zero dead volume 1 µm filter (Upchurch, M548) attached to the exit of the RP1 column. A fused silica capillary (100 µm ID, 360 µm OD, 20 cm long) packed with 5 µm Zorbax SB-C18 (Agilent) was connected to SCX as the analytical column (RP2). The electro-spray tip of the fused silica tubing was pulled to a sharp tip with the inner diameter smaller than 1 µm using a laser puller (Sutter P-2000). The peptide mixtures were loaded onto the RP1 column using the custom pressure cell. Columns were not re-used. Peptides were first eluted from the RP1 column to the SCX column using a 0 to 80% acetonitrile gradient for 150 minutes. The peptides were then fractionated by the SCX column using a series of salt gradients (from 10 mM to 1 M ammonium acetate for 20 minutes), followed by high resolution reverse phase separation using an acetonitrile gradient of 0 to 80% for 120 minutes. To avoid sample carry-over and keep good reproducibility, a new set of three columns with the same length was used for each sample.

Spectra were acquired on LTQ linear ion trap tandem mass spectrometers (Thermo Electron Corporation, San Jose, CA) employing automated, data-dependent acquisition. The mass spectrometer was operated in positive ion mode with a source temperature of 150°C. As a final fractionation step, gas phase separation in the ion trap was employed to separate the peptides into 3 mass classes prior to scanning; the full MS scan range was divided into 3 smaller scan ranges (300–800, 800–1100, and 1100–2000 m/z) to improve dynamic range. Each MS scan was followed by 4 MS/MS scans of the most intense ions from the parent MS scan. A dynamic exclusion of 1 minute was used to improve the duty cycle.

MS/MS spectra were collected for secreted and whole *M. incognita* proteins (2,904,233 and 947,474 spectra, respectively). Raw data were extracted and searched using Spectrum Mill (Agilent, version A.03.02). MS/MS spectra with a sequence tag length of 1 or less were discarded. MS/MS spectra were searched against the protein databases (**Table S3**). The enzyme parameter was limited to full tryptic peptides with a maximum mis-cleavage of 1. All other search parameters were set to Spectrum Mill's default settings (carbamidomethylation of cysteines, +/−2.5 Da for precursor ions, +/−0.7 Da for fragment ions, and a minimum matched peak intensity of 50%).

To eliminate redundant protein identifications, proteins with one or more shared peptides were grouped. The numbers of proteins we report in this paper are protein group numbers. A concatenated forward-reverse database was constructed to calculate the *in situ* false discovery rate (FDR) [22]. We used an identification filtering criteria of 0.1% FDR at the peptide level for every search. A total of 486 secreted proteins from the forward database were identified, while 2 proteins (0.4% protein FDR) from the reverse database were identified.

Spectrum counting was used to determine the relative protein amounts in the secretome and the extract from intact nematodes. The number of valid MS/MS spectra from each protein was normalized to the total MS/MS spectra number of each dataset. The normalized spectrum count ratio of each protein (secretome/intact nematode proteome) was used to evaluate whether the protein was enriched in the secretome. The data associated with this manuscript may be downloaded from ProteomeCommons.org Tranche, http://tranche.proteomecommons.org, using the following hash (without the quotes): “FXMi2Tyve1I0DfzhT9FN17TmpNTTDiggs7Njjoh7MYMouHYIx+xUoDILMXFl17RZrVjueXuCZc5c3005l9fdKISeVUEAAAAAAAB0ug =  = ”.

While this paper was under review, Abad et al. [89] reported the draft genome sequence of *M. incognita*. The 9,538 contigs resulting from the *M. incognita* genome assembly and annotation were deposited in the EMBL/Genbank/DDBJ databases under accession numbers CABB01000001–CABB01009538 for release at a future date. When these contigs become publicly available, further bioinformatics analysis of our mass spectra can be conducted to search for additional secreted proteins.

### Functional assignments using BLASTP and KOG

Identified protein sequences were BLASTed against the non-redundant database at NCBI (http://www.ncbi.nlm.nih.gov/). euKaryotic Orthologous Group (KOG) annotations were assigned based on sequence similarity searches against the KOG annotated proteins (http://www.ncbi.nlm.nih.gov/COG/grace/kognitor.html).

Putative nuclear function was assigned based on homologous proteins found using BLASTP or on the identification of a Nuclear Localization Site (NLS). The NLS search was performed using the PredictNLS search engine available at http://cubic.bioc.columbia.edu/predictNLS/ [90].

### *In situ* hybridization

*In situ* hybridizations were performed on freshly hatched J2s as described in Rosso et al. [38]. Briefly, freshly hatched J2s were fixed in 2% paraformaldehyde for 16 h at 4°C and 4 h at room temperature. Nematodes were cut into sections and permeabilized with proteinase K, acetone, and methanol. The sections were then hybridized at 45°C with the sense or antisense probe. Clone and primers are listed in **Table S9**.

## Supporting Information

Table S1 Ranked list of secreted *Meloidogyne incognita* proteins. Proteins are ranked based on the number of unique peptides identified. Proteins are listed according to the protein database that was used for their identification. Proteins that share the same set or subset of peptides are grouped together into protein groups. All proteins were identified by at least two mass spectra using a filtering criterion of 0.1% FDR at the peptide level. A description of each “protein class” is available on Table S6.
(1.32 MB DOC)

Table S2 Ranked list of secreted *Meloidogyne incognita* proteins identified by single peptides. All proteins were identified by at least two mass spectra using a filtering criterion of 0.1% FDR at the peptide level.
(7.87 MB DOC)

Table S3 Protein databases used for protein identification.
(0.03 MB DOC)

Table S4 Deduced amino acid sequences from the *M. incognita* EST library (INRA-Sophia Antipolis; France). Peptides identified from the secretome are in red. Sites that could be recognized by trypsin are underlined.
(0.45 MB DOC)

Table S5 Correspondence between our observations and previous reports of secreted proteins from parasitic nematodes. Proteins are ranked by alphabetical order according to nematode species and then by number of unique peptides per protein.
(0.31 MB DOC)

Table S6 Functional classification of secreted proteins. The secretome was classified into 9 subfamilies (Protein class) using KOG and BLASTP.
(0.07 MB DOC)

Table S7 *Meloidogyne incognita* proteins potentially targeted to the plant nucleus. Three sets of proteins were recognized. The first set contains both an NLS and a DNA binding domain, the second set contains only an NLS, and the third contains only a DNA binding domain. Proteins are listed according to their number of unique peptides. All proteins were identified by at least two mass spectra using a filtering criterion of 0.1% FDR at the peptide level.
(0.19 MB DOC)

Table S8 Plant protein contaminants of the nematode secretome. Plant proteins were identified from root exudates that diffused across the molecular cut-off membrane into the medium for treating nematodes. No nematodes were present. All proteins were identified by at least two mass spectra using a filtering criteria of 0.1% FDR at the peptide level.
(0.04 MB DOC)

Table S9 Oligonucleotide primers that were used to prepare probes for *in situ* hybridization. Two of the probes (calreticulin [1] and β-1,4-endoglucanase [2]) were made based on previous work to serve as an internal control.
(0.08 MB DOC)
