# Supplementary material for: Direct Identification of the Meloidogyne incognita Secretome Reveals Proteins with Host Cell Reprogramming Potential
Source: PLoS Pathog. 2008 Oct 31;4(10):e1000192. doi: 10.1371/journal.ppat.1000192 (PMC2568823; doi:10.1371/journal.ppat.1000192)
Supplement: Table S1 — Ranked list of secreted Meloidogyne incognita proteins. Proteins are ranked based on the number of unique peptides identified. Proteins are listed according to the protein database that was used for their identification. Proteins that share the same set or subset of peptides are grouped together into protein groups. All proteins were identified by at least two mass spectra using a filtering criterion of 0.1% FDR at the peptide level. A description of each “protein class” is available on Table S6. (1.32 MB DOC) [file ppat.1000192.s001.doc]

**Supplementary Table S1:** **Ranked list of secreted *Meloidogyne incognita* proteins.**

| Protein number | *Organism* | Corresponding M. incognita Contig or accession nb. | Number unique peptides | Secretion/Whole Mi - normalized | KOGs | Protein class | BLASTP top hit | E. value | Group members (Accession numbers) |
| --- | --- | --- | --- | --- | --- | --- | --- | --- | --- |
| 1 | *M. incognita* | CL100Contig1_1_AA | 21 | 2.34 | OR | 3 | heat shock protein 70 [Heterodera glycines] | 0 | CL100Contig1_1_AA|12044387|26422391|17541098|3924750|51338753|123585|110433182|156352|4235279|7673686|6941872|39585245|9438176|2104672|156070|MH02643|MI01579|1346316|AC03416|AE00939|AE00172|MI02065|MP00290|156706|156068|159887|AE01363|17534013|175340 |
| 2 | *M. incognita* | CL1Contig12_1_AA | 20 | 0.96 | Z | 1 | Actin | 0 | CL1Contig12_1_AA|CL1Contig5_1_AA|6626|17557190|21264389|3879474|113291|17563820|17563822|3879475|3879477|6624|1439650|1703120|17568985|829164|14278147|51011295|71994099|6628|15011774|17568987|477248|3860544|17551718|3880219|23194503|147907831 |
| 3 | *M. incognita* | CL2154Contig1_1_AA | 18 | 9.83 | G | 4 | enolase [Anisakis simplex] | 9.00E-99 | CL2154Contig1_1_AA|MA01679|MI02132|MI02338|GP00380|MH01493|GR02309|HS00220|51011814|71995829|13937125|14703.m00079|39597532|17536383|2494354|3879986|cr01.Contig2.wum.59.1|25815065|32563855|AC00849|XI02792|AE03626|MH07948|MH02925|MI08623|MH01935 |
| 4 | *M. incognita* | CL2470Contig1_1_AA | 17 | 5.07 | O | 3 | 14-3-3b protein [Meloidogyne incognita] | 2.00E-116 | CL2470Contig1_1_AA|40388674|GR00286|GR00700|MI02312|AE01373|cr01.Contig11.wum.103.1|17568359|2492485|3877418|AC01065|AE00783|39594741|66732615|HC02275|17530147|MP01563|AS03025|50507501|71991223|MH01804|AE00949|HG00334|HG00067|MC05194|AE01892|MI04996 |
| 5 | *M. incognita* | CL14Contig3_1_AA | 17 | 1.19 | J | 6 | Hypothetical protein CBG24566 [C. briggsae] | 9.00E-88 | CL14Contig3_1_AA|CL14Contig1_1_AA|1072161|1255296|1706582|17552884|17569207|80973236|AE01353|CL14Contig2_1_AA|39579274|39593947|39594197|27669314|32566303|6103738|119152|62866511|62866521|62866513|62866519|62866515|62866517|MA01825|AE01370 |
| 6 | *M. incognita* | CL2Contig2_1_AA | 16 | 2.46 | C | 4 | arginine kinase [Heterodera glycines] | 6.00E-125 | CL2Contig2_1_AA|CL2Contig16_1_AA|MI02629|MJ00387|CL2Contig6_1_AA|MI02256|MH02673|MA01152|MI01640|MP01465|MP00405|HG00839|CL1797Contig1_1_AA|HC04049|AE03500|CL2Contig9_1_AA|HG00296|HG00215|HG00783|HS00393|HG00180|MH02862|PP00600 |
| 7 | *M. incognita* | CL673Contig1_1_AA | 16 | 2.92 | O | 3 | protein disulfide isomerase [Ancylostoma caninum] | 4.00E-78 | CL673Contig1_1_AA|MH00909|AS16616|MC00432|39919142|39919144|SS01561|687235|16945685|86161652|14972.m07552|OS00811|OS01836|SS00165|HC01304|MH02103|GR00230|PT00448|AS00959|AS01707|TX01085|BM00118|MI07274|GR00257|AS09688 |
| 8 | *M. incognita* | CL1067Contig1_1_AA | 15 | 0.46 | Z | 1 | tropomyosin [Heterodera glycines] | 2.00E-68 | CL1067Contig1_1_AA|33333845|GR00832|MJ04971|PE00209|MI02523|HS00425|HG00906|PE00234|MC00914|MH07965|MH02432|42559676|321012|136098|161677|1208413|15718291|42559735|71992941|1208409|GP00317|42559665|MC04602|MI06734|773389|17646751|2072534|42559553|161 |
| 9 | *M. incognita* | CL289Contig1_1_AA | 15 | 3.8 | O | 3 | Hsp90 [Heterodera glycines] | 0.9381 | MH02599|40956306|MA00525|MC00861|MA01245|19855062|3256076|3096951|11640609|47559088|47559090|MA02284|MI04564|MA00121|CL289Contig1_1_AA|74847037|17559162|3875041|74963152|MJ04742|GR00658|39589853|MJ02702|14992.m11078|144228163|MJ01828|51861123|MI02976 |
| 10 | *M. incognita* | 207a11c1.1_1_AA | 14 | 9.02 | O | 3 | calreticulin [Meloidogyne incognita] | 2.00E-58 | 17530149|207a11c1.1_1_AA|207a11r1.1_1_AA|MI02454 |
| 11 | *M. incognita* | CL5Contig2_1_AA | 14 | 14.2 | ? | 9 | SEC-2 protein [Globodera pallida] | 1.00E-45 | MA00545|MA00575|MI02617|MI02633|MJ00851|MA00573|MI02522|MJ00831|MP00598|MP00683|CL5Contig2_1_AA|CL5Contig1_1_AA|MI01705|MH02761|MJ04510|MH01097|MJ04357|MJ03774|MA02843|MH05406|MA02986|MI03753|MH07198|MH07718|MH08616|MA03309|MH01816 |
| 12 | *M. incognita* | CL78Contig1_1_AA | 13 | 1.21 | Z | 1 | alpha-tubulin MEC-12 [C. elegans] | 0 | CL78Contig1_1_AA|11874732|14573940|17554312|1854669|2401255|63029700|1729842|477265|18250220|25150752|60549977|159155|39580244|39597706|62546341|MH02406|39595426|cr01.Contig74.wum.24.1|1096149|1405416|17509355|3876416|303528|3874966|464842|71982026 |
| 13 | *M. incognita* | CL312Contig1_1_AA | 13 | 2.04 | ? | 9 | No significant similarity found | No | CL312Contig1_1_AA|CL1Contig22_1_AA|MI03301|MH08063|MH01803 |
| 14 | *M. incognita* | CL174Contig1_1_AA | 12 | 6.44 | G | 4 | glyceraldehyde-3-phosphate-dehydrogenase [Globodera rostochiensis] | 4.00E-155 | CL174Contig1_1_AA|MI02487|MI02562|CL174Contig2_1_AA|MI02559|MI00212|MA00494|MH02558|MH00555|MC00207|MJ00491|MA02128|MA02573|MA01345|MJ00076|MA01086|GP03356|MI00448|MP01989|2293134|6016070|MH06174|MP01986|MH00554|39597690|417018|120646|6744|12060 |
| 15 | *M. incognita* | CL28Contig1_1_AA | 11 | 0.08 | Z | 1 | Hypothetical protein CBG23416 [C. briggsae] | 3.00E-125 | CL28Contig1_1_AA|39592202|cr01.Contig1.wum.332.1|127737|32566139|33300386|6799|GR00838|MI01764|MI03844|MC01153|MI02292|MH02583|MJ04760|MA03051|MI01769|MC03252|MH07342|MC04270|MC03948|MC01919|MC01292 |
| 16 | *M. incognita* | CL1191Contig1_1_AA | 10 | 4.37 | O | 3 | Cell Division Cycle related family member (cdc-48.1) | 1.00E-92 | CL1191Contig1_1_AA|cr01.Contig2.wum.540.1|39587267|17532375|3874892|3875715|6226902|MI00204|MA01267|MH06053|AE01815|GR01850|GP02038|HS01078|MP00640|XI00601 |
| 17 | *M. incognita* | CL480Contig2_1_AA | 10 | 10.35 | G | 4 | Hypothetical protein CBG21017 [C. briggsae] | 2.00E-89 | CL480Contig2_1_AA|MI02324|MJ00169|CL480Contig1_1_AA|MI08897|MH04477|MP02232|14940.m00172 |
| 18 | *M. incognita* | CL321Contig1_1_AA | 10 | 2.01 | DZ | 1 | Translationally-controlled tumor protein homolog (TCTP) [C. briggsae] | 2.00E-64 | CL321Contig1_1_AA|MI01566|MI01662|MJ01483|MA01239|MA01287|MA02447|MC00901|PE00058 |
| 19 | *M. incognita* | MI02657 | 10 | 40.94 | ? | 9 | hypothetical protein [Neurospora crassa OR74A] | 0.11 | MA00417|MI02657|MA00412|MJ03923|PT00685|MJ01800|PT00616|MP00684|MJ01407|MJ03217|MI02705|MI05162 |
| 20 | *M. incognita* | CL107Contig1_1_AA | 9 | 3.5 | G | 4 | aldolase [Heterodera glycines] | 4.00E-102 | CL107Contig1_1_AA|MI01894|MP00554|MI01017|MI02212|CL107Contig2_1_AA|MI08503|MI00246|MH08785|MI01982|20163188|38046603|26419333|38231223|HS00385|GP02969|45758650|45643643|45643645|45758654|45643649|45643647|MJ04041|MA02860 |
| 21 | *M. incognita* | CL12Contig1_1_AA | 9 | 14.32 | T | 5 | hypothetical protein [Bos taurus] | 9.00E-82 | CL12Contig1_1_AA|CL12Contig2_1_AA|CL12Contig3_1_AA|NB01099|AS02137|GP00259|17564542|2291247|29726960|39588502|4160167|55976211|AC00968|AE00979|GR00767|cr01.Contig21.wum.111.1|MC00704|MJ01181|14992.m10856|MA02717|MH01947|NB00618|HS01093 |
| 22 | *M. incognita* | CL349Contig1_1_AA | 9 | 6.55 | OT | 3;5 | Hypothetical protein CBG18253 [C. briggsae] | 5.00E-46 | CL349Contig1_1_AA|MP01282|MI01649|MJ05064|MI06517|MH02495|MA00770|MC00315 |
| 23 | *M. incognita* | CL809Contig1_1_AA | 8 | 4.89 | O | 3 | Cell Division Cycle related family member (cdc-48.2) [C. elegans] | 2.00E-60 | CL809Contig1_1_AA|MP01852|MA02657|XI04846|XI03814|1729896|17531535|3874005|cr01.Contig2.wum.168.1|39597425|PP00529|AE04337|15264.m00007|SS01112|MI03466 |
| 24 | *M. incognita* | CL213Contig1_1_AA | 8 | 1.87 | J | 6 | 40S ribosomal protein S4 [Ixodes scapularis] | 3.00E-94 | CL213Contig1_1_AA|MI01528|MI01825|MA01742|MJ01464|MH02435|MP00809|MI02669|MC00892|RS00010|HG00673|GR00528|GP03213|MI03709|MH09703|PP00601|13260.m00097|AS02609|SR01007|PT00758|AS02866|AS01972|SS01487|SS00026|AS16449|AS07662 |
| 25 | *M. incognita* | CL98Contig1_1_AA | 8 | 1.15 | O | 3 | Hypothetical protein CBG24861 [C. briggsae] | 1.00E-29 | CL98Contig1_1_AA|MI02295|MI00420 |
| 26 | *M. incognita* | 220d23r1.1_1_AA | 8 | 1.09 | ? | 8 | pectate lyase 3 [Meloidogyne incognita] | 2.00E-155 | 57792469|220d23r1.1_1_AA|CL2624Contig1_1_AA |
| 27 | *M. incognita* | MI08392 | 8 | 17.02 | R | 3 | Hypothetical protein CBG20552 [C. briggsae] | 2.00E-69 | MP01526|MI08392|MJ02071|MH02533|MI04178 |
| 28 | *M. incognita* | MI02513 | 8 | 7.64 | P | 4;7 | cytoplasmic Cu/Zn-superoxide dismutase [Brugia pahangi] | 2.00E-48 | MI02513|MI02469|MI01350|MI03511|MH00939|MC00554|MH02613 |
| 29 | *M. incognita* | CL2Contig18_1_AA | 7 | 1.07 | Z | 1 | hypothetical protein F53A9.10 - C. elegans | 3.00E-57 | CL2Contig18_1_AA|CL2Contig14_1_AA|MI02592|MI02352|MP01136|MI03046|MH02541|MA00922|CL2Contig3_1_AA|MJ03757|MA00497|MI01979|211c12c1.1_1_AA|CL2Contig19_1_AA|CL2Contig15_1_AA |
| 30 | *M. incognita* | CL114Contig1_1_AA | 7 | 1.13 | Q | 4;7 | Hypothetical protein CBG13395 [C. briggsae] | 2.00E-26 | CL114Contig1_1_AA|MI02012|MJ05041|MJ02027|MA02137|MH02863 |
| 31 | *M. incognita* | CL41Contig1_1_AA | 7 | 1.2 | C | 4 | R11A5.4a [C. elegans] | 4.00E-106 | CL41Contig1_1_AA|MI04253|MC00216|CL41Contig2_1_AA|MH02352|MI04457|MI02415|MI03664|3879140|71989645|39582104|cr01.Contig242.wum.5.1|42734243|71989658|42734242|71989653|42734244|71989649|MI03895|AS08262 |
| 32 | *M. incognita* | CL47Contig1_1_AA | 7 | 0.35 | Z | 1 | Hypothetical protein CBG24046 [C. briggsae] | 1.00E-56 | CL47Contig1_1_AA|MA00546|MI02538|MI02595|MP00513|MH02730|MJ00682|MJ00594|MH02838|MI05197|MI08109|MH02837|MI05734|GR00822|MC01211|MH07187|HS00707|HC01638|cr01.Contig5.wum.351.1|39579600|156370|1709055|25151365|485116|AS02561|AE00045|AE00678|MH03169 |
| 33 | *M. incognita* | CL333Contig1_1_AA | 7 | 4.28 | J | 6 | ribosomal protein L7 [Crassostrea gigas] | 4.00E-74 | CL333Contig1_1_AA|MA01606|MI02217|MI00075|MC00318|MH02024|GP02234|GR00793|MJ00170|MP00579|MC00366 |
| 34 | *M. incognita* | CL822Contig1_1_AA | 7 | 1.72 | W | 9 | Hypothetical protein CBG02518 [C. briggsae] | 9.00E-68 | CL822Contig1_1_AA|MC01639|MC00254|MH08409|MH03301 |
| 35 | *M. incognita* | CL130Contig2_1_AA | 7 | 2.09 | ? | 9 | LL20 15kDa ladder antigen | 1.00E-05 | CL130Contig2_1_AA|CL130Contig1_2_AA|MH06844 |
| 36 | *M. incognita* | CL2605Contig1_1_AA | 6 | 1.93 | Z | 1 | beta-tubulin [Haemonchus contortus] | 0 | CL2605Contig1_1_AA|226737|32697980|71983645|897749|CL608Contig1_1_AA|159161|10179942|1217658|124244617|124244619|159159|159157|125968515|125968520|146424423|74763811|439735|2501421|135473|1572737|17569053|6892|17553980|3924793|1174596|17549915 |
| 37 | *M. incognita* | CL1607Contig1_1_AA | 6 | 4.85 | ? | 9 | No significant similarity found | No | MJ00780|CL1607Contig1_1_AA|MI06837|MI06500|MP00199|MJ00086|MI06486 |
| 38 | *M. incognita* | 214n04r1.1_1_AA | 6 | 4.95 | R | 7 | Hypothetical protein CBG00891 [C. briggsae] | 3.00E-44 | MJ00119|MJ00537|MI01318|MI02490|214n04r1.1_1_AA|MJ00761|MC00564|MP02107|MJ01478|PE00163|HG00704|MH02532|MH02088 |
| 39 | *M. incognita* | CL843Contig1_1_AA | 6 | 3.85 | A | 2 | Hypothetical protein CBG22235 [C. briggsae] | 2.00E-89 | CL843Contig1_1_AA|MI08267|MA00496|MI01140|MC00836|MH09146|MJ01047 |
| 40 | *M. incognita* | CL1Contig68_1_AA | 6 | 15.28 | O | 3;7 | glutathione S-transferase-1 [Meloidogyne incognita] | 6.00E-114 | MI02372|MJ01358|MP01600|215m02r1.1_1_AA|MI02449|CL1Contig24_1_AA|MJ02723|MI04402|CL1Contig68_1_AA|206d07r1.1_1_AA|MI08730 |
| 41 | *M. incognita* | CL68Contig1_1_AA | 6 | 0.69 | Z | 1 | Calponin protein 3 [C. elegans] | 1.00E-46 | CL68Contig1_1_AA|MI02270|MI02152|MJ00518|MJ00729|MJ00809|MP00642|MH02735|MI05861|207n07c1.1_1_AA|MJ01523|MH02836|MH09345 |
| 42 | *M. incognita* | CL1Contig27_1_AA | 6 | 1.49 | ? | 8 | beta-1,4-endoglucanase [Meloidogyne incognita] | 0 | 40037070|CL1Contig27_1_AA|MI02610|5639669|13398418|125716438|CL1Contig21_1_AA|40037077|MA03107|221m13c1.1_1_AA|MA00503|MC00710|CL1Contig52_1_AA |
| 43 | *M. incognita* | CL2084Contig1_1_AA | 6 | 27.5 | O | 3;7 | Hypothetical protein CBG02268 [C. briggsae] | 1.00E-40 | MI02263|CL2084Contig1_1_AA|MA00182|MI09097|CL66Contig2_1_AA|MI01397|CL66Contig1_1_AA|MJ02976|MJ03725|MA00038|MH02526|MA00574|MI02597|MP01144|GR00579|GP02055|MH02773|MC01116|MH10161|MH11086|MC01370|MH09750|MH10717|MH02455|MJ01918|MH01948 |
| 44 | *M. incognita* | CL3Contig3_1_AA | 6 | 0.44 | Z | 1 | putative calponin [Meloidogyne incognita] | 0 | CL3Contig3_1_AA|12697208|MI02660|MI02710|MA00588|MH02769|MJ02358|MI02661|MA00550|MA00026|MA00603|CL3Contig1_1_AA|114152904|3886093|498330|71983400|7511668|50058068|71983410|14574042|498328|498331|71983406|498329|498332|MA00589|MJ00875|MJ00878 |
| 45 | *M. incognita* | CL2882Contig1_1_AA | 6 | 2.21 | O | 3 | Hypothetical protein CBG21516 [C. briggsae] | 3.00E-92 | CL2882Contig1_1_AA|MI08534|MC02110|HC04960|MJ02279|115533048|82775242|115533046|3881186|405831|cr01.Contig1282.wum.2.1|cr01.Contig269.wum.11.1|39583450|AS02303|AE04352|XI01123|SR00060|AC04870|SR00044 |
| 46 | *M. incognita* | CL1451Contig1_1_AA | 6 | 2.15 | ? | 9 | Hypothetical protein CBG07652 [C. briggsae] | 4.00E-65 | CL1451Contig1_1_AA|MP00435|MH10615 |
| 47 | *M. incognita* | 219i04r1.1_1_AA | 6 | 2.26 | B | 2 | similar to heterochromatin protein 1, binding protein 3 [Equus caballus] | 2.00E-60 | 219i04r1.1_1_AA|1708108|17567723|17556046|3979986|75025386|MA02682|MH11226|PE00084|12698.m00326|MC04780|6686276|17532993|17534745|17534753|17537813|17540652|17541088|17559288|17561992|17561998|17562010|17564214|3873702|3875619|3876195|3877574|3879732 |
| 48 | *M. incognita* | CL578Contig1_1_AA | 6 | 2.82 | J | 6 | small subunit ribosomal protein 8 [Koerneria sp. RS1982] | 2.00E-79 | CL578Contig1_1_AA|MP00552|MI02433|MJ00739|MP00019|MI01620|MI02917|MH02605|MC01089|MA01847|GP01153|AS03160|GR00642|AE00308|AS15523|AE01316|NB01157|HC02126|HC02151|PE00088|PE00121|MI08674|MI00178|MJ03479|HG00553|AC00915|1065942|1351011|17542014|AE00150 |
| 49 | *M. incognita* | CL2889Contig1_1_AA | 6 | 2.01 | J | 6 | similar to tax-responsive element binding protein 107 [Ornithorhynchus anatinus] | 2.00E-38 | CL2889Contig1_1_AA|MI00223|ZP00069|14979.m04592|PE00094|DI02498|MH03663|MH03699|MH04761|AS02925|AS02659|PE00168|AS15498|AS00277|MC00521|GR00654|GP00242 |
| 50 | *M. incognita* | CL406Contig1_1_AA | 6 | 0.62 | CE | 4 | Hypothetical protein CBG22943 [C. briggsae] | 3.00E-82 | CL406Contig1_1_AA|MI02504|MA02496|MA00109|MI00556 |
| 51 | *M. incognita* | CL1779Contig1_1_AA | 6 | 0.47 | C | 4 | GEX Interacting protein family member (gei-7) [C. elegans] | 2.00E-70 | CL1779Contig1_1_AA|212b10r1.1_1_AA|MA00488|MJ04009|MA01674 |
| 52 | *M. incognita* | CL2662Contig1_1_AA | 5 | 5.44 | T | 5;7 | Y57A10A.26 [C. elegans] | 6.00E-45 | CL2662Contig1_1_AA|PT00605|MJ00559|MI00218|MJ00007|MP00680|MI05856|CL614Contig1_1_AA|39591426|NB02271|17537617|5832943|HG01040|MH02461|MH08007|GR01919 |
| 53 | *M. incognita* | 220i21c1.1_1_AA | 5 | 1.99 | O | 3 | chaperonine protein HSP60 [Onchocerca volvulus] | 1.00E-68 | 220i21c1.1_1_AA|MA01915 |
| 54 | *M. incognita* | 206k03r1.1_1_AA | 5 | 1.13 | C | 4 | ATP synthase subunit family member (atp-2) [C. elegans] | 0 | 7436125|cr01.Contig139.wum.26.1|17976515|21431750|25144756|AE00387|AC03063|MH01836|AC03397|SR00949|206k03r1.1_1_AA|AC03030|14980.m02712|39586537|AE01771|HC05986|HG01358|MP00209|PT01519|MI01627|SR03257|AS02001|AC03595|XI04703|XI04888 |
| 55 | *M. incognita* | CL999Contig1_1_AA | 5 | 1.4 | K | 2 | elongation factor 1-beta [Ixodes scapularis] | 6.00E-38 | MA02262|CL999Contig1_1_AA|MI01026 |
| 56 | *M. incognita* | CL543Contig1_1_AA | 5 | 2.04 | O | 3 | Hsp90 [Heterodera glycines] | 2.00E-110 | CL543Contig1_1_AA|85540596|133952901|85540598|85540602|133952903|133952906|85540600|85540604|85540606|133952909|AS02212|AE00511|AS15524|133952891|85701365|SR01606|SS00740|NB01011|GP01998|HC00327|XI01101|HC00330|TX01759|MH00309|DI02266|85540594 |
| 57 | *M. incognita* | 209b10c1.1_1_AA | 5 | 13.09 | R | 3 | Chain A, Structure Of C. Elegans Leucine Aminopeptidase (Lap1) | 3.00E-28 | 209b10c1.1_1_AA |
| 58 | *M. incognita* | CL36Contig1_1_AA | 5 | 0.26 | Z | 1 | UNCoordinated family member (unc-27) [C. elegans] | 5.00E-36 | CL36Contig1_1_AA|MI02495|MP00517|MA00591|MI01978|MA00549|MJ00609|MJ00690|MI00790|MI04352|MJ00348|MJ00459 |
| 59 | *M. incognita* | 202g16c1.1_1_AA | 5 | 1.63 | G | 4 | Hypothetical protein CBG05956 [C. briggsae] | 7.00E-71 | 202g16c1.1_1_AA|MA01869|219n02r1.1_1_AA|cr01.Contig20.wum.111.1|17506831|3876398|17506829|3876399|71984413|74834711|39580705|38422281|71984406|AE02746|14971.m02822|XI03072|MA02972 |
| 60 | *M. incognita* | CL2297Contig1_1_AA | 5 | 2.89 | J | 6 | Hypothetical protein CBG09153 [C. briggsae] | 9.00E-73 | CL2297Contig1_1_AA|MJ00145|MC00561|MI02072|MP00256|MI06332|MA01756|MH02232|MC04731|MC05114|GR00662|PE00067 |
| 61 | *M. incognita* | CL1432Contig1_1_AA | 5 | 1.9 | O | 3 | peptidyl-prolyl cis-trans isomerase [Brugia malayi] | 2.00E-41 | MI00211|CL1432Contig1_1_AA|MI02534|MJ01280|MP00609|MI01089|MH02296|MI03722|MI03700 |
| 62 | *M. incognita* | CL2981Contig1_1_AA | 5 | 8.25 | J | 6 | Similar to ribosomal protein L15 isoform 1 [Homo sapiens] | 1.00E-76 | CL2981Contig1_1_AA|MJ01471|MI00160|MI00051|MC01030|MH02628|MA02356|14544.m00114|GR00819|RS00094|AS13249|GP00370|AS03011|DI02128|AS02201|AS01501|AS02835|AS00321 |
| 63 | *M. incognita* | CL315Contig1_1_AA | 5 | 1.7 | C | 4 | Hypothetical protein CBG18957 [C. briggsae] | 4.00E-73 | CL315Contig1_1_AA|AS03132|AS03026|MA02459|OS04191|HC00594|ZP00123|AC03314|MI02361|MI01383|PP00284 |
| 64 | *M. incognita* | CL2887Contig1_1_AA | 5 | 2.8 | G | 4 | F25H5.3b [C. elegans] | 3.00E-71 | CL2887Contig1_1_AA|MC03161 |
| 65 | *M. incognita* | CL53Contig1_1_AA | 5 | 5.64 | J | 6 | Ribosomal Protein, Large subunit family member (rpl-5) [C. elegans] | 9.00E-91 | MA02294|CL53Contig1_1_AA|MH02336|MI01881|MI02190|MI02308|MH02902|MC00610|MH02973 |
| 66 | *M. incognita* | CL2Contig8_1_AA | 5 | 2.62 | W | 9 | lectin, galactoside-binding, soluble, 9 isoform 2 [Bos taurus] | 7.00E-13 | CL2Contig8_1_AA|CL2Contig21_1_AA|MI02539|MA02941|MI01410|MI04146 |
| 67 | *M. incognita* | 206d07c1.1_1_AA | 5 | 4.01 | O | 3;7 | glutathione S-transferase-1 [Meloidogyne incognita] | 1.00E-93 | 206d07c1.1_1_AA|MI03245|MH02442 |
| 68 | *M. incognita* | CL19Contig2_1_AA | 5 | 0.89 | T | 5 | cAMP-dependent protein kinase [Onchocerca volvulus] | 2.00E-42 | CL19Contig2_1_AA|CL19Contig1_1_AA|MJ03176|MI02413|MH07236|MI02883|MI00332|GR00531|MA02373|MA02446|MI04852|MA01349|MI05519|MA00976|MI00636|MA01279|MI02658|MI02659|MA02131|MI02644|MI02302|MA00584|MI00632|MI02713|MI05068|MI00038|MI00769|MI05368 |
| 69 | *M. incognita* | 205o21r1.1_1_AA | 5 | 1.43 | J | 6 | Hypothetical protein CBG02260 [C. briggsae] | 3.00E-101 | 205o21r1.1_1_AA|MH02657|MA00425|MC00989|MI02219|MP00634|MI02320|MP00447|MA00921|MH00482 |
| 70 | *M. incognita* | CL423Contig1_1_AA | 5 | 4.28 | ? | 9 | unnamed protein product [Aspergillus oryzae] | 0.23 | CL423Contig1_1_AA|MA00772 |
| 71 | *M. incognita* | CL623Contig1_1_AA | 5 | 2.39 | J | 6 | 40S ribosomal protein S2 [Urechis caupo] | 3.00E-79 | CL623Contig1_1_AA|MI02394|MP01467|MJ01709|MI01144|MP00533|MP00572 |
| 72 | *M. incognita* | CL930Contig1_1_AA | 5 | 3.27 | J | 6 | similar to 40S ribosomal protein S6 [Tribolium castaneum] | 1.00E-73 | MI02510|MI01874|MA00410|MH02031|CL930Contig1_1_AA|MP02036|MC00816|PE00248|RS00489|MI02664 |
| 73 | *M. incognita* | CL613Contig1_1_AA | 5 | 3.06 | K | 2 | Hypothetical protein CBG04320 [C. briggsae] | 9.00E-49 | CL613Contig1_1_AA|MI00872|MC04781 |
| 74 | *M. incognita* | CL2128Contig1_1_AA | 5 | 2.04 | J | 6 | 60S ribosomal protein L3 | 0 | CL2128Contig1_1_AA |
| 75 | *M. incognita* | CL1662Contig1_1_AA | 5 | 2.7 | T | 5 | protein phosphatase 2 (formerly 2A), regulatory subunit A, beta isoform [Danio rerio] | 6.00E-62 | CL1662Contig1_1_AA|MH08724|MH03745 |
| 76 | *M. incognita* | CL1471Contig1_1_AA | 5 | 0.13 | Z | 1 | myosin heavy chain [Haemonchus contortus] | 2.00E-61 | MI04341|CL1471Contig1_1_AA |
| 77 | *M. incognita* | CL1656Contig1_1_AA | 5 | 1.76 | O | 3 | Hypothetical protein CBG06014 [C. briggsae AF16] | 4.00E-126 | CL1656Contig1_1_AA |
| 78 | *M. incognita* | MI00148 | 5 | 4.07 | O | 3;7 | Hypothetical protein CBG02268 [C. briggsae] | 1.00E-39 | MI00148|MI02106|MI00112|MJ00142|MJ00037 |
| 79 | *M. incognita* | MI01715 | 5 | 0.59 | E | 4 | Hypothetical protein CBG01953 [C. briggsae] | 8.00E-54 | MP01114|MI01715|MH07533|MH01647|MH11011 |
| 80 | *M. incognita* | MI02284 | 5 | 2.55 | G | 4 | Probable inositol monophosphatase (IMPase) | 8.00E-52 | MI02284|MC00727|MI00197 |
| 81 | *M. incognita* | CL227Contig1_1_AA | 4 | 0.98 | OR | 3 | UBiQuitin family member (ubq-1) [C. elegans] | 0 | CL227Contig1_1_AA|25151716|32564339|17554758|3881474|136665|CL241Contig1_1_AA|226k01c1.1_1_AA|CL728Contig1_1_AA|212p17r1.1_1_AA|24418263|MI02546|MJ00836|MI00143|MP01673 |
| 82 | *M. incognita* | CL860Contig1_1_AA | 4 | 3.9 | O | 3 | Hypothetical protein CBG09267 [C. briggsae] | 2.00E-155 | CL860Contig1_1_AA|MH02398|PP01033|MA01297|MI08215|MJ00426|HS00131|39593059|cr01.Contig13.wum.23.1|17563250|2315358|3122624|14992.m11288|TS03879|SR02646|OS00450 |
| 83 | *M. incognita* | CL375Contig1_1_AA | 4 | 2.91 | T | 5 | RACK1 (mammalian Receptor of Activated C Kinase) [C. elegans] | 5.00E-91 | CL375Contig1_1_AA|MI01882|MP00013|MP00429|MA00158|MP01074|MI01200|MJ00370|MI09151|14972.m06982|AS03219|DI00127|BM00042|MH02655|MA02265 |
| 84 | *M. incognita* | CL928Contig1_1_AA | 4 | 3.06 | J | 6 | Y-box factor homolog (APY1) [Aplysia californica] | 8.00E-27 | MI00877|CL928Contig1_1_AA|MI01057|MI00054 |
| 85 | *M. incognita* | CL1Contig35_1_AA | 4 | 1.35 | O | 3 | cyclophilin [Steinernema carpocapsae] | 3.00E-77 | CL1Contig35_1_AA|CL1Contig14_2_AA|MJ00094|MP01502|MI02067|MH02707 |
| 86 | *M. incognita* | CL79Contig2_1_AA | 4 | 0.59 | W | 9 | beta-galactoside-binding lectin [Onchocerca volvulus] | 5.00E-108 | CL79Contig2_1_AA|CL79Contig1_1_AA|MC00842|MJ03450|MA00712|MH02645|PE00147|MI01813|MI02491|MI01226|HG02020|MI04516 |
| 87 | *M. incognita* | CL604Contig1_1_AA | 4 | 3.27 | J | 6 | ribosomal protein S5 [Crassostrea gigas] | 2.00E-92 | CL604Contig1_1_AA|MI00088|MI01858|MC00911|MH02636|RS00638|SS01463|SR01030|PT03073|AS00900|MP00010|MI00005|AS03193|PP00615|NB01165|AE01259|AE01341|39593711|cr01.Contig1710.wum.2.1|cr01.Contig32.wum.82.1|1351001|17542204|3879461|AC02987|AC03047|AS03354 |
| 88 | *M. incognita* | CL1Contig23_1_AA | 4 | 4.58 | ? | 9 | No significant similarity found | No | CL1Contig23_1_AA|MI02249|MP01021|MP00934 |
| 89 | *M. incognita* | 203i02r1.1_1_AA | 4 | 2.16 | E | 4 | Hypothetical protein CBG13820 [C. briggsae] | 2.00E-91 | 203i02r1.1_1_AA|CL967Contig1_1_AA|MI00633|MH09091 |
| 90 | *M. incognita* | CL320Contig2_1_AA | 4 | 6.35 | I | 4 | embryonic fatty acid-binding protein Bm-FAB-1 [Brugia malayi] | 5.00E-29 | CL320Contig2_1_AA|MJ00535|MA02647|MI02389|MI01078|CL320Contig1_1_AA|MH02592|MP00343|215p01r1.1_1_AA |
| 91 | *M. incognita* | CL793Contig1_1_AA | 4 | 3.16 | ? | 9 | prophage LambdaSa2, PblB, putative [Streptococcus agalactiae CJB111] | 2.8 | CL793Contig1_1_AA|MA00482|MI01296|MA03073|MA00458|MJ00050|MA03153 |
| 92 | *M. incognita* | 214g02r1.1_1_AA | 4 | 0.77 | E | 4 | Hypothetical protein CBG01953 [C. briggsae] | 2.00E-54 | 214g02r1.1_1_AA|MI00608|MI00393|216a13c1.1_1_AA |
| 93 | *M. incognita* | CL124Contig1_1_AA | 4 | 0.72 | J | 6 | peroxiredoxin [Globodera rostochiensis] | 6.00E-88 | CL124Contig1_1_AA|MI02613|PT00761|MI02462|MP00535|MH01325|MA00411|MJ00986|MA03144|MI00671 |
| 94 | *M. incognita* | CL2714Contig1_1_AA | 4 | 1.38 | G | 4 | Hypothetical protein CBG21737 [C. briggsae] | 1.00E-72 | CL2714Contig1_1_AA|MA01923|MH01878|MI04319|MH08848|MA03223|MI04510|25319277|103421843|14530390|17539424|66774192|115530061|133901790|14972.m06957|17507723|3877766|9087145|103421849|14530391|17539422|39586424|115530060|133901788|cr01.Contig43.wum.55.1 |
| 95 | *M. incognita* | 206c09r1.1_1_AA | 4 | 2.48 | G | 4 | Hypothetical protein CBG18265 [C. briggsae] | 1.00E-77 | 206c09r1.1_1_AA|MI02498|MH05459 |
| 96 | *M. incognita* | CL610Contig1_1_AA | 4 | 1.26 | J | 6 | ribosomal protein P2 [Brugia malayi] | 1.00E-15 | CL610Contig1_1_AA|MJ01587|PT04358|MJ05143|MP00534|MI02386|MI02518|MJ01196|MI03349|MJ00904|MI06845|MC05042|MH02440|MC01162|MC05656|MH06686 |
| 97 | *M. incognita* | CL2812Contig1_1_AA | 4 | 1.7 | I | 4 | Lipid Binding Protein family member (lbp-5) [C. elegans] | 6.00E-26 | CL2812Contig1_1_AA|MP00028|MP00671|MI01946|MJ01203|MI00824|MP02048 |
| 98 | *M. incognita* | CL110Contig1_1_AA | 4 | 5.31 | J | 6 | large subunit ribosomal protein 2 [Koerneria sp. RS1982] | 1.00E-110 | CL110Contig1_1_AA|MJ00167|MI02311|MI01995|SR01039|SR03374|MH01685|MC04879|SS01205|MI01385|MP02051|MC02307 |
| 99 | *M. incognita* | CL13Contig6_1_AA | 4 | 1.83 | S | 9 | vap-1 [Heterodera glycines] | 6.00E-35 | MI01295|CL13Contig6_1_AA|MP00225|MI04454|CL13Contig1_1_AA |
| 100 | *M. incognita* | CL225Contig1_1_AA | 4 | 0.11 | Z | 1 | myosin heavy chain [Haemonchus contortus] | 2.00E-26 | CL225Contig1_1_AA|MC03610 |
| 101 | *M. incognita* | 203j07c1.1_1_AA | 4 | 2.89 | ? | 9 | F40F8.11 [C. elegans] | 2.00E-10 | 203j07c1.1_1_AA|MP00997 |
| 102 | *M. incognita* | 209h03r1.1_1_AA | 4 | 1.83 | G | 4 | independent phosphoglycerate mutase isoform 1 [Brugia malayi] | 0 | 56646763|56646765|209h03r1.1_1_AA|209h03c1.1_1_AA|MI05984|MI00279|MI03421 |
| 103 | *M. incognita* | CL1120Contig1_1_AA | 4 | 2.04 | J | 6 | Hypothetical protein CBG17994 [C. briggsae] | 3.00E-85 | CL1120Contig1_1_AA|MP00658|MI02488|MI02116|MJ01869|MH08392|MI01365 |
| 104 | *M. incognita* | CL212Contig1_1_AA | 4 | 1.83 | J | 6 | large subunit ribosomal protein 10 [Pristionchus sp. 10 RS5133] | 7.00E-98 | CL212Contig1_1_AA|MP00440|MI02429|MI02635|MJ00737|MJ00014|MA00215|MC01155|MH02433|GR00811|HS01590|GP00348|MH11022|MH10541|MC03495 |
| 105 | *M. incognita* | CL983Contig1_1_AA | 4 | 3.06 | O | 3 | T complex protein [Drosophila melanogaster] | 4.00E-30 | CL983Contig1_1_AA|MH00604|MP00516|MA02491 |
| 106 | *M. incognita* | CL737Contig1_1_AA | 4 | 4.12 | ? | 9 | hypothetical protein DDBDRAFT_0167552 [Dictyostelium discoideum AX4] | 1.00E-47 | CL737Contig1_1_AA |
| 107 | *M. incognita* | CL407Contig1_1_AA | 4 | 1.58 | J | 6 | similar to L7a protein [Pan troglodytes] | 1.00E-73 | MI02619|MI02216|CL407Contig1_1_AA|MP00232 |
| 108 | *M. incognita* | CL145Contig1_1_AA | 4 | 2.47 | K | 2 | PurA ssDNA and RNA-binding protein [Brugia malayi] | 9.00E-44 | CL145Contig1_1_AA |
| 109 | *M. incognita* | CL765Contig1_1_AA | 4 | 1.76 | R | 3 | Hypothetical protein CBG01630 [C. briggsae] | 4.00E-45 | CL765Contig1_1_AA|MJ03296 |
| 110 | *M. incognita* | CL434Contig1_1_AA | 4 | 2.18 | Z | 1 | similar to kinesin heavy chain [Monodelphis domestica] | 6.00E-15 | CL434Contig1_1_AA|MC03208 |
| 111 | *M. incognita* | CL639Contig1_1_AA | 4 | 1.75 | O | 3 | heat shock protein 16-48a [C. elegans] | 2.00E-10 | CL639Contig1_1_AA |
| 112 | *M. incognita* | MI00853 | 4 | 0.44 | B | 2 | Hypothetical protein CBG21612 [C. briggsae] | 4.00E-12 | MI00853|MP00168|MP00020|MI07232|MA00200 |
| 113 | *M. incognita* | MI02091 | 4 | 4.38 | C | 4 | Hypothetical protein CBG09958 [C. briggsae] | 2.00E-78 | MI02091|MI05971 |
| 114 | *M. incognita* | MI01923 | 4 | 2.95 | I | 4 | Lipid Binding Protein family member (lbp-6) [C. elegans] | 2.00E-29 | MJ00018|MJ00020|MI01923|MI07723|MI08064|MI08396|MI07724|MI07749|MI07865|MI07907|MI07961|MI08067|MI08073|MI08389|MI08451|MI08466|MI08677|MI08735|MI08759|MI08761|MI08790|MP01462|MP02153|MP02205|MI09111|MP01487|MA00083 |
| 115 | *M. incognita* | MI06166 | 4 | 2.62 | O | 3 | Proteasome Alpha Subunit family member (pas-2) [C. elegans] | 5.00E-69 | MI06166|MI00580|MJ00052|MJ00172|MH07943|MC00970|MC01238|MI08570 |
| 116 | *M. incognita* | MI07537 | 4 | 0.9 | ? | 9 | caveolin-1 [Trichinella spiralis] | 2.00E-16 | MI07537 |
| 117 | *M. incognita* | 214a10c1.1_1_AA | 3 | 7.91 | Z | 1 | beta tubulin isotype 2 [Cylicocyclus nassatus] | 1.00E-68 | 214a10c1.1_1_AA|MA00442|2827988|4558495|203n05c1.1_1_AA|MA00761|1354512|39594164|cr01.Contig0.wum.271.1|CL2421Contig1_1_AA |
| 118 | *M. incognita* | CL109Contig1_1_AA | 3 | 4.01 | T | 5 | troponin C-like protein [Meloidogyne incognita] | 4.00E-74 | CL109Contig1_1_AA|MJ00730|MI02503|MJ00104|MJ00573|MA02856|57792488|MH02647|MA02314|MI03018|MI03778|PE00200|PE00156 |
| 119 | *M. incognita* | CL157Contig1_1_AA | 3 | 2.56 | ? | 9 | Hypothetical protein CBG15443 [C. briggsae] | 3.00E-38 | CL157Contig1_1_AA|CL157Contig2_1_AA|MI01963|MI03689|MH02688|MI05163 |
| 120 | *M. incognita* | CL2446Contig1_1_AA | 3 | 4.82 | T | 5 | Phosphatase 2A regulatory A subunit family member (paa-1) [C. elegans] | 2.00E-46 | CL2446Contig1_1_AA|MI01725|MA01498|MH03553|MJ03687|MH05521 |
| 121 | *M. incognita* | CL37Contig1_1_AA | 3 | 13.75 | J | 6 | Hypothetical protein CBG16683 [C. briggsae] | 2.00E-46 | MI02581|MH02420|CL37Contig1_1_AA|MJ00778|MI00938|MP02054|MC01065 |
| 122 | *M. incognita* | CL701Contig1_1_AA | 3 | 2.89 | J | 6 | hypothetical protein OsI_007391 [Oryza sativa (indica cultivar-group)] | 4.00E-59 | CL701Contig1_1_AA|HS01573|GR00786|HG00503|DI00385|MJ00733|MI02358|MP00464|100913234|14250.m00301|MC01100|AS02875|AS03055|AS01405|HS01585|GP00119|100913253|DI02049|AS13072|DI02528|DI02526|AS12075|DI02085|DI02536|AS07676|AS07727 |
| 123 | *M. incognita* | CL344Contig1_1_AA | 3 | 0.56 | J | 6 | translation elongation factor aEF-2, putative [Brugia malayi] | 0 | CL344Contig1_1_AA|HG01030|MC02878|MH04202 |
| 124 | *M. incognita* | CL441Contig1_1_AA | 3 | 5.24 | J | 6 | ribosomal protein L27 [Strongyloides papillosus] | 3.00E-36 | CL441Contig1_1_AA|MJ01057|MI02165|MH01697|MI01957|MJ01348|MC00898|224n14r1.1_1_AA|MC05340 |
| 125 | *M. incognita* | CL285Contig1_1_AA | 3 | 18.33 | ? | 9 | glycyl-tRNA synthetase beta subunit | 0.88 | CL285Contig1_1_AA|MI07866|MJ04960|MI01235 |
| 126 | *M. incognita* | CL1096Contig1_1_AA | 3 | 10.83 | J | 6 | Ribosomal protein, small subunit protein 10 [C. elegans] | 3.00E-36 | CL1096Contig1_1_AA |
| 127 | *M. incognita* | CL2396Contig1_1_AA | 3 | 1.65 | J | 6 | Ribosomal Protein, Small subunit family member (rps-7) [C. elegans] | 2.00E-56 | MJ00541|MP00136|MP00457|MI02627|MA00091|CL2396Contig1_1_AA|MH02500|MC00705|MI04206|MH07039 |
| 128 | *M. incognita* | CL164Contig1_1_AA | 3 | 3.44 | J | 6 | ribosomal protein LP1 [Argas monolakensis] | 3.00E-19 | MJ00781|MP00675|MI02431|MI02643|CL164Contig1_1_AA|MI05951|MH02516|MC01146|MC01252|MH02887|MJ01035|MC05546 |
| 129 | *M. incognita* | CL257Contig2_1_AA | 3 | 3.53 | J | 6 | threonyl-tRNA synthetase, cytoplasmic, putative [Brugia malayi] | 0 | CL257Contig2_1_AA|CL257Contig1_1_AA |
| 130 | *M. incognita* | 209c16r1.1_1_AA | 3 | 8.15 | O | 3 | T10B5.5a [C. elegans] | 5.00E-69 | 209c16r1.1_1_AA|MI01207|MC04688|GR02165 |
| 131 | *M. incognita* | CL561Contig1_1_AA | 3 | 0.94 | Z | 1 | Profilin protein 3 [C. elegans] | 6.00E-40 | MI01347|CL561Contig1_1_AA|MC02631|RS00413|GP00710|MH02577|HG00340 |
| 132 | *M. incognita* | CL195Contig1_1_AA | 3 | 2.62 | J | 6 | hypothetical protein FG09874.1 [Gibberella zeae PH-1] | 1.00E-47 | CL195Contig1_1_AA|MP00539|RS00516|MH01227|GR00687|PP00556|MC01057|GP01033|HS01226|MC01244|RS00272|MH00043|HS00954|MI08119|MP01289|MA02157|215b19c1.1_1_AA |
| 133 | *M. incognita* | 203f14r1.1_1_AA| | 3 | 1.31 | J | 6 | F33D11.10 [C. elegans] | 4.00E-137 | 39585896|cr01.Contig78.wum.6.1|31746585|71995724|cr01.Contig20.wum.108.1|17507121|2773184|14080.m00070|39595374|AE00366|MA00556|AC02178|AS02029|203f14r1.1_1_AA|14037.m00204|124217|532818|6700|71987143|cr01.Contig26.wum.12.1|39593936|MI02063|AS10493 |
| 134 | *M. incognita* | CL1426Contig1_1_AA | 3 | 3.53 | J | 6 | large subunit ribosomal protein 14 [Pristionchus sp. 15 RS5229] | 3.00E-34 | CL1426Contig1_1_AA|MI08686|MJ00999|MJ00118|MI01061|MH02032|MI01166|MP01546 |
| 135 | *M. incognita* | 218p13c1.1_1_AA | 3 | 4.07 | J | 6 | ribosomal protein L27a [Brugia malayi] | 2.00E-58 | 218p13c1.1_1_AA|MJ00011|MP00027|MA00226|MI01917|MI02186|MI02222|MJ00557|MP00015 |
| 136 | *M. incognita* | CL1135Contig1_1_AA | 3 | 2.41 | O | 3 | Hypothetical protein CBG09365 [C. briggsae] | 3.00E-58 | CL1135Contig1_1_AA|GR00306|MJ00164|MH00550|MA02146 |
| 137 | *M. incognita* | CL1413Contig1_1_AA | 3 | 0.89 | O | 3 | Proteasome regulatory particle, atpase-like protein 5 [C. elegans] | 3.00E-87 | CL1413Contig1_1_AA|MH02569|MA00837|MH02291|MA01551 |
| 138 | *M. incognita* | CL210Contig1_1_AA | 3 | 4.17 | O | 3 | ubiquitin carboxyl-terminal hydrolase L5, isoform CRA_f [Homo sapiens] | 7.00E-70 | CL210Contig1_1_AA|MI07467|MJ00042|MJ00036|MI08552 |
| 139 | *M. incognita* | CL187Contig1_1_AA | 3 | 0.78 | U | 6 | Hypothetical protein CBG24609 [C. briggsae] | 3.00E-47 | MI00558|MI00596|CL187Contig1_1_AA|CL187Contig2_1_AA|MI02403 |
| 140 | *M. incognita* | CL224Contig1_1_AA | 3 | 4.01 | U | 6 | C07A12.7b [C. elegans] | 7.00E-105 | CL224Contig1_1_AA |
| 141 | *M. incognita* | 210o05c1.1_1_AA | 3 | 2.29 | E | 4 | Hypothetical protein CBG05011 [C. briggsae] | 2.00E-78 | 210o05c1.1_1_AA|GR01466 |
| 142 | *M. incognita* | CL151Contig1_1_AA | 3 | 1.28 | J | 6 | small subunit ribosomal protein 1 [Pristionchus sp. 3 CZ3975] | 1.00E-112 | CL151Contig1_1_AA |
| 143 | *M. incognita* | CL1795Contig1_1_AA | 3 | 1.75 | O | 3 | Hypothetical protein CBG00674 [C. briggsae] | 3.00E-74 | CL1795Contig1_1_AA|MP01315 |
| 144 | *M. incognita* | CL2282Contig1_1_AA | 3 | 3.06 | O | 3 | Proteasome beta subunit protein 3 [C. elegans] | 9.00E-56 | CL2282Contig1_1_AA|MI00845|MP01532|MI00586|MI03355 |
| 145 | *M. incognita* | CL2864Contig1_1_AA | 3 | 4.58 | U | 6 | similar to epsilon subunit of coatomer protein complex isoform c isoform 4 [Canis familiaris] | 2.00E-31 | CL2864Contig1_1_AA|MA00204|MI00715|MI01165|MJ01983 |
| 146 | *M. incognita* | CL1Contig60_1_AA | 3 | 3.06 | ? | 9 | related to Islet cell Diabetes Autoantigen family member (ida-1) [C. elegans] | 5.00E-36 | CL1Contig60_1_AA|CL1Contig65_1_AA|MI04338 |
| 147 | *M. incognita* | CL34Contig1_1_AA | 3 | 1.25 | ? | 9 | No significant similarity found | No | CL34Contig1_1_AA|MI02571 |
| 148 | *M. incognita* | CL1Contig37_1_AA | 3 | 0.55 | R | 7 | Hypothetical protein CBG07317 [C. briggsae AF16] | 2.00E-63 | CL1Contig37_1_AA |
| 149 | *M. incognita* | CL1354Contig1_1_AA | 3 | 12.22 | ? | 9 | ABC multidrug transporter, putative [Aspergillus fumigatus Af293] | 3.9 | MI07890|MI06519|MI06337|MI06204|MJ01221|MJ01473|MJ00965|MI00276|MI05862|MI07325|CL1354Contig1_1_AA |
| 150 | *M. incognita* | CL1175Contig1_1_AA | 3 | 0.43 | C | 4 | TP synthase subunit family member (atp-2) [C. elegans] | 1.00E-102 | CL1175Contig1_1_AA |
| 151 | *M. incognita* | CL29Contig1_1_AA | 3 | 0.24 | ? | 9 | Hypothetical protein CBG19014 [C. briggsae AF16] | 9.00E-160 | CL29Contig1_1_AA |
| 152 | *M. incognita* | CL551Contig1_1_AA | 3 | 0.39 | C | 4 | GEX Interacting protein family member (gei-7) [C. elegans] | 2.00E-85 | CL551Contig1_1_AA |
| 153 | *M. incognita* | CL1347Contig1_1_AA | 3 | 2.29 | I | 4 | Hypothetical protein CBG01370 [C. briggsae AF16] | 6.00E-81 | CL1347Contig1_1_AA |
| 154 | *M. incognita* | CL288Contig1_1_AA | 3 | 2.75 | J | 6 | 60S ribosomal protein L24 (L30), putative [Brugia malayi] | 2.00E-49 | CL288Contig1_1_AA |
| 155 | *M. incognita* | CL633Contig1_1_AA | 3 | 13.74 | R | 9 | class V aminotransferase [Heterodera glycines] | 2.00E-154 | CL633Contig1_1_AA |
| 156 | *M. incognita* | CL268Contig1_1_AA | 3 | 3.44 | S | 9 | RO (Ro) ribonucleoProtein family member (rop-1) [C. elegans] | 4.00E-151 | CL268Contig1_1_AA |
| 157 | *M. incognita* | CL525Contig1_1_AA | 3 | 2.29 | ? | 8 | beta-1,4-endoglucanase [Meloidogyne incognita] | 6.00E-144 | CL525Contig1_1_AA |
| 158 | *M. incognita* | MI04764 | 3 | 7.55 | O | 3 | Proteasome Alpha Subunit family member (pas-5) [C. elegans] | 3.00E-66 | MJ00168|MP00008|MJ00787|MI04764|MA01388|MP00021|MH02725|MC01075|MH02844|MI06108 |
| 159 | *M. incognita* | MI01047 | 3 | 5.93 | J | 6 | 40S ribosomal protein S19S [Ascaris suum] | 2.00E-50 | MP00211|MH02561|MI01047|MI02550|MJ00672|MI00056|MC01029|MH09289|MI07778|GR00693|HG00598|MH09186|MH10303 |
| 160 | *M. incognita* | MI01542 | 3 | 5.35 | C | 4 | inorganic pyrophosphatase [Ascaris suum] | 2.00E-90 | MI01542|MC05189|30145752|44889057|71984883|30145751|71984889|cr01.Contig1370.wum.2.1|39585749|30145754|71984880|cr01.Contig1370.wum.2.2|cr01.Contig65.wum.9.2|25290351|30145753|71984895|HC00436|AE00298|GP03270|GR02148 |
| 161 | *M. incognita* | MI02399 | 3 | 4.58 | J | 6 | Hypothetical protein CBG01314 [C. briggsae] | 8.00E-74 | MA00956|MI02399|MH02221|MJ00591|HG00597|RS00459|GP01937|GR00147|MC04854 |
| 162 | *M. incognita* | MI00295 | 3 | 1.69 | ? | 2 | helicase domain protein [Paracoccus denitrificans PD1222] | 0.93 | MI00295|MI01791|MA02357|MH02360|MC04763 |
| 163 | *M. incognita* | MI02175 | 3 | 3.93 | J | 6 | R06C7.1 [C. elegans] | 3.00E-18 | MA00570|MJ00812|MI02175|MJ00181|MP01758|MJ03332|MP01637|MP00299 |
| 164 | *M. incognita* | MI02343 | 3 | 13.75 | O | 3 | proteasome Regulatory Particle, Non-ATPase-like family member (rpn-9) [C. elegans] | 4.00E-33 | MI02343|MP01655|MI03888|MH01957 |
| 165 | *M. incognita* | MI00116 | 3 | 2.04 | R | 9 | conserved hypothetical protein [Trichomonas vaginalis G3] | 9.00E-08 | MI00116|MI01040|MP01576 |
| 166 | *M. incognita* | MI08601 | 3 | 4.17 | G | 4 | PhosphoGlycerate Kinase family member (pgk-1) [C. elegans] | 3.00E-76 | MA01007|MI08601 |
| 167 | *M. incognita* | MI02110 | 3 | 2.08 | ? | 9 | putative amphid protein [Globodera rostochiensis] | 3.00E-22 | MI02110|MJ00102|MI08338 |
| 168 | *M. incognita* | MI02603 | 3 | 0.36 | ? | 9 | No significant similarity found | No | MI02603|MI02017|MI06756|MJ05058 |
| 169 | *M. incognita* | MI02588 | 3 | 4.58 | O | 3 | Hypothetical protein CBG08425 [C. briggsae] | 2.00E-58 | MA00577|MI02588|MI02701 |
| 170 | *M. incognita* | CL221Contig1_1_AA | 2 | 28.64 | E | 4 | putative acetyltransferase [Acidovorax avenae subsp. Citrulli AAC00-1] | 9.00E-56 | CL221Contig1_1_AA|CL221Contig2_1_AA|MI02646|MI00991 |
| 171 | *M. incognita* | 223g03c1.1_1_AA | 2 | 3.67 | A | 2 | SR Protein (splicing factor) family member (rsp-6) [C. elegans] | 1.00E-27 | 223g03c1.1_1_AA|PE00452 |
| 172 | *M. incognita* | CL254Contig1_1_AA | 2 | 1.88 | J | 6 | similar to insulinoma protein (rig) [Pan troglodytes] | 3.00E-50 | CL254Contig1_1_AA|MH02619|MI01071|MI02054|MI02609|MP00615|MJ01065|MI08679|MI08630|MI08974 |
| 173 | *M. incognita* | CL2910Contig1_1_AA | 2 | 5 | J | 6 | ribosomal protein L12 [C. briggsae] | 6.00E-70 | MP00563|MP00660|MC00303|MH01917|MI02420|CL2910Contig1_1_AA|AS02111|PE00140|PT00573|OS01580|HG00609|GR00705|AE00822|AE01105|17541134|3924781|39587700|48429086|48429087|7637748|cr01.Contig33.wum.90.1|HC00529|13282.m00276|PP00572|AS02862|8572161 |
| 174 | *M. incognita* | CL2467Contig1_1_AA | 2 | 10.83 | ? | 9 | Beige/BEACH domain containing protein [Trichomonas vaginalis G3] | 0.001 | CL2467Contig1_1_AA|209p17c1.1_1_AA |
| 175 | *M. incognita* | CL239Contig1_1_AA | 2 | 0.94 | C | 4 | Malate DeHydrogenase family member (mdh-1) [C. elegans] | 1.00E-121 | CL239Contig1_1_AA |
| 176 | *M. incognita* | 218l10c1.1_1_AA | 2 | 3.44 | J | 6 | mCG49427 [Mus musculus] | 9.00E-42 | MI05928|MJ02276|MH02228|MJ01260|MI00767|218l10c1.1_1_AA|AS02926|TX00496|TX00238|AS02712|AS03196|HG00509|AS04972|AS03349|AS05079|AS04652|AS05725 |
| 177 | *M. incognita* | CL2Contig17_1_AA | 2 | 0.39 | ? | 9 | TropoNin T family member (tnt-2) [C. elegans] | 0.047 | CL2Contig17_1_AA|MJ03223|MI03770|MI08225|MI06592 |
| 178 | *M. incognita* | CL350Contig1_1_AA | 2 | 5.24 | ? | 8 | cellulose binding protein precursor [Meloidogyne incognita] | 4.00E-113 | CL350Contig1_1_AA|MA00303|MA00559|126153877|126153879|126153883|126153881|MA00036|126153869|126153875 |
| 179 | *M. incognita* | CL1325Contig1_1_AA | 2 | 3.44 | J | 6 | Hypothetical protein CBG06842 [C. briggsae] | 3.00E-34 | CL1325Contig1_1_AA|MI08481 |
| 180 | *M. incognita* | 209b10r1.1_1_AA | 2 | 2.62 | R | 3 | Chain A, Structure Of C. Elegans Leucine Aminopeptidase | 2.00E-57 | 209b10r1.1_1_AA|MA01003 |
| 181 | *M. incognita* | CL181Contig1_1_AA | 2 | 1.26 | S | 9 | venom allergen-like protein [Meloidogyne incognita] | 4.00E-25 | CL181Contig1_1_AA|MA00499|MA00565|MA00246|MA00576|MI02255|MA00333|CL181Contig2_1_AA|MI00544|MA02803|MI04097 |
| 182 | *M. incognita* | CL811Contig1_1_AA | 2 | 1.31 | Z | 1 | F42H10.3 [C. elegans] | 5.00E-60 | MJ03499|CL811Contig1_1_AA|MI03682|MA00620 |
| 183 | *M. incognita* | CL2116Contig1_1_AA | 2 | 2.62 | ? | 9 | ZK829.7 [C. elegans] | 3.00E-50 | CL2116Contig1_1_AA|MA01326 |
| 184 | *M. incognita* | CL405Contig1_1_AA | 2 | 1.65 | ? | 9 | No significant similarity found | No | CL405Contig1_1_AA |
| 185 | *M. incognita* | CL781Contig1_1_AA | 2 | 7.33 | ? | 3 | Cysteine Protease Inhibitor family member (cpi-2) [C. elegans] | 6.00E-12 | CL781Contig1_1_AA|PT00412|MI01889|MJ03141|MI01073 |
| 186 | *M. incognita* | CL1Contig18_1_AA | 2 | 0.69 | C | 4 | ALdehyde deHydrogenase family member (alh-5) [C. elegans] | 5.00E-62 | CL1Contig18_1_AA|MA01001|MJ00868|MJ00616|MI01781 |
| 187 | *M. incognita* | 206n08r1.1_1_AA | 2 | 45836 | H | 4 | S-adenosylhomocysteine hydrolase; AHH [C. elegans] | 9.00E-93 | cr01.Contig6.wum.264.1|PT00487|HC02865|AE00271|MI03684|AC03154|134182|156326|17506425|2804454|298816|39595800|14990.m07753|TS01190|PP00231|SR00649|XI03299|SS01194|MA01725|AE04175|MI01009|AC04650|AS00791|206n08r1.1_1_AA|MH02989|AS07962 |
| 188 | *M. incognita* | CL2678Contig1_1_AA | 2 | 0.83 | O | 3 | peptidylprolyl isomerase B [Danio rerio] | 3.00E-69 | CL2678Contig1_1_AA|MI02520|MI00132 |
| 189 | *M. incognita* | 208l10r1.1_1_AA | 2 | 3.93 | O | 3 | proteasome Regulatory Particle, Non-ATPase-like family member (rpn-11) [C. elegans] | 2.00E-83 | 208l10r1.1_1_AA|MA00847 |
| 190 | *M. incognita* | 217o11r1.1_1_AA | 2 | 1.62 | O | 3 | CCT-2 [C. elegans] | 8.00E-62 | 217o11r1.1_1_AA|MC00890 |
| 191 | *M. incognita* | 218i05r1.1_1_AA | 2 | 0.8 | J | 6 | similar to cytoplasmic polyadenylation element binding protein 1 isoform 6 [Macaca mulatta] | 2.00E-43 | MJ01736|MJ01733|MI00818|MP01584|MI02296|MI02536|CL1125Contig1_1_AA|218i05r1.1_1_AA|MC00775|MJ00475|MH02720|MP02245|MI01896 |
| 192 | *M. incognita* | 203b23r1.1_1_AA | 2 | 1.83 | O | 3;7 | F17C11.9b [C. elegans] | 7.00E-35 | MA00137|203b23r1.1_1_AA|MH01793 |
| 193 | *M. incognita* | 222f20r1.1_1_AA | 2 | 3.93 | O | 3 | Hypothetical protein CBG18052 [C. briggsae] | 1.00E-69 | 222f20r1.1_1_AA|MC00110 |
| 194 | *M. incognita* | CL1842Contig1_1_AA | 2 | 1.25 | ? | 9 | putative Type IV secretory pathway VirB6 components [Ehrlichia canis str. Jake] | 0.27 | CL1842Contig1_1_AA|MI07859 |
| 195 | *M. incognita* | CL2Contig4_1_AA | 2 | 22.91 | ? | 9 | No significant similarity found | No | CL2Contig4_1_AA |
| 196 | *M. incognita* | 210j11r1.1_1_AA | 2 | 1.05 | C | 4 | Hypothetical protein CBG06335 [C. briggsae] | 9.00E-119 | 210j11r1.1_1_AA |
| 197 | *M. incognita* | 210o05r1.1_1_AA | 2 | 2.5 | E | 4 | C14F11.1a [C. elegans] | 7.00E-76 | 210o05r1.1_1_AA|MI00680 |
| 198 | *M. incognita* | CL2805Contig1_1_AA | 2 | 4.07 | EO | 4;3 | aminopeptidase [Heterodera glycines] | 2.00E-80 | CL2805Contig1_1_AA |
| 199 | *M. incognita* | CL1Contig57_1_AA | 2 | 0.63 | O | 3;7 | secreted glutathione peroxidase [Globodera rostochiensis] | 1.00E-77 | CL1Contig57_1_AA|MI02671|MI04155|MI02670|MA00328 |
| 200 | *M. incognita* | CL2715Contig1_1_AA | 2 | 1.83 | O | 3 | Hypothetical protein CBG24861 [C. briggsae] | 4.00E-23 | CL2715Contig1_1_AA |
| 201 | *M. incognita* | 210a01r1.1_1_AA | 2 | 0.56 | O | 3 | Hypothetical protein CBG00721 [C. briggsae] | 7.00E-103 | MH02329|MP00358|MI02019|210a01r1.1_1_AA |
| 202 | *M. incognita* | 210k21r1.1_1_AA | 2 | 4.07 | O | 3 | Ubiquitin carboxyl-terminal hydrolase family protein [Brugia malayi] | 8.00E-24 | 210k21r1.1_1_AA |
| 203 | *M. incognita* | CL15Contig1_1_AA | 2 | 0.67 | AJ | 2;6 | polyadenylate-binding protein 1, putative [Brugia malayi] | 0 | CL15Contig1_1_AA |
| 204 | *M. incognita* | CL712Contig1_1_AA | 2 | 6.87 | AR | 2 | KH domain containing protein [Brugia malayi] | 2.00E-62 | CL712Contig1_1_AA |
| 205 | *M. incognita* | CL687Contig1_1_AA | 2 | 0.52 | I | 4 | Hypothetical protein F53A2.7 [C. elegans] | 2.00E-57 | CL687Contig1_1_AA |
| 206 | *M. incognita* | CL886Contig1_1_AA | 2 | 1.72 | J | 6 | large subunit ribosomal protein 1 [Pristionchus americanus] | 3.00E-94 | CL886Contig1_1_AA |
| 207 | *M. incognita* | 208l23r1.1_1_AA | 2 | 3.44 | J | 6 | eukaryotic translation initiation factor 3 subunit 6, putative [Brugia malayi] | 5.00E-80 | 208l23r1.1_1_AA |
| 208 | *M. incognita* | CL1707Contig1_1_AA | 2 | 1.83 | O | 3 | Hypothetical protein CBG21707 [C. briggsae] | 2.00E-66 | CL1707Contig1_1_AA|MI01709|MI08718 |
| 209 | *M. incognita* | CL1833Contig1_1_AA | 2 | 0.92 | O | 3 | hypothetical protein [Ornithorhynchus anatinus] | 8.00E-24 | CL1833Contig1_1_AA|MI02845 |
| 210 | *M. incognita* | 217d04c1.1_1_AA | 2 | 4.58 | O | 3 | ThiF family protein, putative [Brugia malayi] | 3.00E-23 | 217d04c1.1_1_AA |
| 211 | *M. incognita* | CL2540Contig1_1_AA | 2 | 1.41 | Q | 4 | Hypothetical protein CBG06017 [C. briggsae] | 7.00E-40 | CL2540Contig1_1_AA|MI03030 |
| 212 | *M. incognita* | CL1Contig20_1_AA | 2 | 6.87 | ? | 9 | No significant similarity found | No | CL1Contig20_1_AA |
| 213 | *M. incognita* | CL2269Contig1_1_AA | 2 | 2.29 | ? | 9 | conserved hypothetical protein [Brugia malayi] | 3.00E-24 | CL2269Contig1_1_AA |
| 214 | *M. incognita* | CL252Contig1_1_AA | 2 | 0.38 | ? | 9 | unnamed protein product [Kluyveromyces lactis] | 2.9 | MI04412|CL252Contig1_1_AA |
| 215 | *M. incognita* | CL32Contig1_1_AA | 2 | 2.29 | ? | 9 | Hypothetical protein M02E1.2 [C. elegans] | 1.6304 | CL32Contig1_1_AA|MI01712 |
| 216 | *M. incognita* | CL929Contig1_1_AA | 2 | 0.54 | ? | 9 | putative short-chain dehydrogenase/reductase [Rhizobium leguminosarum] | 0.75 | CL929Contig1_1_AA |
| 217 | *M. incognita* | CL431Contig1_1_AA | 2 | 0.5 | C | 4 | hypothetical protein CBG09810 [C. briggsae AF16] | 0 | CL431Contig1_1_AA |
| 218 | *M. incognita* | CL383Contig1_1_AA | 2 | 3.67 | E | 4 | spermidine synthase [C. elegans] | 2.00E-116 | CL383Contig1_1_AA |
| 219 | *M. incognita* | CL455Contig1_1_AA | 2 | 0.43 | I | 4 | Hypothetical protein CBG14783 [C. briggsae AF16] | 8.00E-164 | CL455Contig1_1_AA |
| 220 | *M. incognita* | CL555Contig1_1_AA | 2 | 0.87 | J | 6 | 60S ribosomal protein L23a, putative [Brugia malayi] | 8.00E-57 | CL555Contig1_1_AA |
| 221 | *M. incognita* | CL228Contig1_1_AA | 2 | 1.31 | M | 9 | C47B2.6b [C. elegans] | 4.00E-127 | CL228Contig1_1_AA |
| 222 | *M. incognita* | CL2808Contig1_1_AA | 2 | 9.16 | O | 3 | PCI domain containing protein [Brugia malayi] | 5.00E-54 | CL2808Contig1_1_AA |
| 223 | *M. incognita* | CL2824Contig1_1_AA | 2 | 1.67 | O | 3 | proteasome Regulatory Particle, ATPase-like family member (rpt-6) [C. elegans] | 2.00E-106 | CL2824Contig1_1_AA |
| 224 | *M. incognita* | CL846Contig1_1_AA | 2 | 0.87 | O | 3 | Hypothetical 19.4 kDa protein ZC395.10 in chromosome III, putative [Brugia malayi] | 2.00E-19 | CL846Contig1_1_AA |
| 225 | *M. incognita* | 206m13r1.1_1_AA | 2 | 0.61 | O | 3 | T-complex protein 1, gamma subunit, putative [Brugia malayi] | 8.00E-104 | 206m13r1.1_1_AA |
| 226 | *M. incognita* | CL2458Contig1_1_AA | 2 | 0.29 | P | 4 | Na,K-ATPase alpha subunit, putative [Brugia malayi] | 0.8636 | CL2458Contig1_1_AA |
| 227 | *M. incognita* | CL1518Contig1_1_AA | 2 | 1.67 | S | 9 | hypothetical protein [Nasonia vitripennis] | 1.00E-25 | CL1518Contig1_1_AA |
| 228 | *M. incognita* | CL144Contig1_1_AA | 2 | 2.62 | T | 5 | rasputin, putative [Brugia malayi] | 4.00E-20 | CL144Contig1_1_AA |
| 229 | *M. incognita* | CL1803Contig1_1_AA | 2 | 0.87 | Z | 1 | Hypothetical protein CBG23416 [C. briggsae AF16] | 3.00E-37 | CL1803Contig1_1_AA |
| 230 | *M. incognita* | 201b04r1.1_1_AA | 2 | 0.61 | Z | 1 | Hypothetical protein CBG14139 [C. briggsae AF16] | 1.00E-106 | 201b04r1.1_1_AA |
| 231 | *M. incognita* | 224n10r1.1_1_AA | 2 | 4.58 | ? | 9 | hypothetical protein RBE_0619 [Rickettsia bellii RML369-C] | 0.73 | 224n10r1.1_1_AA |
| 232 | *M. incognita* | MI06758 | 2 | 6.55 | ? | 9 | Spike glycoprotein [Bat coronavirus HKU4-3] | 1.8 | MI06758|MC00805 |
| 233 | *M. incognita* | MI00269 | 2 | 2.23 | O | 3 | chaperonine protein HSP60 [Onchocerca volvulus] | 4.00E-109 | GP00165|4680247|15413.m00008|MI00269 |
| 234 | *M. incognita* | MI02712 | 2 | 14.66 | ? | 9 | No significant similarity found | No | MP01958|MJ01184|MJ00881|MI02712|MJ00674 |
| 235 | *M. incognita* | MI01032 | 2 | 6.11 | O | 3 | similar to S-phase kinase-associated protein 1A isoform b [Canis familiaris] | 3.00E-20 | MI01032|MA00455|MP00379|MP00221|MJ03874|MI03284 |
| 236 | *M. incognita* | MI03955 | 2 | 0.19 | ? | 9 | As37 [Ascaris suum] | 4.00E-139 | MJ00732|22036079|14972.m07771|AS03014|MA00884|MA00741|MJ01234|MI03955|MH07343|MA00152|PP00729|MP01882 |
| 237 | *M. incognita* | MI00505 | 2 | 0.63 | C | 4 | arginine kinase [Heterodera glycines] | 2.00E-65 | MI00505|MJ04090|MJ04969|MH00371|MI00497|MH03404 |
| 238 | *M. incognita* | MI00222 | 2 | 2.08 | O | 3 | Hypothetical protein CBG12349 [C. briggsae] | 1.00E-80 | MI00222|GR00704|MC05227|MH10200|MA00145 |
| 239 | *M. incognita* | MI05568 | 2 | 1.39 | V | 9 | Hypothetical protein CBG09861 [C. briggsae] | 2.00E-48 | MJ00832|MH00317|MI05568|MJ00307|MJ00041|MI01055|MJ00935|MI00573 |
| 240 | *M. incognita* | MI00994 | 2 | 1.18 | Z | 1 | Profilin-1 [C. elegans] | 4.00E-38 | MI00994|MI02236|MC01061 |
| 241 | *M. incognita* | MI01375 | 2 | 36.63 | B | 2 | Hypothetical protein CBG03777 [C. briggsae] | 1.00E-43 | HG00010|14500.m00157|HG00576|12743.m00017|MP00329|MI01375|MA03266|HG00145|MJ00579|DI02331|MJ00400|RS00588|MH01450|MH01881|SS00585|GR00162|MC01010|GR00056|GR00125|DI01375|12698.m00329|MC00067|SR04594|MC00785|MC05047 |
| 242 | *M. incognita* | MI03461 | 2 | 1.26 | O | 3;7 | putative glutathione peroxidase [Globodera rostochiensis] | 9.00E-48 | MA02847|MH02288|MJ04414|MI03461 |
| 243 | *M. incognita* | MI08683 | 2 | 18.33 | R | 9 | similar to LOC594885 protein [Gallus gallus] | 2.00E-52 | MJ03382|MA02024|MI08683 |
| 244 | *M. incognita* | MI08556 | 2 | 0.44 | ? | 9 | K03E5.2b [C. elegans] | 2.00E-39 | MI08556|MI01836|MH02574|MJ05009|MC03779 |
| 245 | *M. incognita* | MI04304 | 2 | 1.15 | G | 4 | Hypothetical protein CBG04165 [C. briggsae] | 5.00E-76 | MA00877|MI04304|MP01586 |
| 246 | *M. incognita* | MI00037 | 2 | 1.83 | J | 6 | large subunit ribosomal protein 30 [Koerneria sp. RS1982] | 1.00E-42 | PT04847|PT00519|MA02741|MH02591|MI00037|MI01651|MI02160|MJ00555|MJ01308|MP00229|MC01059|HG00072|HG00754|MI04059|MI04853|PE00456 |
| 247 | *M. incognita* | MI01604 | 2 | 1.53 | J | 6 | 60S ribosomal protein L18a, putative [Brugia malayi] | 4.00E-80 | MI01604|MJ00553|MC00945|MJ02253|MP02263|MC05140 |
| 248 | *M. incognita* | MI02122 | 2 | 2.75 | J | 6 | Hypothetical protein CBG02309 [C. briggsae] | 3.00E-29 | MH00977|MI02122|MP00042|MI00100 |
| 249 | *M. incognita* | MI05517 | 2 | 0.95 | L | 2 | protein kinase N3 [Rattus norvegicus] | 1.00E-46 | MA00426|MI05517 |
| 250 | *M. incognita* | MI01739 | 2 | 1.83 | O | 3 | Hypothetical protein CBG02839 [C. briggsae] | 9.00E-21 | MI01739|MA01043|MI00704|MJ00224|MJ00317|MP01177 |
| 251 | *M. incognita* | MI02172 | 2 | 1.62 | O | 3 | chaperonin 10 [Strongyloides ratti] | 3.00E-33 | MJ00163|MI02172|MI02286|MP00351|MC00790|MJ01845 |
| 252 | *M. incognita* | MI02651 | 2 | 13.74 | O | 3 | Hypothetical protein CBG16680 [C. briggsae] | 1.00E-28 | MA01997|MI02651|MJ00866|PT04899|MP00626|MI01767|MJ00642|MJ00843|PT04465|MH00627|MC00973|MI04039 |
| 253 | *M. incognita* | MI03652 | 2 | 5.5 | O | 3 | furin [Dirofilaria immitis] | 5.00E-36 | MI03652 |
| 254 | *M. incognita* | MI00527 | 2 | 3.44 | R | 3 | Hypothetical protein CBG22302 [C. briggsae] | 4.00E-63 | MI00527|MA01808 |
| 255 | *M. incognita* | MI09006 | 2 | 13.74 | U | 6 | similar to coatomer protein gamma 2-subunit, partial [Strongylocentrotus purpuratus] | 0.001 | MI09006 |
| 256 | *M. incognita* | MI01475 | 2 | 3.06 | ? | 9 | hypothetical protein 461.t00009 [Entamoeba histolytica HM-1:IMSS] | 1.5 | MI01475 |
| 257 | *M. incognita* | MI03503 | 2 | 5.5 | ? | 9 | F22A3.6a [C. elegans] | 5.00E-25 | MA00041|MA00506|MJ03344|MI03503|MA00554|MI02494|MH02430 |
| 258 | *M. incognita* | MI01821 | 2 | 9.16 | A | 2 | nucleolar RNA-binding Nop10p-like protein [Oryza sativa] | 2.00E-14 | MH01721|MI01821|MJ01421|MI08376 |
| 259 | *M. incognita* | MI06351 | 2 | 18335 | A | 2 | similar to CG2163-PA, isoform A [Tribolium castaneum] | 2.00E-45 | MI06351 |
| 260 | *M. incognita* | MI03941 | 2 | 2.62 | C | 4 | Probable vacuolar ATP synthase subunit G [C. briggsae] | 2.00E-25 | MJ01885|MA00622|MA02584|MI03941|MI08905|MJ01496|MP01168|MH02668 |
| 261 | *M. incognita* | MI06202 | 2 | 1.41 | E | 4 | similar to CNDP dipeptidase 2 [Strongylocentrotus purpuratus] | 1.00E-77 | MI06202|MI06995|MI00956|MH05737|MC03885|MC00159|MC05122|MI05920|MI06553 |
| 262 | *M. incognita* | MI01658 | 2 | 0.5 | K | 2 | elongation factor 1-beta [Artemia salina] | 2.00E-35 | MI01658 |
| 263 | *M. incognita* | MI01668 | 2 | 2.62 | N | 9 | Hypothetical protein CBG22765 [C. briggsae] | 6.00E-06 | MI01668|MI07929 |
| 264 | *M. incognita* | MI00440 | 2 | 1.67 | O | 3 | unnamed protein product [Tetraodon nigroviridis] | 2.8 | MI00440|MJ05025|MI00540 |
| 265 | *M. incognita* | MI00905 | 2 | 18.32 | O | 3 | Proteasome (prosome macropain) subunit beta type 4 [Xenopus tropicalis] | 4.00E-39 | MH01866|MI00905|MP00992|MA00437|MI00672 |
| 266 | *M. incognita* | MI01518 | 2 | 4.58 | S | 9 | Hypothetical protein CBG11576 [C. briggsae] | 3.00E-31 | MI01518 |
| 267 | *M. incognita* | MI05343 | 2 | 4.58 | U | 6 | similar to CG10686-PA [Tribolium castaneum] | 1.00E-07 | MI05343 |
| 268 | *M. incognita* | 210d13r1.1_1_AA | 1 | 4.01 | J | 6 | Ribosomal Protein, Small subunit family member (rps-26) [C. elegans] | 1.00E-39 | 210d13r1.1_1_AA|MJ00967|MI04025|MC01091|MP01453|MI02408|MI00136 |
| 269 | *M. incognita* | 210k07r1.1_1_AA | 1 | 45836 | ? | 9 | No significant similarity found | No | 210k07r1.1_1_AA|MI02416 |
| 270 | *M. incognita* | CL1689Contig1_1_AA | 1 | 6.87 | J | 6 | Ribosomal Protein, Large subunit family member (rpl-23) [C. elegans] | 7.00E-66 | HC01800|CL1689Contig1_1_AA|MH02247|MI02519|MJ00807|MP01322|MI02206|OS01021|AC02996|HC04460|AC02982|NB01127|XI04298|AC00955|AE01441|AE00256|AE00415|AE01112|HC04338|XI02122|HC02560|MI09077|HC04845|HC03748 |
| 271 | *M. incognita* | CL895Contig1_1_AA | 1 | 4.28 | TU | 5;6 | K10C3.2 [C. elegans] | 9.00E-20 | CL895Contig1_1_AA|MP00551 |
| 272 | *M. incognita* | 204h01r1.1_1_AA | 1 | 4.58 | E | 4 | Hypothetical protein CBG13820 [C. briggsae AF16] | 3.00E-61 | MA01305|204h01r1.1_1_AA |
| 273 | *M. incognita* | CL2258Contig1_1_AA | 1 | 1.75 | U | 6 | Probable coatomer delta subunit, putative [Brugia malayi] | 2.00E-67 | CL2258Contig1_1_AA|MA02041 |
| 274 | *M. incognita* | CL381Contig1_1_AA | 1 | 2.08 | ? | 9 | No significant similarity found | No | CL381Contig1_1_AA |
| 275 | *M. incognita* | CL378Contig1_1_AA | 1 | 0.23 | C | 4 | Vacuolar H ATPase family member (vha-12) [C. elegans] | 4.00E-98 | CL378Contig1_1_AA|cr01.Contig0.wum.839.1|39583624|cr01.Contig147.wum.16.1|17510931|7331730|13801.m00221|39597843|74907055|MH02509|MJ04552|GR01851|AS07318 |
| 276 | *M. incognita* | 203p11r1.1_1_AA | 1 | 1.08 | E | 4 | similar to serine hydroxymethyltransferase 1 (soluble) [Gallus gallus] | 7.00E-65 | 203p11r1.1_1_AA|MP00416 |
| 277 | *M. incognita* | CL1630Contig1_1_AA | 1 | 1.31 | K | 2 | ebna2 binding protein P100 [Aedes aegypti] | 1.00E-30 | CL1630Contig1_1_AA|MI02938|MI01313 |
| 278 | *M. incognita* | CL129Contig1_1_AA | 1 | 2.16 | P | 4;7 | mitochondrial manganese superoxide dismutase [Macrobrachium rosenbergii] | 2.00E-85 | CL129Contig1_1_AA |
| 279 | *M. incognita* | CL520Contig2_1_AA | 1 | 12.22 | R | 9 | hypothetical protein Bm1_17170 [Brugia malayi] | 7.00E-14 | CL520Contig2_1_AA|CL520Contig1_1_AA |
| 280 | *M. incognita* | 206l24r1.1_1_AA | 1 | 4.58 | U | 6 | MGC80100 protein, putative [Brugia malayi] | 4.00E-88 | 206l24r1.1_1_AA |
| 281 | *M. incognita* | CL500Contig1_1_AA | 1 | 4.58 | ZT | 1;5 | CAP protein [Brugia malayi] | 7.00E-66 | CL500Contig1_1_AA|CL2891Contig1_1_AA |
| 282 | *M. incognita* | CL139Contig1_1_AA | 1 | 4.58 | ? | 9 | hypothetical protein UM00777.1 [Ustilago maydis 521] | 0.61 | CL139Contig1_1_AA|MI01708|MJ00465|MA02945|MI02164 |
| 283 | *M. incognita* | CL46Contig1_1_AA | 1 | 0.83 | ? | 9 | Hypothetical protein CBG06665 [C. briggsae] | 8.00E-27 | CL46Contig1_1_AA|MJ03353 |
| 284 | *M. incognita* | CL1575Contig1_1_AA | 1 | 1.53 | G | 4 | fructose-1,6-bisphosphatase [C. elegans] | 1.00E-51 | CL1575Contig1_1_AA |
| 285 | *M. incognita* | CL2965Contig1_1_AA | 1 | 2.5 | K | 2 | small nuclear ribonucleoprotein N [Macropus eugenii] | 6.00E-30 | CL2965Contig1_1_AA |
| 286 | *M. incognita* | CL1793Contig1_1_AA | 1 | 9.16 | Q | 4;7 | Hypothetical protein CBG13395 [C. briggsae] | 2.00E-12 | CL1793Contig1_1_AA |
| 287 | *M. incognita* | CL26Contig3_1_AA | 1 | 5.5 | T | 5 | Protein kinase domain containing protein [Brugia malayi] | 3.00E-103 | CL26Contig3_1_AA |
| 288 | *M. incognita* | 211k05r1.1_1_AA | 1 | 18335 | U | 6 | protein F32E10.4 [C. elegans] | 1.00E-64 | 211k05r1.1_1_AA|MI03155 |
| 289 | *M. incognita* | CL280Contig1_1_AA | 1 | 0.76 | Z | 1 | Hypothetical protein CBG14139 [C. briggsae] | 0 | CL280Contig1_1_AA|1206048|17569483|39597692|14920.m00400|cr01.Contig0.wum.997.1|MH05990 |
| 290 | *M. incognita* | CL2Contig10_1_AA| | 1 | 0.04 | Z | 1 | myosin regulatory light chain [Meloidogyne incognita] | 4.00E-81 | MA00485|MJ00605|MJ00748|MJ00792|MI02591|MP00106|17530145|MA00544|MA00563|MI02479|MI02526|MP00408|MP00662|CL2Contig10_1_AA|MJ02440|CL2Contig5_2_AA|MI03312 |
| 291 | *M. incognita* | 221l04r1.1_1_AA | 1 | 0.56 | ? | 8 | polygalacturonase [Meloidogyne incognita] | 0 | 21628922|221l04r1.1_1_AA |
| 292 | *M. incognita* | 223a18c1.1_1_AA | 1 | 2.29 | ? | 9 | Hypothetical protein CBG22240 [C. briggsae AF16] | 1.00E-09 | 223a18c1.1_1_AA |
| 293 | *M. incognita* | CL2931Contig1_1_AA | 1 | 1.83 | A | 2 | similar to Small nuclear ribonucleoprotein polypeptide A, partial [Ornithorhynchus anatinus] | 9.00E-33 | CL2931Contig1_1_AA|MI00842 |
| 294 | *M. incognita* | CL6Contig6_1_AA | 1 | 0.83 | A | 2 | splicing factor U2AF65 [C. briggsae] | 1.00E-69 | CL6Contig6_1_AA|MH05634|MH01159 |
| 295 | *M. incognita* | 208a01r1.1_1_AA | 1 | 6.11 | A | 2 | conserved hypothetical protein [Brugia malayi] | 5.00E-09 | 208a01r1.1_1_AA |
| 296 | *M. incognita* | 223a18r1.1_1_AA | 1 | 0.65 | A | 2 | Hypothetical protein CBG22240 [C. briggsae] | 3.00E-41 | 223a18r1.1_1_AA|MJ02934 |
| 297 | *M. incognita* | 215o03c1.1_1_AA | 1 | 4.58 | E | 4 | Hypothetical protein CBG02861 [C. briggsae] | 2.00E-88 | MA02040|215o03c1.1_1_AA|MJ00040|MJ00240 |
| 298 | *M. incognita* | CL2121Contig1_1_AA | 1 | 1.83 | G | 4 | Hypothetical protein CBG03385 [C. briggsae AF16] | 7.00E-65 | MA01672|MA00675|CL2121Contig1_1_AA|MI00461 |
| 299 | *M. incognita* | CL2507Contig1_1_AA | 1 | 1.67 | G | 4 | 2-oxoglutarate dehydrogenase E1 component [C. briggsae] | 2.00E-68 | CL2507Contig1_1_AA |
| 300 | *M. incognita* | CL2771Contig1_1_AA | 1 | 1.83 | G | 4 | Hypothetical protein CBG18265 [C. briggsae] | 3.00E-85 | MH05460|CL2771Contig1_1_AA|MI03426|MA02650|MH09016 |
| 301 | *M. incognita* | 203i19c1.1_1_AA | 1 | 1.08 | G | 4 | Melibiase family protein [Brugia malayi] | 4.00E-21 | 203i19c1.1_1_AA |
| 302 | *M. incognita* | 204p08r1.1_1_AA | 1 | 1.83 | G | 4 | Y110A7A.6b [C. elegans] | 5.00E-97 | 204p08r1.1_1_AA |
| 303 | *M. incognita* | CL651Contig1_1_AA | 1 | 0.57 | I | 4 | 3-ketoacyl-coa thiolase protein 1 [C. elegans] | 4.00E-155 | CL651Contig1_1_AA |
| 304 | *M. incognita* | 206p09r1.1_1_AA | 1 | 2.62 | J | 6 | lysyl (K) tRNA Synthetase [C. elegans] | 5.00E-114 | 206p09r1.1_1_AA |
| 305 | *M. incognita* | CL1031Contig1_1_AA | 1 | 1.83 | O | 3 | Proteasome A-type and B-type family protein [Brugia malayi] | 3.00E-76 | CL1031Contig1_1_AA |
| 306 | *M. incognita* | CL1Contig15_1_AA | 1 | 0.83 | O | 3 | proprotein convertase 2 [Heterodera glycines] | 0 | CL1Contig15_1_AA|14537990|MP00710|MH08418 |
| 307 | *M. incognita* | CL2418Contig1_1_AA | 1 | 0.23 | O | 3 | Heat Shock Protein family member (hsp-6) [C. elegans] | 3.00E-78 | CL2418Contig1_1_AA|MI05469 |
| 308 | *M. incognita* | CL902Contig1_1_AA | 1 | 9.16 | O | 3 | prolyl oligopeptidase family protein [Brugia malayi] | 4.00E-82 | CL902Contig1_1_AA |
| 309 | *M. incognita* | CL135Contig1_1_AA | 1 | 2.29 | R | 9 | similar to juvenile hormone epoxide hydrolase [Nasonia vitripennis] | 1.00E-53 | CL135Contig1_1_AA |
| 310 | *M. incognita* | CL155Contig2_1_AA | 1 | 1.53 | S | 9 | vap-1 [Heterodera glycines] | 7.00E-29 | MA00562|CL155Contig2_1_AA|CL155Contig1_1_AA |
| 311 | *M. incognita* | CL2550Contig1_1_AA | 1 | 2.62 | T | 5 | Protein kinase domain containing protein [Brugia malayi] | 3.00E-58 | CL2550Contig1_1_AA |
| 312 | *M. incognita* | CL3006Contig1_1_AA | 1 | 0.73 | T | 5 | UNCoordinated family member (unc-43) [C. elegans] | 4.00E-159 | CL3006Contig1_1_AA |
| 313 | *M. incognita* | 218g22r1.1_1_AA | 1 | 6.11 | T | 5 | signal transducting adaptor protein [Drosophila melanogaster] | 2.00E-07 | 218g22r1.1_1_AA |
| 314 | *M. incognita* | CL1220Contig1_1_AA | 1 | 3.67 | U | 6 | T08G11.1b [C. elegans] | 6.00E-54 | CL1220Contig1_1_AA|210p13c1.1_1_AA |
| 315 | *M. incognita* | 211k16c1.1_1_AA | 1 | 6.11 | U | 6 | coatomer gamma subunit, putative [Brugia malayi] | 1.00E-80 | 211k16c1.1_1_AA |
| 316 | *M. incognita* | 225m13c1.1_1_AA | 1 | 3.05 | U | 6 | F38E11.5 [C. elegans] | 1.00E-38 | MA01019|225m13c1.1_1_AA |
| 317 | *M. incognita* | CL1865Contig1_1_AA | 1 | 4.58 | UR | 6 | Dynamin central region family protein [Brugia malayi] | 5.00E-72 | CL1865Contig1_1_AA |
| 318 | *M. incognita* | CL1101Contig1_1_AA | 1 | 3.67 | Z | 1 | hypothetical protein CBG09860 [C. briggsae AF16] | 1.00E-44 | CL1101Contig1_1_AA |
| 319 | *M. incognita* | CL384Contig1_1_AA | 1 | 1.15 | Z | 1 | pinin/SDK/memA/ protein conserved region containing protein [Brugia malayi] | 2.00E-43 | CL384Contig1_1_AA |
| 320 | *M. incognita* | CL2552Contig1_1_AA | 1 | 2.29 | ? | 9 | Hypothetical protein CBG04281 [C. briggsae] | 2.00E-38 | 25395550|MH01487|MC02910|AS01790|12984.m00011|GR00748|GP01868|HC04140|39583097|28275165|32564269|cr01.Contig162.wum.10.1|PT03767|MI01519|CL2552Contig1_1_AA|TX01792|MI08800|TX01914 |
| 321 | *M. incognita* | CL600Contig1_1_AA | 1 | 1.02 | ? | 8 | beta-1,4-endoglucanase [Meloidogyne incognita] | 0 | 13398416|MI02720|CL600Contig1_1_AA |
| 322 | *M. incognita* | CL7Contig2_1_AA | 1 | 2.04 | ? | 9 | Hypothetical protein CBG24722 [C. briggsae] | 0.009 | CL7Contig2_1_AA |
| 323 | *M. incognita* | 202c23r1.1_1_AA | 1 | 0.87 | ? | 9 | Hypothetical protein CBG20566 [C. briggsae AF16] | 6.00E-78 | 202c23r1.1_1_AA |
| 324 | *M. incognita* | 210e11c1.1_1_AA | 1 | 2.29 | A | 2 | ATP-dependent helicase DDX1, putative [Brugia malayi] | 5.00E-74 | 210e11c1.1_1_AA |
| 325 | *M. incognita* | CL2363Contig1_1_AA | 1 | 1.53 | E | 4 | Aspartyl aminopeptidase, putative [Brugia malayi] | 1.00E-70 | CL2363Contig1_1_AA |
| 326 | *M. incognita* | 203n01r1.1_1_AA | 1 | 0.76 | G | 4 | carbohydrate phosphorylase, putative [Brugia malayi] | 8.00E-105 | 203n01r1.1_1_AA |
| 327 | *M. incognita* | CL220Contig1_1_AA | 1 | 0.71 | I | 4 | 17beta-hydroxysteroid dehydrogenase [Heterodera glycines] | 2.00E-86 | CL220Contig1_1_AA |
| 328 | *M. incognita* | CL1345Contig1_1_AA | 1 | 0.46 | J | 6 | 40S ribosomal protein S11, putative [Brugia malayi] | 1.00E-62 | CL1345Contig1_1_AA |
| 329 | *M. incognita* | 226d21r1.1_1_AA | 1 | 3.05 | J | 6 | glutaminyl-tRNA synthetase, putative [Brugia malayi] | 3.00E-52 | 226d21r1.1_1_AA |
| 330 | *M. incognita* | 214m01c1.1_1_AA | 1 | 0.61 | O | 3 | similar to proteasome 26S non-ATPase subunit 4 isoform 2 [Strongylocentrotus purpuratus] | 0.15 | 214m01c1.1_1_AA |
| 331 | *M. incognita* | 217d04r1.1_1_AA | 1 | 1.83 | O | 3 | similar to ubiquitin-activating enzyme E1 [Nasonia vitripennis] | 1.00E-60 | 217d04r1.1_1_AA |
| 332 | *M. incognita* | CL2549Contig1_1_AA | 1 | 4.58 | OR | 3 | AGAP004294-PA [Anopheles gambiae str. PEST] | 2.00E-23 | CL2549Contig1_1_AA |
| 333 | *M. incognita* | CL279Contig1_1_AA | 1 | 0.26 | Q | 4 | glutathione reductase, putative [Brugia malayi] | 4.00E-74 | CL279Contig1_1_AA |
| 334 | *M. incognita* | CL2746Contig1_1_AA | 1 | 1.31 | R | 9 | conserved hypothetical protein [Brugia malayi] | 9.00E-53 | CL2746Contig1_1_AA |
| 335 | *M. incognita* | CL2942Contig1_1_AA | 1 | 1.02 | R | 9 | Hypothetical protein CBG14099 [C. briggsae AF16] | 2.00E-08 | CL2942Contig1_1_AA |
| 336 | *M. incognita* | CL2370Contig1_1_AA | 1 | 4.58 | T | 5 | protein KINase family member (kin-1) [C. elegans] | 8.00E-22 | CL2370Contig1_1_AA |
| 337 | *M. incognita* | 215a24r1.1_1_AA | 1 | 1.53 | U | 6 | coatomer alpha subunit , putative [Brugia malayi] | 5.00E-50 | 215a24r1.1_1_AA |
| 338 | *M. incognita* | CL358Contig1_1_AA | 1 | 3.05 | Z | 1 | Kinesin motor domain containing protein [Brugia malayi] | 7.00E-58 | CL358Contig1_1_AA |
| 339 | *M. incognita* | CL1149Contig1_1_AA | 1 | 4.58 | ? | 9 | No significant similarity found | No | CL1149Contig1_1_AA |
| 340 | *M. incognita* | CL2360Contig1_1_AA | 1 | 0.48 | ? | 8 | xylanase [Meloidogyne incognita] | 1.00E-65 | CL2360Contig1_1_AA |
| 341 | *M. incognita* | CL99Contig1_1_AA | 1 | 1.02 | ? | 9 | No significant similarity found | No | CL99Contig1_1_AA |
| 342 | *M. incognita* | 209l14r1.1_1_AA | 1 | 3.05 | ? | 9 | hypothetical protein Bm1_50160 [Brugia malayi] | 8.00E-18 | 209l14r1.1_1_AA |
| 343 | *M. incognita* | MI00879 | 1 | 0.31 | Z | 1 | Myosin tail family protein [Brugia malayi] | 2.00E-24 | MI00879|MP00497|MI06808 |
| 344 | *M. incognita* | MI02052 | 1 | 10.69 | ? | 9 | hypothetical protein MM_2923 [Methanosarcina mazei Go1] | 0.006 | MI02052 |
| 345 | *M. incognita* | MI02465 | 1 | 2.62 | A | 2 | Hypothetical protein CBG18686 [C. briggsae] | 4.00E-21 | MJ00819|MP00250|MI02465|MI02515|MA01280 |
| 346 | *M. incognita* | MI07708 | 1 | 7.64 | S | 9 | cytokine induced apoptosis inhibitor 1 [Mus musculus] | 2.00E-24 | MI07708 |
| 347 | *M. incognita* | MI01934 | 1 | 0.78 | C | 4 | NADP-specific isocitrate dehydrogenase [Candida albicans SC5314] | 7.00E-46 | MC03154|MI01934 |
| 348 | *M. incognita* | MI04721 | 1 | 2.82 | ? | 9 | No significant similarity found | No | MI04721 |
| 349 | *M. incognita* | MI07344 | 1 | 2.75 | A | 2 | Hypothetical protein CBG21723 [C. briggsae] | 2.00E-09 | MI07344 |
| 350 | *M. incognita* | MI01479 | 1 | 5.5 | J | 6 | large subunit ribosomal protein 16 [Pristionchus sp. 10 RS5133] | 4.00E-72 | MI01479|MA00213|MC00978|MC04337 |
| 351 | *M. incognita* | MI02218 | 1 | 0.81 | J | 6 | Ribosomal protein, small subunit protein 3 [C. elegans] | 9.00E-97 | MI02218|MI01064|MP00592|MC00908|HS00404|MJ03603 |
| 352 | *M. incognita* | MI00138 | 1 | 5.5 | RV | 2 | Hypothetical protein CBG07016 [C. briggsae] | 3.00E-44 | MC05730|MI00138|MI00335|MJ00323 |
| 353 | *M. incognita* | MI00242 | 1 | 0.39 | ? | 9 | hypothetical protein EUBVEN_01005 [Eubacterium ventriosum ATCC 27560] | 0.086 | MJ02240|MP00432|MI00242 |
| 354 | *M. incognita* | MI01914 | 1 | 6.87 | ? | 9 | No significant similarity found | No | MI01914|MA01699 |
| 355 | *M. incognita* | MI01755 | 1 | 3.06 | E | 4 | H-protein [Gallus gallus] | 1.00E-36 | MI01755 |
| 356 | *M. incognita* | MI04073 | 1 | 3.06 | E | 4 | Hypothetical protein CBG06199 [C. briggsae] | 1.00E-90 | MC01426|MI04073|MA00263 |
| 357 | *M. incognita* | MI02767 | 1 | 3.06 | O | 3 | ENSANGP00000009085 [Anopheles gambiae str. PEST] | 1.00E-28 | MI02767 |
| 358 | *M. incognita* | MI08334 | 1 | 1.41 | O | 3 | Hypothetical protein CBG10813 [C. briggsae] | 2.00E-76 | MI08334|MJ01028|MP01446 |
| 359 | *M. incognita* | MI08326 | 1 | 3.06 | R | 2 | RNA binding motif protein 8A, isoform CRA_a [Homo sapiens] | 2.00E-32 | MI08326|MJ01517|MH00600 |
| 360 | *M. incognita* | MI06265 | 1 | 0.06 | Z | 1 | Myosin tail family protein [Brugia malayi] | 1.00E-23 | MI06265 |
| 361 | *M. incognita* | MI05245 | 1 | 2.62 | ? | 9 | Hypothetical protein CBG05010 [C. briggsae] | 3.00E-47 | MP00104|MA01757|MJ03272|MI05245|MP01379|MI00779|MJ00267 |
| 362 | *M. incognita* | MI06174 | 1 | 2.04 | ? | 3 | Aspartyl protease inhibitor precursor (Tco-API-1) | 2.00E-27 | MI06174|MP02100 |
| 363 | *M. incognita* | MI02078 | 1 | 0.83 | A | 2 | similar to Sm protein G isoform 2 [Gallus gallus] | 3.00E-24 | MJ00156|MC00718|MH02347|MI02078|MJ01455|PT00615|MI08667 |
| 364 | *M. incognita* | MI02098 | 1 | 4.58 | BD | 2 | putative nucleosome binding protein [Anisakis simplex] | 5.00E-51 | MP01140|MI02098|MJ03518|MI07957 |
| 365 | *M. incognita* | MI02034 | 1 | 1.31 | F | 4 | Hypothetical protein CBG14689 [C. briggsae] | 3.00E-37 | MI02034 |
| 366 | *M. incognita* | MI00978 | 1 | 1.83 | GC | 4 | hypothetical protein [Strongylocentrotus purpuratus] | 4.00E-63 | MI00978|MH02218|MJ01133|MI00103|MC00918|MI06977 |
| 367 | *M. incognita* | MI00906 | 1 | 1.83 | J | 6 | small subunit ribosomal protein 24 [Koerneria sp. RS1982] | 4.00E-38 | MP02001|MJ00738|MJ00734|MP00482|MI00906|MC01181|MP02010 |
| 368 | *M. incognita* | MI01802 | 1 | 1.31 | J | 6 | 40S ribosomal protein S23 [C. elegans] | 1.00E-63 | RS00119|MJ00550|MA00512|MJ00747|GP00384|HG00785|MI01802|MI01980|MP00438|TX00282|MI00693|MC00905|MH02590 |
| 369 | *M. incognita* | MI02234 | 1 | 1.02 | J | 6 | hypothetical protein CC1G_05715 [Coprinopsis cinerea okayama7#130] | 6.00E-22 | MA01050|MC01125|MA00023|MH02578|MI02234|MI02493|MP00602|MC00825|MA00020|MP00169 |
| 370 | *M. incognita* | MI02344 | 1 | 0.16 | J | 6 | translation elongation factor aEF-2, putative [Brugia malayi] | 4.00E-103 | HS01272|MI02344|MH00519 |
| 371 | *M. incognita* | MI05700 | 1 | 1.31 | J | 6 | Elongation factor 1 gamma, conserved domain containing protein | 2.00E-19 | MI05700 |
| 372 | *M. incognita* | MI06613 | 1 | 2.29 | JT | 6;5 | Hypothetical protein CBG03991 [C. briggsae] | 1.00E-58 | MI06613 |
| 373 | *M. incognita* | MI01515 | 1 | 0.11 | K | 2 | Nascent polypeptide-associated complex subunit alpha (NAC-alpha) [C. elegans] | 2.00E-45 | MI01515|MP01401|MJ00947 |
| 374 | *M. incognita* | MI07562 | 1 | 2.29 | KLB | 2 | HMG family member (hmg-4) [C. elegans] | 2.00E-50 | MI07562|MH05395 |
| 375 | *M. incognita* | MI01648 | 1 | 1.02 | O | 3 | R09E12.3 [C. elegans] | 8.00E-71 | MI01648|MA01865|MH00186|MJ02335 |
| 376 | *M. incognita* | MI09158 | 1 | 3.05 | O | 3 | Ubiquitin-like protein SMT3, putative [Brugia malayi] | 5.00E-16 | MI09158|MI01871|MI07711|MJ00731|MJ00008|MP00368 |
| 377 | *M. incognita* | MI09152 | 1 | 0.31 | R | 9 | HMG family member (hmg-1.1) [C. elegans] | 4.00E-15 | MJ00085|PE00482|MP00681|MP02151|MI09152|MI02283|MI02467|MJ00124|MJ00530|MP00244|MH02699|MJ00084|MI08860|MH10401|MI08833 |
| 378 | *M. incognita* | MI01602 | 1 | 1.31 | T | 5 | CPK31 (calcium-dependent protein kinase 31) [Arabidopsis thaliana] | 1.00E-07 | MI01602|MJ03859|MJ01898|MI08413|MH01458 |
| 379 | *M. incognita* | MI04973 | 1 | 0.51 | U | 6 | Synaptobrevin-1 [C. briggsae] | 7.00E-55 | cr01.Contig1.wum.87.1|XI04818|HG00760|SS01502|XI01166|MH02536|NB00813|TS00133|GR01011|14992.m11406|NB00899|AE00730|HC00604|17563282|2196766|2196768|3193208|39594345|SR00214|TS05785|TS00705|MI04973|TS00574 |
| 380 | *M. incognita* | MI00316 | 1 | 9.16 | ? | 3 | matrix metalloproteinase [Heterodera glycines] | 2.00E-15 | MI00316 |
| 381 | *M. incognita* | MI01173 | 1 | 0.38 | ? | 9 | transthyretin-like protein 2 precursor [Radopholus similis] | 2.00E-38 | MI01173 |
| 382 | *M. incognita* | MI01389 | 1 | 3.05 | ? | 9 | Conserved hypothetical protein, putative [Brugia malayi] | 2.00E-10 | MJ00107|MI01389|MH00983|MI02228 |
| 383 | *M. incognita* | MI02310 | 1 | 4.58 | ? | 9 | Putative 26 proteasome complex subunit sem1 [C. elegans] | 2.2 | MP01497|MI02310 |
| 384 | *M. incognita* | MI03736 | 1 | 4.58 | ? | 9 | No significant similarity found | No | MI03736 |
| 385 | *M. incognita* | MI03884 | 1 | 9.16 | ? | 9 | Hypothetical protein CBG18765 [C. briggsae] | 1.00E-04 | MI03884|MJ04461|MJ04466 |
| 386 | *M. incognita* | MI04342 | 1 | 1.02 | ? | 3 | cysteine protease [Aster tripolium] | 0.65 | MI04342 |
| 387 | Other worms | 115534393 | 7 | 9.76 | B | 2 | similar to germinal histone H4 gene [Canis familiaris] | 9.00E-43 | 115534393|17534747|17534755|17537811|17539964|17540632|17541086|17559296|17561984|17561990|17562000|17562012|17562018|17568543|18376579|3873698|3875125|3876196|3877575|3879736|3880073|3881588|3881590|51317324|225950|17538226 |
| 388 | Other worms | 17561988 | 4 | 5.67 | B | 2 | HIStone family member (his-4) [C. briggsae] | 1.00E-55 | 17561988|3879733|17559286|17559300|17540950|3873704|3877576|3877984|45476816|17561996|17562004|17562008|45476817|17558794|3875123|45476815|17532989|17534749|17534757|17541092|3875623|3881581|3881583|45644941|17540654|71994490 |
| 389 | Other worms | 58419220 | 4 | 55003 | K | 2 | DNA-directed RNA polymerase, fusion of beta and beta' subunits [Wolbachia endosymbiont strain TRS] | 0 | 58419220|58584904|75497690 |
| 390 | Other worms | MH00967 | 3 | 7.05 | DR | 9 | lectin associated matrix protein [Coturnix japonica] | 8.00E-35 | MH00967|MH04974 |
| 391 | Other worms | 14990.m07633 | 3 | 0.34 | C | 4 | Hypothetical protein CBG08717 [C. briggsae] | 0 | 14990.m07633|3878073|71988063|75029335|39580722|cr01.Contig6.wum.8.1|42733088|71988080|HS01167|31441843|71988074|MC00179|SR00961|MH10530|PT00179 |
| 392 | Other worms | MA01689 | 3 | 7.86 | P | 4 | Y37A1B.5 [C. elegans] | 5.00E-59 | MA01689|MJ01967 |
| 393 | Other worms | MH03105 | 3 | 5.09 | U | 6 | hypothetical protein LOC613110 [Xenopus tropicalis] | 6.00E-66 | MH03105 |
| 394 | Other worms | 1350745 | 3 | 6.55 | Z | 1 | Actin-2 [Onchocerca volvulus] | 3.00E-77 | 1350745 |
| 395 | Other worms | MA00435 | 3 | 5.24 | O | 3 | conserved hypothetical protein [Aedes aegypti] | 1.00E-23 | MA00435 |
| 396 | Other worms | MH09264 | 3 | 1.75 | O | 3 | proteasome Regulatory Particle, ATPase-like family member (rpt-4) [C. elegans] | 4.00E-107 | MH09264|XI04334|15617798|21264496|71987364|55710062|71987372|7499759 |
| 397 | Other worms | AE03100 | 2 | 1.81 | ? | 9 | type II keratin E3 [Oncorhynchus mykiss] | 5.00E-29 | AE03100 |
| 398 | Other worms | AS01127 | 2 | 20.16 | Z | 1 | actin 2, putative [Brugia malayi] | 9.00E-77 | AS01127|AS00003|AS03166|AS02932|AS02630|AS01783|AS02135|AS03113|AS00162|AS01619|AS01350|AE03060|AE03386|AS00113|AS00632|AE00612|AS03386|AS04250|AS04222|AS00396|AS03385|AS09318|AS08532|AS04092 |
| 399 | Other worms | GP02221 | 2 | 7.5 | T | 5 | Hypothetical protein CBG22810 [C. briggsae] | 6.00E-104 | GP02221 |
| 400 | Other worms | 121998 | 2 | 2.37 | B | 2 | HIStone family member (his-68) [C. elegans] | 5.00E-65 | 121998 |
| 401 | Other worms | 17562198 | 2 | 54.95 | C | 4 | ALdehyde deHydrogenase family member (alh-2) [C. elegans] | 0 | 17562198|5921971|16950426|25144435|39587434|cr01.Contig27.wum.36.1|24636031|32564736|SR03212 |
| 402 | Other worms | MP00648 | 2 | 9.17 | O | 3 | Hypothetical protein CBG05716 [C. briggsae] | 3.00E-45 | MP00648 |
| 403 | Other worms | cr01.Contig195.wum.1.1 | 2 | 45836 | ? | 9 | Hypothetical protein CBG15443 [C. briggsae AF16] | 2.00E-97 | cr01.Contig195.wum.1.1|39586634 |
| 404 | Other worms | MH04448 | 2 | 36669 | EO | 4;3 | aminopeptidase [Heterodera glycines] | 2.00E-54 | MH04448 |
| 405 | Other worms | 82620076 | 2 | 36669 | J | 6 | EF1A_TRIRE ELONGATION FACTOR 1-ALPHA (EF-1-ALPHA) [Gibberella zeae PH-1] | 1.00E-44 | 82620076|82620078|82620084|82620090|82620186|82620796|134284918|82620094|82620092|82620080|134270192|134270198|134270253|134270275|134270283|134270285|134270287|MP02095|134284898|134284896|134284890|134284892|134284894|134284900|134284924|134284920 |
| 406 | Other worms | MA01524 | 2 | 0.89 | J | 6 | F58G1.1 [C. elegans] | 1.00E-04 | MA01524 |
| 407 | Other worms | PP00571 | 2 | 36669 | O | 3 | 14-3-3b protein [Meloidogyne incognita] | 8.00E-110 | PP00571 |
| 408 | Other worms | MP01130 | 2 | 1.83 | Z | 1 | beta-tubulin [Haemonchus contortus] | 7.00E-121 | MP01130|XI00865 |
| 409 | Other worms | 17569439 | 2 | 36669 | ? | 9 | Sex determination and Dosage Compensation defect family member (sdc-2) [C. elegans] | 0 | 17569439 |
| 410 | Other worms | MP00988 | 2 | 3.06 | AJ | 2;6 | W01B11.3 [C. elegans] | 1.00E-70 | MP00988|MJ03076 |
| 411 | Other worms | 39582247 | 2 | 27.47 | O | 3 | Hypothetical protein CBG08827 [C. briggsae AF16] | 0 | 39582247|17562024|1825782|2851603|762922 |
| 412 | Other worms | MA00799 | 2 | 4.58 | O | 3 | Hypothetical protein CBG04294 [C. briggsae] | 2.00E-30 | MA00799|MH01905 |
| 413 | Other worms | MJ03706 | 2 | 27502 | R | 2 | Putative helicase C28H8.3 [C. elegans] | 9.00E-31 | MJ03706 |
| 414 | Other worms | 14992.m11170 | 2 | 9.16 | T | 5 | Protein Phosphatase 2A (Two A) Regulatory subunit family member (pptr-1) [C. elegans] | 0 | 14992.m11170|SS01250|115534753|82658174|cr01.Contig42.wum.73.1 |
| 415 | Other worms | PP00360 | 2 | 3.06 | B | 2 | HisTone variant H2AZ homolog family member (htz-1) [C. elegans] | 3.00E-61 | PP00360|AS01521|GR00766|MJ00556|MP00492|MH01711|cr01.Contig33.wum.181.1|39587596|1397320|17541830|74967342|14972.m07436|HC00403|MC00063|MC01032|RS00629|MH10232|SS03510 |
| 416 | Other worms | 17570289 | 2 | 18335 | E | 4 | W07E11.1 [C. elegans] | 0 | 17570289|3879588|3880576|39590998|14972.m07899|cr01.Contig36.wum.27.1 |
| 417 | Other worms | MP00119 | 2 | 4.58 | E | 4 | Hypothetical protein CBG24539 [C. briggsae] | 8.00E-64 | MP00119 |
| 418 | Other worms | MJ04053 | 2 | 6.11 | G | 4 | Hypothetical protein CBG18265 [C. briggsae] | 3.00E-26 | MJ04053 |
| 419 | Other worms | MJ00281 | 2 | 2.04 | JK | 6;2 | hypothetical protein C17G10.8 [C. elegans] | 3.00E-69 | MJ00281 |
| 420 | Other worms | MA02593 | 2 | 2.29 | L | 2 | UV excision repair protein Rad23 containing protein [Brugia malayi] | 8.00E-16 | MA02593 |
| 421 | Other worms | cr01.Contig10.wum.49.1 | 2 | 9.16 | O | 3 | Hypothetical protein CBG18577 [C. briggsae AF16] | 9.00E-87 | cr01.Contig10.wum.49.1|39586917|1109668|11514370|15988249|1706248|17559074|3881312|9257006|AE01120|AE01335|39582839|6004521 |
| 422 | Other worms | MJ03129 | 2 | 1.83 | O | 3 | hypothetical protein F46E10.7 [C. elegans] | 6.00E-35 | MJ03129 |
| 423 | Other worms | MP00052 | 2 | 1.83 | O | 3 | 20S proteasome alpha5 subunit, putative [Brugia malayi] | 1.00E-26 | MP00052|MJ04192 |
| 424 | Other worms | MH02402 | 2 | 4.58 | Q | 4;7 | glutathione synthetase [Aedes aegypti] | 7.00E-14 | MH02402|MP00377 |
| 425 | Other worms | MA01831 | 2 | 18335 | V | 9 | Similar to squamous cell carcinoma antigen [Bos taurus] | 4.00E-24 | MA01831 |
| 426 | Other worms | MC01039 | 1 | 2.28 | B | 2 | Histone H2A [Danio rerio] | 7.00E-46 | MC01039|PE00071|MC00292|MH04040|MH00300|MJ04645|13648.m00017|14538.m00482|MP01035|MC02608|AS03042|TX01835|MJ00769|MA02732|MP00483|MH02252 |
| 427 | Other worms | GP01134 | 1 | 3.86 | O | 3 | chaperonine protein HSP60 [Onchocerca volvulus] | 1.00E-107 | GP01134 |
| 428 | Other worms | MH02927 | 1 | 4.58 | T | 5 | Hypothetical protein CBG21904 [C. briggsae] | 7.00E-39 | MH02927|MH01897|MH04791 |
| 429 | Other worms | AE03258 | 1 | 2.79 | J | 6 | 40S ribosomal protein S28 [Ostertagia ostertagi] | 5.00E-21 | AE03258|AE01662|AC00703|AE02379|AE03240|AE03312|AC03757|AC03843|AS05114|2995699|51316569|AS01182|AS04693|AS05968|AS05025|AE02614|AC04179|AS02416|AS05781|AS00209|AS03866|AE01773|AS03451|AE01951|AS00176 |
| 430 | Other worms | MJ03828 | 1 | 8.02 | O | 3 | proteasome Regulatory Particle, Non-ATPase-like family member (rpn-1) [C. elegans] | 5.00E-44 | MJ03828|MA01249|MH06298 |
| 431 | Other worms | SS01375 | 1 | 16.04 | O | 3 | similar to tyrosine 3/tryptophan 5 -monooxygenase activation protein, zeta polypeptide [Macaca mulatta] | 8.00E-108 | SS01375 |
| 432 | Other worms | XI01177 | 1 | 64170 | OR | 3 | hypothetical protein [Pan troglodytes] | 3.00E-155 | XI01177|TS01138|XI00074|TS05105|AS03377|TS00600|XI04835|AS10005|TS00854|HC04150|XI00181|TS02206|XI01914|XI01316|SS01416|SR00940|TS03200|TS00037|XI04564 |
| 433 | Other worms | MP00840 | 1 | 3.82 | O | 3 | Hypothetical protein CBG03212 [C. briggsae] | 2.00E-73 | MP00840|MP02090 |
| 434 | Other worms | MC01143 | 1 | 1.24 | ? | 9 | hypothetical protein F53A9.10 - C. elegans | 1.00E-08 | MC01143 |
| 435 | Other worms | cr01.Contig2470.wum.1.1 | 1 | 36669 | C | 4 | Hypothetical protein CBG18746 [C. briggsae AF16] | 7.00E-116 | cr01.Contig2470.wum.1.1 |
| 436 | Other worms | MH08963 | 1 | 36669 | G | 4 | Glucose-6-Phosphate Isomerase family member (gpi-1) [C. elegans] | 2.00E-98 | MH08963 |
| 437 | Other worms | 58418917 | 1 | 36.63 | J | 6 | Translation elongation factor EF-Tu, GTPase [Wolbachia endosymbion strain TRS] | 0 | 58418917|58584601|58419226|58584910 |
| 438 | Other worms | 12276043 | 1 | 27502 | J | 6 | 60S ribosomal protein L44 L41 [C. elegans] | 2.00E-53 | 12276043 |
| 439 | Other worms | XI00124 | 1 | 9.16 | J | 6 | ribosomal protein L11 [Ixodes scapularis] | 4.00E-81 | XI00124|XI01171 |
| 440 | Other worms | MC05088 | 1 | 5.5 | L | 2 | C. briggsae CBR-PCN-1 protein | 2.00E-62 | MC05088 |
| 441 | Other worms | 14652.m00401 | 1 | 13.74 | O | 3 | Hypothetical protein CBG21817 [C. briggsae] | 0 | 14652.m00401|cr01.Contig10.wum.337.1|14574231|17564182|39581773|27885056|32566944|SR03242|AC_59239214.eannot.1004 |
| 442 | Other worms | MH03063 | 1 | 3.93 | O | 3 | Hypothetical protein CBG03440 [C. briggsae] | 5.00E-40 | MH03063 |
| 443 | Other worms | MP00833 | 1 | 1.31 | P | 4 | EXCretory canal abnormal family member (exc-4) [C. elegans] | 2.00E-53 | MP00833|MH04181 |
| 444 | Other worms | 14972.m07376 | 1 | 2.75 | T | 5 | PREDICTED: hypothetical protein [Monodelphis domestica] | 2.00E-173 | 14972.m07376|OS01655|OS01586|GP01881|MJ03742|AE02943|AC_59215778.eannot.1005|AC01689 |
| 445 | Other worms | MP00337 | 1 | 1.31 | TU | 5;6 | Hypothetical protein CBG01077 [C. briggsae] | 3.00E-93 | MP00337 |
| 446 | Other worms | HC02566 | 1 | 3.44 | Z | 1 | actin [Limulus polyphemus] | 2.00E-53 | HC02566 |
| 447 | Other worms | TX00152 | 1 | 27502 | Z | 1 | Actin-2 [Onchocerca volvulus] | 8.00E-152 | TX00152 |
| 448 | Other worms | SR01877 | 1 | 27502 | ? | 9 | E1-E2 ATPase family protein [Tetrahymena thermophila SB210] | 0.56 | SR01877 |
| 449 | Other worms | 14992.m11057 | 1 | 18335 | A | 2 | Poly polymerase central domain containing protein [Brugia malayi] | 0 | 14992.m11057 |
| 450 | Other worms | MJ00054 | 1 | 0.5 | C | 4 | Malate DeHydrogenase family member (mdh-1) [C. elegans] | 5.00E-59 | MJ00054 |
| 451 | Other worms | GR00467 | 1 | 18.32 | I | 4 | 3-ketoacyl-coa thiolase protein 1 [C. elegans] | 7.00E-43 | GR00467 |
| 452 | Other worms | AE00828 | 1 | 18335 | J | 6 | hypothetical protein FG09874.1 [Gibberella zeae PH-1] | 3.00E-55 | AE00828|OS01457|NB00236|AC04584|HC01621 |
| 453 | Other worms | MP01475 | 1 | 2.04 | K | 2 | similar to elongation factor SIII p15 subunit [Pan troglodytes] | 5.00E-48 | MP01475|MJ01017|MH09294|MC00571 |
| 454 | Other worms | cr01.Contig0.wum.390.1 | 1 | 18335 | O | 3;7 | Probable protein disulfide-isomerase A6 precursor [C. elegans] | 0 | cr01.Contig0.wum.390.1 |
| 455 | Other worms | MH05001 | 1 | 18.32 | O | 3 | Hypothetical protein CBG12090 [C. briggsae] | 8.00E-49 | MH05001 |
| 456 | Other worms | MJ00925 | 1 | 18335 | P | 2 | Hypothetical protein CBG12026 [C. briggsae] | 5.00E-59 | MJ00925|MH10214 |
| 457 | Other worms | 31043761 | 1 | 18335 | T | 5 | F25F2.1b [C. elegans] | 0 | 31043761|71984957 |
| 458 | Other worms | 17505619 | 1 | 18335 | ? | 9 | C17E4.2 [C. elegans] | 0 | 17505619|3874365 |
| 459 | Other worms | cr01.Contig1372.wum.1.1 | 1 | 18335 | ? | 9 | putative transposase [Acinetobacter baumannii ATCC 17978] | 8.00E-65 | cr01.Contig1372.wum.1.1 |
| 460 | Other worms | cr01.Contig174.wum.13.1 | 1 | 18335 | ? | 9 | Hypothetical protein CBG13654 [C. briggsae AF16] | 2.00E-103 | cr01.Contig174.wum.13.1 |
| 461 | Other worms | MH11009 | 1 | 1.41 | ? | 9 | flagellar motor switch protein [Helicobacter acinonychis str. Sheeba] | 3.2 | MH11009 |
| 462 | Other worms | 14459.m00240 | 1 | 1.83 | AR | 2 | Hypothetical protein CBG05583 [C. briggsae] | 4.00E-82 | 14459.m00240 |
| 463 | Other worms | 14992.m10966 | 1 | 0.65 | C | 4 | Vacuolar ATP synthase catalytic subunit A (V-ATPase subunit A) [C. briggsae] | 0 | 14992.m10966|cr01.Contig13.wum.322.1|39589917|74791913|17565854|4008438|75028911|SR00518|HS01043|AS10246|TS01564|71057507|90660178 |
| 464 | Other worms | MA02280 | 1 | 3.05 | E | 4 | Hypothetical protein C10C5.3 [C. elegans] | 1.00E-53 | MA02280 |
| 465 | Other worms | MA01250 | 1 | 0.83 | GMO | 4;3 | Hypothetical protein CBG08162 [C. briggsae] | 8.00E-36 | MA01250|MH01397 |
| 466 | Other worms | MA00657 | 1 | 2.29 | J | 6 | aspartyl(D) tRNA Synthetase family member (drs-1) [C. elegans] | 1.00E-72 | MA00657|MJ02483 |
| 467 | Other worms | MC00909 | 1 | 0.51 | J | 6 | 40S ribosomal protein S18, putative [Brugia malayi] | 5.00E-62 | MC00909 |
| 468 | Other worms | MH04679 | 1 | 3.05 | J | 6 | eukaryotic translation elongation factor 1 gamma [Macaca mulatta] | 2.00E-52 | MH04679 |
| 469 | Other worms | MH04769 | 1 | 0.65 | O | 3 | Heat shock protein protein 12.2 [C. elegans] | 1.00E-25 | MH04769|MH02405|MP00509 |
| 470 | Other worms | MP01239 | 1 | 2.29 | O | 3 | Probable 26S proteasome non-ATPase regulatory subunit 3, putative [Brugia malayi] | 1.00E-41 | MP01239|MJ02948 |
| 471 | Other worms | MH02369 | 1 | 1.53 | R | 2 | Hypothetical protein CBG21324 [C. briggsae] | 2.00E-45 | MH02369 |
| 472 | Other worms | MJ05164 | 1 | 1.31 | R | 7 | thioredoxin [Ascaris suum] | 7.00E-43 | MJ05164|MP01763 |
| 473 | Other worms | MP00760 | 1 | 1.83 | S | 9 | similar to CG1433-PA [Tribolium castaneum] | 1.00E-07 | MP00760 |
| 474 | Other worms | MP01299 | 1 | 2.29 | S | 9 | Y25C1A.7c [C. elegans] | 2.00E-40 | MP01299 |
| 475 | Other worms | MC00834 | 1 | 4.58 | U | 6 | F14E5.2b [C. elegans] | 7.00E-52 | MC00834 |
| 476 | Other worms | MJ02223 | 1 | 0.44 | U | 6 | Clathrin Heavy Chain family member (chc-1) [C. elegans] | 8.00E-67 | MJ02223 |
| 477 | Other worms | 5833216 | 1 | 0.65 | ? | 9 | unknown [Heterodera glycines] | 7.00E-110 | 5833216 |
| 478 | Other worms | 126153873 | 1 | 1.31 | ? | 8 | cellulose binding protein precursor [Meloidogyne arenaria] | 1.00E-92 | 126153873|126153867 |
| 479 | Other worms | MH01800 | 1 | 0.14 | ? | 9 | conserved hypothetical protein [Tetrahymena thermophila SB210] | 0.004 | MH01800 |
| 480 | Other worms | MH05913 | 1 | 1.83 | ? | 9 | cuticlin-1 [Meloidogyne artiellia] | 0.001 | MH05913 |
| 481 | Other worms | MJ02674 | 1 | 1.15 | ? | 9 | Hypothetical protein CBG20772 [C. briggsae] | 0.004 | MJ02674 |
| 482 | *Lycopersicon esculentum* | Unknown | 3 | 119172 | ? | 3 | Lemir | 0 | 2654440 |
| 483 | *Zea mays* | Unknown | 3 | 100.74 | B | 2 | Histone H2B | 0 | 1708107|3913804|399853|399854|1346251 |
| 484 | *Arabidopsis thaliana* | Unknown | 2 | 36669 | G | 4 | glyceraldehyde-3-phosphate dehydrogenase | 0 | 21618027|15219206|27754473|4966351 |
| 485 | *Arabidopsis thaliana* | Unknown | 2 | 55003 | B | 2 | Histone H2A | 0 | 27311795 |
| 486 | *Arabidopsis thaliana* | Unknown | 1 | 45836 | C | 4 | citrate (SI)-synthase | 0 | 15231128 |
