# Supplementary material for: Direct Identification of the Meloidogyne incognita Secretome Reveals Proteins with Host Cell Reprogramming Potential
Source: PLoS Pathog. 2008 Oct 31;4(10):e1000192. doi: 10.1371/journal.ppat.1000192 (PMC2568823; doi:10.1371/journal.ppat.1000192)
Supplement: Table S2 — Ranked list of secreted Meloidogyne incognita proteins identified by single peptides. All proteins were identified by at least two mass spectra using a filtering criterion of 0.1% FDR at the peptide level. (7.87 MB DOC) [file ppat.1000192.s002.doc]

**Supplementary Table S2:** **Ranked list of secreted *Meloidogyne incognita* proteins identified by single peptides.**

^1^ Peptide sequence map annotation:

/: Red forward-slashes for locations of y-ions

\: Blue backslashes for locations of b-ions

|: Magenta pipes (vertical lines) for locations of both b- and y-ions

| Protein number | Organism | Corresponding *M. incognita* Contig or accession nb. | Peptide Sequence Map ^1^ | MS/MS spectrum |
| --- | --- | --- | --- | --- |
| 268 | *M. incognita* | 210d13r1.1_1_AA | (R)D I/T D/A|S/V|Y E|Q|Y|A|L|P K(L) | 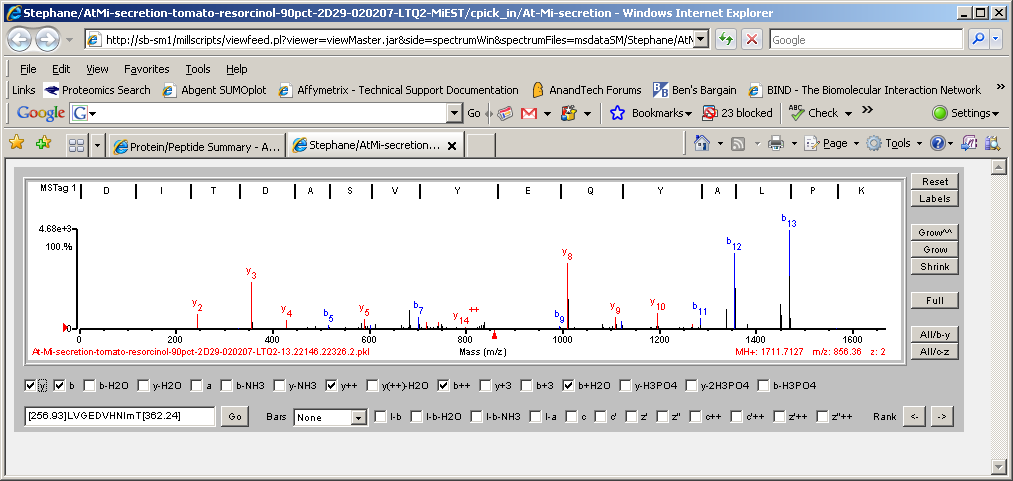 |
| 269 | *M. incognita* | 210k07r1.1_1_AA | (R)S D/S E D/G/F|P/G Q|G F|P/P/P/P P W L K(N) | 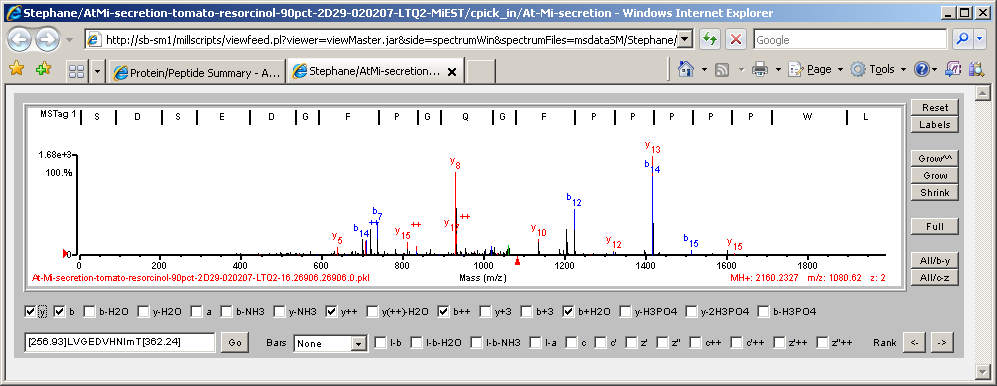 |
| 270 | *M. incognita* | CL1689Contig1_1_AA | (R)I S/L/G/L|P V/G/A/V|I/N/C/A/D N T G A K(N) | 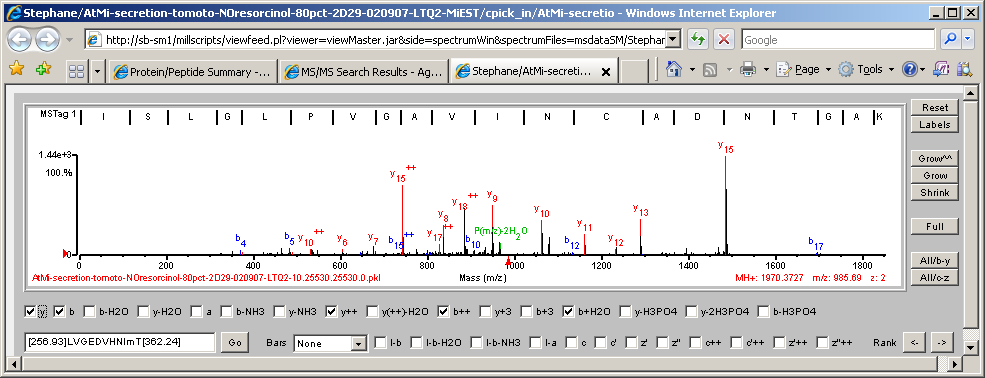 |
| 271 | *M. incognita* | CL895Contig1_1_AA | (K)A D/S/I|P/A D|A|Q A/E/A/V/S V E K(Q) | 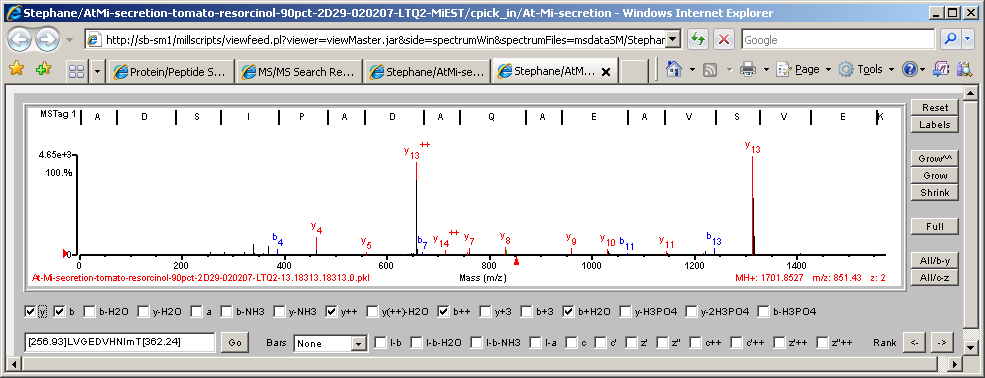 |
| 272 | *M. incognita* | 204h01r1.1_1_AA | (K)A P/F/G/F|P/G|P Q|G|P Y/Y|C G V|G A N K(A) | 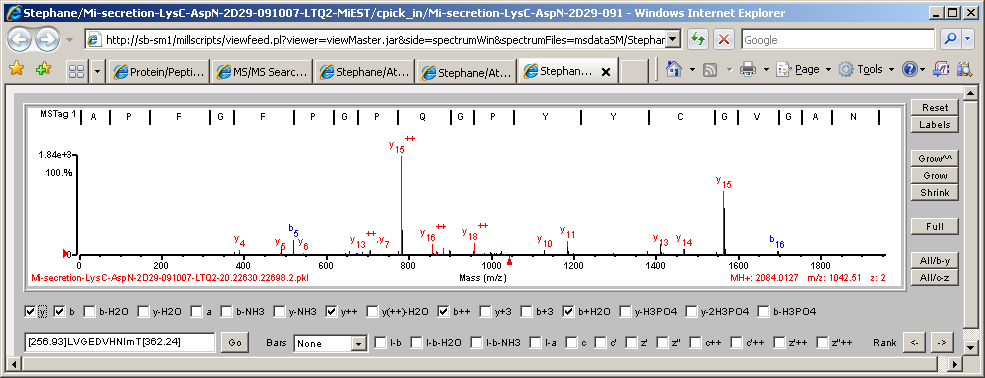 |
| 273 | *M. incognita* | CL2258Contig1_1_AA | (K)L/P/G Y|S/D|L/V|D|D|E|G N\I K(G) | 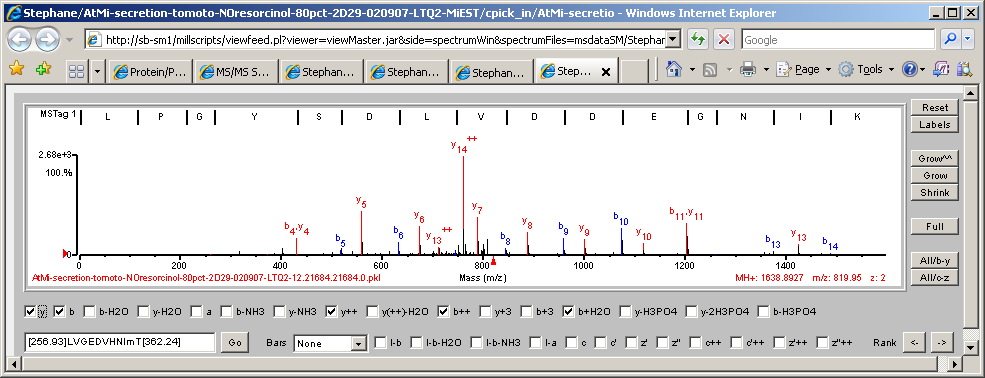 |
| 274 | *M. incognita* | CL381Contig1_1_AA | (K)L E/E/T/N E/N/K L\E|E|P I L\E\K(I) | 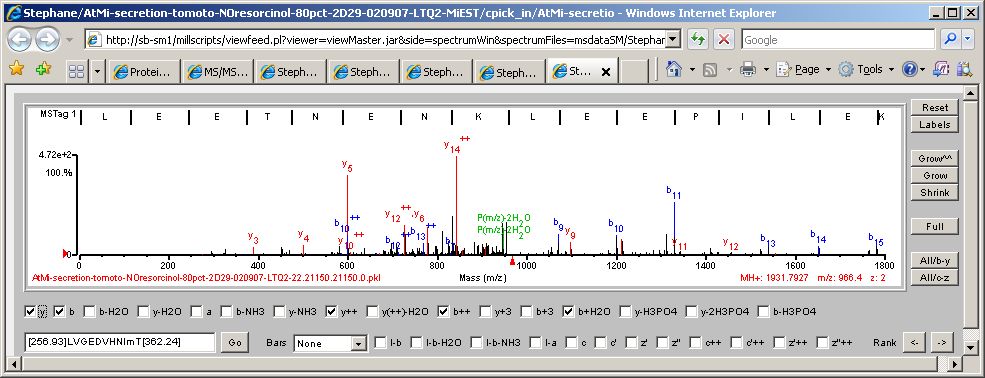 |
| 275 | *M. incognita* | CL378Contig1_1_AA | (K)I/P/I/F/S/A/A G/L|P H/N\E|I/A/A Q I V R(Q) | 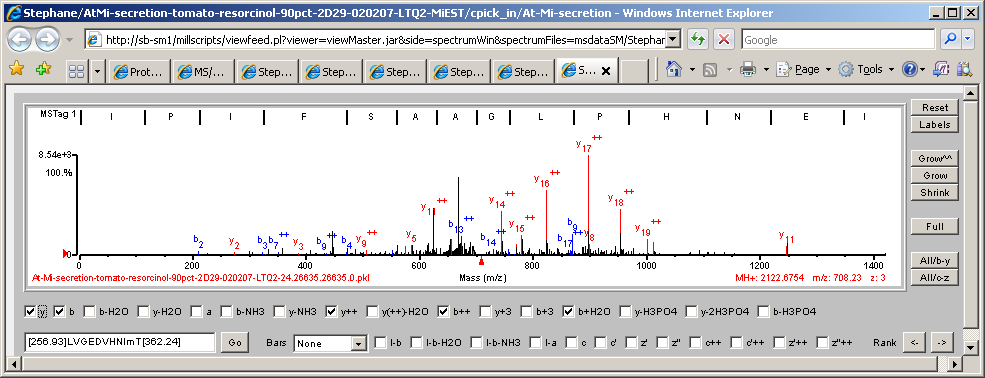 |
| 276 | *M. incognita* | 203p11r1.1_1_AA | (K)D S/V/D|S|V|D|P E I|Y A L\L K(K) | 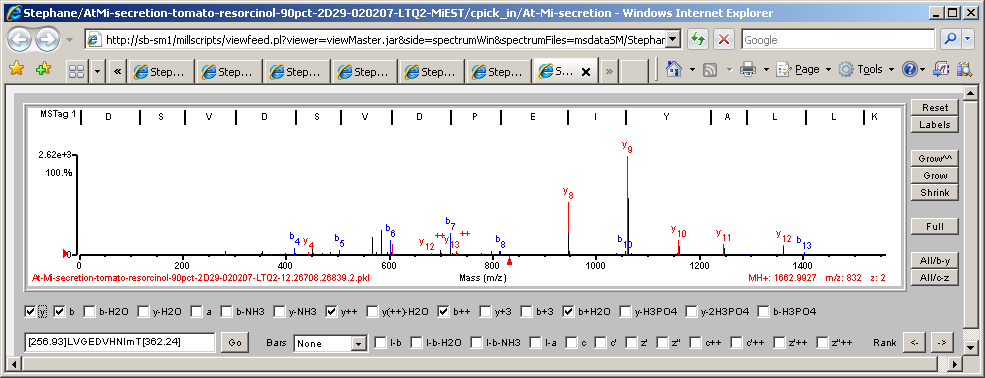 |
| 277 | *M. incognita* | CL1630Contig1_1_AA | (R)F/Q/Q|L|V|D|D/Y/V|E/Q\E T\K(A) | 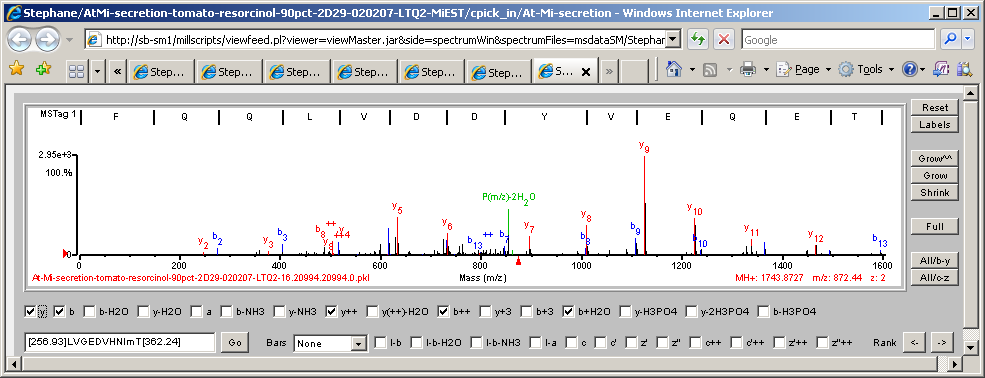 |
| 278 | *M. incognita* | CL129Contig1_1_AA | (K)H/H|A/T/Y/V|N\N L|N M\T E E\K(I) | 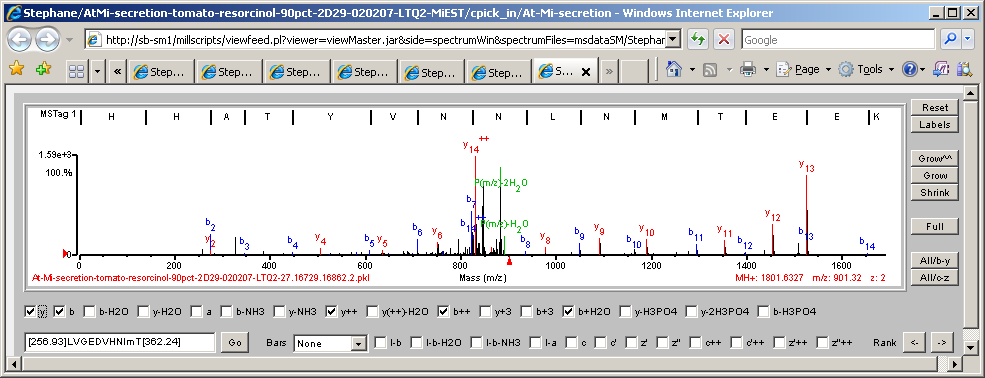 |
| 279 | *M. incognita* | CL520Contig2_1_AA | (K)E L/D D D E|I|V E|N|P A\Y|I\P K(Q) | 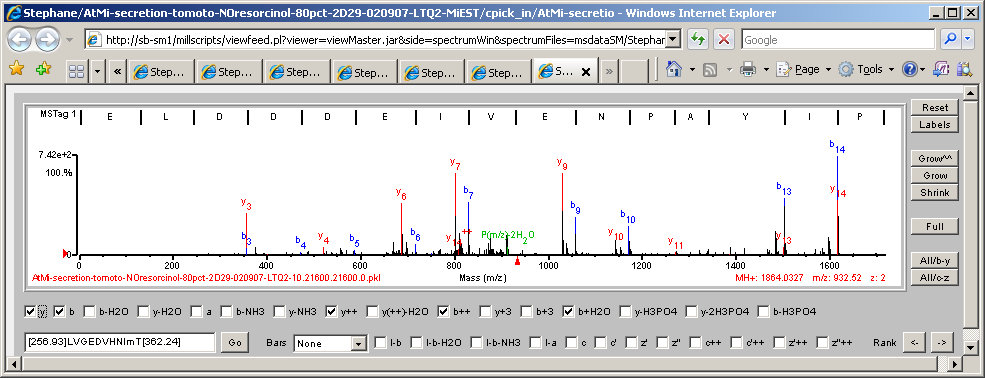 |
| 280 | *M. incognita* | 206l24r1.1_1_AA | (K)V P/G/A/P/T A V/T D|H|G V|P V D|E F G L\P\K(I) | 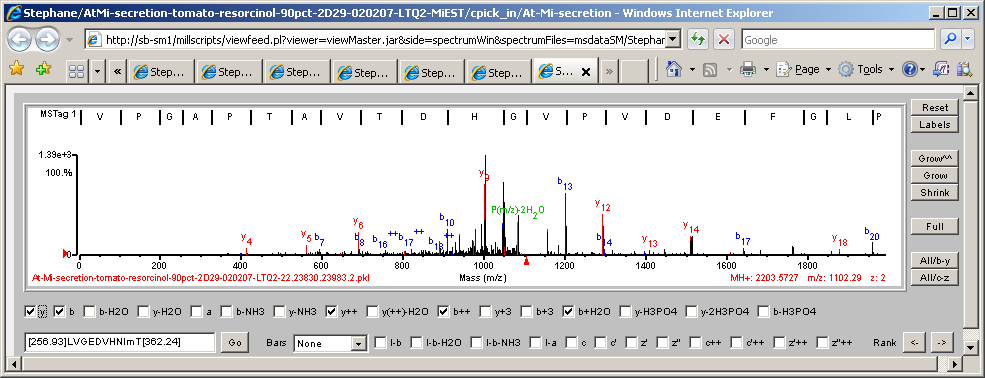 |
| 281 | *M. incognita* | CL500Contig1_1_AA | (K)G A D G D/F|T|E|F|P V|P E Q\F\K(T) | 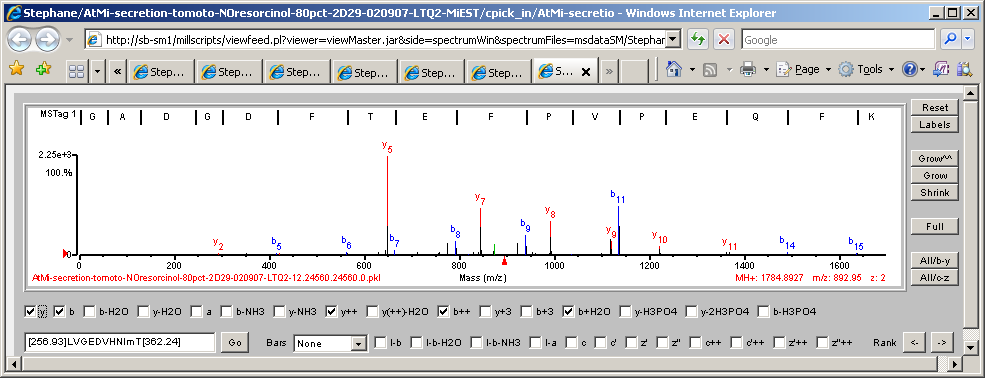 |
| 282 | *M. incognita* | CL139Contig1_1_AA | (R)S D/D/K/E|A|S/A|A|D/E|A/Q|S Q Q Q\K(I) | 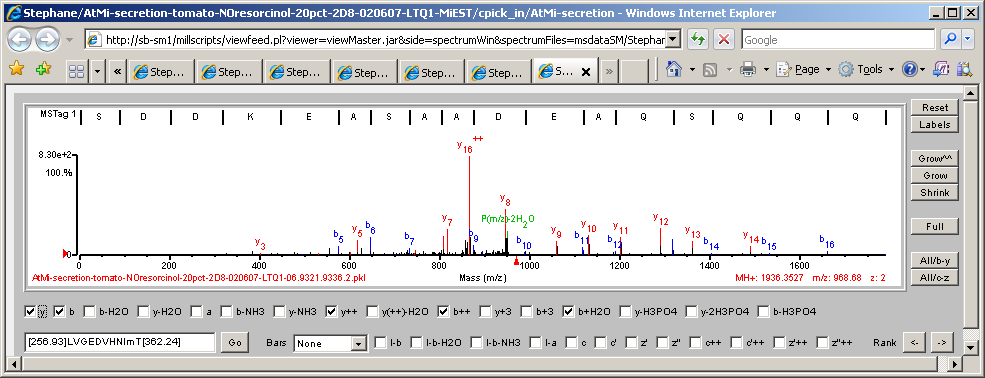 |
| 283 | *M. incognita* | CL46Contig1_1_AA | (K)V/E/N/S/D|P/A|G/E|V|G/D|D|G G S K(F) | 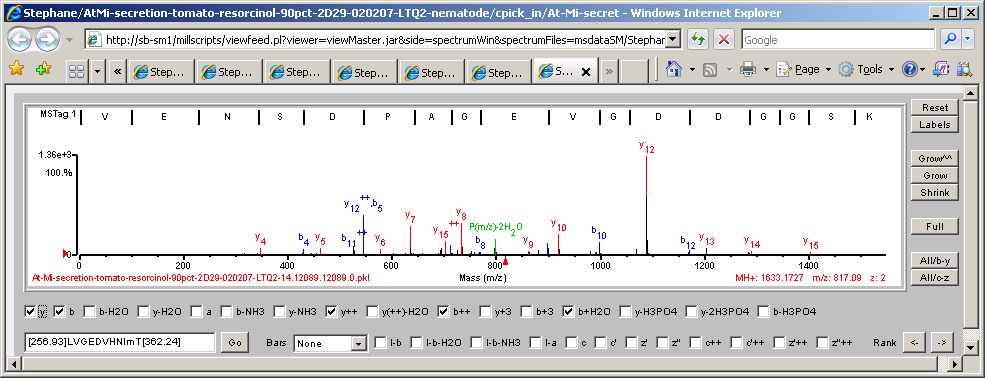 |
| 284 | *M. incognita* | CL1575Contig1_1_AA | (K)G P I/L/D|I|H|P/T/T I|H Q\R(T) | 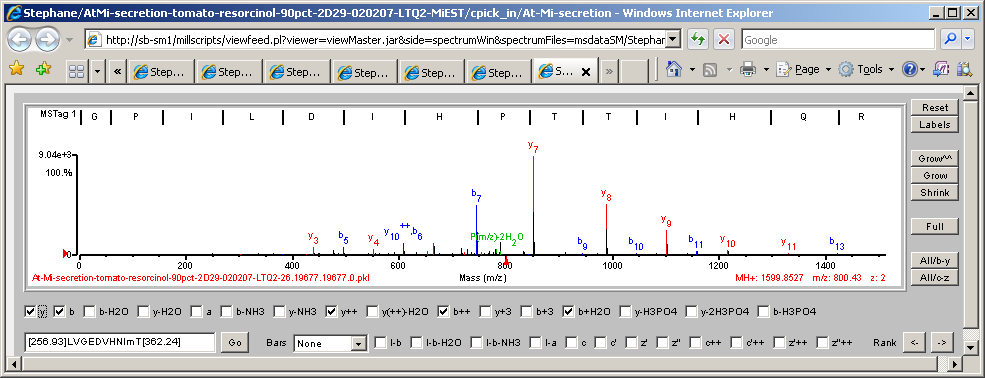 |
| 285 | *M. incognita* | CL2965Contig1_1_AA | (R)I/L|G|L|V|L|L|R(G) | 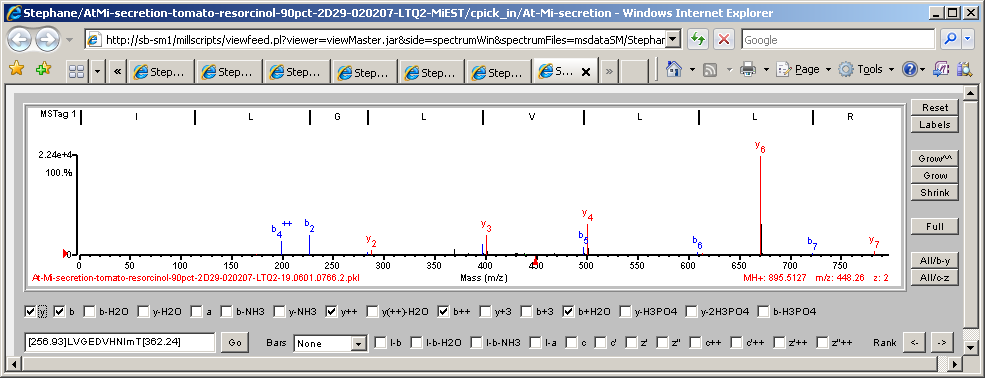 |
| 286 | *M. incognita* | CL1793Contig1_1_AA | (R)A I/E/Y/G/L|E|E|L|S|E\G K(M) | 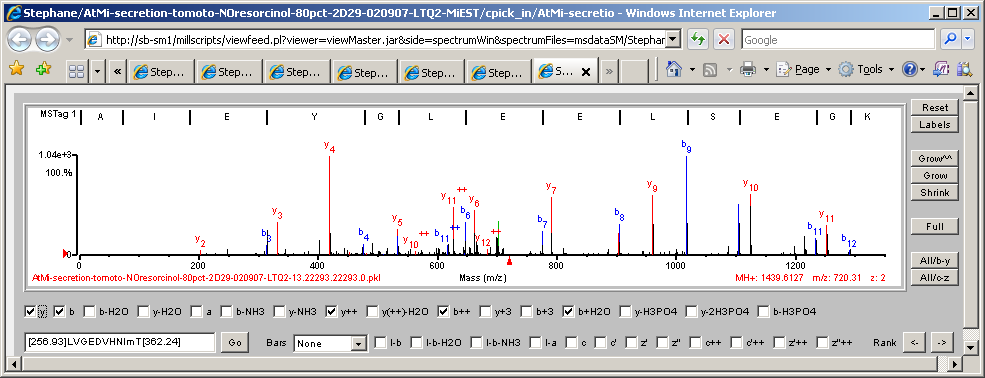 |
| 287 | *M. incognita* | CL26Contig3_1_AA | (K)S E/D|L/N/D|D\E/Q/I|V|N E Q K(A) | 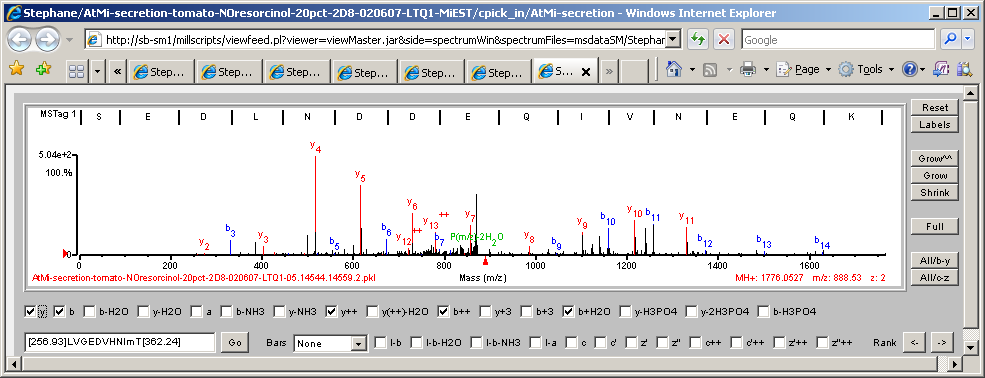 |
| 288 | *M. incognita* | 211k05r1.1_1_AA | (R)A/V/V/Q/A|G|A|V|P H\F\L\K(L) | 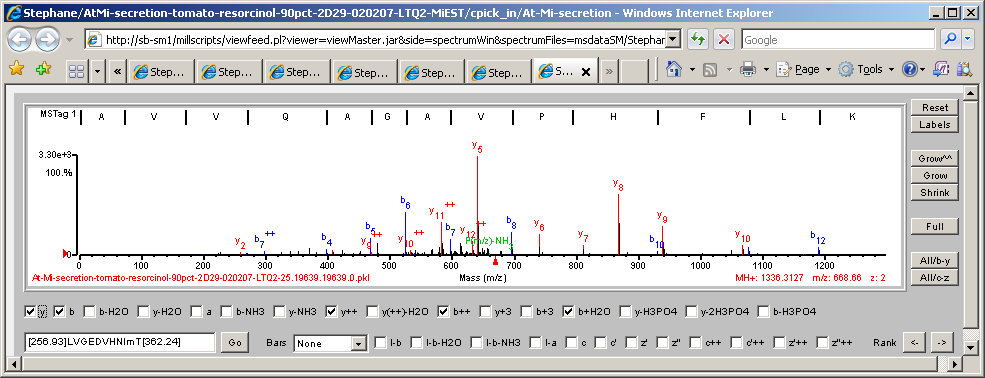 |
| 289 | *M. incognita* | CL280Contig1_1_AA | (R)A L/G Y D/L/P M/V|D/E/G/Q|P E|P E F Q R(I) | 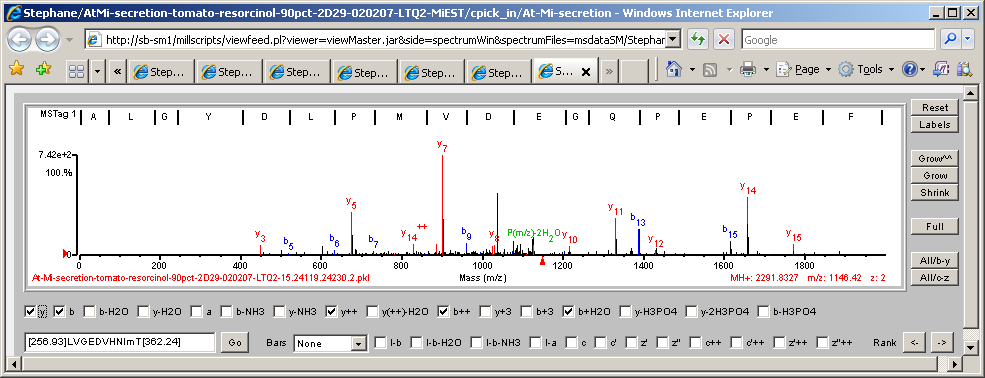 |
| 290 | *M. incognita* | CL2Contig10_1_AA | (R)G E/P L/S|E/D/E/V|Q|S/M|Y K(G) | 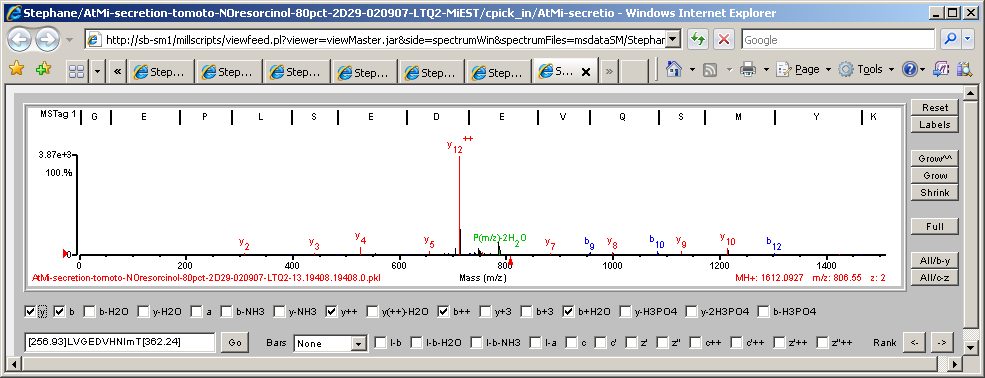 |
| 291 | *M. incognita* | 221l04r1.1_1_AA | (R)A V/D/A N/G A/E|S|G/N|S N Q/V|V\V\K(T) | 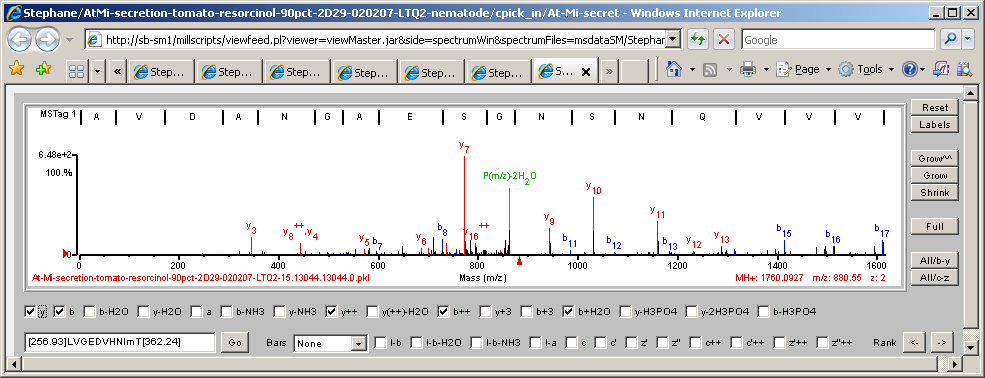 |
| 292 | *M. incognita* | 223a18c1.1_1_AA | (K)V/I/F/E/G N E|I|T/G S/V|L|D|G E E E E K(Y) | 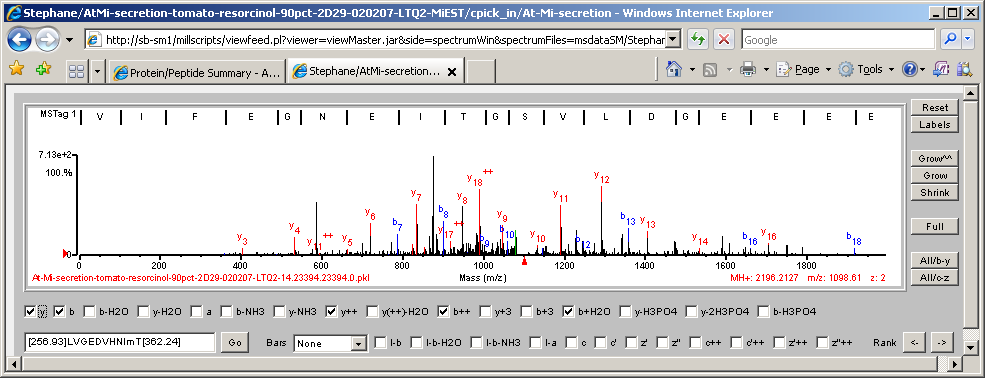 |
| 293 | *M. incognita* | CL2931Contig1_1_AA | (R)I/Q/F/S/H|I\D/S D|V\I\A\K(A) | 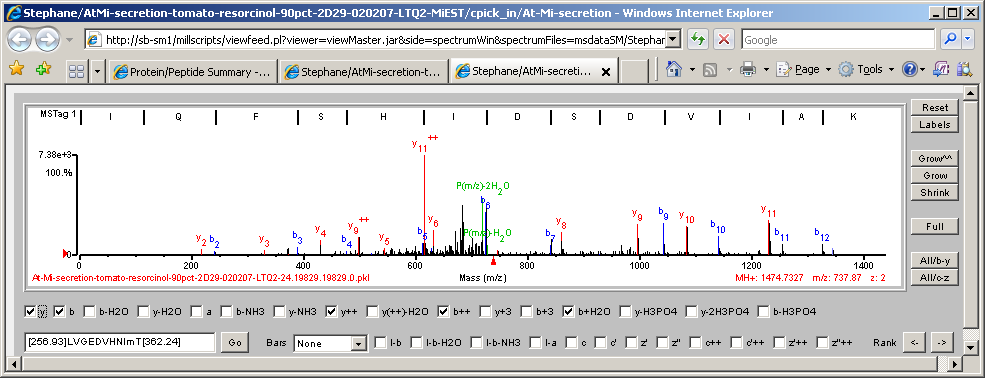 |
| 294 | *M. incognita* | CL6Contig6_1_AA | (R)D Y V/A|P A N N Y E M L G S\L|P V S A/V|V|V|D|S\P Y K(L) | 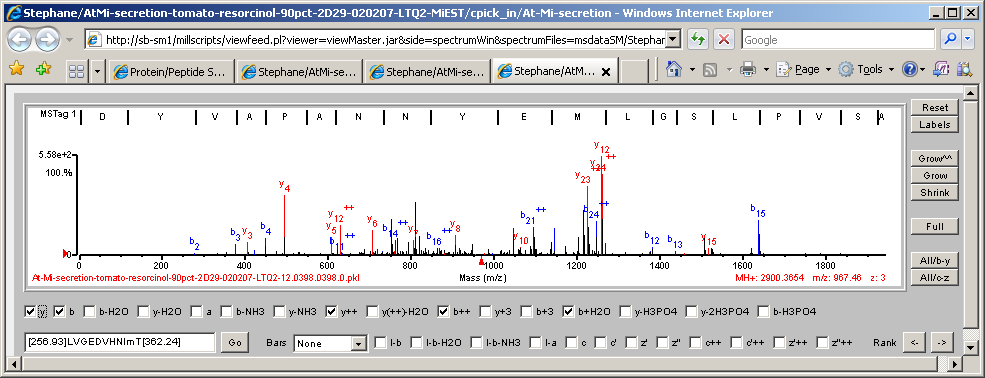 |
| 295 | *M. incognita* | 208a01r1.1_1_AA | (K)T I/E/A E|P D S S|T/G P V|V|D|F Q\D\P R(L) | 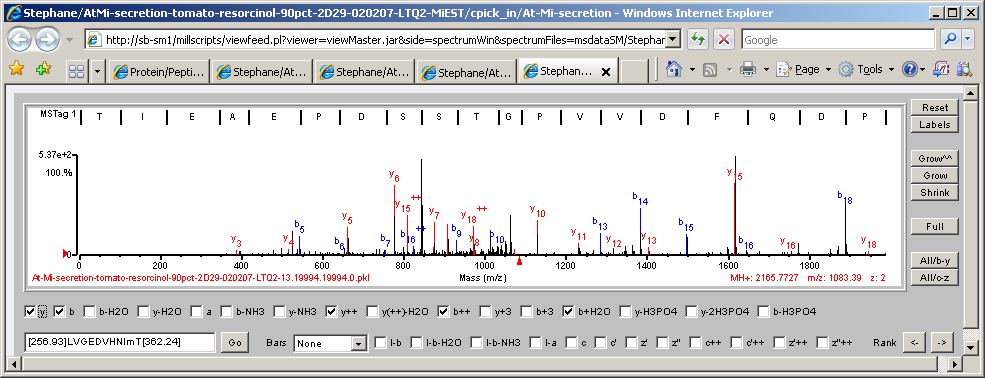 |
| 296 | *M. incognita* | 223a18r1.1_1_AA | (R)L A/A/L/T/Q/D/I|E|N I|T|A|S L K(D) | 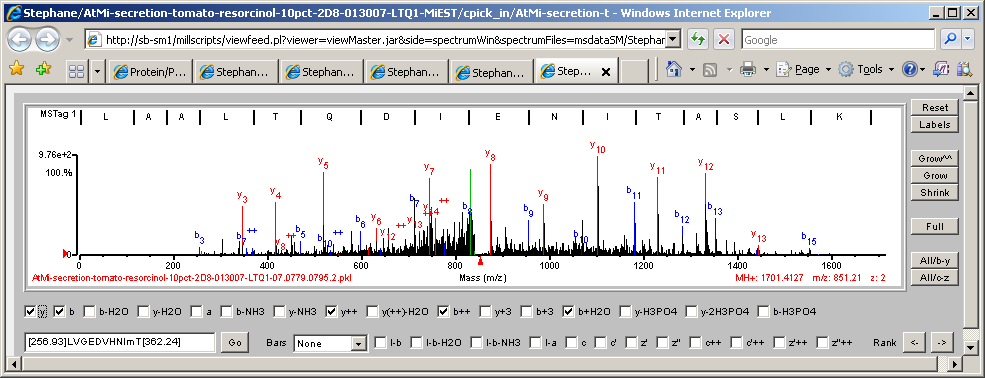 |
| 297 | *M. incognita* | 215o03c1.1_1_AA | (R)T Q/F/N|K|P I|A G|T Q/L V\Q L\K(L) | 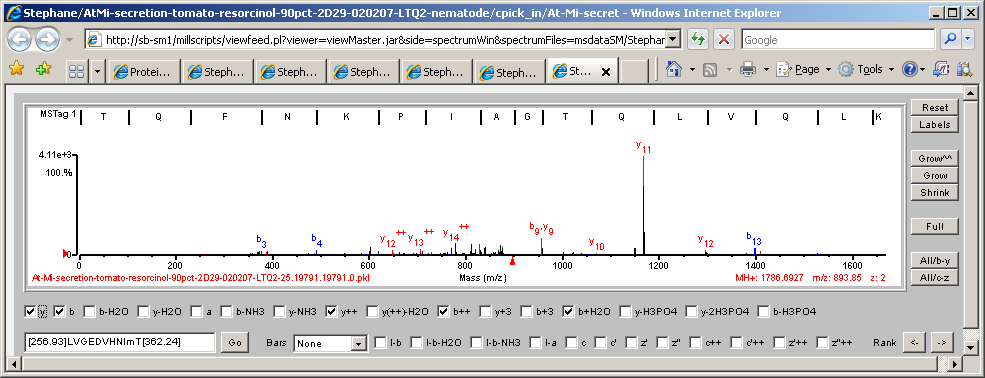 |
| 298 | *M. incognita* | CL2121Contig1_1_AA | (R)S G/T/P D E/L/M/D|L/Y/G I|S A K(K) | 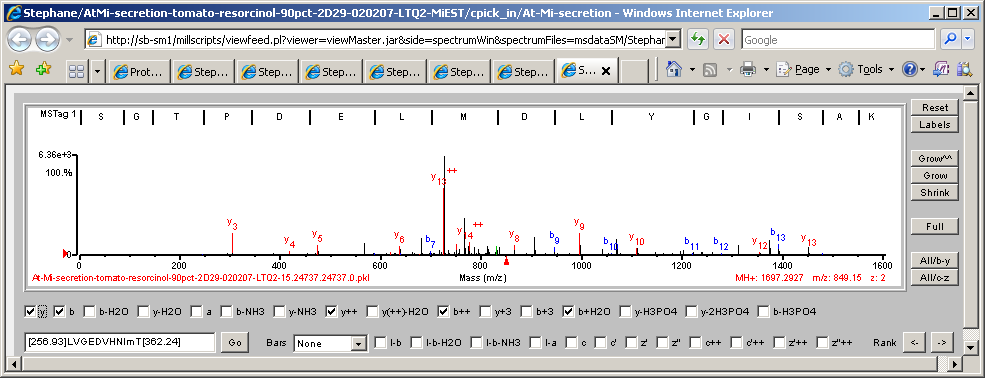 |
| 299 | *M. incognita* | CL2507Contig1_1_AA | (K)E V/Q/P S T/A|T|S/V|I|P A/D D L T R(A) | 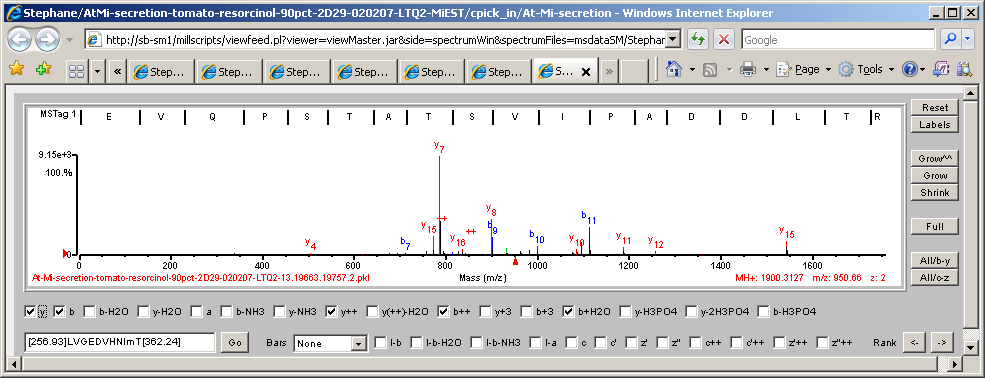 |
| 300 | *M. incognita* | CL2771Contig1_1_AA | (R)I D|I|P/A|G/T|V|L|E|N K(I) | 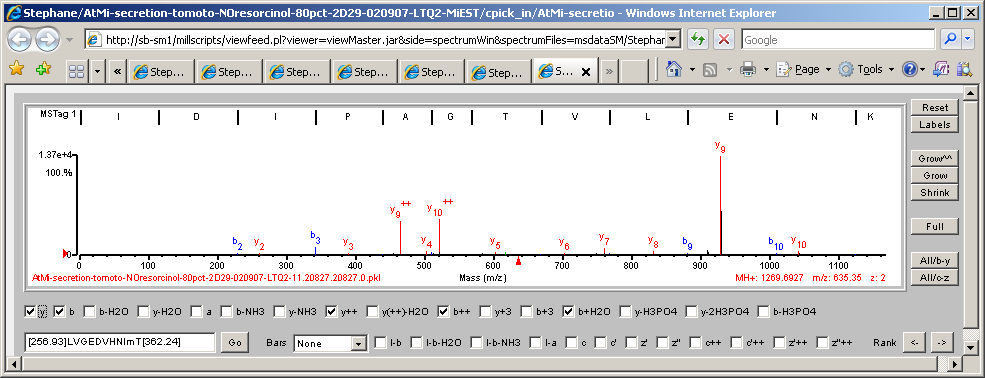 |
| 301 | *M. incognita* | 203i19c1.1_1_AA | (R)I I/A|V|D/Q|D P L|G/I|F G R(M) | 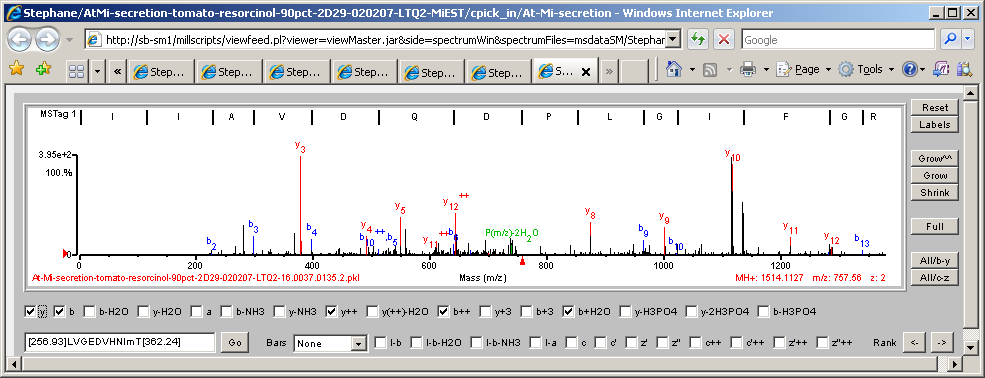 |
| 302 | *M. incognita* | 204p08r1.1_1_AA | (K)I Q/Y/E|P L/N|D/D I/D/D/D/L S\Y\I K(V) | 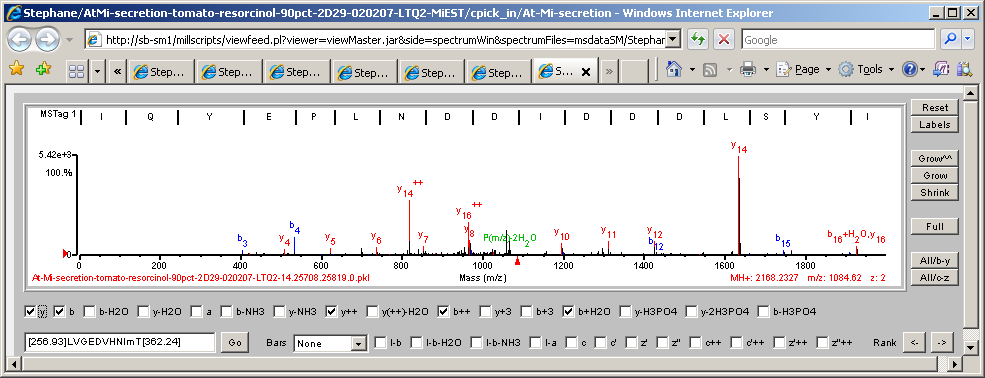 |
| 303 | *M. incognita* | CL651Contig1_1_AA | (K)V N/A/H/G/G A|V|S|L|G/H|P I|G M S\G A R(I) | 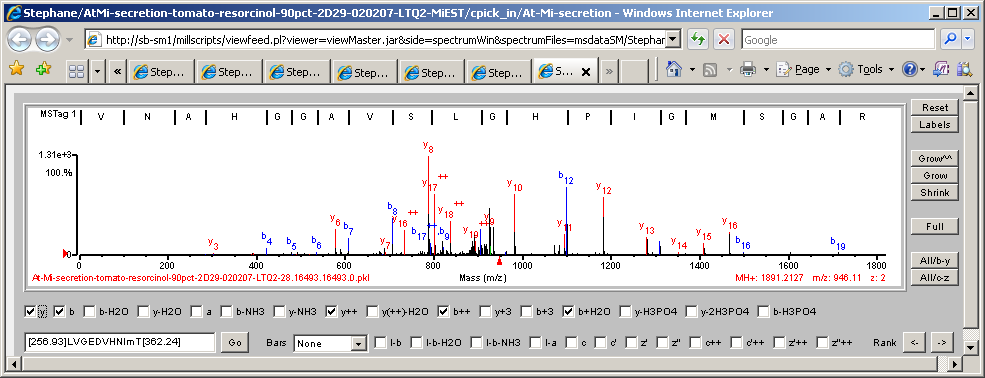 |
| 304 | *M. incognita* | 206p09r1.1_1_AA | (K)E L/C N S/Y/T|E/L|N|D|P I|T Q R(E) | 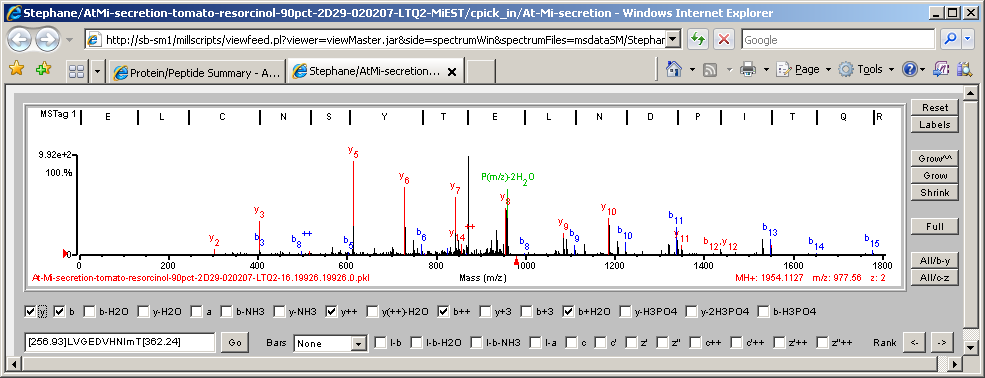 |
| 305 | *M. incognita* | CL1031Contig1_1_AA | (R)D/S/G/S/G/G/S/N Q/V L|L|I|T K(E) | 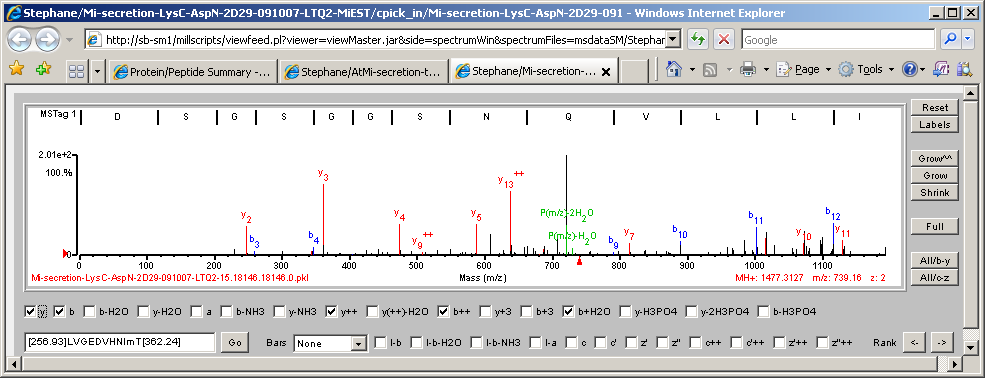 |
| 306 | *M. incognita* | CL1Contig15_1_AA | (R)N/P/E/S/G/V|A|T/T/D/L/Y G R(C) | 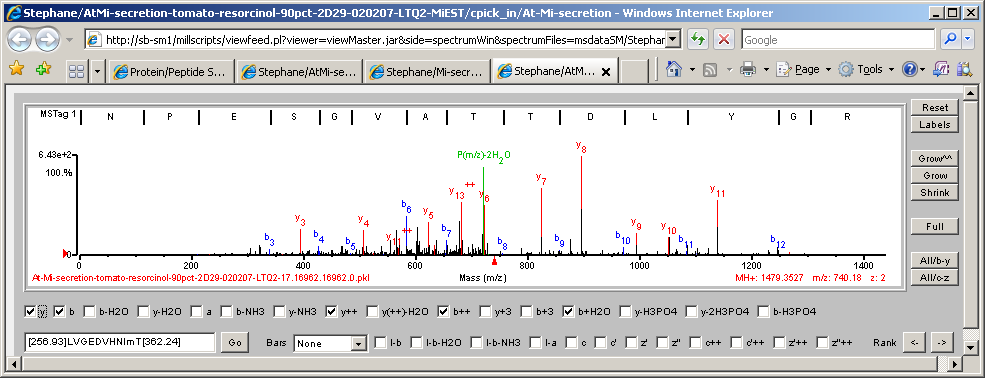 |
| 307 | *M. incognita* | CL2418Contig1_1_AA | (K)M A/E/Y/A|D|Q/L|N|E|D E V\K(V) | 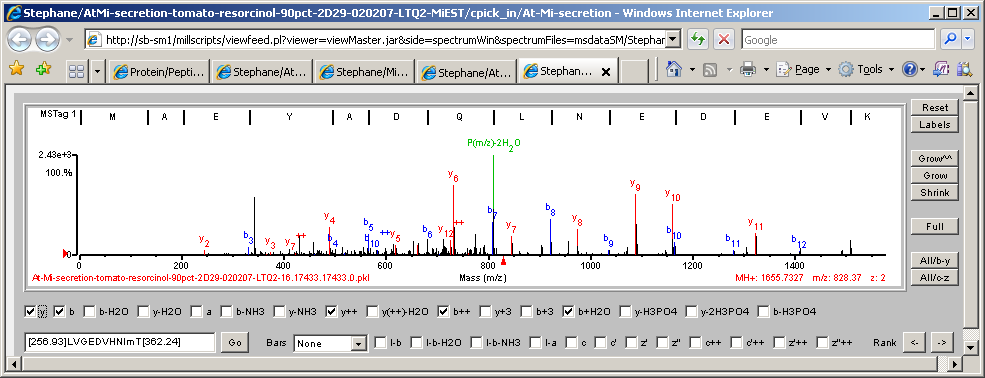 |
| 308 | *M. incognita* | CL902Contig1_1_AA | (R)F/V/H/G/N E|P G S|L|P T A/I|Y T H R(I) | 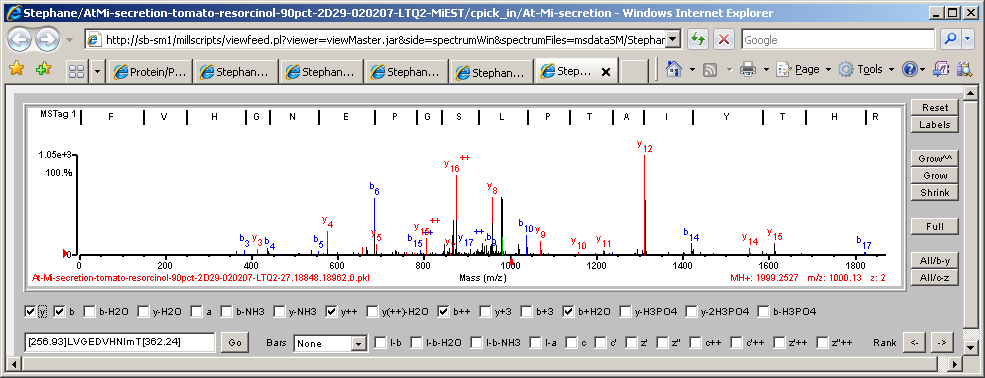 |
| 309 | *M. incognita* | CL135Contig1_1_AA | (K)Y/V/N\I|P T A|Y|A|A|F|P Y D/I G G A T\P K(E) | 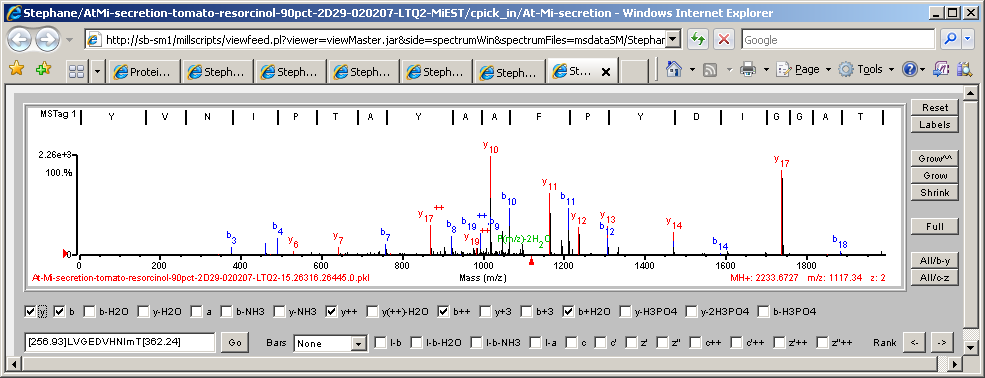 |
| 310 | *M. incognita* | CL155Contig2_1_AA | (K)T H/I|F/C\Q|Y|S|P|P G N/Y\M\G Q\K(I) | 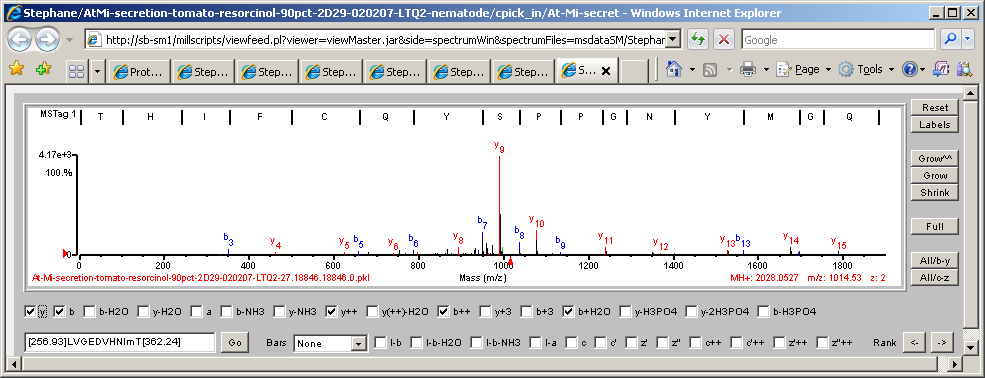 |
| 311 | *M. incognita* | CL2550Contig1_1_AA | (K)M/V/H/S/N/I|S|P N S V|I\I\N\K(K) | 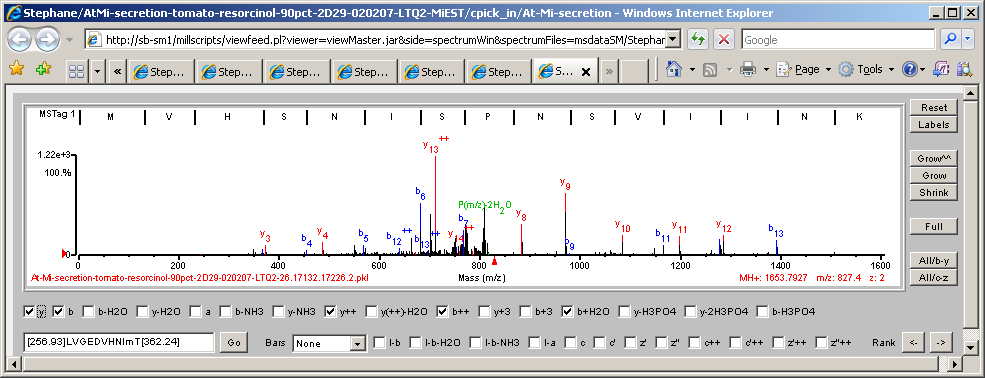 |
| 312 | *M. incognita* | CL3006Contig1_1_AA | (K)N G A/Y/D/Y|P T|P E/W D/T V|T|P E A K(A) | 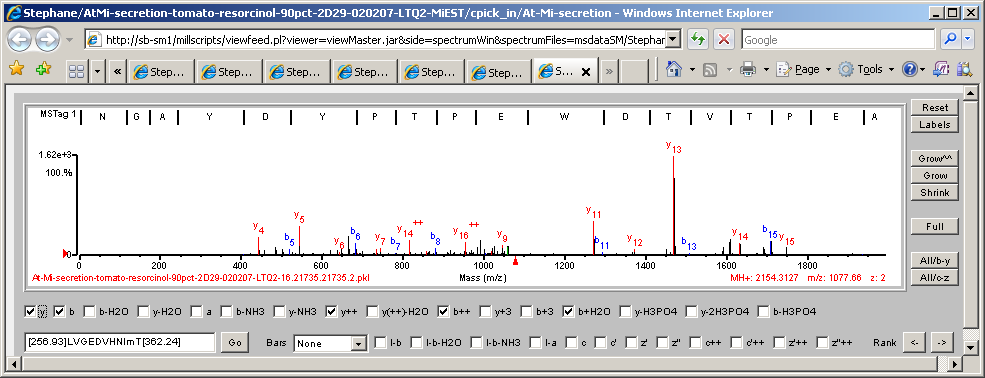 |
| 313 | *M. incognita* | 218g22r1.1_1_AA | (K)D L/L E/Q/G L|S|F/D/N/D|P T L\I\K(K) | 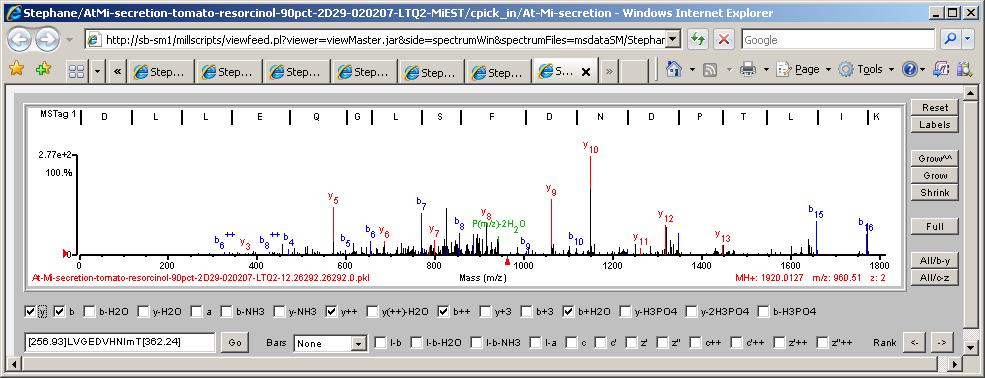 |
| 314 | *M. incognita* | CL1220Contig1_1_AA | (R)I S D S E/W|S/A|E|F|P L|D/S A G S S G R(I) | 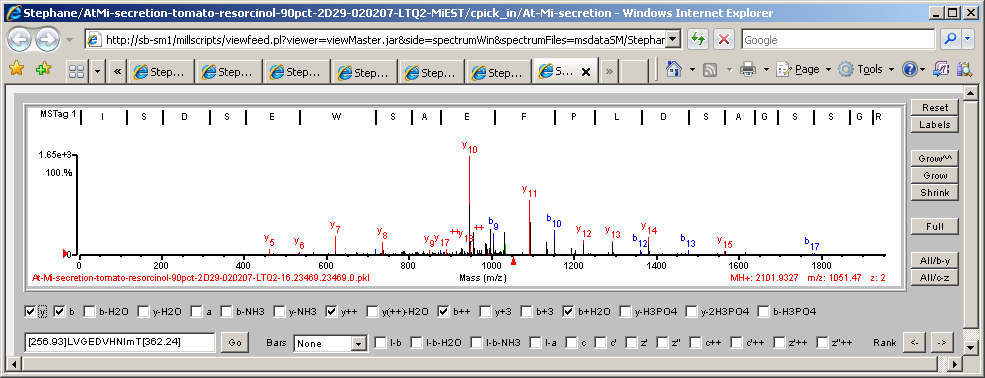 |
| 315 | *M. incognita* | 211k16c1.1_1_AA | (K)A/I V D T I/I|A|I\V|E|D/N/S N A K(D) | 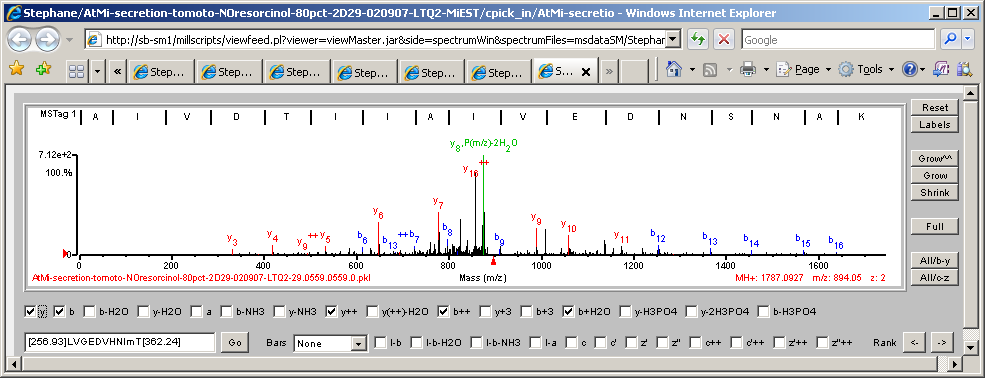 |
| 316 | *M. incognita* | 225m13c1.1_1_AA | (R)D A/A|S|Q|S/L|S|G/I|S\K(R) | 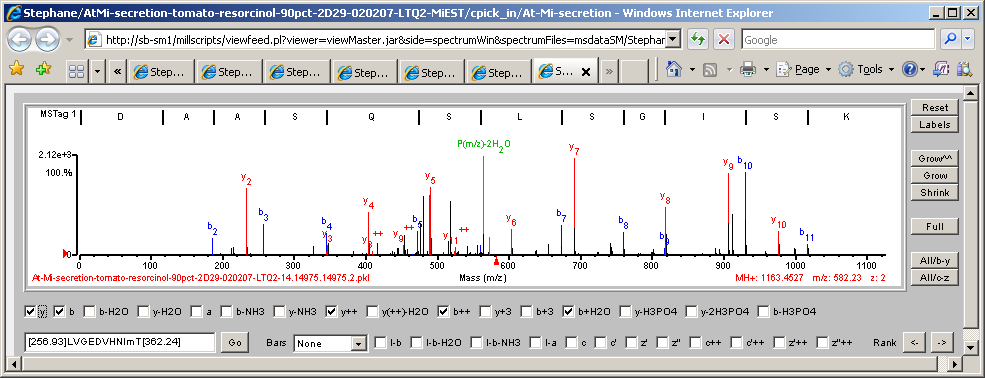 |
| 317 | *M. incognita* | CL1865Contig1_1_AA | (R)L Y N A D D\L/N/T|L|L|S/E|S D A I|A Q K(R) | 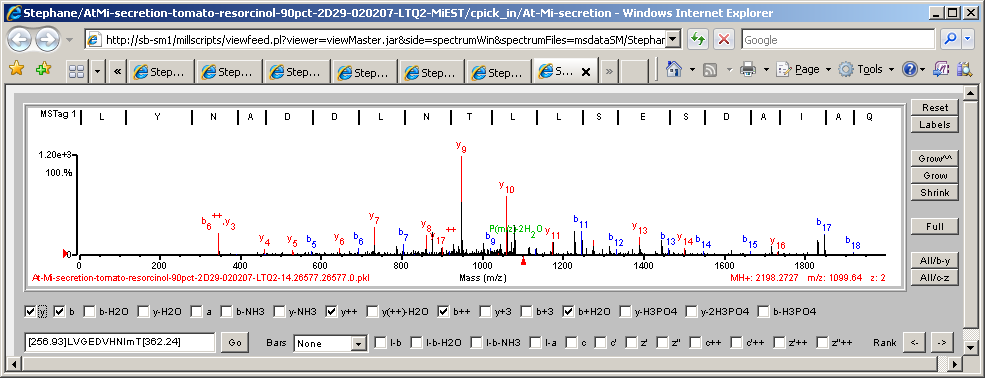 |
| 318 | *M. incognita* | CL1101Contig1_1_AA | (R)Y T|D/E|Q|V|D|E/L|F\R(D) | 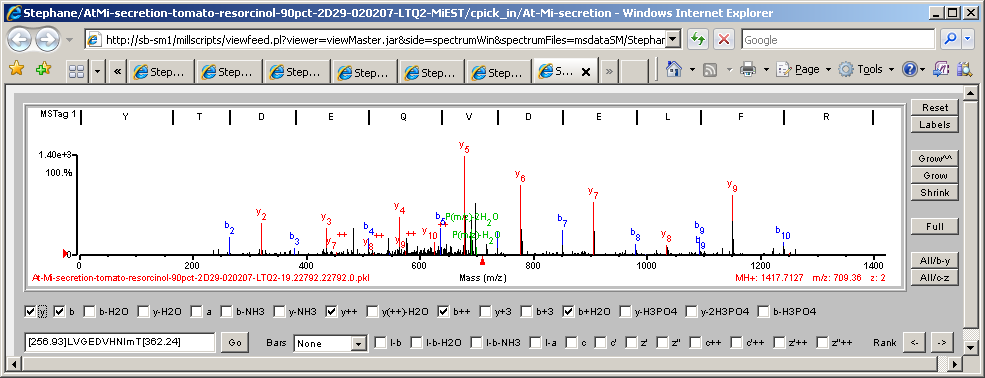 |
| 319 | *M. incognita* | CL384Contig1_1_AA | (K)N V/E/G E/V/E/N E/T|P P H|P T K(S) | 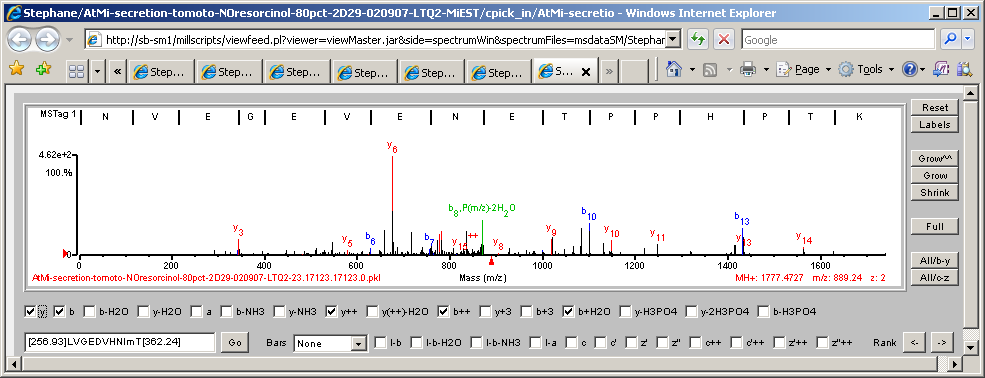 |
| 320 | *M. incognita* | CL2552Contig1_1_AA | (R)I V/D/I|D|T|G|P D P D/D/T L D\E K(F) | 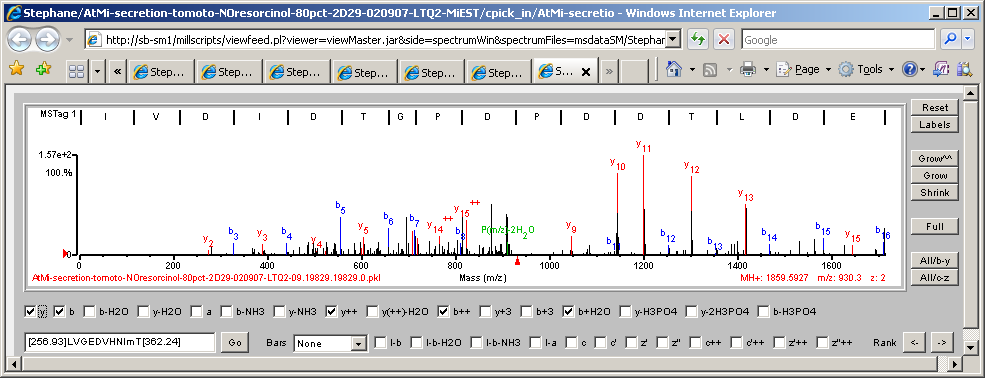 |
| 321 | *M. incognita* | CL600Contig1_1_AA | (K)F/T N S A/I|K|F/F/T/T/I|L\N\K(Y) | 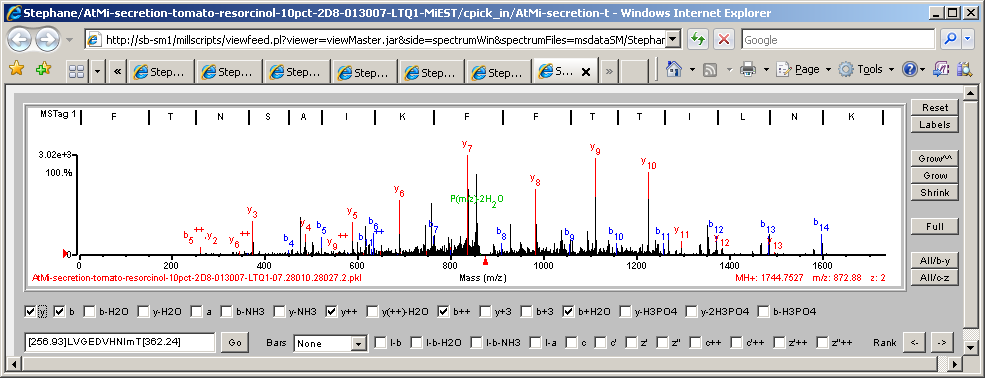 |
| 322 | *M. incognita* | CL7Contig2_1_AA | (K)Y S D V/A/D/A|Y|G G|V|D|P E\T V\G R(S) | 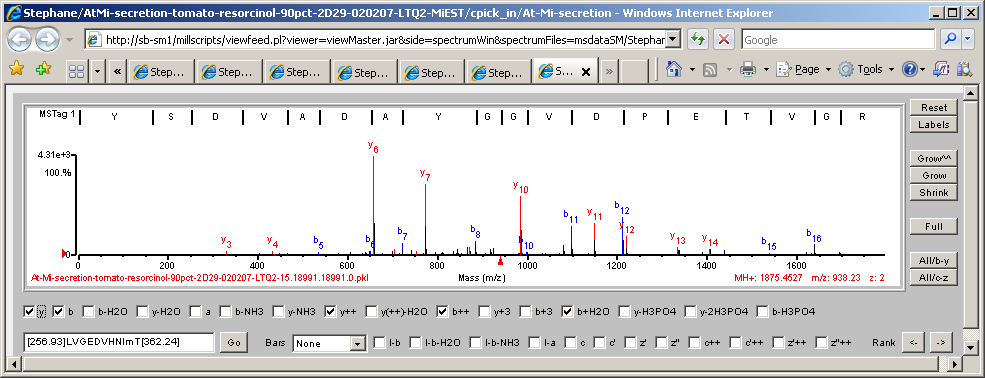 |
| 323 | *M. incognita* | 202c23r1.1_1_AA | (R)T I/A|F|G/P N/Y|G/G C E R(S) | 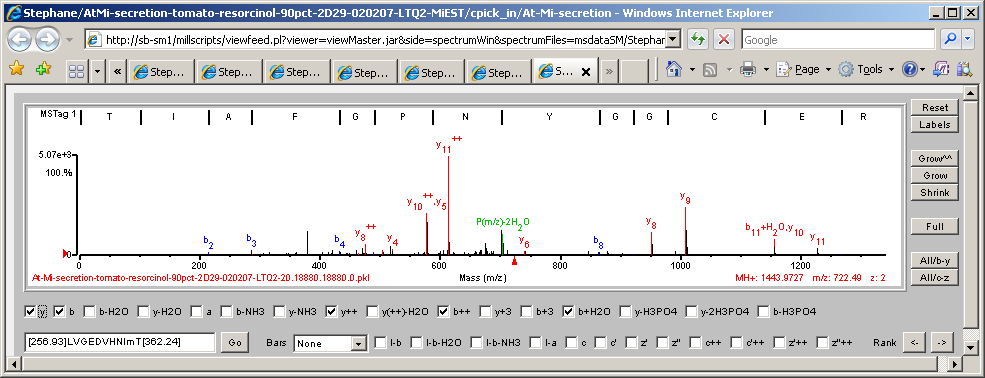 |
| 324 | *M. incognita* | 210e11c1.1_1_AA | (R)V/G/P D/M/A|V|P I D|E|Y D G K(V) | 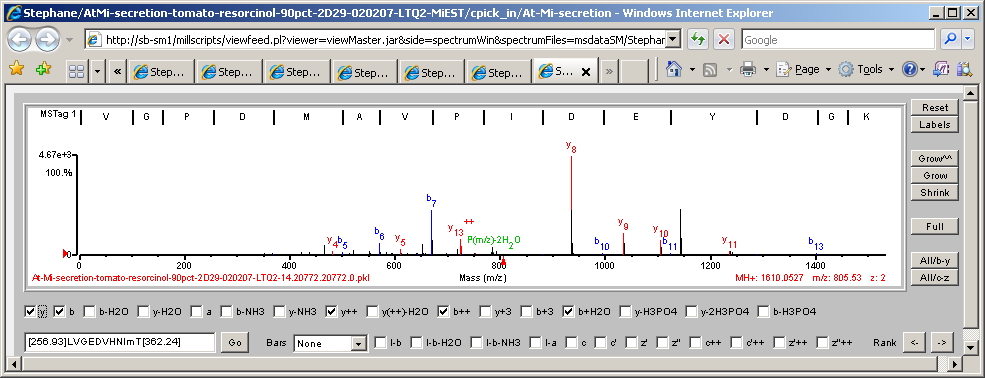 |
| 325 | *M. incognita* | CL2363Contig1_1_AA | (R)I V/A/S|P/D/E|P N A/F/E/C A I\G R(S) | 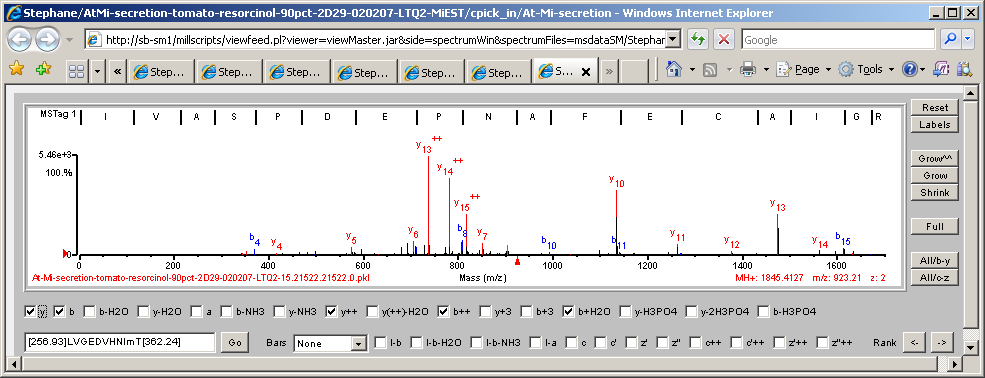 |
| 326 | *M. incognita* | 203n01r1.1_1_AA | (R)D/T/H L/V|F|A|M|P\Y D|T/P V|P G F R(N) | 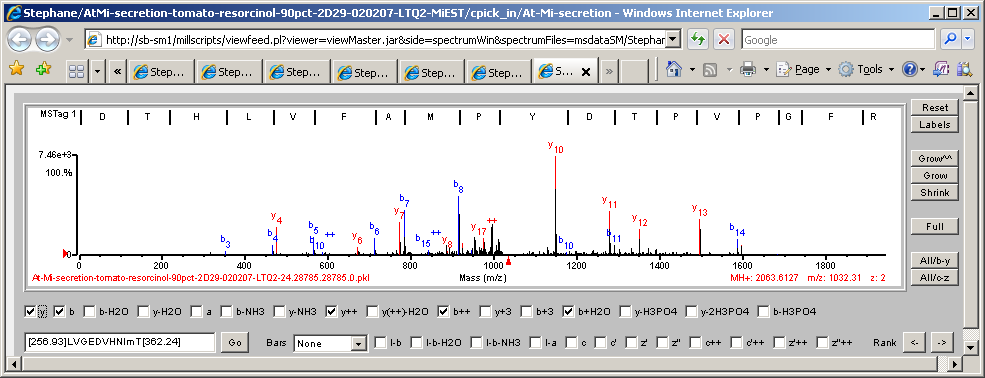 |
| 327 | *M. incognita* | CL220Contig1_1_AA | (R)T I/A/F/D/F/T|N|P N L/E|D Y N K(Y) | 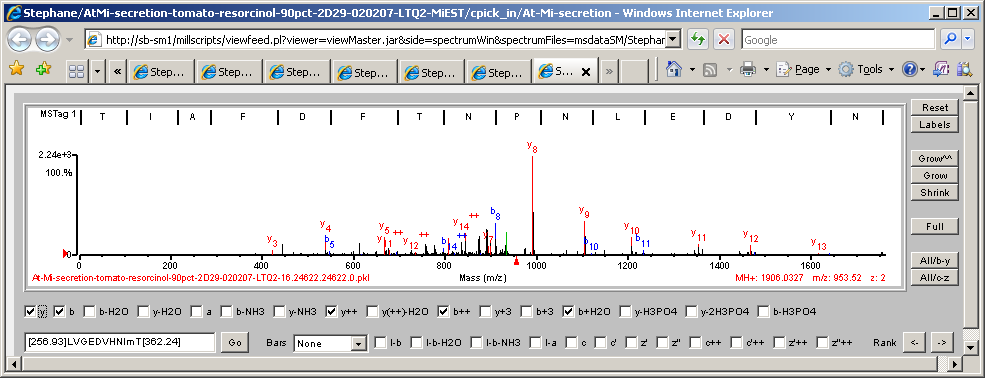 |
| 328 | *M. incognita* | CL1345Contig1_1_AA | (R)R D|Y L\H|F|V|P K(Y) | 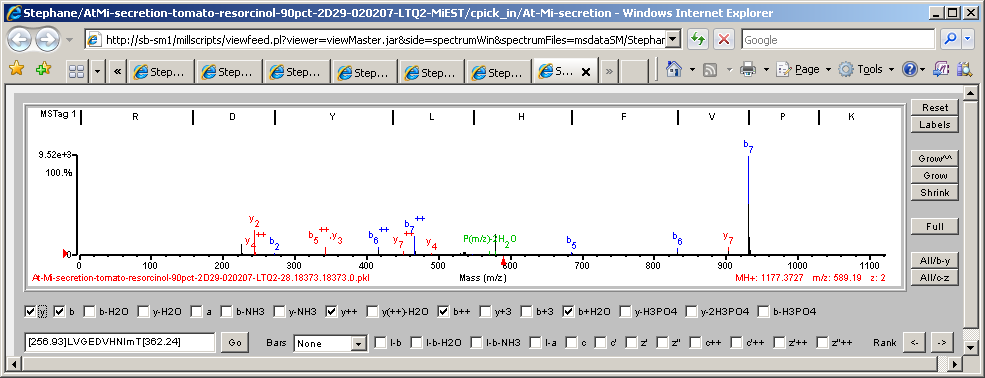 |
| 329 | *M. incognita* | 226d21r1.1_1_AA | (R)L D/D/T|N|P/E/A|E|D|E R(Y) | 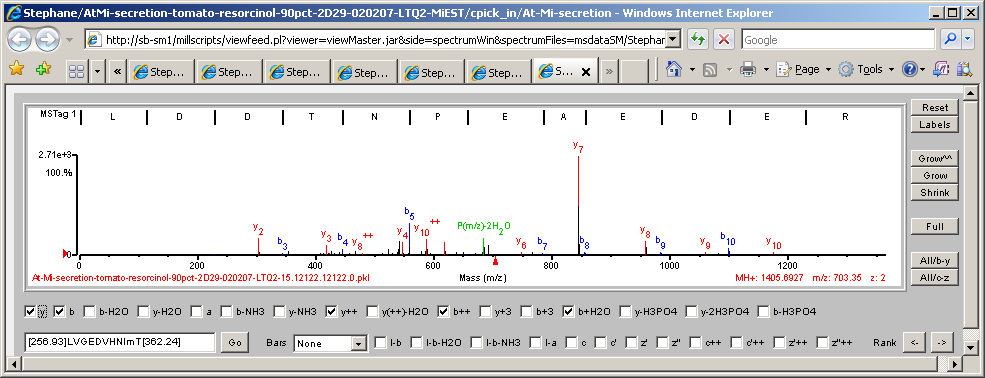 |
| 330 | *M. incognita* | 214m01c1.1_1_AA | (R)M/S/V E/H|A|T/E|S Q Q|P S R(S) | 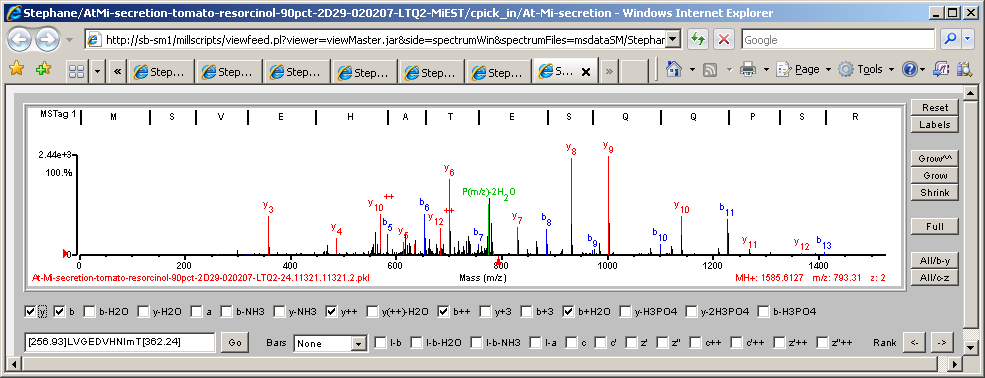 |
| 331 | *M. incognita* | 217d04r1.1_1_AA | (K)V S/L/D|P N T\Q|W|S|I|P R(W) | 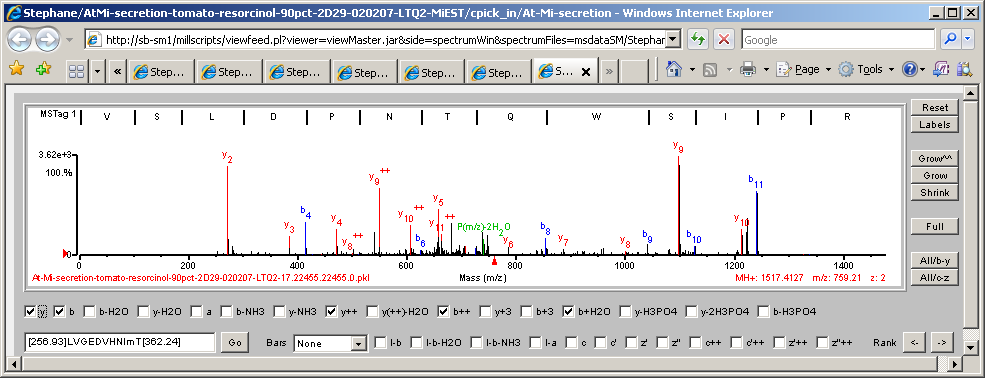 |
| 332 | *M. incognita* | CL2549Contig1_1_AA | (R)S L M T N A/N|P/Q/L/F|Q/A|M A/N\P R(V) | 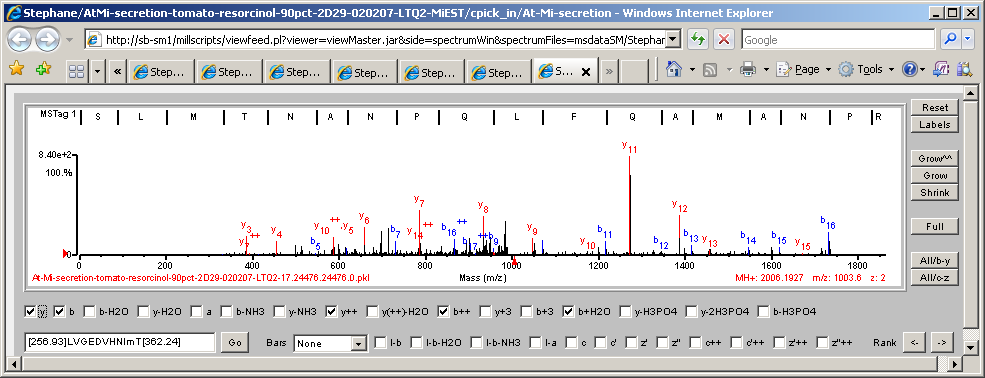 |
| 333 | *M. incognita* | CL279Contig1_1_AA | (R)L L/Y/E/N/I|P T V V|F S H P P|L G T T\G\L T E R(E) | 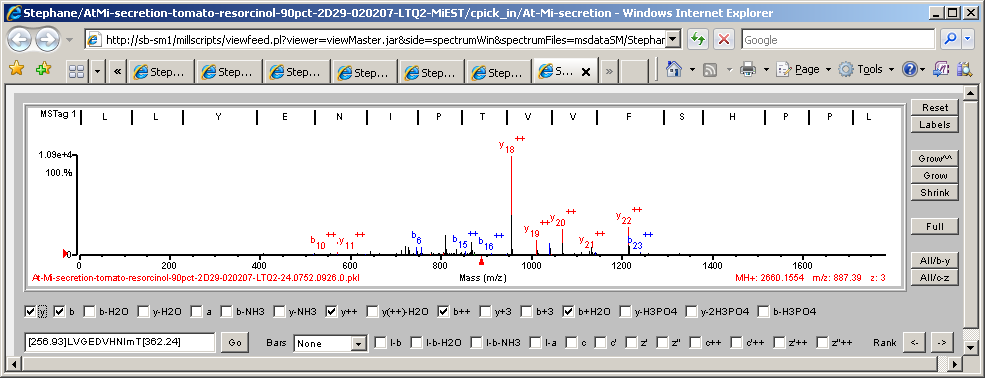 |
| 334 | *M. incognita* | CL2746Contig1_1_AA | (K)N D/S/S N D|T/S T/S/N N N V|E/H\V|E\N V\K(F) | 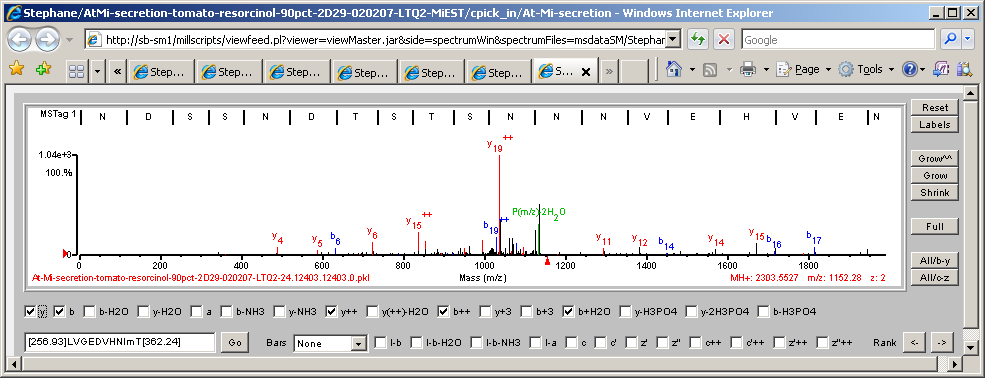 |
| 335 | *M. incognita* | CL2942Contig1_1_AA | (K)K/L E/G Q/E|E/E\E/D|V|V|E\E\E\K(E) | 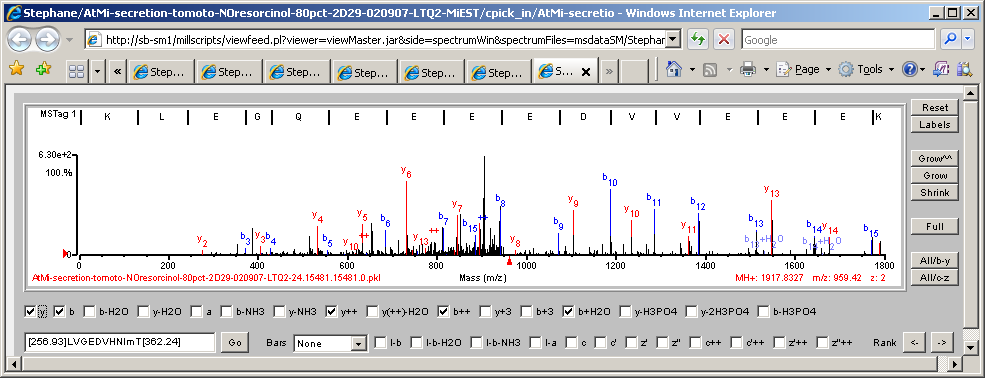 |
| 336 | *M. incognita* | CL2370Contig1_1_AA | (R)G/P G D|S/S/N/F/D|D|Y|E|E|E|P L\R(I) | 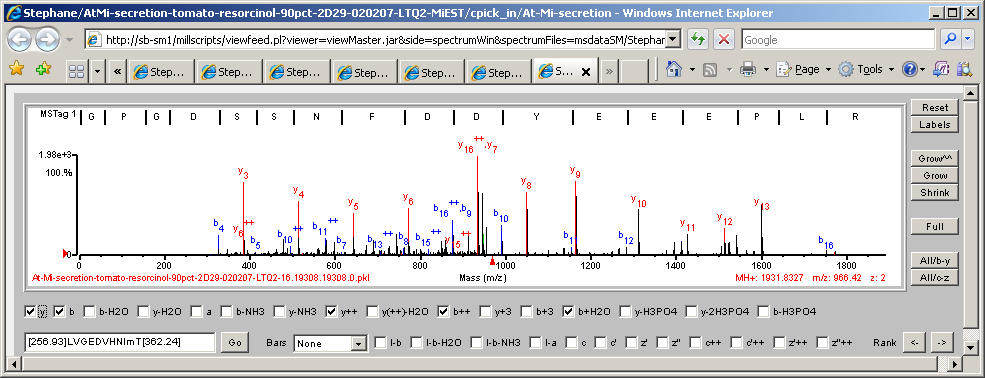 |
| 337 | *M. incognita* | 215a24r1.1_1_AA | (R)I G/A G D/E/A|A/D|D|G F|T/L|P Q R(G) | 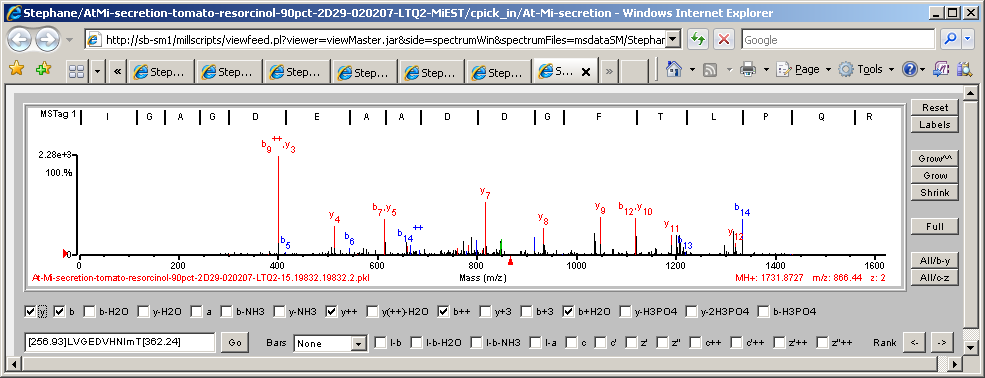 |
| 338 | *M. incognita* | CL358Contig1_1_AA | (R)A S/G/P E/V/D/D/E F/L|S|S|P I Q K(Q) | 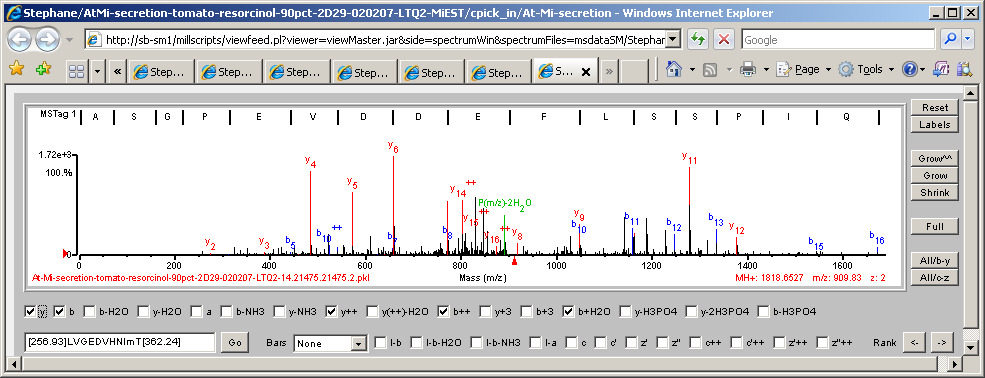 |
| 339 | *M. incognita* | CL1149Contig1_1_AA | (K)V E N L|I|L|E/E|E|N|N A\I\R(M) | 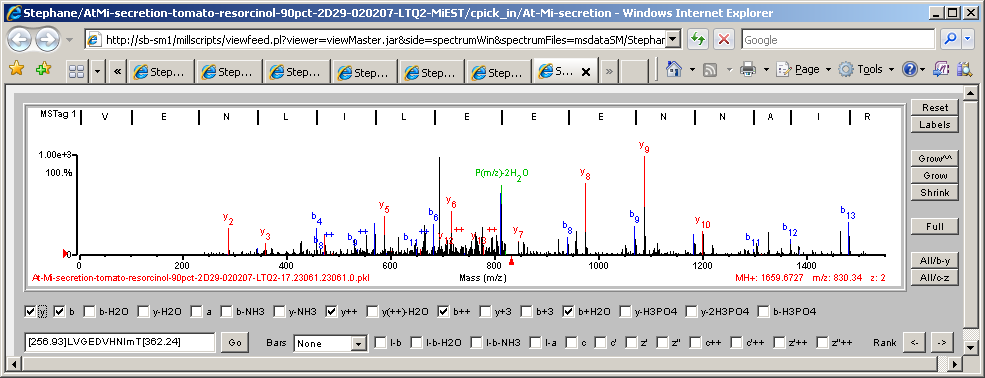 |
| 340 | *M. incognita* | CL2360Contig1_1_AA | (K)S F|T|P/I|I|T|A/P N K(N) | 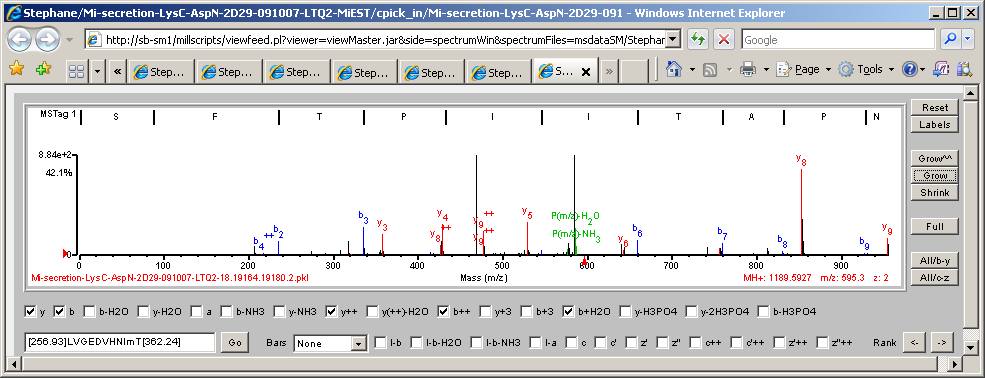 |
| 341 | *M. incognita* | CL99Contig1_1_AA | (K)E V/T/N E N/I|D E|P E/D/K G D\K(M) | 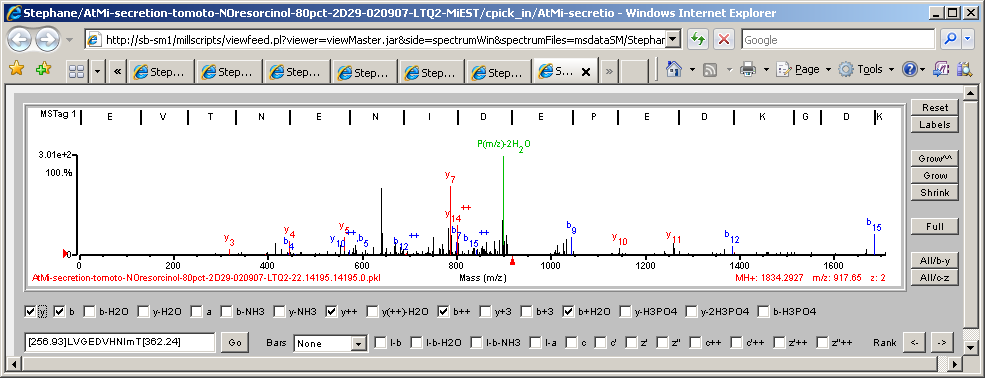 |
| 342 | *M. incognita* | 209l14r1.1_1_AA | (R)N L/A|V/Y/Q/G H/N|K|P L R(M) | 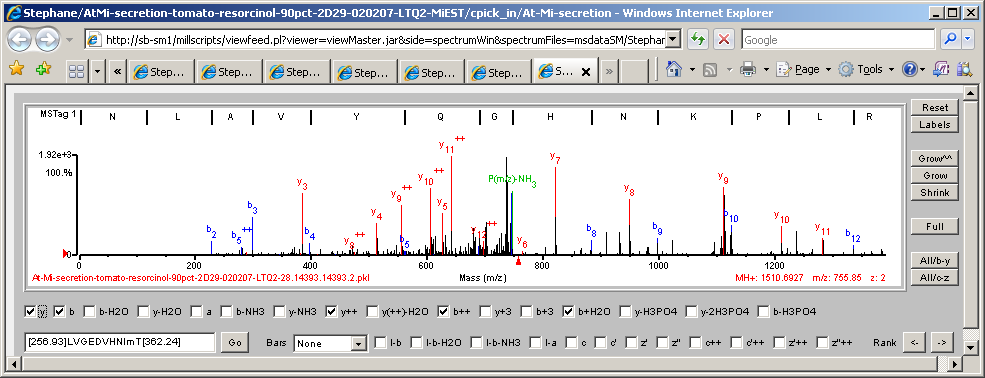 |
| 343 | *M. incognita* | MI00879 | (K)S Y/G D/F/E|S/D|E G|Y|P F L|A\I\S K(D) | 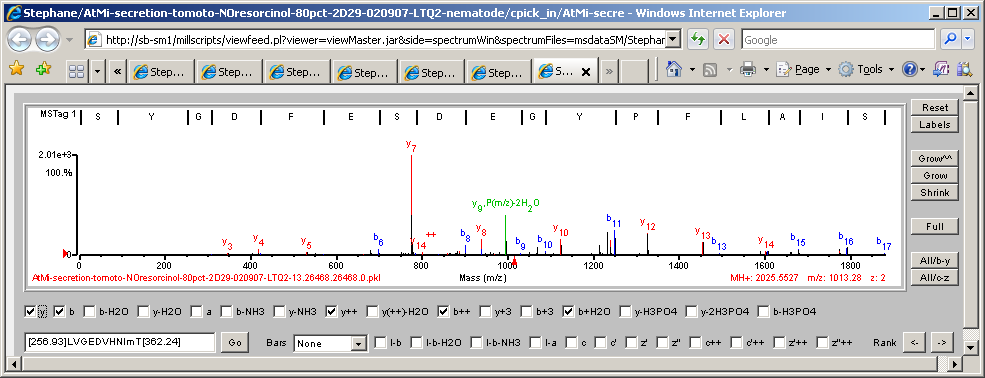 |
| 344 | *M. incognita* | MI02052 | (K)N D/Q/E|P Q|P S|N/N L/E|Q|P E K(L) | 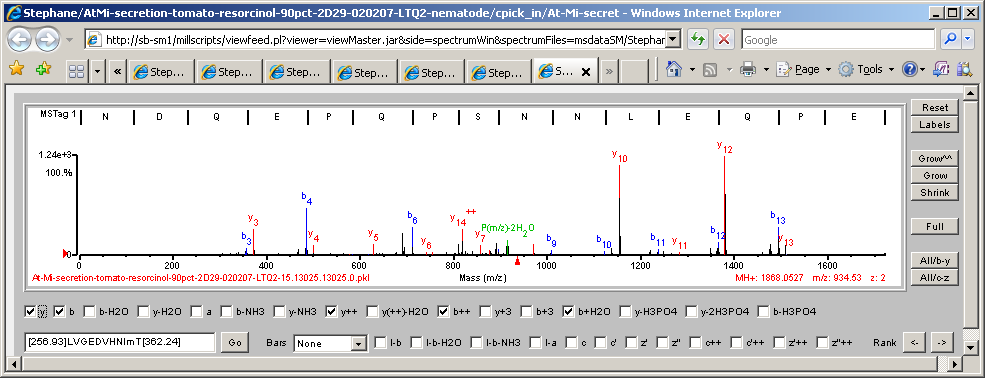 |
| 345 | *M. incognita* | MI02465 | (K)G F/G F C/E/F|Q|D|Q\Q|G A D|D A V\A\K(M) | 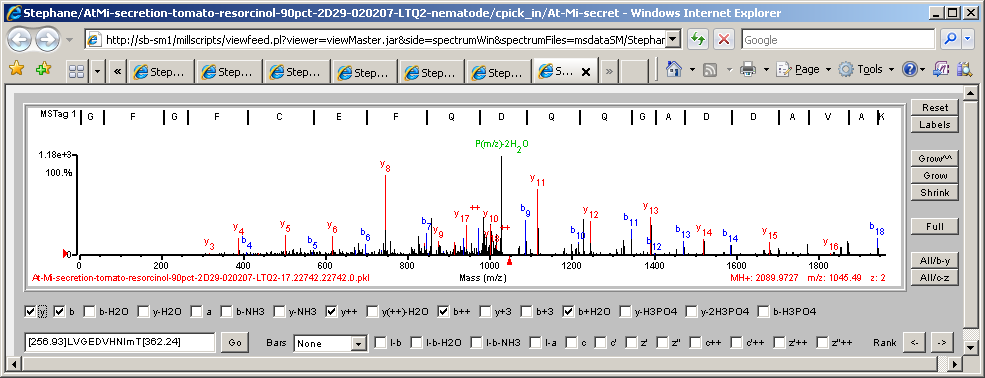 |
| 346 | *M. incognita* | MI07708 | (K)N S/V/G D/D D/L/I|D/E/D/A L/L|E\P K(D) | 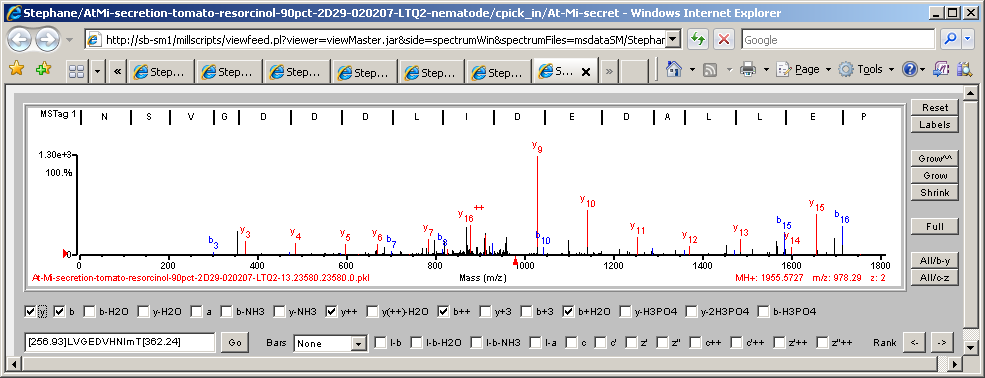 |
| 347 | *M. incognita* | MI01934 | (R)D A/T N/D/Q|V|T F/D/A A E|A I\K(N) | 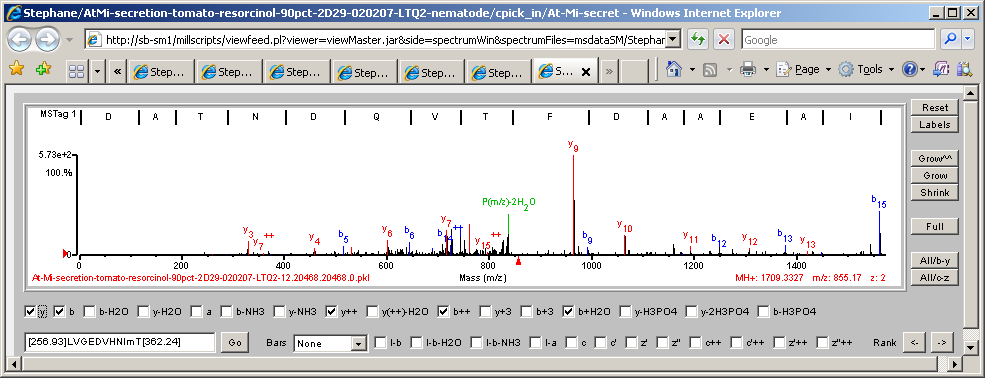 |
| 348 | *M. incognita* | MI04721 | (K)G G/Y/P T/N/F|I|E|E|P C T K(M) | 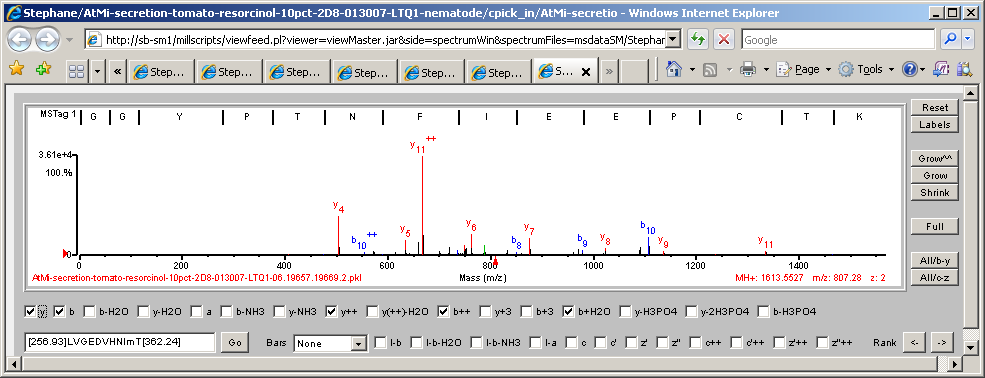 |
| 349 | *M. incognita* | MI07344 | (K)E S E G D D L/Y/D|A|A/I|E|P S/G P V N E K(R) | 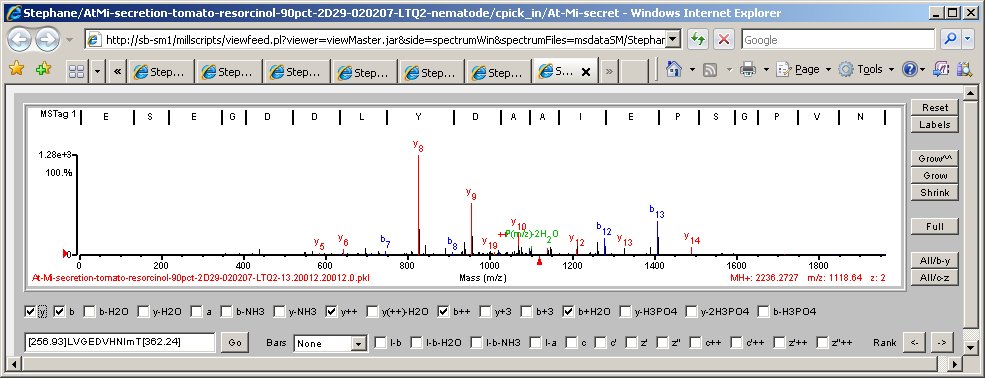 |
| 350 | *M. incognita* | MI01479 | (R)L A/H/E|V|G|W|Q|Y\Q|G I|V\E\K(L) | 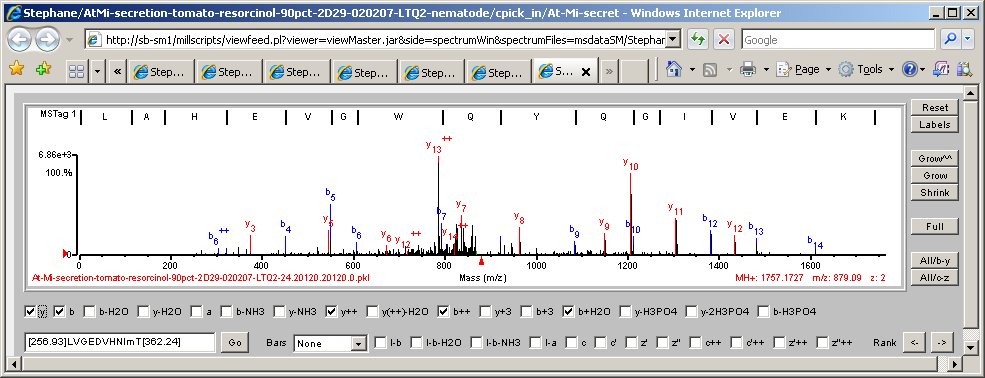 |
| 351 | *M. incognita* | MI02218 | (K)F/V/D/G V/M/I/H|S|G/N|P/V|T/E|Y/I Q E A V R(H) | 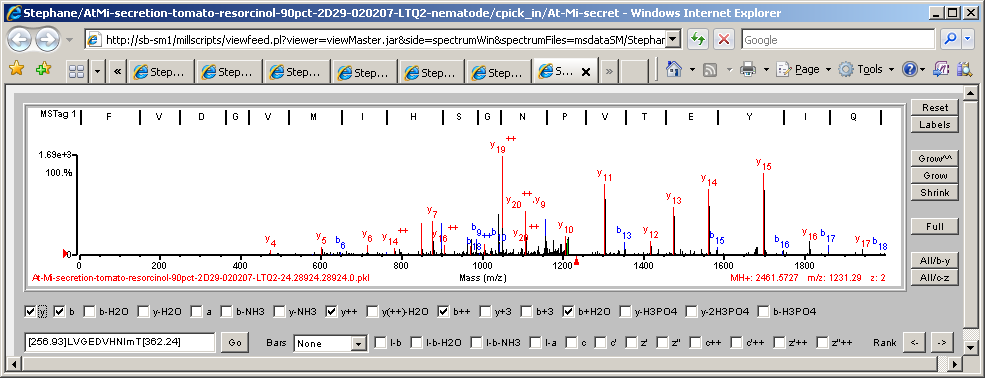 |
| 352 | *M. incognita* | MI00138 | (K)E F D A V|I|L|P|G/G/Q|P G S D/N L A K(D) | 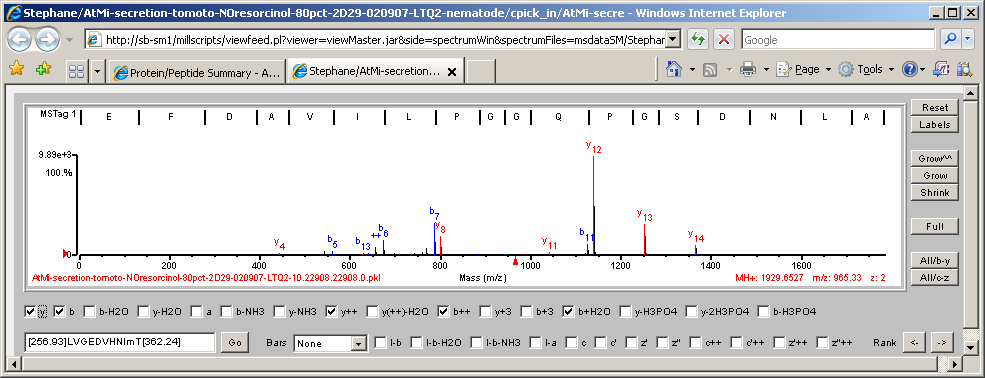 |
| 353 | *M. incognita* | MI00242 | (R)D S I/A G A/A/S/N/A|G Q|A/V|A|N/A|A|V\G A\K(D) | 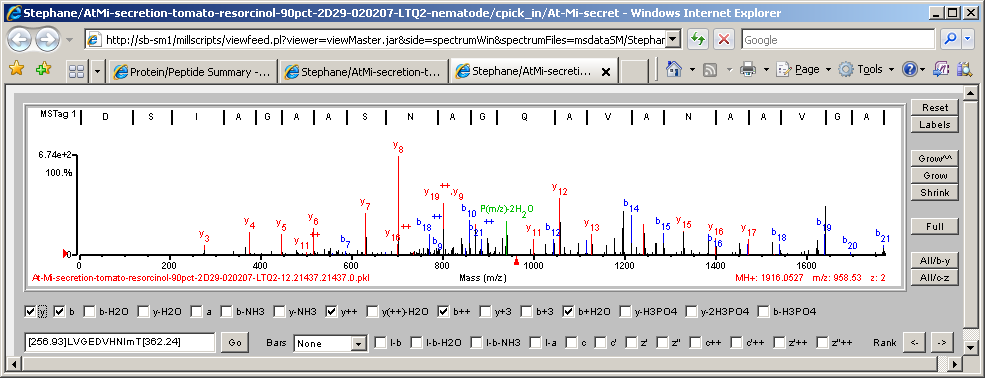 |
| 354 | *M. incognita* | MI01914 | (K)D Y|L/E|E|D|P S/D/T/D/S S R(F) | 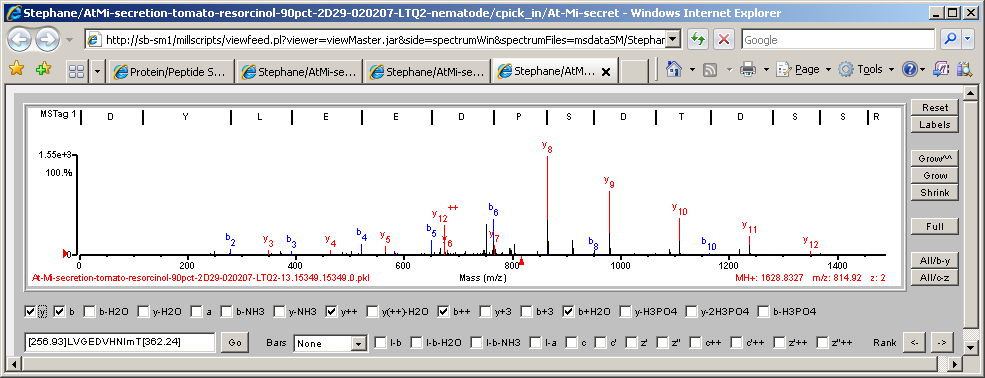 |
| 355 | *M. incognita* | MI01755 | (K)I/V/D|P S/E/L|S Q|L|M|D|E K(T) | 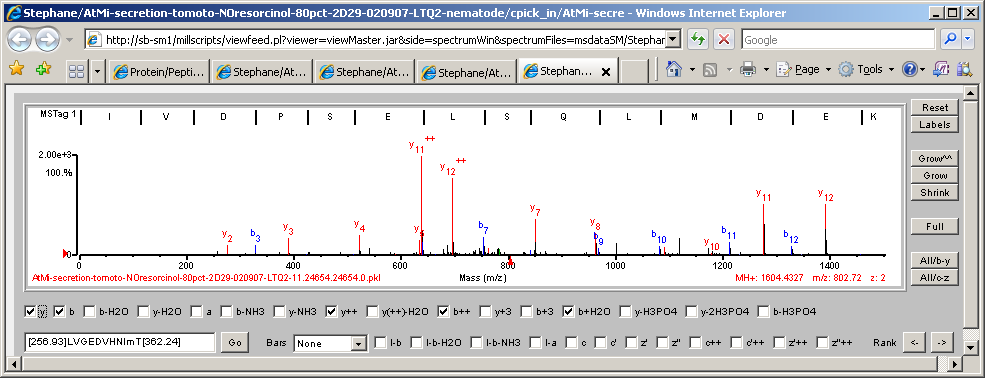 |
| 356 | *M. incognita* | MI04073 | (K)I V/A E/A/A|N|G|P T/T|P A A D R(V) | 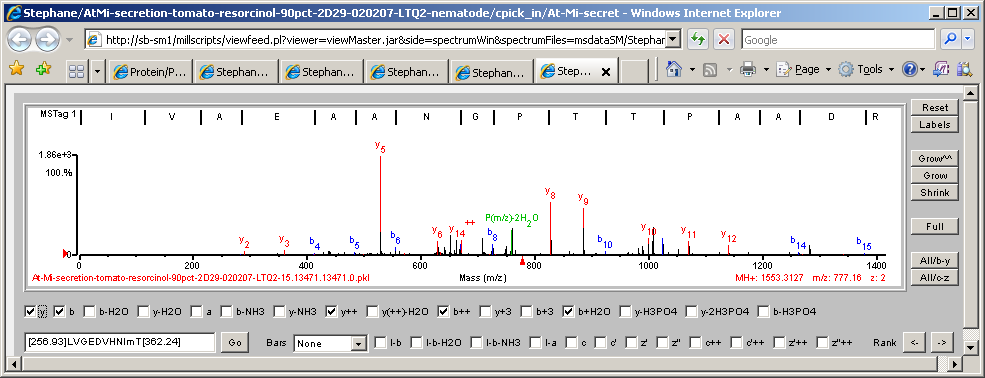 |
| 357 | *M. incognita* | MI02767 | (K)L G T I/A/D/G D/G D|I|Y/S/Y/S/E D|A P V R(D) | 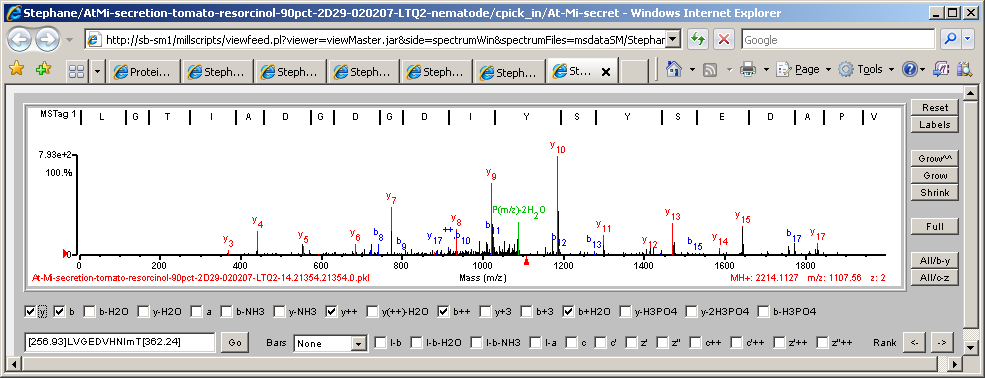 |
| 358 | *M. incognita* | MI08334 | (R)S S Y E D D|I|P I/E Q/L/V|S L|L|A D\F K(Q) | 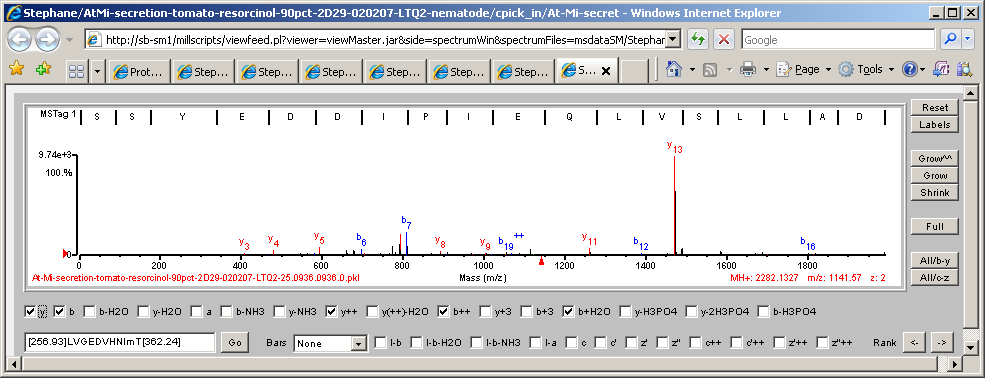 |
| 359 | *M. incognita* | MI08326 | (K)E Y/E/T/L|G/D/D/G/F|Y|G/P Q R(S) | 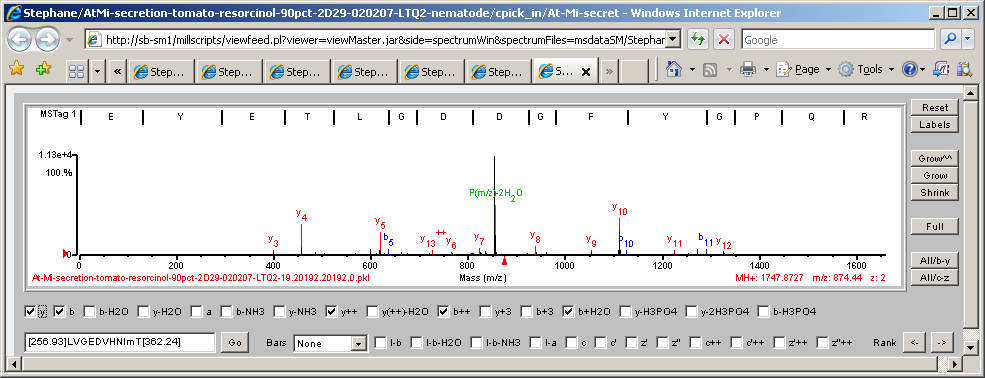 |
| 360 | *M. incognita* | MI06265 | (R)Y G D F/E|S/D|E|G/Y|P F L|A\I\S K(D) | 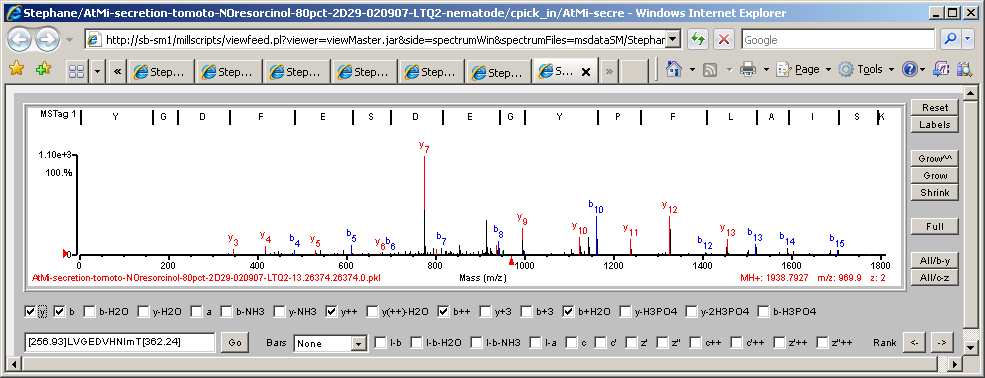 |
| 361 | *M. incognita* | MI05245 | (R)I/S/P E I|E|T|L|S/T|I E G L\K(L) | 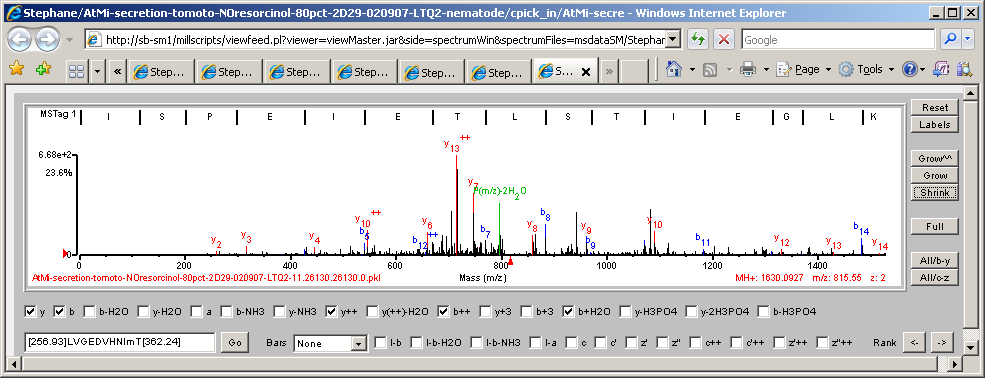 |
| 362 | *M. incognita* | MI06174 | (K)D L/N/P D/E|V|Q/E|L|E|Q\Y\K(N) | 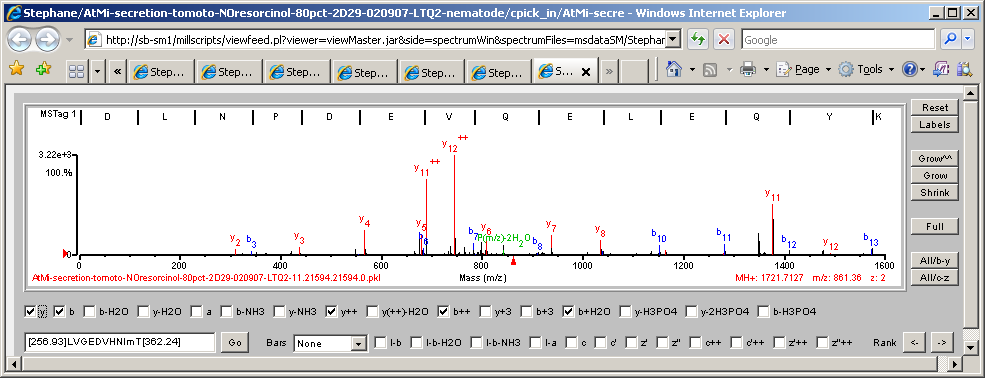 |
| 363 | *M. incognita* | MI02078 | (R)G N S I|V|I|M|E|P\M E R(I) | 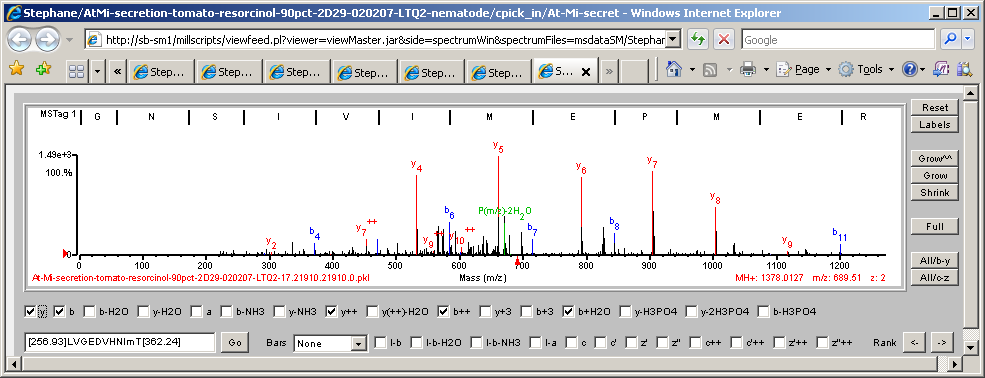 |
| 364 | *M. incognita* | MI02098 | (R)D D|F|E|T/G|Q|I|I/R(D) | 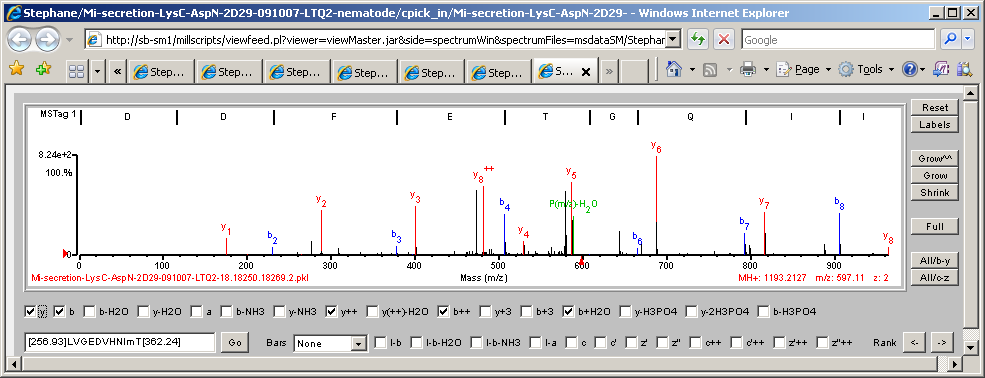 |
| 365 | *M. incognita* | MI02034 | (K)D/S/P D I/E/S/L|L|E|L|N|P R(V) | 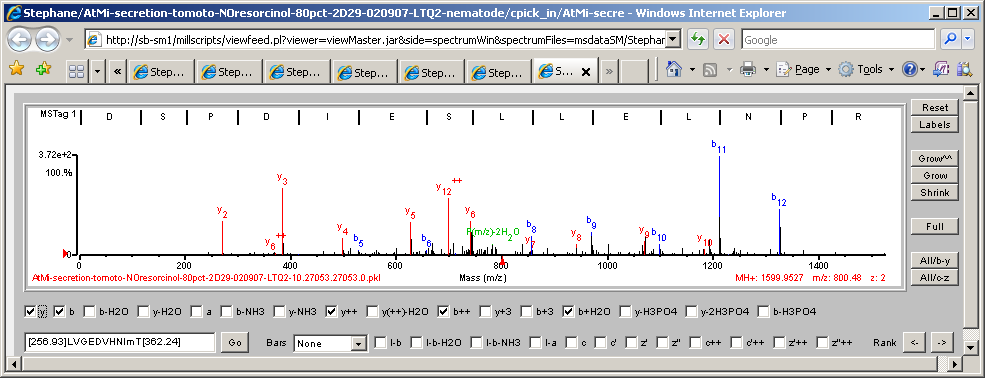 |
| 366 | *M. incognita* | MI00978 | (K)G L A/F I/E/D|P D/G Y W/I|E|I|F|N\P N K(V) | 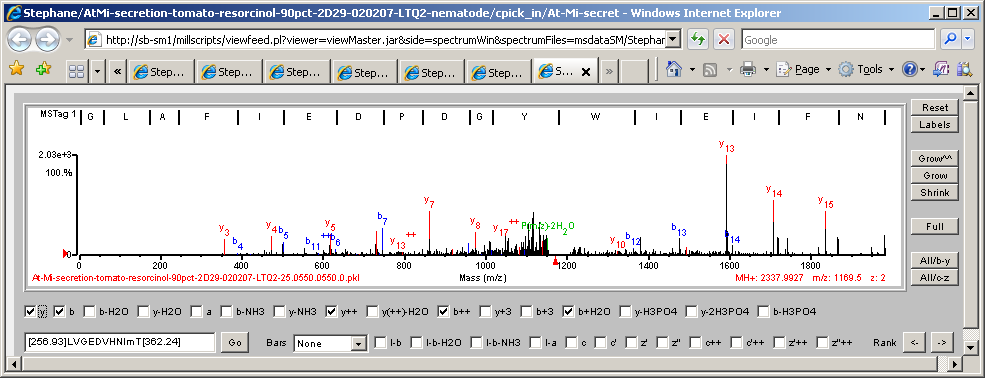 |
| 367 | *M. incognita* | MI00906 | (R)K/V/L|T|N|P L/L/Y\R(R) | 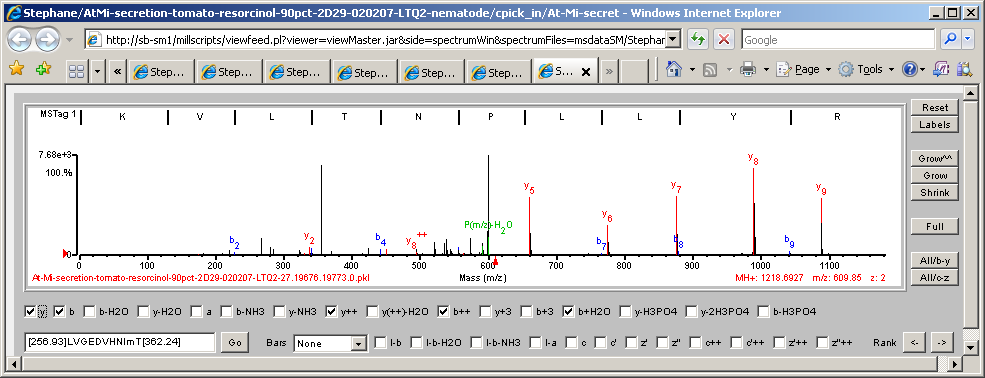 |
| 368 | *M. incognita* | MI01802 | (K)V A/N/T|S|L/L|A|L|F\R(G) |  |
| 369 | *M. incognita* | MI02234 | (K)V A G G A/Y|V|Y|S/T/T|T/A|A|T V\R(S) |  |
| 370 | *M. incognita* | MI02344 | (R)Y I E/P I E D I|P A\G/N I A/G\L|V/G/V|D/Q Y\L\V\K(G) |  |
| 371 | *M. incognita* | MI05700 | (K)F/V|D|P L/A|A|L/P K(G) |  |
| 372 | *M. incognita* | MI06613 | (K)S T/R|P V/N|S A/A\I|S|P T\R(D) |  |
| 373 | *M. incognita* | MI01515 | (K)V A/E/A/A|G/I|A/E|Q|V|T|D\K(G) |  |
| 374 | *M. incognita* | MI07562 | (K)D Q A\I|V|T|L|P\D I L|L\A|T\P R(G) |  |
| 375 | *M. incognita* | MI01648 | (K)A I/E/L/D|P N N I|V|F|Y|N N K(A) |  |
| 376 | *M. incognita* | MI09158 | (A)D N N T T/A/A/T|G/E|T/T|Q|P S A E Y I\K(I) |  |
| 377 | *M. incognita* | MI09152 | (R)I/K|K|P/G M|S V A D|V\A\K(A) |  |
| 378 | *M. incognita* | MI01602 | (K)D/G G I/T/F|L|E L|M|D|S V E\K(G) |  |
| 379 | *M. incognita* | MI04973 | (A)D A/L|Q|E|G/A|S/Q|F E K(S) |  |
| 380 | *M. incognita* | MI00316 | (K)Q V/I/Y/L|F|E/N Q|N L|W\K(H) |  |
| 381 | *M. incognita* | MI01173 | (R)E/H D/T F D|P D/D|S L/S T/T/T/T|G/P D/G T F E V R(G) |  |
| 382 | *M. incognita* | MI01389 | (A)D L|A|N/E|Y|E|R\G\E\I(D) |  |
| 383 | *M. incognita* | MI02310 | (K)D E/E E/E|F|E|E|F|P I|Y\R(E) |  |
| 384 | *M. incognita* | MI03736 | (R)D/E/D/D/D/T|P/L/H|D|D|E E\I\E\K(S) |  |
| 385 | *M. incognita* | MI03884 | (K)S A/D/T/S/Q|E/T|L/Q|S|P K(K) |  |
| 386 | *M. incognita* | MI04342 | (R)N I D D T T T/T/T|M|T|P P I|A V|T\P K(N) |  |
| 426 | Other worms | MC01039 | (K)L L/S/G/V|T/I|A Q/G/G/V|L|P N I H A V|L L\P K(K) |  |
| 427 | Other worms | GP01134 | (K)I S/Q/I|Q|D|I|V|P A L|E/L A N K(Y) |  |
| 428 | Other worms | MH02927 | (K)D/V/A/P Q/A|P T\H/F L V\I|P K(E) |  |
| 429 | Other worms | AE03258 | (R)E G D/I/L|T|L|L|E|A|E\R(E) |  |
| 430 | Other worms | MJ03828 | (R)A V/P L A L A|L|L|S|P S/N P Q L\T V/I\E T L S K(Y) |  |
| 431 | Other worms | SS01375 | (K)E A F D D/A/I|A|E|L D/T/L|N/E/D S Y K(D) |  |
| 432 | Other worms | XI01177 | (K)T I T/L/E|V|E|P S D/T I|E|N V\K(A) |  |
| 433 | Other worms | MP00840 | (K)I L/V/S/G S/G D/G Q/N/V|Q|V|T|N D|G A/T I L K(S) |  |
| 434 | Other worms | MC01143 | (K)S/P/P/A|S|P|L\K(S) |  |
| 435 | Other worms | cr01.Contig2470.wum.1.1 | (P)D/H Y Y Q P G|F T\L V\G/G/G/L M T L E A N R K\K(Q) |  |
| 436 | Other worms | MH08963 | (K)H/F/V/A/L|S\T\N\A\T\K(V) |  |
| 437 | Other worms | 58418917 | (R)H/Y|A/H/V|D|C P G H\A\D|Y\V\K(N) |  |
| 438 | Other worms | 12276043 | (K)H/F|E|L|G\G\Q\K(K) |  |
| 439 | Other worms | XI00124 | (K)V L/E/Q/L|S|G Q|T|P V|Y\S K(A) |  |
| 440 | Other worms | MC05088 | (K)L L/D/L D/N\E/H\L|G/I|P D|Q K(Y) |  |
| 441 | Other worms | 14652.m00401 | (K)L/P/I/G/D|V|A|T/Q|W|F/A D R(D) |  |
| 442 | Other worms | MH03063 | (K)I A V/T D/A E/A|A|A|N|D E|G A|P E|G\D D S E K(L) |  |
| 443 | Other worms | MP00833 | (K)T L/T|I|P E/D V/L/Q/D|I\R(R) |  |
| 444 | Other worms | 14972.m07376 | (R)A/H/Q/L/V|M|E|G/Y|N/W|C H\D\R(N) |  |
| 445 | Other worms | MP00337 | (K)N S T/N/V|E/Q|A|F|T/T/M/A/T E I\K(N) |  |
| 446 | Other worms | HC02566 | (R)V A/P E E H\P V\L\L|T\E|A|P\L\N\P(-) |  |
| 447 | Other worms | TX00152 | (R)V A P K E/H|P V|L L|T E|A|P L N\P K(A) |  |
| 448 | Other worms | SR01877 | (K)L E/L E A\P S|K\E\V\I\D|G\Q\K(E) |  |
| 449 | Other worms | 14992.m11057 | (K)T S G E S N S P Q I|S\S S T D P E I\Q|S A K C\Q\N\K(L) |  |
| 450 | Other worms | MJ00054 | (R)S D V/V/Q/G L\E|Y F|A|G|P V E L\G P K(G) |  |
| 451 | Other worms | GR00467 | (K)V/N/A/H/G/G A|V|S|I/G H|P I|G M S\G A R(I) |  |
| 452 | Other worms | AE00828 | (K)I/W/L/D|P N\E|V S|E|I|S/N A N S R(Q) |  |
| 453 | Other worms | MP01475 | (R)A M/L/S/G/P G Q|Y/A/E N/E/T/N/E/V|N F R(E) |  |
| 454 | Other worms | cr01.Contig0.wum.390.1 | (A)D S|L|L/A|E|V|K(K) |  |
| 455 | Other worms | MH05001 | (K)V S/H/C/L|S|S|V Q|A|N|L|P V\N\K(V) |  |
| 456 | Other worms | MJ00925 | (K)D D/W G S/A/L|E|A|F|E|A|A|L|A|L\E K(F) |  |
| 457 | Other worms | 31043761 | (R)D K|S V|Y/V S T I\A|P\V V T\K(V) |  |
| 458 | Other worms | 17505619 | (F)D L|T|L|E|I|M L L K(N) |  |
| 459 | Other worms | cr01.Contig1372.wum.1.1 | (R)I A/V|L|N K F T E|L\G|R P H T R(V) |  |
| 460 | Other worms | cr01.Contig174.wum.13.1 | (R)V C/Q|I F I E\V H G T P\S E H/L K M L\Q T\I A\K(Y) |  |
| 461 | Other worms | MH11009 | (R)M N/L/E|S|F|Q|P T/N/D/Y Q L\K(M) |  |
| 462 | Other worms | 14459.m00240 | (K)V L|I|P S A|A V|G|A\L/I\G K(G) |  |
| 463 | Other worms | 14992.m10966 | (R)L G E/M/P/A/D|S|G/Y|P/A/Y/L/A A R(L) |  |
| 464 | Other worms | MA02280 | (R)I N/T/E/Q|P N|P D/Y|Y/G C R(D) |  |
| 465 | Other worms | MA01250 | (K)M Y/L/S|P/G A|I|Y/T/D/T/P L E K(I) |  |
| 466 | Other worms | MA00657 | (R)E A/G V/E/Q/G D/E/E/D|L|S|T/P N E K(L) |  |
| 467 | Other worms | MC00909 | (R)K/A/D|V/D|I/H|R(R) |  |
| 468 | Other worms | MH04679 | (K)A I/P Y F W E|N/F|E|P E N/Y|S I/W/Y A E Y K(Y) |  |
| 470 | Other worms | MP01239 | (K)L/P/E|S/A|T/N/N/D|W\A R(W) |  |
| 471 | Other worms | MH02369 | (K)Y I|E/E|N|G/T/D|P V|T\G G K(L) |  |
| 472 | Other worms | MJ05164 | (K)N Q/E/H/Q|L|I|N|G G Q\E\L E\G K(L) |  |
| 473 | Other worms | MP00760 | (R)D A E/E/E|D/D\E|D/G/I|F|T\T K(D) |  |
| 474 | Other worms | MP01299 | (K)S T/D/P S N S/S Q/S/T/T/G M|L|L|D|G S S I Q K(D) |  |
| 475 | Other worms | MC00834 | (K)L/W/Q|I/A|H/S/E|Y Q\M\K(L) |  |
| 476 | Other worms | MJ02223 | (K)A D/D|P/S/A|F|I|D\V|A\K(K) |  |
| 477 | Other worms | 5833216 | (R)T Q/E/T V/T/E|T/G/Q Q L|A|P K(I) |  |
| 478 | Other worms | 126153873 | (R)D I/G V/V|Y|N/D|V|P D/P L|P T I K(V) |  |
| 479 | Other worms | MH01800 | (K)G T A E/V|V|G|E D I|S|E\A A\K(E) |  |
| 480 | Other worms | MH05913 | (R)V D/Y A/N/P D|P M|T/V|V/A/E|A/D/D\E/E/P G G G A K(R) |  |
| 481 | Other worms | MJ02674 | (K)E I/A/G Q|P E/A/D|G S I|S D\D V|P G K(N) |  |
| 486 | *Arabidopsis thaliana* | Unknown | (K)L/Y/P N/V|D|F/Y|S/G L|I N R(A) |  |
