# Supplementary material for: Direct Identification of the Meloidogyne incognita Secretome Reveals Proteins with Host Cell Reprogramming Potential
Source: PLoS Pathog. 2008 Oct 31;4(10):e1000192. doi: 10.1371/journal.ppat.1000192 (PMC2568823; doi:10.1371/journal.ppat.1000192)
Supplement: Table S3 — Protein databases used for protein identification. (0.03 MB DOC) [file ppat.1000192.s003.doc]

**Supplementary Table S3: Protein databases used for protein identification.**

| Database | Source | Type of database | Number sequences | Date of sequence collection | Comment |
| --- | --- | --- | --- | --- | --- |
| A | Parasitic nematode proteins (http://www.nematode.net) | Translated Clustered ESTs | 169,594 | 1/31/07 | References: [1,2] |
| B | NCBI nr nematode proteins | Protein | 108,230 | 10/2/07 | By searching NCBI nr proteins using “nematode” as the keyword |
| C | *M. incognita* EST from INRA-Sophia Antipolis (France) | Translated Clustered ESTs | 5,686 | 11/7/07 | unpublished |
| D | NCBI nr plant proteins | Protein | 72,309 | 6/5/07 | By limiting taxonomies to: *Arabidopsis thaliana, Glycine max, Hordeum vulgare, Lycopersicon esculentum, Maize, Nicotiana tabacum, Oryza, Oryza sativa, Pisum sativum, Solanum tuberosum, Triticum aestivum, Zea mays* |

1. McCarter JP, Mitreva MD, Martin J, Dante M, Wylie T et al. (2003) Analysis and functional classification of transcripts from the nematode Meloidogyne incognita. Genome Biology 4(4): -.

2. Wylie T, Martin JC, Dante M, Mitreva MD, Clifton SW et al. (2004) Nematode.net: a tool for navigating sequences from parasitic and free-living nematodes. Nucleic Acids Research 32: D423-D426.
