# Supplementary material for: Direct Identification of the Meloidogyne incognita Secretome Reveals Proteins with Host Cell Reprogramming Potential
Source: PLoS Pathog. 2008 Oct 31;4(10):e1000192. doi: 10.1371/journal.ppat.1000192 (PMC2568823; doi:10.1371/journal.ppat.1000192)
Supplement: Table S4 — Deduced amino acid sequences from the M. incognita EST library (INRA-Sophia Antipolis; France). Peptides identified from the secretome are in red. Sites that could be recognized by trypsin are underlined. (0.45 MB DOC) [file ppat.1000192.s004.doc]

**Supplementary Table S4: Deduced amino acid sequences from the *M. incognita* EST library (INRA-Sophia Antipolis; France).**

| **Contig** | **Amino acid Sequence** |
| --- | --- |
| **201b04r1.1_1_**AA | LIRSGHYAADDVRQRMDDVNEARKRLEDAWVQRRKILDQCLELQLFYRDCEQCDTWMSAR**EAFLAQEDPTGDNVESLIK**KHEDFDKAIASQQEKLNNLDQLAKQLVASEHYAKPAINTKREQIFDRWDRLKERLIEKR**SQLGESQTLQQFSR**DADEVENWISEKFQVAQEADYRDPTNIQQKHQKQQAFEAELSANADRIATIISAGQNLISAAXCGGGEDAVSQRLNALNDQWELLVKTSTEKSARL |
| **202**C**23r1.1_1_**AA | GEPTCAASLYGCCKDRR**TIAFGPNYGGCER**SSLSCELTEFGCCPDGTTPALGKNGKGCGASCLLTRYGCCPDGITHAKGPDNDGCGCVFSQYGCCPDGKNSAKGPGFYGCPESCAQSKYGCCPDGQTASRGPNKEGCPCQYSRWGCCKDGQTTAIGPHEEGCDDCRYGKYGCCQDGSSKAFGPDFAGC |
| **202g16**C**1.1_1_**AA | LISKCNIAGKPVICATQMLESMVSKPRPTR**AEGSDVANAVLDGADCVMLSGETAK**GEYPVETLITMHNICK**EAESAFFHAK**FFQEICKDTKKPTDKTQTIAIAATSAAISCRASAIVVMTTTGKSAIDCSRYKPPVPIIAVSRDAQVAR**QLHLYQGVFPLHYPK**MEREPDWTTDLEK**RIEVGIDEGK**SRGFIHTGDIVVVITGWRTGSGATNTLRLKTIE |
| **203b23r1.1_1_**AA | LIILFVIMTKLYGNPDNFRVKKVLVAAKLANKEIKTTNEAPPSELFPLELVPALEDGDVHLFGAVAIAKYILGNNSQYFPKDPLLEQWLFWGDNYLLSNVLSYVLPSISAAKIDQENVEKARTELLDQLTRLDSIILHKTFLVGER**ISFADISLAANLLPAYQYVLGEEER**KKICNVTRWFQTVINHPGVNEVIGELK**FITKPAK**FNDKEFEKIAGMLSRPSAEEKTPKDKGKKDKGQKQKEQTPKQEKQQ |
| **203f14r1.1_1_**AA | MSDAKLQKKDEVKSEENIPVAVTPNGSTSQQMDDGYTIQSNTNEVIESFDNMNLKELLLRGIYSFGFEKPSAIQQRAIVPCCTGR**DVIAQAQSGTGK**TATFSVSVLQRVDDKDPNVQALVMAPTRELAQQIMLVMRALGEYLDVKFHLCIGGTSVQDDQRKLQGGIQVIVGTPGRVNDMIQRESLKTKSIK**MFVLDEADEMLSR**GFKDQIYDVFKSLPNDVQVVLLSA |
| **203i02r1.1_1_**AA | MGDHLWVARYLLHRVAEEFGVIVSFDPKPISGDWNGAGCHTNFSTEKMR**KPGGYAEILNAIEALSKSHHEHIAYYDPSGGKDNER**R**LTGLHETATIDK**FSYGVASRCSSVRIPRQTEVDGYVSGRKLFKIESILKXIFRDIXEDRRPSSNCDPYLVTDALVRTCCLGVKKLSRSYIPQDAESIRKMINELRGGKIQE |
| **203i19**C**1.1_1_**AA | LLPEHREILLNPR**IIAVDQDPLGIFGR**MVYNEKSVYVFVKEMTPAILSRNLYSYAVAVLNYGTKPVQFATDLKSIGLTNKAGYSVQELWKGTNLGHFSPSTVYNITLEKNDVAIFKATLDGITKNIY |
| **203j07**C**1.1_1_**AA | YANIFTLQHRFDFIIDGETYKSIDQYYQQQKVK**DLTGISSGK**FTDGSTRDYSSLARELLRQASIKRSDVESWRTGRGLDVIQKAILEKLRQCQDMR**NALTSTGDKILVQSFGGDDYYGSGTPAK**YVKDWCSGIEKNKGSLKFPMVFPLTEEYVKYIPVIGKGK**NVLGALFMILR**EKLNNSQLDDLNFSFSKSASVGGGQTGMDFTVDSNS |
| **203n01r1.1_1_**AA | MIGXRFGNPWEKARPEYMLPVNFYGKVEKDSSGKSHWR**DTHLVFAMPYDTPVPGFR**NNVVNTLRLWSAKAENHFHLQFFNDGEYIQAVMDRNVSENITRVLYPNDNIFVGKELRLKQQYFLVAATLQDIIRRFKSSQYGNRNPIRVEFDSFPDKVAIQLNDTHPSIGIPELVRLLVDVEGLDFDHAWDICIKTFAYTNHTLLPEALERW |
| **203p11r1.1_1_**AA | MSTNNSNLLNGFNPTHVERENYKGNSILK**DSVDSVDPEIYALLK**KEKERQKLGIHLIANENFTSKAVSETVGSCLTNKYSEGYPGVRYYGGNEYIDQVENICRKRALQLFGLNSEKWGVNVQALSGSPANFAVYTAIVEPHGRLMGLELTDGGHLTHGFFSPTKKVSATSLFFESMPYRVNPNTGLIDYDLLEQNALLFRPKLIIAGISCYAQLLDYKRFREICDKCGAFLMADMAHI |
| **204h01r1.1_1_AA** | MMNLREQFSTNKYAAGRFMQLPTHNKIQVEYVWIDGSIQNVRSKTKTVDFIPKKVDELPIWNFDGSSTDQASGADSDVFIKPVAMFNDPFRLGNHKIVMCEAFDNKMQPHITNNRNHCRQTMETAKNEHAWFGMEQEYTLLDVDHHPFGWPK**APFGFPGPQGPYYCGVGANK**AYGRDIVESHYRACLYAGITIS |
| **204p08r1.1_1_**AA | MHTRVHQYSTRDQVRVPNVIALVGLPARGKTYISHKLCRYLNWIGIKTKAFNVGEYRRQACDFFQQGASDFFSPLNEQGTQIRDHCAKLAMEDMGRYLENKEGEVAIFDATNTTRERRRWLVDFCQEGEREPKFRIFFIESICDDPEIINSNIAEVKISSPDYMGKMTEEEARQDFLKRIENYK**IQYEPLNDDIDDDLSYIK**VINAGRSFYVHNVNGHVQSRVV |
| **205o21r1.1_1_**AA | FLILILLLLSAAIMGKGDRTAWKANYFTR**IISLFDDYPK**CLLVGVDNVGSKQMQEIRQAMR**GHGEILMGK**NTMIRKAIRGHLQK**NPLLEK**LLPYIVGNVGFVFTTEDLSEIRKKLLENKRGAPAK**AGALAPLDVK**LPPQNTGMGPEK**TSFFQALQIPTK**ISRGTIEILNEVHIIKLGEKVGASEAALLNMLNIQPFSYGLAVQQVFDAGAIYSPDVLDITMDDLRARFMNGVRNIAAVSLGISYP |
| **206**C**09r1.1_1_**AA | LMANQLGNLLLSSTPGETLSTAAHRELRSSEEWKSSTKR**ETFSLLNQELDR**LLKTAPANKAKDWQKEFENFKSLFIRFLRARTVIDWKEVRPLQEELIIPYAQLNMVRGQKEIVRELLSSLVVVKLNGGLGTSMGCKGPKSMISVR**NDLTFLDLTLQQIQNLNREYDVSVPLVLMNSFNTDEDTQK**LLKK**YTNVHVEVHSFPQSK**YPRIDRESYVPIARSVDDNDIECWYPPGHGNFYESFLASGLLDIFLGSRKEI |
| **206k03r1.1_1_**AA | MASRTFARIASKLRYFSHRSTNTILAARNHHFGIVRPLHSSASNLAAAAATAEKPPTPTQTTKGSKGRIVAVIGAVVDVQFDEGLPPILNSLEVQGRKGSKLILEVSQHLGDNVVRTIAMDGTEGLIR**GQEVIDTGDPIKIPVGPETLGRIMNVIGEPIDER**GPIAAKNFAPIHAEAPEFVEMNVEQEILVTGIK**VVDLLAPYSK**GGKIGLFGGAGVGK**TVLIMELINNVAK**AHGGFSVFA |
| **206l24r1.1_1_**AA | MNRIFGTSKPKAPPPNLTDAISTIDARGESIDKKIAQLDGELVKLRDQMKKMREGPSKNLVKQKALRIMKQKRTYENQRDQLSTQSFNMEQSNFAIQSMKDNQVTVAAMQSGLVAMKKEQKKLNIDKIENLQDDMEEMLEMNNEVQDALSRQYDTPDIDEADLEAELEALGDELIADGDTSYLDEAISAPSVPTK**VPGAPTAVTDHGVPVDEFGLPK**IPAT |
| **206m13r1.1_1_**AA | AIRRQCEDIIKLKPALVFTEKGISDLAQHYLVRAGITALRRLKKTDNNRLARVTGARIVNDTIDLQESDIGTQADLFEITKIGDEYFTWVTSEKTTAVTIVLRGPSK**DIINEVDR**NIQDAVSCVRNVMLKPRIVPGAGALEMALSTALTEKSK**SIQGIHNGPYK**AIANALEIIPRTLVQNCGGSVIRQLTALRAKHAQDREKNWTWGINGQTGDLADMNQLGIWDPYVVRLQALKTSIETAIMLL |
| **206n08r1.1_1_**AA | AMREKYGTQKPLKGARIAGCLHMTVQTAVLIETLLELGAEVQWSSCNIFSTQDHAAAAIAAKGISVYAWKGETEEEYEWCIEQTIIFPNGQPLNMILDDGGDLTNLVHNKYPELLNKIIGISEETTTGVHNLAKMLESGELKVAAINVNDSVTK**SKFDNLYGIR**ESLPDGIKRATDVMLAGKVAVVCGYGDVGKGSAA |
| **206p09r1.1_1_**AA | QTHNPEFTSCEFYMAYADYEDLMKLTEDLLSKMVYSLHGTYKIQYHPNGPDTVPVYQVDFTPPFKRVNMYEELGKVLGITLPPPDQLSTPEARDVFDKICIEKGVDCSAPRTTGRLLDKLIGEFIESQCINPTFLVGHPQIMCPLAKWHRSIPGLTERFELFAVTK**ELCNSYTELNDPITQR**ELFEQQAKFKADGDEEAQLVDENFCTALEYGLPPTAGWGLGLDRLAMLLTDSHNIKEVLFFPAMRPD |
| **207a11**C**1.1_1_**AA | IHPEIDNPDYKVDDELYMR**EDWGSVGIDIWQVKSGTIFDNIIVTDSIDEAK**AHAKETFEPLRDAEKKQKEAADEEERKKFDEEEKKRKEEEESKKKDEDK**DGDEEEDKDEEEEKKVEEEHDEL** |
| **207a11r1.1_1_**AA | MVSRFCFVSLLIACWPIFGVFGEVFFK**EEFSDESWTDR**WIQSKHKSDYGK**FELSSGK**FFGDKERDQGLKTSQDARFYSISAKFPKKFSNKGKTLVIQFSIK**HEQEIDCGGGYLK**LMASTINQEDFHGETPYHLMFGPDICGPGTKK**VHVIINYK**DKNQLIKKDIRCKDDVLTHLYTLILNPDNTYEVQIDGEK**AESGELEADWELLPEK**KIKDPDATKPEDWDETEYIDDP |
| **208a01r1.1_1_**AA | MSGSSRRSNEIPKKRGRSDVTNGRRDSDRSDRHRSEKSKEVFTPKTEKSKETLAEELKTKEFVYEAKMQIQERMKGLGLPLHQLSAKSDKFNETVLRPDEAIAFAQLHMEKVQRLNEMKTKLAKSLSNVNVKTPSLVLESMASDNKWGILSEQQSKLKPVKKEESNFEEKNAQTTPILKK**TIEAEPDSSTGPVVDFQDPR**LYLKPALRKRRHFDFKQPGEYEKMAK |
| **208l10r1.1_1_**AA | MGAGLSATPHDSNQVDTSETVYISSLALLKMLKHGRAGVPMEVMGLMLGDFVDDYTVNVIDVFAMPQSGTGVSVEAVDPVFQAKMIDMLKQTGRPEMVVGWYHSHPGFGCWLSGVDINTQQSFEQLSNR**AVAVVVDPIQSVK**GKVVIDAFR**TISPQTVALQQEPR**QTTCNIGHLQKPSIQALIHGLNRHYYSIPIAYRTTENEQQMLLNLNKRSWMDSMML |
| **208l23r1.1_1_**AA | MEYDLTRRLAXXFFDLHLVIPILEFIEPRKIYDEESFKKVHLSVFRK**TNMVDNLINDYSDDENLVK**EFETKKKEIMEERERLRGICNPVLTVLDRDDVKEVMESMTRDREGNTKMLEYLEQKSEFKMEMLDSLYKYAKFLYECGNYLATSTYLEFYRILIPQDDPNYLNALYGKLASEILMQSWDHARDDLNRLR**AYIDSDPFESELELLQHR**AWFLHWSLYVYFNYPKGRDEIIELFLNQQPYLNT |
| **209b10**C**1.1_1_**AA | MSSTLQINLGLNLER**KLPDTQIVILGR**KKFLKGLEFEKNQLSK**KFEPLVGNDLFK**K**AINQIETNGGSVPLYLDLAKIVSVSDDISR**HNTPSNAYAITREVKAIKSIKGIKNLSILLYCDFSNIMASCAAIAR**AFPLFSLK**TTKSGYESIEIELVLPNEEEKPTDSDIKFLQYLCTNIRECCRLVDTPADILHTEAFLDEALSQIEQTGKPVTKTIIKGEELKE |
| **209b10r1.1_1_**AA | KTVEVNNTDAEGRLVLADGVFYAKETLKAK**TIIDMATLTGFQPMTTGR**FHAAILSNYEQLENECVIAGKKSGELVHPLPFSPELHFFDMSSKVADMKNACLGVSSGPPTALAGLFIGTQIDYGGRK**NVAAVENGEGK**EGSEEKNEETKSGADWLHIDMCGLTALEKWDGRATGYGVALLCSLLSKHVDVKIAK |
| **209**C**16r1.1_1_**AA | MSVPIILLREGTQTKQGRGQILSNISACCAVADSVRTTLGPRGLDKLLVDSKGQTTISNDGATILK**QLDIVFPAAIVMSDIAKSQDAEVGDGTTSVVILAAEFLKAAKPFIEEGVSPQLIIK**AFEDASKEAINKLRELAVKIVGTTESIEMLVRCAATTLSSKLLKQERHHFSTMVVDAVSYLDIDLPLNMIGIKKVSGGSIRDSRLVKGVAFKKAFSYAGF |
| **209h03**C**1.1_1_**AA | EERCMVPSPK**EVATYDLKPEMNAAGVAEKMVEQIESGR**HPLVMCNFAPPDMVGHTGKFEPAVKACQATDEAIGKIFEACQTYNYVLMVTSDHGNAEKMIAPDGSEHTAHTCNLVPFTCSSKTFVFKSTPPTGDDGKERARALRDVAPTVLQLMGLPVPPEMDGVPLLEQRG |
| **209h03r1.1_1_**AA | MDNYQNIQQKVCLVVIDGWGLSDEQHGNAIAKAKTPIMDKLCSGNWQKLEAHGLHVGLPEGLMGNSEVGHLNIGAGRVIYQDIVRINLAVQR**NEFVTNPQIVASAER**AKKGNGRLHLLGLVSDGGVHSHIDHLFALIRAFKQLQVPKVFIHFFADGR**DTSPTSGAGYLEQLLQFIASEK**YGELATITGRYYAMDRDKRWERIKMAYEAIVGGIGQKATVDKAVDVVRERYAPS |
| **209l14r1.1_1_**AA | MRNVVSNLYLDKEMAXAVCDTIGKLKPTDSSQLENLLHDLELNISCQCFSGCLRQNNDEKFKALLIVTDSEKVEQSGGEQTLNETGRHIWEAAEMGDSLCDNNGMLLIYVRDKR**NLAVYQGHNKPLR**MFSNKDIANLHELAIQNNGTGNVTSLNDFQSVHTPPSPSSS |
| **210a01r1.1_1_**AA | MASPGEAILALHGKRTTGQSIRTQNVMAAVAISNVVKSSLGPVGLDKMLVDDVGDVTVTNDGATILKLLEVEHPAGKILVDLAQLQDEEVGDGTTSVVVVAAELLKAADELVQK**KIHPTNIINGYR**LACKEAIR**YMTENLSFPVEELDK**GLIIKTAKTSISSKLIGSESDFFAENVCG |
| **210d13r1.1_1_**AA | NNGRNKNGRGHVKPIRCTNCGRSCPKDKAIKKFVVRNIVEAAAVR**DITDASVYEQYALPK**LYHKLHYCVSCAIHSKVVRNRSREARKDRNPPPRFGQRSAADRAPRPGAQGGGTGGQGTGGPVVPALRNA |
| **210e11**C**1.1_1_**AA | HGDRSPDERTRNLKLFKEKKAKFLICTDVAARGLDVTGIPFVINVTLPPAEEKANYVHRIGRVGRAERMGLAISLVSAFPEKVWYHQCKSRGANCSNARLITQGGCAKWFDEINYLGEIEEHLGVTINR**VGPDMAVPIDEYDGK**VVYGAKRTNEGGPQFGHAVELKGILGELGNLEREVQWSYLKLLKSSQFVK |
| **210j11r1.1_1_**AA | IKR**NYEVEDLLK**LRGSIDIDYTLATRGANKLWQLLHTEPYIAALGALTGNQAVQTVRAGLKAIYLSGWQVAADANTAGDMYPDQSLYPANSGPELARKINRALRRADLVECVEAEDYKSQRDWYVPIVADAEAGFGGALNCFELMK**AYIEAGASGVHFEDQLGSEK**KCGHMGGKVLIPTAQHIRHLNAARLAADVCGVPTIIVARTDAESARLITSDIDERDHPFIDKH |
| **210k07r1.1_1_**AA | MRSIVLMFLLAVVVISFEKSIAR**SDSEDGFPGQGFPPPPPWLK**NKGSNGGRFGARPNGFGGGDGEEGPGGRPGGFGRGGGGF |
| **210k21r1.1_1_**AA | SFRLWDFQDDDRQGLHRFRCENSRPR**NFLDPNETEDYSVPIVQIDR**FVYLETGKPGILGSDIHGLKPYDRNNDIFTFLKLYNPERQQTEFKGTVILNLGNEVGAHFEEINQIIGLPPDTRLNLYVEYKPDRIIPIMNANTRIR**DVQMLTNDGSIIIAEDASK**ITEESNTQIYFRALYNRIEVEAHFHEFVLGAKPELEPPMQPIRGQIGLDWKLPKVCAWIASQIDYDPAKIILFKNATHTEKPANPLTHQHM |
| **210o05**C**1.1_1_**AA | RRFVEAGHDICLAQSFAKNMGLYGERVGAFTVICSNQEEAERVLSQLK**IIIRPMISNPPIHGAR**IAAK**ILGDSDLR**QKWLADVKSMADRIISMRVQLKELLVNAGSQRNWNHIVDQIGMFCYTGLNPEQVDRLTNEFSIYLTKDGR**ISMAGVTSGNINYVANAINEVTK** |
| **210o05r1.1_1_**AA | MSLPRINVFCLLSHGKRIKMSTFSNVEMGPPDAILGVTEAFKRDTNPKK**VNLGVGAYR**DDQGKPYVLPSVREAEAQLLAANLDKEYAGIAGIQEFTSK**AIQLALGDDSAVLK**EKRNATVQSVSGTGALRTGSEFLSKWYLPSKVVYLPSPTWANH |
| **210p13**C**1.1_1_**AA | GQRNDLTVIKFLSETSNKELCPGLNWSIEFLRTTLSLFTPYWMVNDTGKELIYQGPIEKSIQTTPQAHHLRASIFGQCCTSILPKSFTEGEKQTKHSEIKHLPNQNPIILPLNEEDFYEKKKAKLR**ISDSEWSAEFPLDSAGSSGR**ITCHSEKKDFELTVDVKLCQSGLTKVVTISPFYLLQNDSKYCIEVREPNRNEWIIMPQTSVIGFWP |
| **211k05r1.1_1_**AA | PDPEVQMLAVTQARKLLSSDRNPPIDDLISSGILPILVNCLESTNSTLQFEAAWALTNIASGTSEQTR**AVVQAGAVPHFLK**LLDSQNMNVCEQAVWALGNIIGDGPHFRDYCIELGIVQPLLKFVAPEIPLNFLRNVTWVMVNLCRSKDPPPNRQIVQMLLPALAMLIHHQDTSILVDTVWALSYLTDGGNDQIQLVIDSGVVQFLVPLLATPEVKVQTAALRAVGNIVTGTDEQTQLVLDCGALQLMQPLLSH |
| **211k16**C**1.1_1_**AA | RKHATLMNFLASMLRDEGGFEYKK**AIVDTIIAIVEDNSNAK**DLGLSHLCEFIEDCEHAALATKVLHLLGREGPTTNHPARYIRFIYNRVILETTQVRAAAVTALAKFGAECPKLRPNIIILLKRCLLDTDDEVRDRATLYLSILKSEDQTAIVTYILDTLKVSIAGLQHSLEKYVQNEHFEEPFDIKQVPLSLEPLTNNAKKEKKSTMLIEE |
| **214a10**C**1.1_1_**AA | LTVAAIFRGRMSMR**EVDDQMMSVQNKNSSYFVEWIPNNVK**TAVCDIPPRGLK**MSATFIGNSTAIQELFK**RISEQFTAMFRRKAFLHWYTGEGMDEMEFTEAESNMNDLISEYQQYQDATVEDEGEFEGEDTNQATVEQE |
| **214g02r1.1_1_**AA | MASRDYVGEDVCSLIGNTPMVYLNRLPNPNGARVAVKIEWMNPACSVKDRIAHAMVIAAEREGK**IKPGTTTLIEPTSGNTGIALAMVAAAR**GYR**LI**C**VMPSTMSGER**RTLIR**SFGAEMVLTDPVLGIK**GTIDKANELAK**IIPNSFMPLQFDNPVNPQIHYSTTGAEIFR**QTGGRIDACVFGIGTGGTMTGAGRYLRERKPNLLLYAVEPTESAVLSGGQPGPHKIQGMGAGIVPAVL |
| **214m01**C**1.1_1_**AA | QGGGDAPPQVEEQPQQEEDNAMDVGEERGDEGGSKKTADAAEKSQVPPINFEEMTEEEQMAYALR**MSVEHATESQQPSR**SRSPSMATPAPTPMETEEDGGGGQKVAGAGVVGDLLNDPARLQQLVDQRGKQSPEVKVTQYPNSDKKTDKTAGTSKEDKEKGQKK |
| **214n04r1.1_1_**AA | MSQVLAGQKIILKDESK**ANAEEYLNDK**VVGLYFSAMWCPPCRAFTPKLKKFYEDLKAAGK**NFEVIFVSR**DRTANDLK**EYYNDHHGEWTYLEFGDPKIDEFLSK**YEVKTIPTFR**IIKPDGTVVIEDAR**TEVQEKGVENALALW |
| **215a24r1.1_1_**AA | FVPPKPLYPLTENWPQLTVSVGPFDTQLLSNATTKKAPTAKTPALNAFAAEQNEDHTEGEAWGVDGDLLLDEEGNPEMDEIEMIGGEEDEEGGWDVDEEITQAVESMR**IGAGDEAADDGFTLPQR**GHAPPFYWPGNSRLTADHVAAGAFDSAARLLEESLGIVQIGPFKSLFLSTYAKSRVACCAMPLSPPNFIYPIRNWQESLPKSSLPAVSLKLNDLAQRLQIVIN |
| **215m02r1.1_1_**AA | MVQYK**LHYFDLPGR**AEAIRMLFYYKGQPFEDYRIKKEDWPTIK**SNYIFGQVPVLEVDGK**QLAQAGVILQFLGK**KFDLAGKNEWEEAKAMEIIFLNDEFGVAVGPYIGAK**FGFREGNVEQLRK**DVFLPAIER**YFPFYSLPQLKEYLSKKKC |
| **215o03**C**1.1_1_**AA | LETPKIEGKISLRTSITGQILMDEVAVPEENLLPGATGLSGPFGCLNNARLGISWGALGAAEACFQAARDYAMDR**TQFNKPIAGTQLVQLK**LADMLSEISIGLQACLRVSRLKDEGKVAPEQISLIKRNSCGKALDIARKARDILGGNGIVDEYHIMRHMVNLESVNTYEGTHDIHALILGKAITGIQAFQ |
| **217d04**C**1.1_1_**AA | PNPRGKILVDEKPAPPNPNCYVCSVKGQVLIRLNLERMLLK**TFLSTILLK**TLNMLKPDVMDICQKHRVLIFSEEGETDGIMDRTLQTLGVLNGSQLECDDYAQQLNFKIIIFHDDKLDADGFAVDSNVGNVEESVTGK**IAATALDNENALDKDEELK**RKRPSTAQETREVCMNEEDRMKDGSNDIEISPKRSRLE |
| **217d04r1.1_1_**AA | FSLIMSALDNRAARSHVNRLCLVADVPLVESGSAGYLGQVSVILKGRTECYDCTPKPIQKTFPSCTIRNTPSELIHCIVWAKSAFNQLFGENDPDDDVSPEMDSMESLPTVSENSSTDNASTNGHHTTKENSANGSSQHIEQPKIHLNTRQLAENNGYEPKHLFDKLFHTDINYLLLMADLWKERKKPTPIKFSELVSSGLGGSNVEK**VSLDPNTQWSIPR**WAQVFEESTVELACKYKEIQQQSDKNT |
| **217o11r1.1_1_**AA | VPLGQACSVVLRGATQQILDEAERSLHDALCVLTTHVRDKRVVPGAGASEMLTATAVMSESQKVPGKESLAMEAYARALIK**LPTII**C**DNAGLDSAEIISHIR**AAHVKGMHNIGIDVENGTIADVLEMGVLESHAVK**LGVIASGTEAAEQLLR**VDCIIKCAPRPRTKDRRPC |
| **218g22r1.1_1_**AA | MIESLYK**DLLEQGLSFDNDPTLIK**KAPIISNDPNVVSSDQEQADIARAIALSLQESGGNKQQNSTKANNSSYPALSSSDFNSNVNFPNSSGHQ |
| **218l10**C**1.1_1_**AA | MVVKKLKVEKKGKSSLNKVVTREYTIHLHKRIHGIGFKYRAPRAIKEVKKFAEQQMGTPDVRVDTRLNKFLWSKGIRNVPYRVRVRLSRRRNDDEDSPHK**LYTLVTHVPVTSFK**R**LTTVNVDSED** |
| **218p13**C**1.1_1_**AA | MATTKKNTRKLIGHVSHGHGRVGKHRKHPGGPGNAGGMHHHRINFDK**YHPGYFGK**VGMRNYHLRK**NSYYCPVINLDKIWTLVTEETR**KKYADVKDRAPVIDVCRAGYHKVLGKGLLPKQPVIVKARFFSHTAE |
| **219i04r1.1_1_**AA | FLPHFSKVKSVQRLKNNNKAFKXMARTKQTARKSTGGKAPRKQLATKAARKSAPATGGVKKPHR**YRPGTVALR**EIRRYQK**STELLIR**KLPFQRLVR**EIAQDFK**TDLR**FQSSAVLALQEASEAYLVGLFEDTNL**C**AIHAK**RVTIMPK**DIQLARR**IRGERT |
| **220d23r1.1_1_**AA | MKFFIIISIVFLLPLSSLALNHEGAAFCKFPKPTSTQTITKTLTIAKNTDFKMK**RIIFNGK**ENTCRKNIPGWTNNWDHAIIVENGVTISNLILGESPIGTSSDIICKGSCTLKNVFFENVCWR**AGTFIGASNSKPGDKR**KFTYIVDGGGALDGFQK**IFCTGGPGQTIVK**NFCSVNNSIGVISAGMTSIQYTR**DVTVENSKFMGPMLTIIGGNR**RYNDKLTLRNVQIYGNNK |
| **220i21**C**1.1_1_**AA | DYEKEKLNERLAKLSTGVALLK**VGGSSEVEVNEK**KDRVTDALCATR**AAVEEGIVPGGGVALLR**ALKKLDDIKAPNDDQLKGIKIVQKAVR**QPIATIVANSGLEPSGIVEKILANSNINFGYDAMKDSFVDMLEAGIIDPTK**VIRTALQDAAGVASLLSTTECVITEVPKEEPAMPAGGGMGGMGGMGGMY |
| **221l04r1.1_1_**AA | MKYYILSAILKRIILIKIIFLGTTNFALSQTITPPCSCSNVKPIFGTNSNIPQQLCVPPLAYDQKSVWLTWNKPDNYENIADFNVYMGGKKIGSAKANSVVNILSGPYIQNFYKNDLNNFHTKILFTTYLVTGLNPNTIYTFTVR**AVDANGAESGNSNQVVVK**TAENYGKIADITTFGATGDGTTLNTQTIQKAIDSCSSSTSAFGCKVSIPKGIFLSGPLFLRSQMTFELANGAILRATSDP |
| **222f20r1.1_1_**AA | MATAAGQLLFDESGQPFILIREQERQKRLTGVEALK**SHILAAK**QVANTLKTFLGPRGLDK**MLVSPDGEVTITNDGATIMK**NMDVEHHVAKLMVELSQSQDDEIGDGTTGVVVLAGALMEQAESLLDKGIHQTKIADGFEMACKKALEVLDSIAEKFPIDNREALIRCAMTSLGSKIVNKCLYQLASIAVDAILAVADLEQRDVNFELIKIVGKEGGQLEDTALIK |
| **223a18**C**1.1_1_**AA | LKTFFEPFGKPAYVNIEGNEATIRFFSVEEDAASSAWQKAVNSAKENGDSDGK**VIFEGNEITGSVLDGEEEEK**YWADFSKAKLAKHDLVEKQQKGKKQFDRNSKTNRKRRADKGSGHVAKNEKKGKRTVFDNDEDEENEKGDGKNTEEMDENALSVEDNKKTAEEDEPVEQKKKKVET |
| **223a18r1.1_1_**AA | MSASVNGNSKDTNPNKCEKIMKQVEYYFGDINLPRDKFIQEEMKKDNGWIPLSTMLKFNR**LAALTQDIENITASLK**DSHLIEISDDNLKIRRNPEVPMPENTLEYWQEIKRRTVYLKGFPLEATLDEISEFVGKFGVVENILMRKTKVGKDTPRMFKGSIFVTFKDKDQAKRLADIKDLKFRDEFQLVNKMQDAYWADKHAERVKQKDLKKQMKKTQIEQQNKAHFKKGVVLKICGMKNEDVNH |
| **223g03**C**1.1_1_**AA | MNEQIYKLFFENNLSYKMSNR**RGGFVEGKVYVGGLPEDATSEELDDAFHK**FGRIRKIWVARRPPGFAFVEFDDHRDAEDAVRSMDGTRICGVKARVELSVSASGRPSGGGRGGFRGGGGGGYGGGGGGGRGYRDRSRDRHDRDRDRDRSRSAVFSNTHTTTQTSHHQPTIFSTSSHLNPNNPSFSQFVH |
| **224n10r1.1_1_**AA | QYADDPNGGSVGQNAGSLGTNSVFRNDFHNLPPNVKTNFILLMLTSFNKTTNDLNNACLQVLAGQPSQTEMCTELKNMETK**KQEAFQEFENSLPDGSQK**TIVDQFNQQVIKNNDMTLGDKCTK**FFELLSQLDAATK**ALLIKDMFSKFQGHGGMPTSGGSNGMSGGIPGSMSGNMPGNLGAAQGGFFSGAMAPSSSSAATTSGGSANPCSIIENFMAKTAAYVENHQITGPPSS |
| **225m13**C**1.1_1_**AA | LASCSGSNKLMSQLAKESYNNNQHNVSFLSSYLLGDLQQCLEILIENGRIPEATFFAHTYAPSQVPRLVSLWR**DAASQSLSGISK**RNVGESLADPLKYENLFPGYDKALEAEKQRDGASQNGVVVPEQQDDLKDVLAESELKSLNGTVTDQEEEEHENSSGEQLSAEDEEVEDKES |
| **226d21r1.1_1_**AA | MLFPLFKNXLSPELIEDQVNEILAKHKNEILRLRYNFEFEPIFDELCKLLPNAKPEDIRKELDMRILLLLGLNQVVTRFPPEPNGILHIGHAKAINIDFGTAKAKGGITYLR**LDDTNPEAEDER**YVNEIIEMVKWLGFNPYKITHSSDYFDQLYEWAYVLINKGLAYVCHQGIEEMRGFDPPPSPWRDRPIEESIKL |
| **226g24r1.1_1_**AA | MGXDVGVNIEWLTDDQLSELSETEGRPARLVKLKQFYDALEDSKAKTEATNKIVEQCYDWLSEVVSEEEKSELEQLHEANHEECHKKVHEYLNRLGEDKRGQIEEKLPFCEHVWYGQAHKHENPENDSGKEEEKTEAKEENKVDEKKEEHQHEHHHAKKRWAHRHRRDHDHFAHDDGVHEHHELEDYLR**THLSWLTDEQK**AEMRS |
| C**L100**C**ontig1_1_**AA | MSKANAVGIDLGTTYSCVGVFQHGK**VEIIANDQGNRTTPSYVAFTDTER**LIGDAAK**NQVAMNPSNTVFDAK**RLIGRKFDDPAVQSDMKHWPFKVIQGEGARPKIQVEVKGEMK**AFFPEEVSAMVLTK**MK**ETAEAFLGQTVKDAVITVPAYFNDSQR**QATK**DAGTISGLNVLRIINEPTAAAIAYGLDKK**GQGERNVLIFDLGGGTFDVSILTIEDGIFEVK**STAGDTHLGGEDFDNRMVNHFVAEFK**RKHKKDLATNPRALRRLRTACERAKRTLSSSTQASIEIDSLFDGIDFYTNITRAR**FEEL**C**ADLFR**STMDPVEKSIRDAKMDK**SQIHDIVLVGGSTR**IPKVQKLLSDFFSGKELNK**SINPDEAVAYGAAVQAAILSGDK**SENVQDLLLLDVAPLSLGIETAGGVMTPLIKR**NTTIPTK**TSQTFTTYSDNQPGVLIQVYEGERAMTK**DNNLLGKFELSGIPPAPR**GVPQIEVTFDIDANGILNVSAQDKSTGKQNKITITNDKGR**LSKDEIER**MVQEAEK**YRGEDEIQR**DRVSAK**NGLESYCFNIK**QTMEDDKLKDKISEDDKKKVLDKC**QETLSWLDANQTAEK**EEFEHHQKELEAICNPIISKLYQSAGGAGMPGGMPGGMPGAGGMPGGGGGAGGAGGPTIEEVD |
| C**L1031**C**ontig1_1_**AA | MYYRDIRVDECNDDWKKLANMDDTKQEFIYPPLGIKPKVFVNHHFGPNSKLPMAQFNKGTTTLAFHYKPKTPNDKGGVVISVDSRASGXXVLLPKVFVNHHFGPNSKLPMAQFNKGTTTLAFHYKPKTPNDKGGVVISVDSRASGGSFIMCKDVNKILPINERMVATMAGGAADCQYWISVVTRYCNLFELREGRPITVSATSKYLANIMYSNRNNGLNLGSMVAGYDHKGPSIYLVDMSGMRCKVPDFVSVGSGSLNAYAVLDSRYKEEMTDEEAIKLGREAIMHATYR**DSGSGGSNQVLLITK**EGKTKFPLMDVSDMYYDFAKSKGIDIYKLMDKMN |
| C**L1067**C**ontig1_1_**AA | MDAIKKKMQAMKIEKDNAIDR**ADAAEEK**CRQTQERLERVEEELRDTQKKMMAIENELDK**AQEDLSSANNALEEK**EKKVQEAEAEVASLNRR**MVLLEEELDKLEER**CKVATEK**MEEATVN**C**DESER**QRKVMENRSLQDEER**ANAIEEQLKEAQSLAEEADRK**YDEVAR**KLAMVEADLER**AEERAEAGENK**IVELEEELR**VVGNNLK**SLELSEEK**AMQR**EEAYEEQVR**SMEVRLKEAETR**AEFAER**SVQKLQK**EVDRLEDELVHEK**ERYKAISEELDSTFQELSGYXRKSNNFIWLYGTGLGNNGIEKLKRFKGSFRNKIPPLKAKFFLFNCPKRGMEINLK |
| C**L107**C**ontig1_1_**AA | MDSYKDPLSKEQK**DELRAIAQK**ILQPGK**GILAADESTGSIGK**R**FEGISVENNEENR**RKYRQLLFSTPNFGQNISGVILFDETFRQKADSGERFVDLIKKNGAVAGIK**VDTGVVKLAGTLDEGTTQGLDNLSQR**CAEYKKGGCDFAKWRCVINIGPHKPSHLALLENANVLARYASICQQNGLVPIVEPEVLCDGDHDIDRCAKVTEMTLAYTYK**ALADHHIYLEGTLLKPNMVTPGQSNPK**K**ATPEEIGLATVTALR**RGVPIAVPGVVFLSGGQSEIEATQNLNAINSVPGPRPWTLTFFYGRALQSSVLKSWKGADSNIAEAQKVLLHRAK**ANGDAQLGKYTGEEGAASAAESLFVAK**HAY |
| C**L1096**C**ontig1_1_**AA | MLIPQADRKKIYEHLFEDGVCIAKKDYVSK**SHPEIK**GVRNLYVIKALKSLTSKGLVREQYAWR**HYYFYLK**EDGVPYLR**EYLGLPEDVLPNTHK**QRTEPSRMSYPDKMGGGGAGRGFRSGGDDRGAYRTMGDKQADAGPGSMPVRQGFGRGGPPTSVPTPPPQEPQGGDDGGNFATGDSGW |
| C**L109**C**ontig1_1_**AA | MAENIEEILAEIDGSQIEEYQRFFDMFDRGKNGYIMATQIGVIMNAMEQDFDEKTLRKLIRKFDADGSGK**IEFDEFCALVYTVANTVDKDTLR**KELREAFRLFDKEGNGYISRPTLK**GLLHEIAPDLSDKDLDAAVDEIDEDGSGK**IEFEEFWELMAGETD |
| C**L1101**C**ontig1_1_**AA | MINEASGTINFTMFLTLFGEKLTGTDPEEVIKNAFQCFDEDNSGTISEERLRELLTTMGDR**YTDEQVDELFR**DAPIKNGRFDYMEFTRMLKHGTKEKDEEKTK |
| C**L110**C**ontig1_1_**AA | MGRRIRAQRKGRGGIFKSHSKHRKGPAKLRPVDYSERHGYIRGIIKDIIHDPGR**GAPLAVVHFR**DPYKYRIKKELMIAAEGLYTGQFIYCGKKAQVQIGNVMPIGELPEGTTVCNLEEKAGDRGRLAR**TSGNYATVIAHKPDVK**RTR**IRLPSGAK**KVVPSANR**AMIGIVAGGGR**TDKPLLKAGRSYHKYKAKRNSWPKVRGVAMNPVEHPHGGGNHQHIGHPSTVKRGTSAGRKVGLIAARRTGRIRGGKPEKRGKDEAM |
| C**L1120**C**ontig1_1_**AA | VIAFCNTDSPLK**FIDIAIPCNLK**NKYSIGLVWWLLAR**EVLLLR**GKISRQTGFVVDGKYVMPDLYFHR**DPQEAEKEEPVESEHKETWPQAIEQTDYVAPTEPVK**LDFNVPLISDWAAASEWPQEEEAQVAPTAPIGQPQPQQQQTQQGGDWNSGTSGW |
| C**L1125**C**ontig1_1_**AA | MGRVRTKTVKKASRVIIEKYYTRLTYDFHVNKRICEEIAVIPSKRMR**NQIAGFITHLMK**RIEKGPVRGISIKLQEEERERRDNWVPEVSYLDASQHPTISVDQETK**EMVDYLQLNLPVDVK**GQQQQGQAQGGGGQQQQQQPAAAARQ |
| C**L1135**C**ontig1_1_**AA | MFR**NQYDSDVTVFSPQGR**LHQNDYAVEAMRQGSATVALHGKDHAVVVALMRSQSELSSYQSKIFELDGHVGLSMSGLLADGRILARFIQNECATFFSELQTPLPMENLRTKLTLRMQENIQVYGKRPFGVGLLIIGYDDSGPHVLNADPSANVTMVKAASIGARSQSARTYLEKHFDEYANSTDQKNVIR**HALLALK**ETLQTNTK**LDENNTAIAIVGK**RCKFGCLPSEQVKEIIASLNNNQAPEPMQL |
| C**L1149**C**ontig1_1_**AA | LSMDELKIRPRANPHQWPLFESGAQSGTEASPGGDYDKRPESFDNEMQK**VENLILEEENNAIR**MEKEQEMEEQAKVDKKEREDAALLQGDEKKNEEKIKGEEEENLKTCKSFDNEEMKGNKKKIDIVEKPVKEEDVKIGGGNVKGGNEKEAIGKKDGDTTEAKSCVKQQPQSTDSIKTSSHLPPNSDKTINLTKEEKVLSEKENLEKVTEFEEDEEEEKSKDGDGEDESERNEGDDDWMEV |
| C**L114**C**ontig1_1_**AA | MPQQLINNNNQNNINGTIVSPKNYVLEAVPDEQTLAELSCYAIDYSHTIGNVALWNDRKDCHDLSVTPPIALMPSPFPAELFEKAK**AVQETLSELYFRISLDHDFLVDAYR**DVVKADKWIAKQIELMEMVKKDGIRQNISVQLQRADYMSHWDEQQHKMELK**NVEVNIGQVGGPGCATLMSK**LHKRMMEKVGNLRYGQPLSSTVNGKLPENCPRK**GMAEAMLHAWK**LFGDKNAVIVFMNQPELFPVCHFEQLQFIQFELEDLARKEGIYINVVRMSIKETTQRLCLNESDYIMYADGRK**VALIHMAYGYLPEHFPSEK**EWNIRYDMERSKTILSPNIRLSLSGTKKIQQVLAKPGVIERFLPGQTEKIALLRSTFTGLWGLEEDNDEIRAVIK**DAIECPK**KYVMKAQLGAGK**GNYFDDQMANMLSR**MSVKERGAYILQQKIWPVVAKNYMKRPFEKPTLEDIVSEVGIYGTFIGNQENGGKVLWNRVEGYLVRSKAHNVNQGGVSEGGGVVDSLILFPENELKY |
| C**L1175**C**ontig1_1_**AA | AVGFKPTLATDMGSMQERITTTKKGSITSVQAIYVPADDLTDPAPATTFAHLDATTVLSR**GIAELGIYPAVDPLDSTSR**IMDPNIVGNRHYDVARGVQKILQDYK**SLQDIIAILGMDELSEDDKLTVSR**ARKIQRYLSQPFQVAEVFTGHKGK**FVSLEQTIDGFEK**ILRGEMDHIPEVAFYMQGDIDDVHKKAEELAKQ |
| C**L1191**C**ontig1_1_**AA | MASVPTKQDEKTKRNEELATAILKNKPKPNR**LIVDTNPNDDNSTV**C**LSQAKMDELNIFR**GDTVFLKGKKRRETACVVLASETCPNEKILMNRVVRTNLRVKLGDVVCVVPASNIPNGIRIHVLPIDDTVEGLTGNLFEVYLKPYFVEMYRPVYKGDTFTVSAAMRTVEFKVIEVDPSPACIVAPSTIIHCEGDPIKR**EDEEGNLNEVGYDDIGGMR**KQLAQIKEMVELPLRHPQLFKSIGIKPPR**GMLLFGPPGTGK**TLVAR**AVANETGAFFFLLNGPEIMSK**LAGESESNLRKAFSECEK**NSPAILFIDEIDAIAPK**REKTHGEVERR**IVSQLLTLMDGLK**QRSNVIVIGATNRPNSIDPALRRFGRFDR**EIDIGIPDAIGR**LEILRIHTKNMKLADDVDLERIANECHGYVGADLAALCSEAAMQQIRQKMELIDLEAEQIDAEVLNMLAVTMDDFQFALGKNSPSALR**ETVVETPNISWDDIGGLEGVK**R**ELQELVQYPVEHPEMFLK**FGMQPSRGVLFYGPPGCGKTLLAKAIAHECQANFISIKGPELLTMWFGESEANVRDVFDKARAAAPCVL |
| C**L124**C**ontig1_1_**AA | MTSGQAKIGK**LAPEFTTDAVVDSDFK**TVSLSDYKGKYVVLFFYPLDFTFVCPTEIIAFSERSGDFTKINVQLLACSTDSK**FSHFEWINKPR**KEGGLGEMKIPVLSDRNMKIAR**DYGVLK**EDEGIAYR**GLFIIDPK**GILRQITINDLPVGRSVDETLRLVQAFQYTDKHGEVCPANWKPGSDTIKPDPNKSKEYFGKQ |
| C**L129**C**ontig1_1_**AA | MFYKNLNKLSYGIRQLHLSKRFKHALPDLPYDYNALEPVISAEIMKIHHQK**HHATYVNNLNMTEEK**IQEALAKGDIRSVIQLQSALKFNGGGHINHSIFWTNLCKDGGEPSGKLLQAINRDFGSLQVLQARLNAIAIAVQGSGWGWLGYNKIDKRLEVACCPNQDPLEPTTGLVPLFGIDVWEHAYYLQYKNVRADYVNAIWKIADWKNIEERYESAQTD |
| C**L12**C**ontig1_1_**AA | MADQLTEEQIAEFK**EAFSLFDKDGDGTITTK**ELGTVMRSLGQNPTEAELQDMINEVDADGNGTIDFPEFLTMMARK**MKDTDSEEEIR**EAFR**VFDKDGNGFISAAELRHVMTNLGEK**LTDEEVDEMIR**EADIDGDGQVNYEEFVTMMTTK** |
| C**L130**C**ontig2_1_**AA | MLVHKCLKRFIFSILFLISLSSLNSATRTEREGINGQDVGVNIEWLTDDQLSELSETEGRPARLVKLKQFYDALEDSKAKTEATNKIVEQCYDWLSEVVSEEEKSELEQLHEANHEECHKKVHEYLNRLGEEKRGQIEEKLPFCEHVWYGQAHKHENPENNSGKEEEKAEAKEGNKAEEKEEEHHEHHHVKKRWVHRHRCNHDHFAHDDGVHEHHELEEYLRTHLSWLTEEQKVEMRSLKAEGAGKESLRSK**AMQYLEQSTGEQR**NEAIKQLVSGCRELLRRLFGAEAAEELKQMRESGTTFEELEVKIGK**LTEALEDEGK**RQKARDYGPPCKRIFALSMSVPPTTSRRRRECAQCSERLKHAIK**THLSWLSDEQK**AELKADEIAGKTREETKEKVMKWFEAIGEESEKEKARELMKGGCRELIK**EILGEEAASK**IKQMK**ESGATPEALTAEVDKLMGELTDEK**KKETAALYFSACKQVFGIKASRKRRDHHHGHGGGHTLEDYFK**THLSWLDDGQK**ENLKTMKGEEKDKKKKEKEDKKKQEKEEKD |
| C**L1325**C**ontig1_1_**AA | MSSSLPLFDLTECLANSDGWGPGPVK**ESAVLAPFEGLPYQPFNK**CDRIGRIVDWLGVDRYKKSEMRDRYNERLYGSSATAGAQFDYIHDNEDANFQLVDSGRIQRPQRPVYRRQFQFLNRKLAQREQERREYDKYNVPNKMKRSIMKEQQKAYKVFLRRGGGTRGAQQRTARRYNERQPGKGRQPSVQVRPDWEVIKELDFQQLNKLSLPNVEAGKDIEGHCYGVLHYYDKLADRISVNKAVPLQRFNGTYCHITTTEDPIIHKLASSGVGNVFATDIILATLMTCTRSVNSWDIIARRIGNKLFFDKRSVTGVSPIDALWVSETAMEPPSWDGPGVNNAK**TLADEALLLNQNFR**RQFLKRDSQHVFKFENE |
| C**L1345**C**ontig1_1_**AA | DKFRTQGIGKKPKNKDRYWKNVGLGFKTPREAIEGNYIDKKCPFTGNVSIRGRILTGVVIKNKMQRTIVIR**RDYLHFVPK**YRRYEKRHKNMSVHCSPCFRDIFIGDIVTIGECRPLSKTVRFNVLKVIKVSGTKKAFDKF |
| C**L1347**C**ontig1_1_**AA | LGFILADAGFDVWMGNVRGNVYSSKHEKSFVGKDEYWKFTWDEMASIDLPAMVDKALEISGQSKLFYVGHSQGTLIMFAQLASDNQEFKNKIIK**YFALAPVATIK**YMKGLISLSGKLFGKSFNILNKHFGSHEFLPSNWASKLFAKILCSPILSSSICDNIMFLIGGTDNSQLNETRIVVYTKHEPAGTSTR**NIAHWTQMQQSGLVQK**FDYGTENENRLIYGQIKPPIYNLTNINTIPIYLFYSDSDWLATDK**DVEETIIK**QIPKENIKIAKKLPNFNHFDFIWGINAPDKIYKGMIEEISKEN |
| C**L1354**C**ontig1_1_**AA | MSEQKEEESVPGQNTVSEIYESAAERAAMEAEFERQSEEARFR**MAHGYDQSGESVNAK**DEEGKEMAGEAFEETHNHKEPLAH |
| C**L135**C**ontig1_1_**AA | MTERLGFKQYLAQGGDWGSLIVSTLGRYYSTNLIGIHINMAFISPFDSCKHFILSLIGCYKPEWVFSESEHFKNFSIKEKYLGLLEESGYMHIQATKPDTVGVGLNDSPIGLLAYILEKFSTWTNPSFRSLPDGGILKKYSRDDILTIISIYWLQGNILSSQRYYKENFLSKNIFNSK**YVNIPTAYAAFPYDIGGATPK**ELIQLNYNLTQMTIFSDGGHFAAFEMPKELANDILNFGLKF |
| C**L139**C**ontig1_1_**AA | MMNPQRMLFSLNKSFGGFGRVVCICLLVLAISTDAFAPRSFLKR**SDDKEASAADEAQSQQQK**IPAKRLFAPQENLFFQQPQAKRFELADGYYDFPQQKRFDFLDSFYVPATYGGLPAKRFDSFGYGPRPMAAFL |
| C**L13**C**ontig6_1_**AA | MCLNLILLFVTVLIIQTEINALTLDEQNAVLACHNNFRASLANGKEQNKTGMLPSGMNIKKLTYSK**TVEASATNWANK**CTQSHTPSNQRKYGENLAMDGNAKLTTKDALLKACKNWWGEFKK**VGIQASLVVNGNNFIK**IGHATQMAWAETTEIGCGVAKCPNAPYK**TYTVCQYNKPGNYLGQAVYK**KGKACSGCGSKGCSNGLCNN |
| C**L1413**C**ontig1_1_**AA | LDLEDQQEDEGANVDLDAHKTKCAVIKTSTRATYFLPVIGLVDTAELKPGDLVGVNK**DSYLVLEK**LPPEYDSRVKAMEVDERPTETYSDIGGCDTQIK**ELIEAIVLPMTK**KEIFSNIGILPPKGVLMYGPPGTGKTMLARAVAAQTKSAFLKLAGPQLVQMFIGDGAKLVRDAFALAKEKAPAIIFIDELDAIGTKRFNSEKAGDREVQR**TMLELLNQLDGFQPNDDVK**VIAATNRVDVLDPALLRSGRLDRKIELPAPNEKARARILQLHSRKMNVSKEVNFDELARCTDDFNGAQCKAVCVESGMIALRRDATTVGHEDFMDAILEVQAKKKTVLNYYA |
| C**L1426**C**ontig1_1_**AA | VILNIMIYNK**FVEIGR**VVYITKGKDKGK**LAVIANVIDGNKALIDGPSSGVR**RCVCNFKDMQLTKFKINIRVGQRTKNIGKAYDDAEINKKWGETELAKRLARKKLRENLTDFERFKVFKAKQHRNRLIRGEMGKLKKAAKTE |
| C**L1432**C**ontig1_1_**AA | MPVQIDTINEGDGSTFPKNGQK**VS**C**HYILK**LENGQQIDSSRDR**GRPFEFTIGK**GEVIKGWDEGVAKMSVGQRAK**LTISSDLGYGEKGIPGTIPPNSTLVFDVELLAVK** |
| C**L144**C**ontig1_1_**AA | MVEKVNDVVTQVVSAK**SSDNNNNVSEAVAANSPSNK**NSPNKSDLLKVLAFQFFEAYYTKLSDHPDDIGTFYGDDSSFVLGDVEETGRENIQRAIEKMNFGACKSRFYSVKAAPEMGDNCKVFQVCGELTLKGQPAPRRFCQTIVLSCVPPNVLYVKSNIFQWLDIAFPMNSSEKVYFISAPATIPAPASITPVENGTCNINKSIEKDVVVTTSVEPILNGGVILTNGGSTHQSPKPAANLAIVSTTAEAIIAPPPGLEK**VNENVLPLQTNK**SPLPTHSVSGTPSPHQNEVVEVDKGQFTIPTSDSTNKIVTTPSPTLNNNNNIADDQKMKRPSPMKQQHQQQHSNVKSEPKSWNKVVSGGQNKMPNIQPNVTTTTTVPVKSSIDVKKTMQSQDIGSVNNTQKQRQSPPQKQHHAGNNNVGGQPQQKRSSSLGDDNTGKNNNQKQQQMSQSTKFVAPRRDWPHRIYFNNLVKPGHFLDFKTGQKELMKEIMTITPGVEFVGIKPLSLRKEYKTAFGFIDFTTGNDVQNVYDAAKKTGEKRGGDKGGGGGGGLQLRVSIPTFEFDGYITLTSDKTNNLAPGNGTGNNNNNNFR |
| C**L1451**C**ontig1_1_**AA | VPWGQAPDSLDQQYGKWRLSVFQDVQESLDTSK**LYFLYDPIADDT**C**YTTGGR**KGMTCLVVFDTNRKCFVGEINLRVQGRVKFLFALKTPSPSGGTAFALVTQSEDYGQFVMHVWRVNMNYDGMSLMADPHSLLTAPIVIDSEFICTMR**EDEPQLVVVHGPGLNVVK**INAEASAPQEPMDR**FSVPGSELSHFYDGFVSR**GNMYFLSSSPDGHFDHSR**VHILNLSTRGPLSTQYMNADPSR**GFPPPRKQAAIDSISGFILLAGGEIDYGEGGVVRLVDYWVLDLTTFK**WNQVPAQMPVPLIEPR**LTTANSGNVYLWGDFDEPLPGMPPSGTHVRILRIRGLNTTAPPPYAQATAYPVPNQQQYGGTPYPNAGGGHGWNPSQQGAVTAPPYPGAAPYPTGAAPYPAGSSSYPPGNTGSDPPYNQQGSYPHYPP |
| C**L145**C**ontig1_1_**AA | MSSSSGSVKDNKNGVVRADAGGRQGEEIASKSFMIQSKRFYLDLKQNNRGRFVKLAEVSLNGRKNRLFMTMLVCKNLKDILDKLEKDGRTAVVPKDTTEGKDNTGGVIHTETIINDRRRYYIDLRENHRGIFLRVTQFDIQTGNR**NSVALPLQGVGQFR**DALKEIIDEFGEGYIEESTDLPPSHNFRTDGK**NFFFDPGHNSR**GDFLK**ITELKPSVGVRNTIALSVGAIPQFTQILNK**LHQDFQTLRTPDGAEKATKELAKMEI |
| C**L1471**C**ontig1_1_**AA | AELAKLRR**DLEENNLNHETQLNALR**KKHNDAVAELTDQLEQMQKIKVKVEKDKQQIQRELED |
| C**L14**C**ontig3_1_**AA | MGKEKIHINIVVIGHVDSGK**STTTGHLIYK**C**GGIDK**RTIEKFEKEAQEMGKGSFKYAWVLDKLKAERERGITIDIALWKFETAK**YYVTIIDAPGHR**DFIKNMITGTSQADCAVLVVACGTGEFEAGISKNGQTR**EHALLAQTLGVKQLIVACNKMDTTEPPFSEARFEEVKNEVSSFIK**K**IGYNPATVAFVPISGFNGDNMLEPSDK**MPWFK**GWAIERK**DGNASGK**TLLEALDAILPPSRPTDKPLRLPLQDVYKIGGIGTVPVGRVETGIIKPGMVVTFAPQGISTEVKSVEMHHESLPEAVPGDNVGFNVK**NISVKDIRRGSVTSDSKNDPAKETKQFTAQVIIMNHPGQIAAGYTPVLDCHTAHIACKFAELKEKVDRRTGKKVEDNPKALK**TGDAGIVDLIPTKPM**C**VEAFTDYAPLGR**FAVRDMR**QTVAVGVIK**AVEKVEAGGKVTKSAQKAGAAAGKKK |
| C**L1518**C**ontig1_1_**AA | MK**LLLHNLLSSK**FLKGVNTGFPLILRVTKSEK**IELGENDAEQFVK**QMMPRLDYNAFRDAAQIVDQEQAVNLPSNLPNDWESNTDFLKKMYSLLVGVEIVEGEMECPETHRIFPISEGIPNMLARAD |
| C**L151**C**ontig1_1_**AA | MAVGKNKKMGKKGAKKKAVDPFTRK**DWYDIK**APAMFATRYIGKTLVNKTVGTKISSDFLKGR**VYEVSLGDLNNTEADFR**KFRLICEEVQGKVCLTNFHGMTFTRDKLCSIVKKWHTLIEANVAVKTTDGYLLRLFCIGFTKKAPNQVKKTSYAKSSQVRQIRARMVEIIHREVSSCDLKEVVSKLIPDSIGKDIEK**SCSYFYPLQDVYIR**KVKILKKPKFELGKLLDMHGEGAGGGQVAITTASGEVIESRPDAYEPPVQESV |
| C**L155**C**ontig2_1_**AA | MNFLNYFLFVVVVVEIGTKNKLIEPVHALLANEQKFVQDRHNWRRSQLANGQSQNKTGMMPTGKNIWTMKYDASLEASAQAWTNQCTMTHSGSPNGENGCFISNQETNVVNALSTCCDLWWAELVNNGVPSDLVLTSSNWYPTGHWSQMAWANTLKVGCGLAYCPNSPYK**THIFCQYSPPGNYMGQK**IYEPGPVCSGCNVVNGQLQCQYGLCI |
| C**L1575**C**ontig1_1_**AA | KKGKIYSINEGYAKKWSKGITEYIYSRKFPESGKSAYGQRYVGSMVADVHRTLLYGGIFMYPATSDAQNGKLRLLYECNPMAFIMENAGGLASHGK**GPILDIHPTTIHQR**TPIFLGSKEDVEECVGFLQKYD |
| C**L157**C**ontig1_1_**AA | DEQTNSVFLEVSVLGAEATKTKWYLEEKEIASGTGAYRMSTQEQEGGKKLIICEIKNYDKSMQGTYK**AVFFSPDGK**ENYATFTVKSGSIQTGDGEKCHHSGTNFELCKSLDPERLKNAPEFYEKPKITQKDGGKVIQIKIRVK**SHIEMK**AEWFKDESPLKTDNRIKLSTEK**DSSEPDGTILLLEINDPTK**EDQAKYKCVVKNGEGRNEQSLNLVFD |
| C**L15**C**ontig1_1_**AA | MSATVAPQPPSANANAAAANQAPVLNTPSYPMASLYVGDLHPDVTEAMLFEKFSTSGPVLSIRVCRDAITRRSLGYAYVNFQQPADAER**ALDTMNFDLLLNKPIR**IMWSQRDPSIRRSGAGNIFIKNLDKHIDTKAIYDTFSMFGNILSCKVACDLEGNSKGYGFIHFETEDAANKAIEKVNGMLLDGKKVFVGKFIPRSQREKQIGDAASRFTNVYVKNFGDAINSDK**LHEIFSAFGEIISAK**SMEDNDGKPKGFGFVAFKDHESAARSVEEMNEKEVPGRADLHFTVCRAQKKSERQSELKRRYEQFKADRIQRFQGVNLYVKNLDDTVLDDALRKHFEQHGAIASAKVMCDENGRSKGFGFVCFEKPDDATKAVVEMNNKIIGNKPLYVALAQRKEDRKAQLASQYMQRLAAMRMQNPPGIMGTMYAPASGGFFLPQALQNQRTAPFMQTTNIPGAQFRGMQPRWNGGFGHPMQGYMMGQNAGGPYANQVARGPRGGGGGMGGPGGIRGPYGGGGQQRQQQQSRMPGMAAQQGQMRYQGQPKTMQQTNAVAYSSYPNPMNQQRGPQLSGGGAGHGMAAGRPENLTNQLLTANNIQEQKQILGEQLYPMVSRYCRGEDAGKITGMILENENSEILMVLENDELLRSKVSDAVEMIQASRVAQQQPH |
| C**L1607**C**ontig1_1_**AA | LYSINKIKMSATNAEQKPTQPQK**RAHAGEENGADDGIANK**TKK**VDNGEINKNDLANTENNLKASEAADNEAISAEPK**LTKKTLEENKLADREDGGEESDAESGAVDEEEEDDEVLDDDEEDDDDAVGAEGDDQDDDEDGGEDDEGEEEEEE |
| C**L1630**C**ontig1_1_**AA | SDEPFAVQAMNFTRYKCLQHDVQIEVETMDKAGGFIGYLFVQVERGVWKNLSELLVENGLASVHFTAERSSYYTLLTNAERIAKAAKLGIWKHHVEEEQQLIDDNQKQGNDTTERKLELKKILLTEVYTGFRFAAQTFDDGLSIEKLMNALQLELKTPSIIKFIPKRNQLCAAKYTDGNLWHRARVEGVKGDNVDVLYIDFGNRETLSMSRIAPLPPQFQSQAPFAKEYQLALVSAPPDPSYAEDSMLAFKRLCFSKPYLYLNVEYRIGGLEAVTVFADDNKGEKRDLAKKLITDGYALVEKRREAR**FQQLVDDYVEQETK**ARKAHLNIWRYGDFTGSEL |
| C**L164**C**ontig1_1_**AA | MASNQELACIYAALALQDDGVPITGEKILTILK**AAGVEVEAFWAGLYAK**ALEGVDVK**ALISNIGSGIGSGGGAAASAGTAAPAAAEKAAAPAAEEK**KKKEEPKEESDDDMGFGLFD |
| C**L1656**C**ontig1_1_**AA | KKLVRKVLDMLKKLPAEKFDAFWKEYSTNIK**LGIMEDPSNR**TRLAKLLRFQSSADDKKQVTLQEYLERMK**ENQPAIYYVAGTSRQELENSPFVER**LLKKGFEVLYLTEPVDEYCIQALPEYEGKKFQNVAKEGLKLDEGKKSEEVFKQLGEQFKVLIDWLKDNGLKDLIEKAVISQRLTKSPSALVASQWGWSGNMERIMKSQAYAKSKDPSQEFYAMMKKTFEINPRHPVIKELLRRVQDDPEDPVAKSTAHLLFETATLRSGYSLSDQVGFAERIEGILRKSLDLSATAEVEDEMEIIEDEVEEKKDEDEK**KEDEETIAEEEGVTDEK**S |
| C**L1662**C**ontig1_1_**AA | MNTIPIVQPTIAGQEGEDSLYPIAVLIDELRNEDVQLRLNSIRKLSTIALALGVERTRKELIQFLTDTIYDEDEVLLALAEQLGNFTPLVGGPEYVHCLLPPLENLANIEETVVRDKAVESLQKIAEK**HSAAQLEEYFIPMVLR**LASGDWFTSRTSSCGLFSVAYGRANPARKTELRGAFRSLCRDDTPMVRRAAASKLGEFAKVQESDFLK**NDVVQIFEDLAK**DEQDSVRLLSVEAGIVIVGLLRDDATK**LAHVKPIVKDLVQDK**SWRVRYMAAVKIVEIQQAFGDKIPIEEIIGWYTGLLKDCEGEVRDAAAEKLQAFCAALPEDGRKR**GITDEILPVIKPLTTDANQHVK**IALAHVVMG |
| C**L1689**C**ontig1_1_**AA | LEMSKRGRGGASGAKFR**ISLGLPVGAVINCADNTGAK**NLFVIAVYGIRGRLNRMPSAAVGDMFVCSVKKGKPELRKKVLQAVVVRQRKQFCRKDGTFIYFEDNAGVIVNNKGEMKGFAITGPVAKECAELWPGIASNASSIA |
| C**L1707**C**ontig1_1_**AA | DKELISK**NPNLEIAQLFFLMGQK**NDSSTWLALLEHVKKDEMAPFYEYMCTSLKIPIDQALLDKLKEQNAKRLDEINKQLEDAEKNLGESEVRQAWLAKSEYLCQIGDKENSVAAFNKTFEKTVGVGYR**IDLVFNLIR**IGIFFMDHQLITENIEKAKELMEKGGDWDRKNRLRSYQGLYLMAIRDMKGAADLFLEAVPTFGAYELMNYEELIFYAVITSVCALDRPDLKTKVVNCSEIQQQLNAEIGQNNANLPIAKELLYTFYNCDYSNFMIALGKLERDFLVKDRYLQLHCRYFTRTLRIKAYQQFLTPYKTVSLDVMAKSFGVSKEYLDQQLFNFISSGKLSCRIDACKGIIETAQTDQKNLFYKKVIHDGDILLNKVQKLSRIIN |
| C**L174**C**ontig1_1_**AA | MKPKVGINGFGR**IGRLALR**AAVEKDTVQVVAVNDPFIDLDYMVYMFNYDSTHGRFKGK**IQASNGNLVVEK**EGKSTHTIKVFNFKEPEKIDWAGSGAEFVIESTGVFTTTEKASAHLKGGAKK**VVISAPSADAPMFVVGVNEDKYDPSKHHIISNASCTTNCLAPLAKVINDEFGIIEGLMTTVHAVTATQK**TVDGPSGKQWR**DGRGAGQNIIPASTGAAK**AVGKVIPELDGKLTGMAFR**VPTPNVSVVDLTARLEKPATK**EAIKNAIKTASEGKLKGILGYTEDQVVSTDFLGDTHSSIFDAEALMVLNPHFVK**LVSWYDNEFGYSCRIVDLISHIASK**SG |
| C**L1779**C**ontig1_1_**AA | GHMGGKVLIPTAQHIRHLNAARLAADVCGVPTIIVARTDAESARLITSDIDERDHPFIDKHAGRTAEGFYRLREDNAIQSCIERAKSYAPYCDLIWMETSHPTLTDAREFSEGVRKEFPDKLFAYNCSPSFNWRQHLRPSDMEKFQRELGAMGFKYQFITLAGFHTNNFSVFDLARNYKERGMAAYSELQEKEFAAEKFGYTAVKHQREVGTGYFDYVAQAAAGGISSTTALKGSTEEAQFHTATASADEDEIVTITAPENAGDETILTPDALRFLRELHQKFEPRRHSLLAQRKILQYR**LDQGDYFPDFDSTTK**NIR**DDLGWSGAEIPGDLLDR**RVEITGPTDR**KMVINALNSGAKVFMADFEDANSPTWR**NQIEGQINLHEAIRGR**IHYINPITK**QEYSLKKNTAVLKVRPR**GWHLPEK**HVLINDKPMSASLFDFGLYLFHNAKALQEKGSGPYFYLPKL |
| C**L1793**C**ontig1_1_**AA | MVVEKNSQNQFDQR**AIEYGLEELSEGK**MIIIRLTFVECAEXGLLETRATKYFLRLVLDEKTFNLLLGDKTVGIVYFRTGYLPEDYVSENAWNARLLMERSTAIV |
| C**L1795**C**ontig1_1_**AA | KPESVRTSNIVAAKAVSDSVRTSLGPRGMDKMIQAANGEVTVTNDGATILNQMSVVHPTAKMLVELSKAQDIEAGDGTTTVVILAGSLLDAAEKLLAKGIHPTTISDSFRRAATFSEK**ILDEMSTPVDINNDEELIK**LAATSLNSKVVSQHSQILAPMAVQAVKKIMTNIDDQNVNLRMIKIVKK**LGETVDVSQLIDGALIEQNSMGHGGPSR**VEKAKIGLIQFQISPPKTNMENQVVISDYTQMDRALKEERNYILELCKQIKKTGCNVLLIQKSILRDAVNELALHFFAKLKIMVIKDIER**EDIEFYSK**IIGCRPVASVDHFTPETLGTADLVEEIETADGKVVKVTGLK |
| C**L1803**C**ontig1_1_**AA | NSSRFGKFIR**VHFSGSGKLAGGDIEHYLLEK**SRVVRQAPGERCYHIFYQIMSGFDSGLRAKLQLTNELSYYHFQSQGELTIEGVD |
| C**L181**C**ontig1_1_**AA | MALDQAGR**DAVVYWHNYFR**AELVAGRVKNKTGEFLPKAKDMMQMYFSLELEKQAQAWADKCTYSHSDPYGNYGENFYAYARMDNDSAAIEYVVKGWWSELIYR**GALGPYPGQD**C**VAFDAPQNNR**GIGHWTQLAWWDTNLVGCGIGRCPKYKSYVVCQYKPPGNVYTACVYRAGEPCASCKDKCDSKTKLCLD |
| C**L1833**C**ontig1_1_**AA | VQFKEKYNIDVTKNPRAMFRLLDECEKLKKQMSANSQNIPFHIECFMNDVDVSGSMQRTQFEEMAQHLFEKINLMLHTLFERAQINVEDVSDVEIIGGSSRIPRIKQIIGEFFKKEPK**TTMNQDDAIAR**GCALRSAMFYPAYQMK**EFALEDPFDLVMEASK**LPTEKFVAELLKIEKDMQAADQRATERFDAKNALEEYCFRMQHTFEDTKACEGKVTEDVRLSCLNECFEVLEWLDMNFTELQKKQIECRHFELEKYCKPAFSKLFAVFEAKKEKQADVKQEDSNQNGKDQPNQDIEAKCEDENVDNKTSSNPKPECNDAEMD |
| C**L1842**C**ontig1_1_**AA | IIWLAYFIDFILLTLFVSDMATEQDEQLVQEIIGKVIDLAIKEDENLKISGFEANIDKDSEENKTNGSLIDSPRMDMDDKHTEENETEGLEEEKDNHSEGKKSDLIDAPRLEISGKATDLETKEGEDSKISGFEANIDEHLDEKTDGPLVGSLRMDLVDK**HTEEDKTEESLVDDAPR**SEIFGKVTDLETKEDENLKISGFEANIDEHLDKKTDGPLVGSLRMDLVDK**HTEEDKTDNEESLVVDAPR**SEISGKVTDLETKEDENLKISGFEANIDEHLDKTDGQAKSAQKARTKFCSLL |
| C**L1865**C**ontig1_1_**AA | SLFIPEVSFELLVKKQIERLEEPAITCVDLVYEEMLRIVQHCGIEVQQEMQRFPFLYERIHEVINHVLSDRLPPTKEFVSNLVSIQLAYINTKHPEFASDASLINSLQGASSDESVAIITEKVPASEADGKTASASLPNLENGTNGPSSVFGESAFYTDIHNKVNLKKLHPNYWFNRNSKQDDEDTDLVDNGTADGVLTRTDKTVRRPLSVRERRDIAVIERLIQGYFIIVRKSIQDLVPKAIMNFLVNNVKENLQSELVRR**LYNADDLNTLLSESDAIAQK**RAESAEMLKALNKANMVISEIRETHIW |
| C**L187**C**ontig1_1_**AA | MPNPRIIPKQNFHADQEVHTIYQFFTTKPGADPNYYDDYFSFFVKPSNAQRQQLKEIYRSKYGGADLVEDLKKRLPPSELATNQLFLALLDTPAYHDAKYLQKAMKGLGTDEDALIEILVTRGNIQLRQIK**HAYQELYGK**DLVK**DIASETSGTFKALLLDLLEANR**DESFRTNPEQAKIDADALYKAGEKKWGTDEAVFIKILANQNFNQLSLLFEEYQKLTKHSMEQAIKSEFSGDDCKALLSICNFVRNGIYGEVAEMLHKNIQKGVKDDTLIHLVVAHSELDLGDIADEYFKRFKIPLEQAIEKSGKSTILKRALIYMIKGNK |
| C**L195**C**ontig1_1_**AA | MSNLRLQKRLAAAVLKCGKGK**IWLDPNEVNEISNANSR**QNVRRLVKDGLIIRKPGKVHSRFRARERLEARRKGRHMGFGKRRGTRNARMPEKVLWMRRMRVLRHLLKRYRAAKKIDK**HLYHELYLK**AKGNAFKNKRNLMEYIFKKKTENQRSKQLAEQAEARRNKNKETRKRREERLILKRKELLHKLSESEKQK**TPQLQEEEAK**EASPSPPPPPKATKKQKPPPTEAKKSEEPPKPKADEPSKAKPKEAAKPKQKESGKEGGSSKKKGGK |
| C**L19**C**ontig2_1_**AA | MGQTLRRLTRRNQNQSITSTASTTTAPEVNQKKKNEEEGGVASETKTVEKQCPSSIPTTSKEEVTQQESTSKLPGR**SETVHSVPDEDDTLEEPPR**LPQGNRRR**LGVSAEVPDENEAANYQR**VVIPKDDDTQKALRQAMCRNVLFAHLDVDEQK**AIFDAMFPVEK**KEGEIIIEQGEEGDNFYVIESGEVDCFVNGEFVLSVKEGGSFGELALIYGTPRAATVVAKTPTVKLWAIDRLTYRAILMGSTMRKRKMYDEFLSK**VSILADLDKWERANVADALEQCQFEPGTR**IVEQGQPGDEFFIIVEGEAEVLQRPNDDAPYEVVGKLGPSDYFGEIALLLDHPRAATVVAKGPLKCVKLDRARFERVMGPVREILKRDVSHYNSYVKLMT |
| C**L1**C**ontig12_1_**AA | MDEEVAALVVDNGSGMCK**AGFAGDDAPRAVFPSIVGRPRHQGVMVGMGQKDSYVGDEAQSKRGILTLKYPIEHGIVTNWDDMEKIWHHTFYNELRVAPEEHPVLLTEAPLNPK**ANREKMTQIMFETFNTPAMYVAIQAVLSLYASGR**TTGIVLDSGDGVTHTVPIYEGYALPHAILR**LDLAGR**DLTDYLMK**ILTER**GYSFTTTAER**EIVRDIKEK**L**C**YVALDFEQEMATAASSSSLEKSYELPDGQVITVGNER**FRCPESLFQPSFLGMESAGIHETSYNSIMKC**DIDIRKDLYANTVLSGGTTMYPGIADR**MQK**EITALAPSTMKIKIIAPPERK**YSVWIGGSILASLSTFQQMWISK**QEYDESGPSIVHR**KCF |
| C**L1**C**ontig15_1_**AA | MPPPSNSHARGGIATNPLIIFTKMVATLLLIIPLMSEYTLGIEVFTNHFLVHLNEPGIHNAHKVAKRNGFINRGPLLGSDSEYHFVQPALSHARTRRSIGHHTKLARDPHIKYVEQMTGYKRLKRGYRPLADRLQEQLDFTAVHSPSDPLYQYQWYLKNDGQSQGKPRLDLNVEKAWALGYTGKNITTAIMDDGVDYMHPDLKLNFNAEASYDFSSNDPYPYPRYTDDWFNSHGTRCAGEISAARDNGVCGVGVAYDSKVAGIRMLDQPYMTDLIEANSMGHEPNKIHIYSASWGPTDDGKTVDGPRNATMRAIVKGVNEGRNGLGSIFVWASGDGGEDDDCNCDGYAASMWTISINSAINNGENAHYDESCSSTLASTFSNGGR**NPESGVATTDLYGR**CTRSHSGTSAAAPEAAGVFALALEANPNLSWRDLQHLTVLTSSRNSLFDGRCRELPPLNLKGVTRQLYKGLPNCSHFEWQMNGVGLEYNHLFGYGVLDAAEIVLMAKVWKTMPPRFHCEAGTIEHPTRIPPTGDLVLELNTDACVGTSTEVNFLEHVQAIVSLNTSRRGDTTLYLISPMGTPSMLLSRRPKDDDSKDGFTNWPFMTTHTWGENPRGKWRLVVRFQSGKSTPVEQQKHHTGWLKKFSLMLHGTKEAPYVGIEPLQGHANSKLSVVQGAHKRMA |
| C**L1**C**ontig18_1_**AA | MATNIAEIVANQRKFFRTGATKSLAFRKEQLQKLRVMIENNVERIVEAVYKDLRRNNELNKEMEVGGSKATIDLCLQNIDEWAKPQKVPTNKLGEPEIHYEPKGVVLVIGAWNYPVMLVIQPAAEAITAGNTVVIKPSEVSANSSHLMKELFDKTFPKNFIAVVEGGAEETKEVLNERFDHIFYTGSPNIGKIIMQAAAKYLTPVTLELGGKCPVIVENDADIEKAAQRITEGKWLNSGQTCLAPDYIMTTPETKPKLVEALQKSINKMYGSDPQKSVHYSRMVNQRHFDRVMEMMKASKGKVIHQVGKHDRADVFIPPTIVEADIK**DSLMQDEIFGPVLPILTVK**NMDEAIDIINDGEKPLGAYLFTENDKKMEKFLHSTSSGGVTVNDIIK**HAGIPDLPFGGVGNSGIGNYHGK**HGFIQLSHAKAVLKRRD |
| C**L1**C**ontig20_1_**AA | MSTKCPAPLPDADSSESCSSSQARRECDMDPLFGCHATTTPSNVPTTTASATSTSTATSLTTDSSGVSLDTSSGSQAPANVTISSLTQSTNSFATSNSTSGSCSCVLVTELRRIKFLR**LNFDQQNR**FEVCIKKIEVDLLDVTLSLSSKVIKSVLHLKLCFANNTDIEPLISCEKITGWGRIFEFIIAAFSFCADNMKLAIAPVDNIGNCILTDAIENATANDKCGNGTTAIVLTWTKWFKRICFKNDDDWPCERK**HAYIAMILR**GYCANAAPPAVWQAILKAKISLVGIDLTVEEYLALCDTYTTLSSIGDSMLSGDADSNPLLQSLNNSINSNATAQNYNSSTIAEAKNYXXQHATATSKSKQIVLNV |
| C**L1**C**ontig22_1_**AA | MSELTNGVATEDENVSGAQVEGEESEATLLKKNLK**LESQQAEEDEANKENADEKTEDQVPEITNEDPMTTSMDGIEDIANEPKEDDEQPKENGLDKAEEENTEK**DAAEIPVSILDGGEEXVEEETKEKR**DDKELTHK**VEDETKETREKRLATTKKEK**IKPVDEESKVEEETKEK**RTIKKEKTKTADIEAEADEPRKERMTKKTSTAEKKPIHTRAARATKTGTKTIESKGKGH |
| C**L1**C**ontig27_1_**AA | MNSLLLIAFLSLSFCVPIKAAPPYGQLSVKGSQLVGSNGQPVQLVGMSLFWSSCGEGEVFYNKATVNSLKCSWNSNVVRAAMGVEYSGCQRPGYLDAPNVELGK**VEAVVK**AAIELDMYVILDFHDHNAQQHVK**QAIEFFTYFAQNYGSKYPNIIYETFNEPLQVDWSGVK**SYHEQVVAEIRKYDTKNVIVLGTTTWSQDVDTAANNPVSGTNLCYTLHFYAATHKQNIRDKAQAAMNKGACIFVTEYGTVDASGGGGVDEGSTK**EWYNFMDSNKISNLNWAISNK**AEGASALTSGTSASQVGNDDRLTASGVLVKKYIKSKNTGVSCNGASPGSGSGSNPSGNKPSNSQTSTAKTSSNSGNKGGNSNTGNNANNSGSKPGNSGSNTGNTGSNAGASSGNTGTSTSGSSVTASVQVPDKWDNGARFQLVFKNNASTKKCAVKFSLTFASGQQITGIWNVQNVTGNSFVLPDYVTIEAGK**QYTDAGMNINGPATPPQIK**VLGDGKCVF |
| C**L1**C**ontig35_1_**AA | MELFSDIVPKTAENFRALCTGEKGMGHSGKPLHYKGSKFHR**VIPNFMLQGGDFTR**GNGTGGESIYGEKFADENFKEKHTCAGQLSMANAGPNTNGSQFFITTVK**TEWLDGK**HVVFGKVIEGIDVVK**AVEAIGSQSGK**TSK**DAVIADCGQLA** |
| C**L1**C**ontig37_1_**AA | MVVARSPSAAAAASMADSLFERFEVEFDAGTHPVFHGGELITGSLKIQLKKEVTINAIRIQFRGRAVYLDPKHPTKEAAEKVYFDKNFILLERPPGHPEPGHFPWSANFLYSLPFECPLPKGCETSYEGPHGFIRYYARAILETAEPDKAKYIVKQCFSIISSPELHQLVPPISDPISEKKTVRFGSCCCRGKMTAEILLPKSSYAPGEDVIGNFTMDSSTAKNALEHIEARLIDRLQRITSDLEPIKEATPTVTEPVNGVETKSGKKLKNKQKQKNGTNDNSANKAEDQQQKEETKKQKKSNSIPTTKPSPSSTNSFERHRVVFGRRLVKEDFKGETTPERRNSGENKNVITKTNVYFLRIPAIISTTQLNDQQQQQSASQFDELVDGVDAENGQFHRLLESPSTATLRARREPFLRVDYALQIYLGTHVLVEIPIQVHPIPIYAKGIGFQPFAGGAQSVAESDESSYIPYKGPFIFAPLYPVYLEPPKNLIVSEETILTPISEPAIKTTTNVKSQHPKQSINGGIKSEELQQQPFIVVTSTSTTKEENTKKTITTFPETTTTTTTITEERTTITKEEEENKIITEEIVSGKDNFPEVSELNKGGSEIKEKPLEQIIIK**EEKPETIIIEEQEQLPNGGTK**TTK**IEETILQQNGDESDEIVR**KSVVETEAYEINNDDGSTVSVQK**TTTTTTELTTTTPTVVINR**EHYNDINNDQQNEVDE |
| C**L1**C**ontig57_1_**AA | IPDYQNLSIYDFEVETLEGEFKDLSAYKGQPLLVVNVATFCAYTQQYIDFNPLIEKNLANGGQFTILAFPCNQFYLQEPAENHEIMNGIKYVRPGNGWKPHKNLHIYGKLEVNGANQHPLYEFLKDSCPQTVTQIGKREELMYNPIK**VNDVTWNFEK**FLIDAEGRPRFR**FHPTAWSHGQVVQK**FIDQLAKEKHGGSLKQVEGEIRKQ |
| C**L1**C**ontig60_1_**AA | MAKCCSVVVGRTKMMTTFPLSKQSQIYKLLLLFLLPILFCSLRGEAALLQGCDFGEELCDPGEICIPDGLFGQCYSDPEAAFVRPLVLERSLTEKQAKMLRSELSVLAKLGLDWPHARSQCVLAYFKLAAAFRLEYDPEFCDVRDPANIWALVQR**VQNSLLAEVEQVEDEEEEK**EK**QLEEAQIGEDEGQSLLTGVK**EEEENEGNEEEDNEGVGEKGRDEGREEGRDGNEEEESPPSVKLEFIPVLLLDPNEEEDKSSKNNAYQIAEENEEKGEEKDEEENKNKEEPTKLVEKTKRNDQTDTLNAPENAQLEQILQKLLSQKEGEDGQKEGMGVKTLSDKQITRLVKFVKQLQNLVEEGIERRAEEEEEKLVNSMKKGQEEVESKENEEEEEGVDEVNDLSGRPVLLLKKDSEQFNNADMGLAHTVHKIVKGGIQRVEGNRVYLRVAKENLTEEELYKLIAYLDRKIAAPNNLYFDQFLYEDGQLSFRISNPSLTMQKPTKVDSASGIAQAVYKRRKDIQTLAGVR**VDETGIGSGEDVVPVER**SQRDQLFIPILAVSA |
| C**L2084**C**ontig1_1_**AA | MVQYKLYYFDLR**GLGEAIR**LCFHYAGETFEDTRFTAENWPNEKNKFFYGK**VPVLEVDGKQLSQSGAILQYLSNK**FGEKYLFSKNSFLRXKGLAGKDDWEKAKIVEIFDFYK**DVYNELAPYIYAK**WGIREGDVDKLRKDVFIPGAERVFPLFVKLLAESGSGFFVKSGLTYVDFVLAEYFELVR**SFEPEIVGK**YKELTDFVDKVYAEPKLKTYLSTRKTT |
| C**L210**C**ontig1_1_**AA | MGTDAGDWCLIESDPGVFTELINGIGVQGVQIEELYSIDDEEHLDKLKPIYGLIFLFKWR**QGEEVEGTLQPDAPVYFAK**QVITNACASQAIINLLLNIDSNDVSLGNTLENFKNFTQNFDAGLRGLTLSNSEEIRKLHNSFSREHFMEMDLPKIK**SEDNFHFIAYVPVEDK**IYELDGLR**DAPIFLADINK**EEGNNSTDWIDIVRPFIKRRIEKYTAGEIHFNLMALVPNLRSKYEQRIAELSTMEMDSAEVGLEISQLQNLIEDEKDKMKRYKIENNRRRHNYMPFLIELLKCLAKEGKLVDLVCEAQEKKKKSATSPTKKVVK |
| C**L2116**C**ontig1_1_**AA | EGANVIFDFVTSPRTVNRFLRCLFEGGVLFVGGLCGLDVTVPIKLIARSRLAIMGITRGSIDQLK**SLVSLLAEGK**IK**APNYSIYPVDQASHVLR**QLSMSEVEGRAILEVYNPDEDKDDIEMEETQTSG |
| C**L2121**C**ontig1_1_**AA | IVLYPSDAVSTEYATELAVNYKGITFTRTGRPNTPVIYPNDEKFEIGKCKVIRQTNEDKYLLIGAGVTLYECIKAHDILSSEGIQVAVIDIFSVKPLDNQTLIEHAKRVGGKVLTVEDHYQTGGIGEAVALALGDVPNVRVRSLCVKEIPR**SGTPDELMDLYGISAK**KIIAAVKNF |
| C**L2128**C**ontig1_1_**AA | SMGFTPKKRSKRHVGKCKAFPKDDPSKPIHLTAFIGYK**AGMTHIVR**DVDKIGSKVHKKEVVEAVTIIETPPMVIVGVVGYIETPRGPRPLKAVFAEHLSEDCKRRFYKNWYKCKKKAFTKYAKKWQDEDGQK**SIEADLNK**IKKYCAYVR**VIAHTQMK**LMKHRNKKAHIMEIQINGGTVSEKVDWAREHFEKQIPVDQVFEQDEMIGCIGVTKGKGFKGVTSRWHTKKLPRKTHKGLRKVACIGAWHPSRVQFTVARAGQKGYHHRTEINKKIYRIGK**SCLTPEGK**KNGSTDFDTTEK**TINPMGGFPHYGLVNQDFVMIR**GCCVGPKKRPITLRKSLITQTKRFAFEKIDLKWIDTSSKFGHGRFQTSAEKKAFMGKLKKDFLAETTVS |
| C**L212**C**ontig1_1_**AA | FELKMGRRPARCYRYIKNKPYPKSRFCRGVPDPKIR**IFDLGR**KRATVDEFPCCVHMISNEREHLSSEALEAARICANKYMVKNCGKDGFHMRVRIHPFHVIRINKMLSCAGADRLQTGMRGAFGKPQGLVAR**VGIGTVLMSVR**VR**DQHQAHALEAFR**RAKFKFPGRQYIVVSRKWGFTQFDR**EVYENYR**KEGRVVPDGVHCKLIREHGPLTRWIKNPI |
| C**L213**C**ontig1_1_**AA | MARGPKKHLKRLAAPK**NWMLDK**LGGVFAPRPMCGPHKLR**ESLPLILFLR**NRLKYAQSYNEARMICKQRLIKVDGKVRTEMRFPAGFMDVVSIEKTGEVFRLLYDVKGRFITHR**IQKEEGQLK**LCKVVKQAIGPK**QVPYIVTHDAR**TIRYPDPHIK**VDDTVAVDINTGK**VTDHIKFDSGNVCMITGGHNMGRVGIVGHRER**HPGSFDIVHIKDSAGHAFATR**LNNVFIIGK**GTVPMISLPADK**GIRLTIAEERDKRLAQKHKAGQ |
| C**L2154**C**ontig1_1_**AA | MSIKRVHAHYIYDSR**GNPTVAVDLETDKGLFQAAVPSGASTGIYEALELR**DGDKAVHHGKGVLKAVDNVNSKIAPALIAK**NFNVTDQAAVDKFLIELDGTDNK**GNFGANAILGVSLAVCKAGAVHKGVPLYK**YIAELAGIQKVILPVPAFNMINGGSHAGNKLAMQEFMVLPVGAK**NFK**EAMQIGSEIYHHLK**KVIHKRYGLDATAVGDEGGFAPNILDNNEGLDLLLEAFKASGHADK**VVIGMDVAASEFYK**SDAK**KYDLDFK**NPNSDQTK**WLTGDQLADVYK**SFITKYNVK**SIEDAFDQDDWDNWSKLLSQTDIQLVGDDLTVTNPK**RIKQAVEKKACNCLLLK**VNQIGTVTESIEAATLARSNGWGVMVSHRSGETEDTFIADLVVGLATGQIK**TGAPCRSERLAKYNR**IMVIEDELGDGAVYAGQNYR**KP |
| C**L220**C**ontig1_1_**AA | MDFILVAILGFIFLKYILFSFLCALFRIFFPFFFPFPKNLHKLAGAKWALITGGTDGIGKEYVNQLAKKQFNIIIISRSQSKLDNVAKELREKFKGIEVR**TIAFDFTNPNLEDYNK**YILNRIDDVDIGILINNVGMFNEYPERFEKTEGGIKKLTDMALINMLPQTILSHYILRQMIPRKKGIIVNISSTINYFEWYYLAIYSASKKYAQWLSAILQAEYKGSGITIQTICPGVVSTKMSKNPKPTFTEPTPVKFVGQAIHSIGLIKETTGCFAHQLNFELLSILPNFIVAIFTKRMSLKMKNQYLKNGRFM |
| C**L221**C**ontig1_1_**AA | TMSDQQNYGKHPKDPSKPMKERMLAGELYCVNDVLEQEMNLTAKWLARLNDSSCSSRSERQQIIRERLGAMGEGCDIRPPFYCDYGSNIFMGKDVILNFNCCILDVVTVTIGDGTLFGPNVQIYPADHPRDKETR**LEGWEFGRPIK**IGKNVWIGGGAMILPGVTIGDDAIIGAGSVVTR**DVLPGTTVAGNPARPIIK**KYVN |
| C**L221**C**ontig2_1_**AA | KTMSDPQNYGKHSKDPNKPMKER**MLAGELYR**IDNVLDQEQSLTAKWLARLNDSSCSSRSERQQIIRERIGAMGDGCDIRPPFYCDYGSNIFMGKDVILNFNCCILDVVTVTIGDGTLFGPNVQVYPADHPRDKETRREGWEFGRPIKIGKNVWIGGGAMILPGVTIGDDAIIGAGSVVTR**DVLPGTTVAGNPARPIIK**KDVN |
| C**L224**C**ontig1_1_**AA | KLKKIRNMENVQGKMSNVQEQVSNAMERMGEAAQSVGQK**VSDFFQGNPFDTPVGR**KIELATDATRLATENWGLNMEICDFINSTNEGPRDAVKAIKKRLQTQMGKNNATVMYTLTVLETCVKNCDERFTTLVCHKEFVADMIRLISVKYDAPQIVQER**VLALVQSWADAFR**DNPTFSGVVEMYDELKSK**GVEFPATNLDSMAPIITPK**QTVFVPSSQTAPPQQQQFPNYPISSSQPTLQLLVQDQMAKLRSELDLVHVNMTVLRELIAQMKPGNNGKEPPEDFYFINELYSTCKEMQKRVLELIPVVANEEVTYELLSINDEFNATFEKYERCMANFNANGIDLTMVESGSKININEEKLKESGGGGSDLIDFGENN |
| C**L2258**C**ontig1_1_**AA | EMAK**LPGYSDLVDDEGNIK**GARIQVHPNLDKKEWQNNSVLRLKSEQKPYPVDVDVGILKWSVKVNDESIPLTLNCWPNVSPDSCVVSIEYTLQRTDMELRNVQITIPLPPATTPLISECDGSYEYQKSKSQLVWSLPSIDKTNKNGTLEFSTPNGHADHFFPVNIRFVSLDSFCKITVDSVEKGQESEQVEFTSEHSLITEKYEIV |
| C**L225**C**ontig1_1_**AA | GLDLQACIDLIEKPLGVISMLDEECIVPKASDMTFVQKLQDQHLGKHPNMQKPKPPKGKQAEAHFAIIHYAGTVRYNANNFLEKNKDPLNDSAVSILKSAQNNQLLLDIFEDYVTQEEAAELAKSGQSSGKKKGKSSSFLTVSMIYRESLNNLMSMLYQTHPHFIRCIIPNEKKASGVIDSALVLNQLTCNGVLEGIRICRKGFPNR**MLYPDFK**HRYAILAADESKVPDEKAASKGITDRLCREDSLKDEDFKLGNTKVFFKAGVLARLEDLRDEKLGIILTAFQTRIRWYLAQKDVKRRIQQRAGLLILQRNIRSWCTLRNWDWFKLYGKVRPLLKMGKEQEELDSLQVKIKELEESLAKEEGNRK**ELEVQVAK**LVEEKNAIFLNLEKEKAALQESEEKANKLQSLKNDLDRQLGDLQDRLGELEDRNADLQRVRKKGEQEIEGLKK**NVQDLELSLR**KAESEKQSREHNLR**SLQDEMAQQDENIAK**L |
| C**L2269**C**ontig1_1_**AA | RLCYSDFGSASPSPMKTPTSIKRSEASLIATPKTPQHKSPKGRGSTTPRSNRKKINLAGSPEDNKPDTTDMPVLSAYGKEEQDDEQVEGNNEDIK**SDDIEDKNLTNK**SSPRITRSRASNSNVTTPVKQQTPRKSPRSAKKNETK**EEEVDDYLETTDEK**IEVTINEEDLDSFNDSHNSTIIEDSTPQRKPSPFDAVQILSPVQEITELNTTGSYSSSAGSVGEAQLFVGFKFVLTSANRPNKVSDFNKRDYRTKIEERGGIVMEDFSTLQEGEHAFLIADTFYRTHKYLCALSLSIPCLSYLWIQECVSKVLFKYNFDLIFYFXKKLVEYEEFLLPAGESTSEPGRICQWYLKSFHFSLNFFSYIYRKPLKGVLFNGKRVIIYNRHYNQDPNVIPFGEIWIALMRNLGATVVGIGHGEPFSKEANLTASLE |
| C**L227**C**ontig1_1_**AA | KSSFKQSTEDQALNMQIFVKTLTGK**TITLEVEASDTIENVK**AKIQDKEGIPPDQQRLIFAGKQLEDGR**TLADYNIQKESTLHLVLR**LRGGKVHGSLARAGKVRAQTPKVEKQEHKKKKRGRAFRRIQYNRR**FTNVATSGAGR**RRGPNSNAA |
| C**L2282**C**ontig1_1_**AA | DFNLEMSDPYSLHGGTVVAMRGKDCFCIANDLRLGSQMSTIATNVQK**AHLIGDK**LFVGLTGFNCDTATFVDKIKQAK**TLYELEEDRPMKPKVLAHLISNMQYQR**RFGPYLINVLVAGIDLTSKKPFIAGSDAVGGMSEFKDFVCVGAGDEFALASCETFWRPDLTAEQLTEATAQVMLGVIERDAATGWGAIVYTVTQSGITSHALKCRLD |
| C**L228**C**ontig1_1_**AA | MSKGTVLVTGAAGFIGSHTVLQLLEADYDVLALDNFSNSIPQDEGKNNAVSLQRVSELSGKNIK**FFQADVLDLPK**LEELFEKEKFRSIIHCAALKSVGESVAKPLDYYNNNIVGSLNLIKCCQKFNVKEFIFSSSATVYGEPESLPLTEESR**VGLGITNPYGQTK**SMVERILMDLKRAEQDWKISILRYFNPVGAHPSGLIGEDPQGIPNNLMPYIAKVAVGKLPHLNIFGTNYNTPDGTGVRDYIHIVDLAKAHVSALDNIGKDIPKGSNGEELAEIYNLGTGKGYSVKEMVAALEKASGKKLTVKEVEPRLGDLAILYCDPSLALKKLGWKAEYGIDEMCRDTWNWCVKNPDGFAKKSE |
| C**L2297**C**ontig1_1_**AA | MKLIESSDTVKFPEGVKFSVKNRLVKVTGPRGTLTRDFRHLNIEITQEGANILRVRKWFGIRK**ELAAIR**TVCSHIVNMIKGVTKGFRYKMR**SVYAHFPINITLQEK**GEVIEIR**NFLGEK**FVRRVKLPEGVFAAISTK**LKDELIIDGNDVQK**VSQAAARIQQSTTVKNKDIR**KFLDGIYVSEK**VTVADD |
| C**L2360**C**ontig1_1_**AA | IMAPECAHFVPEYNDAILNNPDVAKGVDIIAWHMYGMQLVSQTKAQKMGKSAWMTEKTNDGNDWKSFMETAKDIHDCMTIANYNAYVYFWFKDPKYVSIVDNNYEITSRGYILGQYAKYIRPGYFRINATENPTKNIYVSAYKGNGKVIIVAINIGWPDKNQQFSIKRIK**SFTPIITAPNK**NMINGTKILVKNGNFNYLLPAMSVVTFVSVN |
| C**L2363**C**ontig1_1_**AA | LGDEQAFSADQNIRLVACFDNEEVGSVSAQGAASHFMEWLMRR**IVASPDEPNAFECAIGR**SYLVSADQAHACHPNYANKHEDCHKPTFNKGVVVKINANQRYATSAVTHSIFKIIAESAGIPIQKVVARSDQLCGSTVGPILSAKLGIQTIDVGCPQLAMHSIRELGDTSSIHYATTLYSQFFNKLPSVLGSLKKHEPPTLMNVD |
| C**L2370**C**ontig1_1_**AA | VDWISIYQSKIEAPFLPKCR**GPGDSSNFDDYEEEPLR**ISGTEKCAKEFAEF |
| C**L2396**C**ontig1_1_**AA | VNKMVATFEKLVKTDGKAPDDLEKQVATAITELFQINGEIKNQLSELYFVGAQQFEFGNKKCILIWIPVPQLRDYQKIHPKLVRELEKK**MSGYHIIFIAK**RRILQKPQRGKNRKPLGQKRPRSR**TLTAVHEAILQDVVFPAEVVGK**RTRVKLDGKQIIKVHLDKAQQTTVEHKVDTFSYLYKRLTGKEVVFEFPEPLF |
| C**L239**C**ontig1_1_**AA | MSGPRAILTLQKTLLGFQKYATSVQSLRNSSSAVKVTLVGASGGIGQPLALLLKKNASIAHLALYDIVGTPGVCADLSHINTPAKVTAHMGPNELSAALK**DADVVVVPAGVPR**KPGMTRDDLFNTNAAIVRDVAEAAAKACPNAFLSIITNPVNSNLPIASEVYKNNGCYDPKKIFGVTLLDVVRAQTFVSVLKNVDVTKTKIPVVGGHSGVTIVPLLSQCQPKVQLSDGEIKALSERIQEAGTEVVKAKAGAGSATLSMALAAEYFVGSLVNALKGQKSVQCAYVR**SDVVQGLDYFAGPVELGPK**GVEKILPLGELSSY320 321EKQLIEKAIPDLKKEIAKGVDFIKRGI |
| C**L2418**C**ontig1_1_**AA | TIPTKKSQVFSTAADGQTQVEIKVLQGEREMAADNKLLGQFSLIGIPPAPRGVPQVEVTFDIDANGIVNVSARDRGTGKEQQVVIQSSGGLFKDQIENMVRDAEKHAAEDAEKKETIEAINHAESVLHDTETK**MAEYADQLNEDEVK**VVKERCESFKQKLTNREALKAFEVREGINELQQKSLKLFEAAYRKMAEKNQQQHQSTDFFAKQEEGEEAAKKEGSQ |
| C**L2446**C**ontig1_1_**AA | AIIDLAQDGKWRVRLALVEYMPLLAEQLGQKFFDEKLLKLCMNWLTDHVYAIREAATNILKQLAEKFGADWAVKSVLPR**VIELAADSNYLHR**MTCLFCFNTLAEALGK**EHVLHEMFPTIK**TLCNDSVPNVRFNVAKTLTRIGK**VLDAQTINTEIKPLVTK**MGEDQEFDVRFFAEETKEALGLAY |
| C**L2458**C**ontig1_1_**AA | SAQAEKVLARDGLNALSPPRTTPEWVKFCKNLFGGFALLLWVGALLCYVAHTVDAMTLEHPSKDNLYLGIVLMTVVIITGCFQYFQERKSSQIMESFKNLVPTFALIIRDGEKRQIPSANLVVGDIVEVKGGDRIPADIRIIRAHGFK**VDNSSLTGESEPQTRTNEQSEDEPLEAR**NLAFFSTNAVEGTATGVVVNTGDRTVMGRIAHLASDLDSGKTPIAREIEHFIHIITAVAVFLGVTFFIIAFILGYHWLTAVVFLIGIIVANVPEGLIATVTVCLTLTAKRMAQRNCLVKNLEAVETLGSTSTICSDKTGTLTQNRMTVAHIWQNLNIETVNTSEDAAADDNVDKQKNASELLRVAALCNRAEFKPGQDDVPILKRDCTGDASEIALLKFAERSLYGGVIQYRAGHPKIAEIPFNSTNKYQVSIHAYKNDSNL |
| C**L2467**C**ontig1_1_**AA | APAEVVNEEARPTPGPFEGETEATENHMEGSEGQLTARTEVKSAAKEELIERQDEAMEENENEEKIKNEEEIKREVISPVEREVGSAMVEDESKNISSSPGREGMEESEEIPQEPSQACSLQQHYERVKREAEIK**AEEKEEEEVFEQPVQKEEIYEDKENEGIISPK**EEIVSTQSPENIIMNGGHYGHNEEVEGSERSVEIKEDTLKEAEPMSDEEKRVSDSRVSFEKSEDSIERSEEYPSERQKSEEAVVEMEELANQNVENEENIQESPVKVTITPSPENRSWGEEEEMKDGKEDEDEEKVVVMAEVPAESPISQHFEAGRDAEVELEGSPRVEEQLEQSESPLDSAVNGIN |
| C**L2470**C**ontig1_1_**AA | MAENK**EELVQRAKLAEQAERYDDMAQSMKKVTELGAELSNEERNLLSVAYK**NVVGARRSSWR**VISSIEQK**TEGSEKKQQMAKEYREKVERELR**DI**C**HDVLDLLDKYLIPK**AGNPESKVFYLK**MKGDYYRYLAEVATGDDRNSVVEKSQQSYQEAFDIAKDKMQPTHPIR**LGLALNFSVFYYEILNSPDKACQLAK**QAFDDAIAELDTLNEDSYKDSTLIMQLLR**DNLTLWTSDTAADEQEGAGETGETGGN |
| C**L2507**C**ontig1_1_**AA | MNGSSGIYIEQMYESWLHDKSSVHKSWDAYFSNVHAGAEPGQAFQSPSIFSQGVPTPHATLAMRDFK**EVQPSTATSVIPADDLTR**AINDHLKIQLLIRSFQTRGHNIADLDPLGINSADLDDTIPRELELDFYGFSEQDLDREFLLPPTTFIGGDKATLPLRDIFERLKKIYCHRTGIEYMHLTNYEQQDWVRKHFEAP |
| C**L252**C**ontig1_1_**AA | MKFFISFTFLLLLIGSFLIDAQFISNAEKQSGTPTIYASHKEHVGNWNPVRRRRNFLSQTQPASK**TPPSYATHSEAVGNWNPK** |
| C**L2540**C**ontig1_1_**AA | EIMASVSGAAAASAKKLVAIVTGGASGLGKATVKYLTGK**GYNVALFDLPSSEGSK**LAEEIGSGIVQFRPLDISSEDQVKEAVDSIAGQFGQLNLVVNCAGVAFAQKLYSTTKADFMKRDRIDKIIKVNLYGTINVIQYSLPHLIASTPDENNQRGLVINTSSIAAFEGQAGQALYFATKGAIASMTLPLARDYAVPGIR**FVTIAPGLFETPLMDSLPDK**VL |
| C**L2549**C**ontig1_1_**AA | QYAANNTQAQNGSQNVSSRSQHAGVENAEALPNPWSRVVNLPSQQNAEGTASAGAAAGAAGSPFAMMNTPGMQSLMRQVLSNSSTVESLLSSANMQQFGQMIGNPAMAEQMR**SLMTNANPQLFQAMANPR**VFTAMTQMHQAMQVLNEECPQLFSSMLGGDMFRNLGGILGSVNQQNQPPTSEASAATGVQQGTNSTSPQGPAVQPTANATSQNPAMPAGFAELLGSIALMNVGNMQNAGNTGAADRQPPEERYRPQLEQLTAMGFIDQQANVQALLASFGDINMAIERLLAGGLSPGGGDTPNANGGNVERTD |
| C**L254**C**ontig1_1_**AA | DGRSXSKKKKRTFRKFVYR**GVDLEQLLEMNDQQFNK**LLPSRQRRKMNRGLNRKHKSLLVRLRKAKLNAPELTKPECVKTHLRDMIVLPEMVGCVLGIHSGKAYNQVEIRAEMIGHYLGEFSITYKPVK**HGRPGIGATHSSRFIPLK** |
| C**L2550**C**ontig1_1_**AA | MDDLKNSRSSVADQDHNEAAQVNLALSDNPILREYTDRQLVASAGPRLYWEIYSATKNSTKEEVAVSIFDKKQMEKWREEQREEYLEVLKRGVSQLTGLEHPSILTVKSALEESNDSLAFCTEPVFTVLANLFDYFDYMRSTRKCYKDFNSESNEIRDGLIQLSKALVFLHHDKK**MVHSNISPNSVIINK**KGVWKLAGFDFCTVGDVSSQDKVIFGIPNWSRNSIWDLRPDLNFVSTELVQGDTFDYSSDIFSLGMLAITCY |
| C**L2552**C**ontig1_1_**AA | MRKQGVAISGRFICGNTSALSNSTKVR**IVDIDTGPDPDDTLDEK**FVDATGAFKLNGYTRELTDIDPVLYVWTRCYSLEAPCHRKIKFLIPKKFIIGEEPKLNEWLDIGIINLQSTFEDEKRECIF |
| C**L257**C**ontig2_1_AA** | MDPEEAAKEEAAKRDHRKIGKDQELFVFIPMSPGSAFWYPKGTFIYNTLVNFIRQEYRKR**GFLEVMTPNIYNVK**LWEQSGHWHHYADNMFKFEIEKEQYGLKPMNCPGHVLMFDHKPRSYNELPIRYADFGVLHRNEMSGALGGLTRLRRFQQDDAHIFCRSDQLADEITACLDFLNFVYVDVFGFSFK**LFLSTRPEDSYLGDISSWELAEK**ELSGALESSGHDWELNAGDGAFYGPKIDIQIRDALGRYWQCATIQLDFQQPQRFDLHYFDENKERHRPVMIHRAILGSVERMIAILAENFAGKWPFWLSPRQAKIICVHPNIVDYATQVKEKIFNSGFEIEFDEDCPDTLNKRIRNAQLEQFNFILVVGKREKENGTVNVRTRDNQVRGEMKVEDLIKKFAKFRDTFAK**DTESAEAFVEENNKEE** |
| C**L2605**C**ontig1_1_**AA | MREIVHIQAGQCGNQIGSK**FWEVISDEHGIQPDGSYKGESDLQLERINVYYNEAHGGK**YVPR**AVLVDLEPGTMDSIRAGPYGELFRPDNFVFGQSGAGNNWAKGHYTEGAELVDNVLDVVR**KEAEGCDCLQGFQLTHSLGGGTGSGMGTLLISKIREEYPDRIMSSFSVVPSPKVSDTVVEPYNATLSVHQLVENTDETYCIDNEALYDICFRTLKLQNPTYGDLNHLVSVTMSGVTTCL |
| C**L2624**C**ontig1_1_**AA | MGPMLTIIGGNRKYNDKLTLR**NVQIYGNNKPETNIKFA**CC**EYLGENAANPWKYSYKPGEAGTSDK**CCKYPASAVKIIN |
| C**L2662**C**ontig1_1_**AA | KHKNTDNEMTKASSK**GQLPFIELNGRQFADSNFIIDHLK**SHFKLTIDENLSDREKADARAFTILIEESFFRCLTYDRAKKXFGWLATDKGILPLQALQKFVFQKILLKQLEKRLK**NALHAQGYGR**HSPEEIEEIAKK**DLTALSTLLGEK**SFMFGDIPSTLDATAFGLLVQFTDAPMTSDKIK**TFMEQNTPNLVEFVK**RIKERYWPDWTQLCETLALNPEDI |
| C**L2678**C**ontig1_1_**AA | MVILLKLAFVLFAYLATIFAEKEKDLRVTQKVYFDISVGGVSKGR**VVIGLFGEIVPK**TAMNFLELFKGSMGFGYKGSKFHRVIRNFMIQGGDFTRGDGTGGKSIYGERFADENFELHHYGPGWLSMANAGQDTNGSQFFITTVHTPWLDGRHVVFGK**ILEGMNIVHEIEDGETGGGDRPK**LDVVIVDCGELPVAEPFIVSREGVV |
| C**L268**C**ontig1_1_**AA | SDGSDSEKSDIIESSEESLRLLDLMVQMSIADIEVPGQQVKIRDDQVRNNAGGYVFKVSDLTRVRRFLILGTDGGTYYVTEQKLTMENMEALIDIIKRGKGGMILREIVSISLAGRSPKQDPLLFALALCARYNVRDRKSLLEKSGEYDLEK**ALLSNTEDFNSDEMK**KFDLYLHKLQQTAFRMVAKVCR**IPTHLFMFVK**YCELIAKSTGEEHTGWGRAMRKCIAEWYLQKSPMELALHITKYPSRESWSHRDLLRLSHPMASSNKKELANSQTLVYDQIFHYACKGDFNPQKSIIVESEGANETPLLKKSRCDYGLTEQMIKYADDSVALEFIRKVMEMSKLKSDNGDDEKRCVELIKKYNFVREHVPTALLNSPKVWTALLQHMPMTALLRNFSKLATLGLLDDELNKECVDLVISKILNQEALQKAR**IHPIAVLLASSVYK**SGHGIKGKLKWEVNDRIEVALEQCFILAFKNVQPTGQRFCLAMDVSGSMGWTNISGGVLSCCEASTAMANVTLRTEENVKCVAFSDELITLPFTRESTITEMIDYNKKINFGGTDCSLPMIWAEENQLSFDVFIVYTDSETWAGGIHPFEALKDYRQQMNIPNAKLIVMGMASTEFTIADPDDPGMLDIVGLDAAVPDLIRSFVLEEI |
| C**L26**C**ontig3_1_**AA | MDWQGGNELNSDITNNGHNELFDFAQIGDEHFTNEDREQHWHALNWRMDKLRWMPEEEAESTIVASDYQCQPQGVDSEGSSYNAHANMSGDGACDDGLIDGSMDNDQEDQPPLGSDDEEQEDPKDYRRGGYHPVRIGDVFKNGRYHVIRKLGWGHFSTVWLCWDIESKRFIAMKIVKSAEHYTEAALDEIKLLECVRDSDPSDMALQRVVQLLDHFTVSGVNGVHVCMVFEVLGCNLLKLILKSDYQGLPIPMVKKIIKQVLEGLHYLHEKCQIIHTDIKPENVLITMSPEEVKKLAEDAILAGKTGKNLSGSAICSSKRCFKKMEESMTKSKKKKLKKKRRRHRNLLEQQLKEIEGMSVDIDSLDSGRNQFLSVDDQLNSSAFSSTERDPDNSETTTGLQLK**SEDLNDDEQIVNEQK**ATNLKIFDEIKIPRIYLNQFAQNEKEGVKAVIKKEVKEEEAGESRREENQAGINGNIISNNKQQQQEKPTKKNSVGNKILPENSNQENNKEKGPPETEPKRTGKKKKGNNAKNKQQNQQQESVKKEKRDESPSPPINRDETDDLIEGGINPKIKNDNPKKQNVNTKQEQNKQLYNGELLMPKIEVKEEIIESPQKDVEENEAIT |
| C**L2714**C**ontig1_1_**AA | MXXTFKGLIK**LINTIQPTEIYHLAAQSHVK**VSFDLPEYTAEVDAVGTLRLLDAIHACGLSNKVR**FYQASTSELYGK**VQEVPQKETTPFYPRSPYAVAKLYSYWIVVNYREAYKMFACNGILFNHESPRRGETFVTRKITRGVAK**IALNQQQFIELGNINAR**RDWGHAREYVEAMWMILQHNEPDDFVIATGKFYTVREFVELAFKEIGKEIMWEGEGINEIGIEKGTNILRVKVNPK**FYRPTEVEQLIGDPTK**AKEKLGWSAKISLKELVHEMVQSDSELMKKNPLA |
| C**L2715**C**ontig1_1_**AA | CSNQIYLATSAK**GLQGSTATVQAIK**NEIMANGPVVACFDVYNDFMSYRSGVYFVTSKDFNRQKNFLEDXANARKVGGHAVRIIGWGTQTCNGKSMPFWLIANSWSTGWGEKGLVKIRSGVNEVGIEK**SGIAFGIPKI** |
| C**L2746**C**ontig1_1_**AA | NEISK**NDSSNDTSTSNNNVEHVENVK**FDMALADSKYINPMRMHYMRKGHQLVWDFALEYDSVSVLLYHLERKELLLVKQFRPPVFIHAVREKPENVEKSLNEIEWSKYPIELGETLELCAGLIXEDKPGTSTLDHIHQEIMEECGYNVPTNLIQPIKRYVTGVSTSGSTQHLFYAEINEAMRVSDGGGNPSEGEFIKKVFMKPEEAAKFRSDINIAIAPPSLLFALTWWLNERRPAS |
| C**L2771**C**ontig1_1_**AA | MLFNSSTASSVLNQSATADKLANRMQRLISTSESSEEXGKAQQKDETAAGAAVKCFKNACGINVPRSRFLPVKKTSDLLVLMSNLYDIENGYVTLSFLRSFPTTPLVKLGSSFDKVKEFSKRFQGIPDLLELDHLTVSGDVWFGRDVTLKGTVIIIANYGDR**IDIPAGTVLENK**IVSGNLRILDH |
| C**L279**C**ontig1_1_**AA | LMTHTKVKKVEKDNVSGKLTIYTDQNAVLESVDQLIWAIGRKPLTAELNLSTVKIETTEKGYIKVDEYQNTTRNGIYAIGDVCTPKFELTPVAIAAGRRLAHRLFNGEKDNR**LLYENIPTVVFSHPPLGTTGLTER**EAVDKFGRDNIKVYTSNFTNMYFAMTTYKEKTIMKLICAGKDEKVVGIHILGMGADEMLQGFAVCVRAGLTKKQFDECVAIHPTAAEELVTMR |
| C**L2805**C**ontig1_1_**AA | MTEEKFSKLPELVKTSRYEIQLAPCLRSSTFKGSEKIYLDILKPTNYFKLHSKEIEIEKTSLKLADGTVIKELKIELDRRWELLTVHFPNELAPQTVELDLEFIGGISNELKGFYRSPYKDQAGNNKMLASTQFESVYARRAFPCFDEPTYKAHFDIQLEVDEQLTALSNMNVTEEKIIGNGKKLVTFAR**TPLMSTYLVAFAVGEFEYIESK**TNDGCIVR**VYTVPGK**KDKGHYSLSVAVKAIE |
| C**L2808**C**ontig1_1_**AA | AFDFLK**KFENVVK**NDARAMTRLISGEIHLYLLDLNENGQCKEMDLVRNMIGETEERLNRLLEVGPVHTAFYKVSAAYQRHVGDYTAYYVEALR**YLG**C**EDLENLK**MTEREEYALLLCVAALLGEEALMGGPKEWIVNALYALNAGDWNEFDKFKGKLQDEAVDILANMKLIEEKLRLLCLMGMAARHPPKQRNLFFANMAKTAPLEEDQVELLVMKALANDLIKGHFDQISQIVNITWVQPRALFFEPITAIGTRLNEWGVEVNKKGKLMGENASALFIACK |
| C**L280**C**ontig1_1_**AA | FIALQELDRQIKSFGVGPNPYTWFTMDALEETWRNLQKIIKERELELQKEHLRQEDNDKLRREFARQANDFHQWLADTRNEMMEASGSLEQQLDSIRQKAQDIRSQRQKLKKVEDLGALLEEHLILDNRYTEHSTVGLAQAWDQLDQLAMRMLHNLEQQIQARNQSGVTEEALREFSMMFRHFDREKLGRLDHQQFKSCLR**ALGYDLPMVDEGQPEPEFQR**ILDVVDPNRDGYVTLQEFMAFMINKETENVRSSEEIEMAFRALSKELRPYVTAEELYANLTPEQAEYCIKRMKPYVDAVSGRTIAGALDFEQFVHSMFQS |
| C**L2812**C**ontig1_1_**AA | MADKFVGKWK**L**C**DSTNFDEYLKQVGVGLLTR**TAAK**AIKPELEFVVDGDKWKMTSTSTFTTWVCEFLLGEEK**EQTTADGRKLKCTFTFVDGKLIEEQKKIKDDDKESHFERYIDADGKLVITCKSGNVEAVRKYEKI |
| C**L2824**C**ontig1_1_**AA | MGPPTNSNSCETMIAQPIQPYKTNEASTLRQYYTSKIQEAKQQLTEKMQNVRRLQAQRNEMNNKVRMLKEELSKLNEQGSNVGEVVKVMDKKKVLSKVHPEGKYVVDIDSSVDLAKLTAGCRVALRADSYALHMVLPNKVDPLVSLMMVEK**VPDSTYEMIGGLDSQIK**EIKEVIELPVKHPELFEALGIAQPKGVLLYGLPGTGKTLLAR**AVAHHTE**C**TFIR**VSGSELVQKFIGEGARMVKELFVMA |
| C**L285**C**ontig1_1_**AA | MKNYKFCIIALLVIYLFILQCEANIKSKNPKGNKEIKRPKRQSSFFGNLLDKPK**NNENLPDLPSTLEKENNFESEINPSSHK**FQKDENK**IGLYPEPSDNFE** |
| C**L2864**C**ontig1_1_**AA | YLGAYQQCINEAQIVKCKTDEERLKK**DYFLYR**AYIALKK**SSIPLSEINSNSGPELVALR**R**LAEYFNNVEK**RSEIIRQVTSELEQVDSEGNEYVNLLNATIFLQEDDAENALRFLSRVSDQTSLECLSMKIQCLLKLWRIDLALAELKKMQEVDEDATIVQLATAWVYMAMGKDKIKDAFYIFQEMVDKYGATPTLLVSQSSCLIQQQKYEDAEKLLMDAQQRDPNNPEALIGLFVIAHFLGKPIEVSNRYMNQLKQDHPTHIWTKDFIGKEQEFDRLVLT |
| C**L2882**C**ontig1_1_**AA | YVYKGGKIYK**VPADEMEALSTSLMGMFEK**RRFKKFLVWVQGFDKNDSK**TWEGMDPNNTIMQQVYEKFGLDDNTADFTGHALALYR**DDNYKSQPYEQTVARIRLYSDSLARYGK**SPYLYPLYGLGELPQGFARLSSIYGGTYMLDKPVDEIVFEGGK**AIGVRSGNEVAKCK**QVY**C**DPSYVDSTR**VKKVGQVVRAICLLNHPIANTNDAASCQIIIPQKQVGRHSDIYISCVSNSNQVAPKTWYIANVSTTVETQTPEAEIQPALQLLGAITEKFVMVFDIYEPIDMGYSSQVFISRSFDATTHFETTCLDVLDIFKRGTTEEFDFTKITHLSLEDNNE |
| C**L2887**C**ontig1_1_**AA | LANTNLEHLSRLNVTTSPFSVRKTGIICTIGPACRSVEMLKKMIENGMNIAR**MNFSHGDHEYHAGTIK**NVR**EAANSFNPPK**IVAIALDTKGPEIRTGLLVGGGSAEVTLEKDASIRLTTDPAFEKSSTGVCLFVDYKNIVKVVKPGSR**VFIDDGLISLIVEEITGDGAVS**C**K**VENGGKLGSRK**GVNLPGTAVDLPAVSEKDTSDLLFGVEQGVDIIFASFIR**DAEAIRTIREVLGEKGKNIKIISKIENQQGVDNADEIIEASDGVMVAR |
| C**L2889**C**ontig1_1_**AA | LPRNYEIAPGVLRFSKARMFHKRGLWEKLKKPYQKQEKKVEAKEKFIVKKIGGDKNGGERKILVQKPPK**LLPQVPQLLK**RNKRKTKKVPLRK**SITPGTVLIILVGR**HRGKRVVFLKQLEK**SGLLLVTGPLK**LNACPLRRISQSFVIATSTKIDIGK**VEVPVHVDDAYFK**RKSGKVADKSKGGIFADGAKQKYEVTEQRKTDQK**AVDAGVLDAIR**AHPEKK**YLFGYLGSR**FHLAKGMYPHKMNF |
| C**L288**C**ontig1_1_**AA | MKLETCAYSGYKIHPGHGKRVIRSDGKVQIFLSAKCQKGSGLKRNPR**DVPWTVLYR**RKHKKGIHADEGQQKKRVKR**TVHATSRPVADMTVEALLAQRNQKPEFR**KQQREAAIKAAKEAVRAKKEETKRKAVKLTKAAQQPKVKTSKQPKAPKAQVAVKR |
| C**L289**C**ontig1_1_**AA | LKEFDGKKLVSVTK**EGLELPESEEEKK**K**FEEDKVK**FEKLCKVIKDILDKKVQKVSVSNRLVSSPCCIVTGEYGWTANMERIMKAQALR**DSSTMGYMASK**K**NLEINPDHSIIK**SLRER**IDSDQDDK**TAKDLVVLLYETALLTSGFSLEDPQQHASRIYRMVKLGLDITEEDLEGGEQQPCTSGEPVEK**IAGAEEDASR**MEEVD |
| C**L28**C**ontig1_1_**AA | ATQTADSELRARQEAEKVIKNLELQLNELQTKADEQSRLLNDLAALKSRLQNENSDLEHQVEDLENQVNSLHRLK**SQLVSQLEEAK**RTADEESRERQTLSGQLK**NVQHENDSLR**EQLDDEQEGKAECLRQISKLNAEIQQWKARFEGEGLVK**LEEIEEAKR**TLQKR**LQELTDANEAANTK**IQSLEKTRHKLMGDLDDAQVDVERAAAYAAALEKKQKGFDKIIDDWRRKCDDMAAELDASQRDARNLATDLFKAK**TIQDELAETLEGTR**RENKALAQEIKDLTDQLGEGGR**SVHELQK**IVRRLEVEKEEIQHALDEAEAALEAEESKVLRAQVEVSQIRSEIEKR**IQEKEEEFENTR**KNHQRALESMQATLEAESRGKQEALRIKK**KLESDINELEIALDHANK**AYADAQKTIKKHQDQIRELQLQVEDEQRQRDELREQLFNSEKRNQILQSEKDELMHQAEQAERARRQAEADLIELR**EAVNDLSNQVNSLNGYK**RKLEGELQALHAELDETLTELKNADELSKKASADAARLAEELRQEQEHSQHVDRLRKGLELQIKEMQVRLDEAEAAALKGGKKIIAKLEERIR**ALEQELDGEQR**RHQETDKNYRKAERRVKELDFQVEEDKKNSERLTDLIDKLQGKLKVYKRQVEEAEEVAATNLGKYRQLQAQMDEAEER**ADLAENSLSK**LRAKNRSSASIAPASVGLATSASAAVLRSTSFARNSFADY |
| C**L2910**C**ontig1_1_**AA | MAPKFDPTEIKIIYLRCVGGEIGATSALAPK**VGPLGLSPK**KIGEDIAKATQDWKGLKVTCKLTIQNRIAKIDVVPSAASRIVKELKEPHRDRKKVKHVK**HSGNLTIEQIINIAR**QMRPRSMAKKLEGTVKEILGTAQSVGCTVDGQHPHDIVDAIRSGKIEIPEE |
| C**L2931**C**ontig1_1_**AA | MADVKPNHTLYINNLNEKIKKEELKKALYAIFSQFGQIIDILAFAHLKMRGQAHVIFKEINSAASALRSMQGFPFYDKPMR**IQFSHIDSDVIAK**AKGTYVERPKKNIAI |
| C**L2942**C**ontig1_1_**AA | MVNVEDDDEPEEEEYAPDGRYIPRLFILXVGEGGEGKKLKFNFXKLFKDKEGHPLAVDNAKNYPKNKQYFPQVPDVIIAMKLGLK**KLEGQEEEEDVVEEEK**EVKKDEKVKEKELKVFEN |
| C**L2965**C**ontig1_1_**AA | FFLLILLFGIADTAQNRQDDDLKEQQDDAAHQLQDXKVIVQDNRTFIGYFKAFDKHMNVILSDCEELRRVRTKTGGKKPVNEEEKR**ILGLVLLR**GDKIISISVEGPPARDDSGVQMPRAGGLGGAGQAKSAGRGVPPMGGGMIPPGPGMPSVPHGLQGAVRGIGGPGMGAMHPGYNMMDGPPRF |
| C**L2981**C**ontig1_1_**AA | RKRQVTKGQTYGKPKTHGVNKLKNAR**SHQAIAEAR**VGRRCGSLR**VLNSYWVGQDSTYKFYEVILIDPSHK**VIR**RNPDTQWITKPVHK**HREQRGLTSAGRKSRGLGKGKNYNKTIGGSRRKCWKRRNTIEFHRKR |
| C**L29**C**ontig1_1_**AA | MDTDFFPSIQPKMAEGGSEFDDEEYEYEYVDEEEQEEEPPKAAAPPPKDSSPPKSPKKEKEPSPPKSPKKEKEPSPPKSPKKDVSPDKSPVGKRKVRTPRQSMEEKPKVEKPKKLEEPTVNEEK**ENNLAVDNNNFVEPKSPSAASVTAEPGK**SFQERNISVSALKKQQMYSGGKPSRHDKAKLMRSEGIIPIQAGTNKYASQKGMTGFGAPRDVVKESKVKSENLAEITDEAKIASLKGSIWLQSGTNKFASQKGMTGMGTPRDVNYRPMGTGGAGDVPEEKAR**LTDGIVPLQSGTNK**LASQAGMTGIGMPRIIDVRKTVDQDRESQGFIHLQMGTNKFANQSGMTGFGMPRREITKYKDEIRGEMPHDESTISRQTSGWKDGASQQGMTGFGAFRNNTLNQLQNQDQRSQGMIPYQMGVNFLDSQAGKTAFGMPRRTYTPYVDDSHEDLPADISRRPEVPFWSGQETDFANQQGMLAFGTPRDVRGEYVRRMW |
| C**L2**C**ontig10_1_**AA | MSEKKVKKVKKKSRTGSEASSFDQKTIQEFKEAFAIMDQDKDGVISKGDLKDLYATLGSIASESQIDAMLKEAPGPINFTVFLTLFGERLTGTDPEATIIGAFQMWDKSDSGFITEEALMKILKNKR**GEPLSEDEVQSMYK**GNPPISGGKCDYKAFAHQICTGAAEELTKMNA |
| C**L2**C**ontig16_1_**AA | MAVPPDVVEK**IEAGYK**KLQEAPECKSLLKKYFTKEVMDQCKGLKTKLGANLLDVIQSGVANLDSGVGVYAPDAESYTLFKPLFDPIIQDYHNGFGPDQK**QPQTDLGEGKTQLLPDLDPEGKFINSTRVR**CGR**SLQGYPFNPCLTKENYTEMQDK**VKGVFVQLK**SDPELGGTYYPLEGMTKEVQTQLIKDHFLFK**EGDRFLQAANACRYWPTGRGIYHNDKK**TFLVWVNEEDHLRIISMQNGGNVGQVLER**LIKGVKAIETKVPFSRDDR**LGWLTF**C**PSNLGTTVR**ASVHIKLPKISAKPDFKKICDEMKLQIR**GIHGEHSETEGGVYDISNK**AR**LGLTEFEAVK**QMYDGVK**KLIELEK**AA |
| C**L2**C**ontig17_1_**AA | MSEEEYTDEEYEEEEVEEEIVESAPPSPSKPKAPPEEKKEEK**IEEEIKEEEKPITPLDK**EPHEVEDKEDEKEEREIQELHPQLRKTEQKPKEPEEKELTEAEIAMMEARKRHEAEEEAKMVGYEERRKQEMAQLDQELNQLKEQHRQRLEERKKEEAEMAAIRKQDEERRRKEEEERKKRVEEEKARKEAERHKRQMMMAGSFIGGGTGEKGVRNFVIEKRGDGEKLGPKSEGGARRKGPSAAEAAEAKRNYMAIVNRPVDVDKMLPNDIKNKIKQLHARIVKLEGEKYDLEKRKGRQEYDLKELSERQKQAARQK**ALNTGVDPTEVEDSNFPPK**VRVASKFDRKTDRREYGERREMFENPAAPPEPEIAHGSGRPPTEWGRKEFEELEQIRKNLEPPKYMEQVVAEGDAARPPVPVIPLQIPGNWNPEDEKSKKKKRVSLGKSENEEEENEENIGESKIKNKESSGGGGGGGGWRDKIKEKEAPAPPPKARA |
| C**L2**C**ontig18_1_**AA | MSDQEEYSEEEVDEEVEEAQDEVSVAAQEVSK**QEEAPPTPK**SPLK**SPPASPLK**SPK**SEKPESEQAEQQEFEEPEGGEDEPPLSPSK**EVRRSSVSTDVTGPQAQLRKTEIKQKEPEEKEMTEAEAAMLAAKKRHEEEEEAKMRDNDERRR**QEMVAVEQELQELK**ERQIQRKAEREQEERELAERRRQDEERRRQAEEERKARIEAEKARREEEKRKRQLMMAGSFGGVVQAEGEGGK**NFVIPEKGAGGGQAPGLPGQASKPR**GLSKEQQEEAKRNYMAIVNRPVDVSNMLPNDIKAKIKQLYSRIVKLEGEKYDLEKRKGRQEYDLKELSERQKQAARQK**ALNTGVDPTEVENTNFPPK**IRVASKFDRQTDRRDYVERRDLFEKPPPELIPTIAHGSGRPPTEWGRKELEELEQIRKNLEPPKYVEQVKAEGDAARPPVPVIPLQVPSEAFINANEAPIEAAPEPPAKPSRRIAAK |
| C**L2**C**ontig2_1_**AA | LRQLKRLFSTIKTIMAVPPDVIEK**IEAGYK**KLQEAPECKSLLKKYFTKEVMDQCKGLKTKLGANLLDVIQSGVANLDSGVGVYAPDAESYTLFKPLFDPIIQDYHNGFGPDQK**QPQTDLGEGKTQLLPDLDPEGKFINSTRVR**CGR**SLQGYPFNPCLTKENYTEMQDK**VKGVFEQLK**SDAELGGTYYPLEGMTKEVQTQLIKDHFLFK**EGDRFLQAANACRYWPTGRGIYHNDKK**TFLVWVNEEDHLRIISMQNGGNVGQVLER**LIKGVKAIETKVPFSRDDR**LGWLTFCPSNLGTTVR**ASVHIRLPK**ISAKPEFK**KICDEMKLQIR**GIHGEHSDSEGGVYDISNK**AR**LGLTEFEAVK**QMYDGVK**KLIELEK**AA |
| C**L2**C**ontig21_1_**AA | MTDFPIPFHHQFVNFKLPTK**IHFGGNIPEGANRFEIDLKSTDGEIFFHFNPR**FSENCVVRSSTKDGQQWQAEERDGGMPFAIGKPFLLEMLIEENGISVNLNFKLXLINXLNFFLCSVDGNQFCFFKARDNLETLISVEVVGDVLLNNFNVTCIGSVEVDEPAGKVPREAGGGGEIPEYTPTEPVAPVPGPQGQLPIPYQGPLDTLLTTNRMR**VVGTPHPGAQR**FTVNWKSSDGETLFHFNTRFDQNCIVRNATVDKQYPPTSEEREGPGCPFHPGK**QFALDFLLHGDIIR**CFVDGIEYCRFQARDDLHKVVLLDIHGDIALDQVLIA |
| C**L2**C**ontig4_1_**AA | KKDRQRNESLDSSDSGLSEDYSERDKRPENNEVEEEMEEVKEEEGGNEKEEEGGDEYEDNREEEEGREENEEGEEEEKDVERGKGNEEEGRKSKEVKIREKEEKEDKNCKKDDDLEEREEWEVRSLSPAAPLVEEEDVNIEEDEQVKDPPIERKNTSKVNSVNNTRQNSLISQQLFQSLSKREAREMLQSARYNLRRMSNKDEINKNTKILLKESEKGKKSPKRRPSSIKKKNEGNGSSRSSGKGRRPRRVEAELPNILR**AEELLER**LKLLARKKGRGEGGKEESETER**NDEEVEK**EEQKKDRREKGEEENKNEEKERERKKSSLEEMEDQNEQSTSSNLTQNPQISTRRNSSTNSSNFEAIWSKFTSHLNEEQKEDLDNRLVDYFFGSRRPPVPDWLFRPVYCTKCCRCIHRLMPKTGKSWPLFLELLTPKERGKTSWRHGVECMGDMQELLRAKRLLLRQMSSLTSGEEEEEVNRGRGRRLVEIEGEENERDLKWTKPPTKLSHCFCPLLIRHRHGLHHIVRASSKSKNRPKRSIKRTNGWVPSWRKREQTPKRKEKNWYKLNLL |
| C**L2**C**ontig8_1_**AA | MTDFPIPFHHQFVNFKLPTK**IHFGGNIPEGANRFEIDLKSTDGEIFFHFNPR**FSENCVVRSSTKDGQQWQAEERDGGMPFAIGKPFLLEMLIEENGISCSVDGNQFCFFKARDNLETLISVEVVGDVLLNNFNVTCIGSVEVDEPAGKVPREAGGGGEIPEYTPTEPVAPVPGPQGQLPIPYQGPLDTLLTTNRMR**VVGTPHPGAQR**FTVNWK**SADGETLFHFNTR**FDQNCIVRNATVDKQYPPTSEEREGPGCPFPPGKQFALDFLLPGDLIRCFVYGLEYCRFQARDDLPKVVLLDIPGDISLDQVLIA |
| C**L3006**C**ontig1_1_**AA | MVVSPGQTNNMMTNSNNQMAATAISQQQQIQMNNATSTKFSDNYDVKEELGKGAFSVVRRCVHKATNMEFAAKIINTRKLSSRDFQKLEREARICRKLKHSNIVRLHDSIQEEGFHYLVFDLVTGGELFEDIVAREFYSEADASQCIQQILESIAYCHDNSVVHRDLKPENLLLASRAKGGSVKLADFGLAIEVKGEDEAWYGFAGTPGYLSPEVLKKDPYGKPVDIWACGVILYILLVGYPPFWDEDHLRLYAQIK**NGAYDYPTPEWDTVTPEAK**ALIDAMLTLNPKKRITAQEALKVPWICDRQRVASMMHRQDTVDCLRKFNARRKLKVSTFLKINLNLYIFVDFFLVFFDFGRCFVYFYVSEIVNVFC |
| C**L312**C**ontig1_1_**AA | MSELTNGVATEDENVSGAQVEGEESEATLLKKNLK**LESQQAEEDEANKENADEK**TEDQVPEITNEDPMTTSMDGIEEIANEEPK**ETVEDDEQPKENGLDKAEEENTEKDAAEIPVSILDGGEEKQEEEPK**VEADSAAESVPVAEEEPAPEEQQTEEAPQEEADPPK**EESVAEPEHFELQAPPVPK**RPRTPNELDEPQPEPEQK**SESEQPVDEQPQEVGEKVEGGTLINGIIEFPR**RESASKQSTIASPIPATPK**SGAPPTPK**SGGAPVTPKSGATATPKSTRSGRAFDFNSNKTGAKKTTFEDEVLSVKSPKSRGSKSPRSPRTPKTPTSQVEEPKPEEAEDPPTENEVADSDAGK**LAAEEEER**QRQASFEERQREREEEEARFKRLTSWDRGEDGMPKEETK**EESISEQKEDTIAEDVEK**ESVKEESK |
| C**L315**C**ontig1_1_**AA | MSTQVPIRVLVTGAAGQIGYSLVIQIAKGDVFGKETPIVLVMLDIPPMAEVLKGVELELYDCALANLIAVEPVTTEEAAFK**DIDYAFLVGAMPR**KEGMERK**DLLAANVK**IFKSQGLALAKYSKPTVK**VLVVGNPANTNAFI**C**AK**YAAEKIPTKNFSAMTRLDHNRAIAQIAARCGVDCGSVKKVIIWGNHSSTQFPDVKHAKVIKGGTEIGAYEAVNDVPWIQNEFISTVQKRGAVIIEKRKLSSAMSAAKAACDHIHDWHFGTKDGDWVSMAVPSDGFYGIPEGLIFSFPITIDPKTRDWKIVQGLELDNFAKQKIEITTK**ELTAEREDALEVSK** |
| C**L320**C**ontig2_1_**AA | MRFFFFPKLSIISIIGFLWGIIAIISISSVSALTSVSTTSKALPDK**FLGSWTVDHSENFDEYLEAK**GYGWFMRQMVKLAGITKTFTKNDDGSYGCKIETTKKNVEWPK**FNLGEEFTAEYLDDSMHKIKFTYDAK**K**DSLTEVHTK**VDAPNDPADVYDYIIDGDGWLVMHMEYNDVKTKRFYKKL |
| C**L321**C**ontig1_1_**AA | MIIYK**DVFTEDELSSDSYPMKLVDDLIFEFK**GRQVVRKEGDIALAGANPSAEEMDEGTEEHVER**GIDFVLNHR**LQEMNCYEDQATFKAYIKDFMKK**VIEHMQK**QGK**SAEQVDAFK**K**KIQSWVVSLLAK**DRFK**TLAFFIGENMAEGKGEGQVAIVEYRQEGDEEVPTLMLIK**EALEEVKC |
| C**L32**C**ontig1_1_**AA | MPSGYLSHNLFGFQHPSR**IGDSGHVNTVSEEPKMVIIEGEQNNDDLETK**TENQNSAEKEIPRRQMRHLLADEFGTGKMGRQYKRMRPCFYSPIQCLMRKRRSGE |
| C**L333**C**ontig1_1_**AA | MTEDQKKLPMVPETVLKRRKVRAAQRASLLKNKLENIKKAKVKTQVIFK**RAEQYLIAYR**RKQKQELQLKRQAKKAGNFYVPDEPKLAFVVRIKGINKIHPRPRKVLQLFRLRQINNGVFIKLNKATIQMLR**IADPYIAWGYPSQK**IIRQLIYKRGYVKVKGQR**MPLTDNNIVEENLGK**QDIICVEDMINQIWTVGPHFK**QVTNFLWPFK**LSNPLGGFNKK**SNHFVEGGDYGNREDQINK**LLERMI |
| C**L344**C**ontig1_1_**AA | VDQYLVKGGTITTYKDAHNLRVMKFSVSPVVRVAVEPKNAGDLPKLVEGLKRLAKSDPMVQCIFEESGEHIIAGAGELHLEICLKDLEEDHACIPIKKSDPVVSYRETVTEESDIMCLSKSPNKHNRLFCK**AKPMATGLPEAIEGGEVSPTDDPK**NRARILAEKYEFDATDARKIWCFGPEGTGANLLVDVTKGVQYLNEIKDSVVAGFQWATKEGVMCDENMRGVRFDIHDVTLHADAIHRGGGQIIPTARRVFYASVLTAKPRIQEPVYLVEIQCPEAAVGGIYGVLNRRR**GVVFEESQIAGTPMFIVK**AHLPVNESFGFTADLRSNTGGQAFPQCVFDHWQILPGDPFESTSRPAQVVAETRKRKGLK**EGIPSLDNFYDKL** |
| C**L349**C**ontig1_1_**AA | FKILMNEMTDEQLSLLKQFVLMCK**ANPGLLHQPQYAFYREYLESLGAK**IPSGTESGGSKSESFKR**QPEETFEENQEEEKESDLPLPELDNSGVIEDK**TCGEEYAMGDPEKEVSDEDMEKAQEHR**QAAQMAFADGDYEK**SVEEYTK**AIEFNPGLAILHAK**RASTLLKMSKLSAAIKDCDKAIAINPDSAAAYKFRGRAHRLLGNWAEAHLDFALSCK**LDYDDQANEWLK**EVEPNAKKLHDYQRAKERLADEKETRARQERVRRAQEENKRAAEENKQK**YGEDSDDFDGDVPPGLFESFK**NNPELAKIMEELKKDPSKIGQYLNDPNVLNLIGMMGGKGGGGFNPSAFNMPTGDTDDSTPFKQEPPK**KAPEPDLD** |
| C**L34**C**ontig1_1_**AA | MKVFTVGLDQKPVEENKEKTDSPPPGYSTTETRIPLPMGPELPRPSLPIPPPQPIIVRKSRGYKGILLVMLSVFLMAIFALVLSEMAYSRQRDENYFKLRWAELKQRFGYENDGNIDYYTQAANAISDKLLSLTKSNEIQLPRVPQSASLEEETTSSSTTQDTPVVAIDTFPMAPSKIGNSADSSISRSSQTSDEVEPPHPFFGSGNFNGAFSQIRDARLKFLRNILQKIKQHAEDIGFDGTMQVSIVEVEPQNSDSSSTSNEDNNNNNNLLSNGNWPSSIFQRPQQQVLQFPQSSSAHFFPHPRPLVEDFKNSRSFLDGFGEMHQPSLFMQNNQVGEQEEQQRPRFFSPWALQQNELPSSHPIMPSGDMQQQQQIPSFIDNLRWPRINGFRQSAPEMLFFHPFFQPQQQQQQQQMNMRFPPPPPPSSDWAHPQQNSFGFGGNSGALPRPSSFEHWNNGVFQPQQNSMPQQQQQIPPSMGMMGGRQIEQQQVQQQPQQPQQPQPEAK**LSAPSVVIGDSGEEKSQVVQQPGQQPQQPK**QPEQKESSWMTPDLLPAWLFDNLSSRK**SNAAGGPPSPQQPAEMAPPK**FPSVSVIENNPSGSKSESDSKSSIDEGNSRSDETENTPSRLSADALPVETPKVIDIAHGEIGKINNNNKDDKDDDKDDTIGNSHQPEEVIEVDAAPQLNEQQTEQKNDEQKNEQNESKDQKEDQPKEQKEDEKTETKPVNVPLQQQKLFPEMPSVFFQVDDPANDHQAAAIQPAQDNLHLAERA |
| C**L350**C**ontig1_1_**AA | MASFFYFLFISVSLLILANADDAGRYPSGDDLVEGTTAARLHSSSDLPDDDEEECECEDDDETTVATHISTRSNGYPSNNGAPTSTKRPSNNGSSNNGGSSSVTGSVILRDKWVNGANCILAFKNNGNARACGVKFELTLGDNQRIQSIWNVEKVGDKVYR**IPDYIQLGPGVENRDIGVVYNDVPEPLPTIK**VLGQEEGCKHY |
| C**L358**C**ontig1_1_**AA | VNKELLALHNLRKAFAQDLSQRIKR**ASGPEVDDEFLSSPIQK**QKIIFLENNLEQLTSVHKQLVRDNADLRCELPKLEKRYRACTERIKSLEAALRETKENAMRDRKKYQFEVERIKEAVRQRNLARRGLQIAKPIRPGQHYGSNVVRPQNVGQS |
| C**L36**C**ontig1_1_**AA | MSVAESEGVPAAKPASDEEAKRQALAAKKAEVRKRLEEAGKKGKKKKGFLTPERKKKLRKLLMIKAAENLKNQQKQLDSERQSVLSKR**ISACPDVDKIDDVGQLSK**IYNDLFQKMLQLEEEKYDINFAVSQKDSEINEMTINVNDLRGK**FVKPTLK**KVSKYDNKFR**KVLAGEGEATSTKPK**EDFRAKLKAVEKK**NVMEVLENK**QKSKAEKPEWSKKGDEKGAGRRASAASSGGGAPPSPKKLTAGQAAPPPEEEEEEIIEDEGEGEEEEE |
| C**L375**C**ontig1_1_**AA | MGEQMKLEGQLR**GHNGWVTQIAASPVYK**NMLVSSSRDKTIILWQLDESGSVLTGKPLKSLHGHGHFVSDVVMSSDGQYALSGSWDKTLRLWDLNTGRTTRQFVSHTKDVLSVAFSADNRQIVSGSRDKKIKLWNTLAQCKHTIVNECHTDWVSTVR**FSPSNTNPVIVSAGWDR**IVK**VWNLGTCQLK**TNHIGHGGYINSVTVSPDGSLCASGGKDGSAMLWDLNEGK**HLYTLGGNDVINALAFSPNR**YWLCAAVGPVVKIWDLEDKTVVDELRLDIATITTGKKQPSPPQCTSLAWSLDGQTLFVGYTDNLIRVWRVSAR |
| C**L378**C**ontig1_1_**AA | MAAAAVDVNKAITGDKAAPIRNYNTQPHLIYKTVTGVNGPLVILGDVKFPQYDEIVRLTLPDGTKRTGQVLEVTKDKAVVQVFEGTSGIDAKNTICEFTGTILRTPVSEDMLGRVFNGSGKPIDKGPNVLAEDYLDINGQPINPWSRIYPEEMIQTGISAIDVMNSIARGQK**IPIFSAAGLPHNEIAAQIVR**QGGLVKLPNKKHGGEEGETDDFAIVFAAMGVNMETARFFKQDFEENGSMENVCLFLNLANDPTIERIITPRLALT |
| C**L37**C**ontig1_1_**AA | MVNPKGYRRGTRYMFARPFRRHGTEPLSTFYRVYK**RGDIVTVK**GNGAFQKGMPHKAYHGKTGKVFNVTK**HALGVIVNK**RVRNRIIPKRINVRVEHVKPSGSRSEFLKRCKENDQQKRDFKEGKAKFVPLKRKPQEPRSAHLVKVRKALQIEMLAPVRFEIVA |
| C**L381**C**ontig1_1_**AA | MGQKHSKKYSLKNGGTNRHSTEINDEVAFPPTASTNGRTASDEPVAVCISTEKNDYIQNAATEMAKVWTDNAVISADNNDENNKEK**LEETNENKLEEPILEK**IPKNEEKQKIQNLKEADTKIEISIEKENNSEPKEEQKTVGDVNLLENNKIEESESKQKEILKEENICELIENKKEDKEEVINNSVNNQEFCQTSKEEEVEEDEKEPVKENGEIEIIKENNFEGGEKEEEKEELNKLSRHLELVEDVLTNNNHTIPTDNLEQSIVCNTGKPSEI |
| C**L383**C**ontig1_1_**AA | MDSLHK**GWFTEFSPDDADR**IAGADGGSKMMHLDGQEMSGAWTGQAFSLDIDKVLFHQKSKYQDVLVLKSKTFGNVLVLDGVIQTTDRDEFAYQEMLAHLPMFSHPNPK**NVLIIGGGDGGILR**EVLKHASVEHVVMCEIDEMVVNVAKEYLHGLSSSFNSPKLELHIGDGFEFLRQRKNQFDVIITDSSDPIGPAESLFGSAYYELVFDALRDDGVLSSQAESIWLHLPLITKLVECVQKIFPSVAYASSSVPTYPSGCIGYLIAGKQKKDLRIPLRSLNNEQCKAMGLQFYNSQMHSASFVLPNFAASVLPPTKM |
| C**L384**C**ontig1_1_**AA | KIIKMAVDVNSALNRAYEELRNIDLDLSTLSGVQDRFSTRKRSANDSGNVTNASNDRRILDGGRRIIVGPDGSAKRSRHDDDLWNEQHQHNRYPQRDYSPPSLRPQRTLSSVIMSSTSGASTIETKSRTAVITEKKKSETKEDAVRNRRLFSNLLVGTLRQFQKEEKTVGVKGQAQLEKQREVERRLEENEKENRERLLRERDSLMEKRRDKEMQIQALRRQKAIEQSGXXEKTKHFQRLACFIQTQTKPPLFFLPSKHTLRTLELLKDSSKKIETLIELRREEMDRELKRLGTYIIGGNGVVMKQEIEEDSDDDNNSKQNNGGSGHPLKSTIVVKKEQQQVDENEVNIGPIIVSSTNRESNK**NVEGEVENETPPHPTK**SVVIDGDEEEGNDDNIEDNNNDVVDNVAVNTDKNGVNDNAFEGQDEFGENI |
| C**L3**C**ontig3_1_**AA | EAKFKEGESEYFYKLFEEQPIVIXQKTFKMPENDYDEEEAPNATMETKVASGGQPKRVGKWTLSQLR**QTDGIIPSQAGWNK**GDSQKLMTNFGTPRNTTTKIRAECLADVPEEIALKSHGEVRLQSGTNRFASQKGMVGFGTGRDVCR**EGVFVSQDPADLEPLPEEK**IRASDGIVRLQSGTNKFDSQKGMVSFGTNRRETTRMKDTKHPEYNHEVNIDQSEIPLQSGTNKFASQKGMTSFGTNRRETTKMLDTAHPEYSHESSIDQTSIPYQMGSNRYASQKGMTCFGQPRWEVLDPSISYQNRKSQGMVRLQSGTNRFASQAGMTGFGTPR**NTTYEAESGELPYEDMKKSETIIPSQAGWNK**GDSQKLMTGFGTPRDVKGKHLKR**IWELEYPEEAEISLDRL** |
| C**L405**C**ontig1_1_**AA | MPGALNIAVAR**DIDSIPSGPK**DAGDYQMITAHKVNR**APKPEDPYAWSAPK**VPAQED |
| C**L406**C**ontig1_1_**AA | IEINLDELVPHVNGPFTPDLAHPIDKLGQHAKENDWPSDIK**VGLIGSCTNSSYEDMAR**SANIARQAYKKGMKLKPIFTVTPGSEQVRATIERDGFIKDFENIGGIVLANACGPCIGQWDRKDVKMGEKNTIVSSYNRNFTGRNDANPATHAFVTSPEMTLALTLAGTLDFDPRR**DYLTAPDGTK**FKLEEPKGECLPEK**GFDPGTDLYQQPK**FSGDVXVDPKSNRLQLLSPFDAWDGKDLTDMVILIKVKGKCTTDHISAAGPWLKYRGHLDNISNNLFLTAKNAENDELNKVRNQLTGEWGPVCQTARDYKAKGQAWVAFGDDNYGEGSSR**EHAALEPR**HLGGRAIIVKSFAR**IHETNLK**K**QGLLPLTFVNPADYDK**VRPDDRVSLIELNTLAPGKKVKCVLKHKDGSKDVIELQHTLNEGQIEWFKAGSALNRMKQLKANK |
| C**L407**C**ontig1_1_**AA | MVKKPAKKKVAALPAGMKKAEAKKPAKVSLFEKRPR**NFAIGQDIQPK**RDLTRFVRWPKYVRLQRQRAVLEKRLKIPPPINQFKFTLDKPNASQLFKLLDKYRPETRQAKKERLRNRAQDRVEGKQEVVTKRPPTVRFGIKDVVKLIENKKASLVAIAHDVDPIEIVICLPALCR**KFGVPYVLVK**GKARLGTVVHRKTAAAVCLCDVNPEDRVALR**NLSDVALNNFNER**GDEIKKNWGGGI240 241MSQRSQARQAKIEKARMKEVRA |
| C**L41**C**ontig1_1_**AA | MSAATTISFPQHQQQPTAEAVPTIPPPFKETQGACSSLRQISADAFYVINEVVLKRLGPVPILKGDFHLLPFKVQRFVAEKAELCRPRGIFICDGSEHEAEEMTDLLVER**GVLTPLTAYENNYLCR**TDPRDCARVESKTWMVTREKFETVTHTPENVEPIMGHWMSPMQFAEELDARFPGCMAGRIMYVIPFSMGPLGSPLSKIGIQLTDSTYVVLSMRVMTRILPDVAEALGDKDFVRGIHSVGLPRPVKQK**VISHWPCNPER**VIIAHRPLEREIWSFGSGYGGNSLLGKKCFALRIASNIARDEGWLAEHMLIMGVTSPEGKEHFIAAAFPSACGK**TNLAMLEPTLPGWK**VRCVGDDIAWMKFDEEDGR**LYAINPEAGFFGVAPGTSMKTNPIAMATFQR**NSIFTNVAETNEGKVYWEGLEDEIKDREDIKIIDWMGNDWKLGESTTPAAHPNSRFCAPAKQCPIIHPNWESPKGVPIDAIIFGGRRPEGVPLVFECFSWEHGIFTGACLKSETTAAAEHKGKTVMHDPMAMRPFMGYNFGKYLEHWCNLKKPGRKMPK**IFHVNWFR**KSSQGKFLWPGYGENIRVIDWIVRR**LDGDNGIGIETAIGTIPTK**DSLNLNGLGEINWEELMSLPPNYWNEDSKEVRKFLEEQVNSDLPEKIRNELNDQEKRIASL |
| C**L423**C**ontig1_1_**AA | GIENITEFISKIFTLSPPKGSEEETEQKLCLEIHLPFAFKGNQETNFSK**NLGALLSIPTLTK**FIGAFSYSTGGCLAEFLTR**FPLLEPNPELFYR**YDINVYCENDTLVENYFSDLERWLTSQWSKAPTQDNLNNVRQARLKFVVDR**EDTAQQIISLLK**EKFETATNQCTFTLRLDCPGFALQPFSVENTNSGEFMAMEFKNEYTIK**ADQDIDDFEEEIEIEPLAK**KKPINEKESKDGRNGAEETEGDGDSNSSSTSVVVWKSYKLVR**ASNANLLGQLMK**GHLPLLKRKLILN |
| C**L431**C**ontig1_1_**AA | MLMGSTPSPRAXIRHEALK**AMVCETSVLDPDEGIR**FRGYSIPECQDKLPKAAGGSEPLPEGIWWLLCTGDFPDQKQVNAISKEWAARADLPDHVAQLLETTPPNLHPMAQFVAAIAALQTESKFAQAYSKGVAKSTYWEYTYEDSMNLLAKLPTIAATIYRNLYREGTSVGVIDVNKDWSANFCSMLGYTDPVFVELMRLYLVIHSDHEGGNVSAHTCHLVGSALSDPYLSFSAAMSGLAGPLHGLANQEVLVFLTK**LVGEVGHDYSEEELKR**WVWDHLKSGKVVPGYGHAVLRKTDPRYTCQREFALKHLPKDPLFKLVSDLYKITPNILLEQGKAKNPWPNVDAHSGVLLQYFGMKEMSYYTVLFGVSRALGCLSQMIWARGMGLPLERPKSHSTEGLMKLAKTAKGEK |
| C**L434**C**ontig1_1_**AA | MVTGK**TYAFDNVFQPNDTQENVYK**GAAHHIVQDVLNGYNGTIFAYGQTSSGKTHTMEGVIGESDKQGIIPRIINDIFNHIFNLDDENLEFHIKVSYFEIYNERIRDLLDVTKTNLTIHEDKNRVPFVKNVTELFVSSPDEVLAAIEDGKTNRQVAVTNMNEHSSRSHSVFQIQVDQENKATQKKLSGKLYLVDLAGSEKVSKTGAEGSVLEEAKNINKSLSALGNVISALAEGTKSHVPYRDSKLTRILQESLGGNSRTTIVICCSPASYNEAETKSTLLFGQRAKTIKNVVVVNEELTAEEWKRRFEREREKVNKLRQHMLIMDQEIKRWRAGEKLSESEWTTSIADLSANLTSNSDMTPTSMTESMIFPPSDRPNPQPLLSSRVGPISDDERRKYEEERTKLYAQLDEKDDEIQSQSQQVERQKQQLMEQEEVIKQRSLTNENLMTEVSKAQEQIAK**SQEETEELFSAIQEIALNLEQK**KTECSQLATDHEQLSEEINKKKLEATLLNTKLEEMKEICLTQKKRVYESVQNMLKEMSELGEANFTLAEKFVNDLDQSDKPIDEELLAHARICISKLSNDFKANQQKLLQLESGSGDAQKKADELSRELDDCKLQVQQLDAKNK**SLHSQIESQETQKR**QLEDEMDALNAKLANVNSSGEKKXEDLQQQHQKLVAQLR**DQIALK**NSQIKELTESIQEMQLAREKLQQDYDRLKNDESDKEKRLKSLSALS |
| C**L441**C**ontig1_1_**AA | MGKIMKPGRVVIMLNGRFAGRKAVVIK**SYDDGSSERPYGHGLVAGIDK**YPRKVVKRMGKKQIAKR**IAMRPFVKVTSLQHLLPTR**CTFEAEFDKSIVNKESIKDPKSKRKAKLHIKKEFETQYKAGKSKWLFTKLRF |
| C**L455**C**ontig1_1_**AA | DTYIGYLPLAHILEVCAELVCLAKGCR**IGYSSPTTLYDR**AMKIKKGSKGDCGELKPTLIACVPAIMDRIFKAVSDDVKERSPIQRELFRICYERKRNRYEEGYTSLVMNRLAFDRIRKILGGNLR**FVLSGGAPLNAETQR**FMNICFCCPVVQGYGLTETCGGASLADEHDLSTGSVGPPLRCCEIMLRDWKEAGYSPYNKPPQGEILIHGDNVAKGYYKMERTEDFIELKGKRWFATGDIGEFREDGSLCIIDRKKDLIKLAHGEYISLGRVETSLLTNQHIDNICVYGNSHYDYLIALVVPNQKNLEALAENNGIDGMPFDKLCESKELCIILQKELQDFVKDKLRRDEIPKKIYICKEPWTPASGLLTEAFKLKRKNIEKEFEQQIKMLYGDKSGVSKG |
| C**L46**C**ontig1_1_**AA | MKRFRFWSNVAK**VENSDPAGEVGDDGGSK**FFGIKSYLHNFYLTPGGLEHIDGVGKGTAENAWYLLPPPPTQRMGLYVCRLLTVLGLLLLLGGATAIIVGYCWPREQNIEGELMRIAIDQDEDGNFYVLPERLAEVMSSLQDPMHKWKMTGFCVFAAGASLMALSLLVPMLAQCCGGTRLAAFSSVDNTPNEPPVRIYPGGGCGIQPSGRFKVVPAKFAGSHKISPTNNGPVPVMEEISKIQPGSKGASKVGSPTLTCAAEEQMLLDQLVDSSDAQPLIR |
| C**L47**C**ontig1_1_**AA | MPSQTEIK**EIFNLYDEELDGK**IDGIQIGEVVR**ACGLKPTNAMVWK**AAGQEYKRKGEKR**LTFEEWLPIFDQLSK**EK**EVGSYADFMEGLKVFDKDESGK**IMAAELR**HVLLALGER**LNTEEVDELLQGVEDGEGMVNYDQFIK**KVLAGPYPEGD** |
| C**L480**C**ontig2_1_**AA | YLHHVAQNIKNGIQVAAQNCYK**VPSGAFTGEISPSMIKDLGVAYVILGHSER**R**HIFEEK**DVLIAEK**AAHALESGLNVIY**C**IGEK**LEEREANQTKEVNFR**QLDALLKVPNIDWK**K**IVIAYEPVWAIGTGK**TASPEQAEEVHKWIR**DYLEEK**VSKEIGQTTR**IIYGGSVTAGNCDELAKQPDVDGFLVGGASLKPDFIK**IFNAKQ |
| C**L500**C**ontig1_1_**AA | SGLKKVTADMQTHKNPQLRTQSTVPADKGKSPQKSVSPSGKAGIEKPPKIELKNQKVWEIEYHKDNRQIQIQADLKHSVYIFRCENSVIQIKGKANSVTVDSCKKTSVVFDALLSQVEVINCQSIELQTLGSLPTISIQKTDGCQVYLSKESLNAEIVTSKSSEMNILVPK**GADGDFTEFPVPEQFK**TTYNVKENKLQTQVSDIV |
| C**L520**C**ontig1_1_**AA | EKEEGNDRQIVEEDHDDEDEWEDAGDGREEDEQLGDEQEFDDEQEDEECDDQEEEDGDEKK**ELDDDEIVENPAYIPK**QGKFYMHDIDRSVPPARRSRVFNPSEDEEEEDPEGISAVSKCEDDMPKTRAERVHKWKHDMFDERAQGPKGRGELIRRYGRDIRRVNEDEDDGNEQQNIQNTDRSQQRTRGIRERGGGGIRRSRGRGLVVTNRRQPVQQQQFSYPKERDGRQEGQEEEGKQQERQSQQFSGSGGRVIRNSGTHGERGRAYGLG |
| C**L525**C**ontig1_1_**AA | MLINKFQLILLFILSVYALTILAVNPPFGRLSVNKGQLVGANGRPVQLRGISFWFSQWLTQWYSPPAVKAIKCFFNGNVVR**AAIGT**CC**SGYLENPQAAIK**AAMTVADAAIANGIYFIIDWHDVANQKCTNDEEFKK**FTNSAITFFTTILSK**YKGSPNLLLELWNEPICPWNKLK**EYYNAVLAR**VRKLDPNVVVILGTPWQSTGPSDDVINSPVSGKNLLYALHFYVVQSNQHIENQKKMILKAKSKGIGTFVSEYGDADVNLPAPLKPNEMKSFWAFMDQNKLSYCKWSLSNKDEVYSLVLPHCTPDQANQERCLSPSGK320 321LLRDHMKNQKNGITGC |
| C**L53**C**ontig1_1_**AA | MTKIVYSFYXKSVINKNRAYFKRFQVKFKRRRQGKTDYYARKRLTVQDKNKYNTPKYRLIVRFTNKRVTAQIAYSR**IQGDIIVNAAYSHELPR**YGIKLGLTNYAAAYATGLLLARRHLQKLKLDSVYKGKEEVDGEYYDVEGSNPNPFTCVLDVGLARTT1TGAKIFAVMK**GVADGGIHIPHSESRFFGFDNESK**QYNAEAHRDRIFGK**HVADYMK**YLKEEDEEAYKRQFSKFIKEGVEPDSIEGIYKKAHEAIRADPSHTKKEPKKPAEKKRWNAKKLTLRERKHRVANKK**AYLLHLK**DLQGQQLEA |
| C**L543**C**ontig1_1_**AA | MSLIINTFYSNKEIFLR**ELISNSSDALDK**IR**YQALTDPAQLETGK**DLYIKIVPNKADK**TLTIMDTGVGMTKADLVNNLGTIAK**SGTKAFMEALQAGADISMIGQFGVGFYSAFLVADRVTVTSKHNDDDCHQWESSAGGSFIIRNCVDPEMTRGTKITLYLKEDQTDYLEERRIREVVKK**HSQFIGYPIK**LLVEKERDKEISDDEAEDEKKDVKKEEEKEEEKEIKKEEGEDKEGE |
| C**L551**C**ontig1_1_**AA | GMAAQIPIK**HDQTANEEALK**AVRKDKEREASDGHDGTWVAHPGLVPVAQAVFDAHMKDGHNQILKQLPDFFTDNQRLTSPPEGVRTEAGFRR**NISITLGYLDSWLR**GTGCVPLYNLMEDAATAEISRAQLWQWLRHEAR**LEDGRTVDPQLVK**MTIASETERRLVRAGSVVNRIPEASELLEKFVLEPELSDFLTLDAYDKLVSEGK |
| C**L555**C**ontig1_1_**AA | MAPQPKQAKGKTGGETKAVATASKGASDNKKRVVKPGGKDRKAVTKALDAKKKLAKGKFTLHKKKIRTSVHFYRPSTLKQARAPRYPRKAVPKRNKLDAFAVIK**HPLTTESAMK**KIEDTNTLVFIVDVRSNKHQIKSAVKKLYNIEIAK**VNTLITPLHTK**KAYVRLAADCDALDVANRIGII |
| C**L561**C**ontig1_1_**AA | LIGTGHVSK**AAICGLDGSIWGK**SDNFK**IDQSEANAAANGLK**NSEGVLASGLRFEGEK**YFVLQADSER**IIGKKTANGFFIYKTDKAFIIGVYESGVQPEMCSKTTGALADYFRSINY |
| C**L578**C**ontig1_1_**AA | MGISQDNWHKRRKTGGKRNPIHKKRKHELGRPSANTKIGPKRIHLVRCRGGNIKHRALR**LDTGNFAWASEG**C**TR**KTR**IIDTVYNASNNELVR**TKTLVK**GSIITIDAVPFRQWYEAHYALPLAR**KKGQKLTEEEEQRISGHKRSKKTLKKYSDRQK**TAAVEPHVLEQFQAGR**LLAR**ISSRPGQSGR**CDGYILEGKELEFYMKKIRAKKTK |
| C**L5**C**ontig2_1_**AA | AFVLLIKRVCVCPSSSVFTFHSINKPTYSLNKQQKMSRKIFFAAILAIIAVSANGASNLPALDMASIPDEYK**DLVPPEVTTFYNELTEDDKK**ILKEVAEK**HSEYATDEDALNALKEK**SEKLYTK**ANELR**NLVKDRISKLNPEAK**TFVDTIIEK**LKALRPK**KDEKPNLTELRKEANEIVEK**FKALSEEAKESLKTNFPK**ITGVIQNEK**FQK**LAQSLLKPEGAAAPA** |
| C**L600**C**ontig1_1_**AA | AVQLRGISLWFSQWLTQWYSPEAVKAIKCFYNGNVVRAAIGTCCSGYLENPSAAIKAATAVADAAIANGIYFLIDFHDVANEKCTNDAEFKK**FTNSAIKFFTTILNK**YKGSPNMLLELWNEPICPWSKLKDYYNAVLPVIRKLDPNVVTILGTPYQSTGPSAEVINNPVSGTNLMYTLHFYTVTDTNHIQQQKQMVLNARSKGLGVFVTEYGDADVFLPAPLNPSSMKDFWKFMDQNKLSYAKWSLSNKDEVYSLVKPYCTPAQAMQESCLSDSGKLLRDFMKTQNNGITGC |
| C**L604**C**ontig1_1_**AA | MADDFAPSGDGLPADDNWGATDVPGGQGDMQMTTIDFPEIKLFGK**WSLSDIEVNDASLVDYIAVK**DKSAKYLPHSAGRYQIKRFRKAHCPIVER**LV**C**SMMMHGR**NNGKKLMAVRIVKHAFEIIHILTGENPVQILVGAVINGGPREDSTRIGRAGTVR**RQAVDVSPLR**RVNQAIWLLCTGSREASFRNIK**TIAE**C**LADELINAAK**GSSNSYAIKKKDELERVAKSNR |
| C**L610**C**ontig1_1_**AA | MKYVAAYMLASLGGNAQPTAKDLEK**ILGAGGLDVDMENAK**AVVKALEGK**NLDEVIAAGEKKLSSVPSGAPAAAGPAATGAAAAAPAAEEK**KEE |
| C**L613**C**ontig1_1_**AA | MNPEKIKKLQQNAEHVRTGGKGTARRKKKVVHKTTALDDKKLQSNLKK**LSVTNIPGIEEVNMIKEDGTVIHFNNPKVQASVPANTFSITGTAENKR**ITDMLPGILNQLGAESLAHLKKLANNVTTQYKPSDDDVPELVGDFEEASKNETK**EVQPQQHHIGGES** |
| C**L623**C**ontig1_1_**AA | MSERAGFQAGFGGAPPAGAAAGGRGVRGGARGRGGPRGRGGRGGRGKDAEREWQPVTKLGRLVKERK**ITTIEEIYLHSLPIKEYEIIDLLLADK**KLKDEVLKIMPVQKQTRAGQRTRFK**AFVAIGDGQGHVGLGVK**CSKEVATAIRGAIIAAKLAIIPVRRGYWGNKIGDPHTVPCKVTGKCASVLVRLIPAPR**GTGIVGAPVPK**KLLQMAGIDDCYTSAVGQTATLGNFAKATYYAIQR**TYSYLTPDLWK**ENMLPKNPLDCSTGSTAP |
| C**L633**C**ontig1_1_**AA | GSGHAGMECAIMNLLDEGETILVVQNGVWGQRAADLSDRLGLNVRKLVVPEGEVISVEDFSEAVDNYRPQVAFLCHGESSTGVAHPLERFGDICNAYDCLFLVDVVASLGGAPFHADDLKVDCVYSATQKVLNCPPGLAPISFSEKALKKMLNRKKRVPSFYLDALELGNYWGCFDEPRRYHHTAPISLVYALREGLSIVAQEGIKNLVERHQKNAKLFYDALK**ELGLEPYVQNENYR**LPCLTTVRVPDGVDWKAVQSK**LMAQGYEIAGGLGPTAGK**IWR**IGTFGVNSNPEDIDALK**LALKSALYEQKEEKTHLKASI |
| C**L639**C**ontig1_1_**AA | FKRTLFNLIFKHSKMLNIPSFTSTR**SMVPWAGHRRHWMTDPLEQMDR**WMQSVDRYFDDRVGFQMRDHAAQMKIEPNGDFTYK**VDASGFRPEELK**VEVHGNEVVISGDHREENQGESVHRQFVRRVYIPEGVKQETVKCEMDNAGRLCVTAHRDVEGKR**NIPIDFKPANATATTGEQPK**TK |
| C**L651**C**ontig1_1_**AA | LKEVFILDACRTPIGSFRSQLASLPAPSLASVCIKELIKRTNIPAKEIQEVFLGQVCQANVGQAPARQAALGAGLDVGVAVTTINKVCSSGLKSIMLAAQQIQTGHQNIVIGGGMESMSQVPYYLERGELPYGGAKLIDGVVKDGLTDAYDQHVHMGVCAEKTAKDNSIGREEQDEYAIQSYKRSAAAWESGAISKEIVSVEVKHRKGIQIVSKDEEFTKVDFDKLKQLRAVFQKENGTVTAGNASTLNDGACAALLSNSEIAKKYSFRPLAKIICFADAATNPIDFPVAPVLLIPKLLSSASLKLGDISLFELNEAFSVVPLAAIKKLGLDPAK**VNAHGGAVSLGHPIGMSGAR**ILTHLVHALKPGQFGLAAICNGGGGASGMIIQKLE |
| C**L673**C**ontig1_1_**AA | GYPTLKLFRNGKPSEYGGGR**DAASIVAWLK**KKTGPPAK**ELTSADELK**EFQDSAEVAAVAYFSDKDSKEAKVYIELASSFDDVPFAIIYDSAVAKDANIKDKGIVLFKKFDEGK**TDFDGEKIELDILK**KWVQSNR**IPLVSEFSQETAAVIFGGEIKSHMLLFVSKESPDFEKLESEFK**AAAKEFKGK**LHFVYINTEVEDNQR**IMEFFGLKKEELPAVR**LISLEEDMTKYKPDWTEITTENVVK**FTSAYLDGK**LKPHLMSEEIPEDWDK**QPVKVLVGKNFEQVARDEKKNVLVEFYAPWCGHCKQLAPIWDKLGEKYQDREDIVIAKMDSTANEVEDVK**VQSFPTIKFFPAGSK**K**VIDYTGERTLEGFSK**FLDSGGK**EGAGLSDTEK**AEK**EAEEEAEEPEEGEDKDEGHTEL** |
| C**L687**C**ontig1_1_**AA | MDLSXAGNASGVCDGAAAVIVADEESVEKHHLTPLVRILGWQSVGCDPTIMGIGPVEAIRILCKKSGVELNK**IDLIEVNEAFAPQVLSVQR**ELGIDLDR**LNVNGGAIALGHPLAASGAR**ITAHLAYELKRRNVRYGIGSACIGGGQGIALLFENVQ |
| C**L68**C**ontig1_1_**AA | MSSRQTPGGLGFQVVAKQASK**YNDYEGEMLLK**WIK**KLSGEGFQPTGNR**ENFYNLLK**DGTLLCKTANALKPGTVK**K**IQNPTGGNFACMENINAFVK**FAK**ETGVPTEELFQSVDLFEAR**DLFSVCVTLLSLGRILEKQGKSNPYSG |
| C**L6**C**ontig6_1_**AA | MLDFFNQQMHLCGLAQAPGNPVIACQINLDKNFAFIEFRSIDETTAGMAFDGVNFMGQQLKIRRPR**DYVAPANNYEMLGSLPVSAVVVDSPYK**LFIGGLPNHLTSDQVKELLSAFGQLKAFNLVMDSVTGFSKGYAFCEYLDSTLTDQAIVGLNGMQLGDNRLLVQLASTGCKTVVPTPGIPSNPIALAGIDLSKGAGPPTEVLCLMNLVVEEELQKDDEYEEILEDIRDECNKYGNVISLEIPRPGYEERGVGKVFVEFQSKEECQKAQAALTGRKFSNRVVVTSYYDPDLYHRRQF |
| C**L701**C**ontig1_1_**AA | MAARKGKQKEEQAVVTLGPQVREGESVFGVAHIFASFNDTFVHVTDLSGRETIVKITGGMRVK**ADRDEASPYAAMLAAQDVAER**CKQVGITALHVKLRATGGTKTKTPGPGAQAALRALARSGMKIGR**IEDVTPIPTDTTR**RKGGRRGRRL |
| C**L712**C**ontig1_1_**AA | MYPGMNQHYSNGGSSGGGGPKRFRGGGGGGGGGHDGVDMYHEALAAGKFELRMLIPSRSAGAVIGRGGEYIKSLRAKYDANINVPDRSTPERVLTIICHQDSIEQCVLEILNKLSEGEKASRGEVDVK**VLVHQSHAGAIIGR**GGSKIKELREQTQCRFKVFQECCPMSSDRVCLITGDSAKLPGAVKILIDFVKDIPFKGPHVPYDSAAYNPQLCHAYGGFEGGNMGAPAPPMPPPHFDFPHHGPPQPGVGYAGFGPGPGGPPTHTPGGPPLMGPPGPYPGGPGGDQITTTQVTIPSKLSGTIIGKRGECINRIRNDSGAR**IEIDTQTDPFGTDER**IITITGTTHQIHMAQFLLQQSVRSSESGRRYLTERQGLE |
| C**L737**C**ontig1_1_**AA | MFCFYFINLLIFLNNINLIYSDK**GIDISSPANLEQTK**CFKKNGYKSIYIRVWQSNNK**FDGNSVNTIK**NARTAGIDNVDCYVFPCAKCINASPKNIIETILKNLKAKNVKCDRIWLDVEGLQYWKTNKQQNVDFIQGMVNELKANKQK**IGIYTSNAQWQPITGGSTKFNDIPLWYPHYDNK**ANFVDFKPFGGWKKPLIKQYDDNKKVCNVNADLNFF |
| C**L765**C**ontig1_1_**AA | KVFNKYGMAAEIVNTVLK**EILAELK**VDAEIGQLCSRGDARILELTSNLFKREKNVQKGIAMPTCISIDNCVCHFSPLRSEPPVLLKEGQVVKVDLGAHVDGYIATAAHTVVIGANRDNKVTGKK**ANVIVSAYNVMEMVLR**MLKPEKNLKNVEISEKMGKLAK**IYETTPIENMLSHNIER**FKPVGDKQIIQNPGDEQKSKSEKCTFETFEVYTIDVLISTGEGKSKTLDARTTIFKKTDDMVYNLKLKASRSFFHEAQQKFGSMPFSIRDFEDEKVAKVGSTECAKHDLMQPYPVLYEKEGDYVAQFKCTAIIMPNGIYKITGIPLDNAILKCDVK**IDNNEFLGLLNEPLKPK**KKKGGGGEKEKEINNKEAENNLVKEKKKEEVEKNV |
| C**L781**C**ontig1_1_**AA | MTKGDSSGGDGGNVGMPGGKVPQDVNREDIKELATKGLAK**INHQSNSAHHFGLAK**IEKAESQVVSGVLYTLTLRVGKTDCLKTQVNSDNLAECNKYTGDLDNTEIYTVEYWLQPWR**NFEQVTITK**GQKEE |
| C**L78**C**ontig1_1_**AA | MREVISIHVGQAGVQIGNACWELYCLEHGIQPDGRMPSEQSAQDDSFSTFFSETGNGRHVPR**AVMVDLEPTVIDEIR**TGTYKQLFHPEQLITGKEDAANNYARGHYTVGK**EIIDPVLDR**IRRIADNCQGLQGFLIFHSFGGGTGSGFTSLLMERLSVDYGKKSKLEFSIYPAPQVSTAVVEPYNSVLTTHTTLEHSDCAFMVDNEAIYDICRR**KLDVERPSYTNLNRLISQVVSSITASLRFDGALNVDLTEFQTNLVPYPRIHFPLATYAPVISAEK**AYHESLSVSEITQMCFEPSNQMVKCDPRHGKYMAVCLLFRGDVVPK**DVNQAIATIK**TNR**SIQFVDW**C**PTGFKVGINYQPPTVVPGGDLAK**VPR**AV**C**MLSNTTAIAEAWARLDHKFDLMYAK**R**AFVHWYVGEGMEEGEFSEAREDLAALEK**DYEEVGMDSAGGEDADEY |
| C**L793**C**ontig1_1_**AA | MNFFLLFPFLLINFCYFTNGEK**ATIEQCDTVMEYRVDDSCICTK**DNLINCLK**TLITEGTCTKENYPFNFIESPCSK**MYSKCSSPKDGCVSGACACFDVVVDCLIAGKCKQLTLTKLLDSPTLNKGVRVSDTASNLNNLFDAHFEKIE |
| C**L79**C**ontig2_1_**AA | ARGDNSTLSSPTNNLITLMATETNYPIPYRSK**LTEQFEPGQTLILK**GKTAEDSVRFTINLHHASPDFSGNDVPLHISVRFDEGKIVYNTFAKGEWGKEERKSNPYKKGDDIDIRIRAHDSKFTIFADQKEIKEYEHRVPLSSVTHFSIDGDILVTHIHWGGK**YYPVPYESGIVGEGFAPGKTMLIFGTPEK**KGKRFHINLLRKNGDIALHFNPRFDEKAIIR**NSLIANEWGNEER**EGKLPLEKGIGFDLKIVNEEYAFQIFLNEERFCSYAHRMEPHDLLGLQIGGDVEITGIQIF |
| C**L7**C**ontig2_1_**AA | MVNGELLKKQIQQFKLGKDAAADYYKELFSK**YSDVADAYGGVDPETVGR**SQRYIMMAMNEIQALMQLPEQVKDERSWRSSLSNVKEHYSDSDVPLSNFIKTKDAWLAIMQKYAGGLSAEQKKEWEELFTKASSDMKKWGWI |
| C**L809**C**ontig1_1_**AA | DR**VINQVLTEMDGMGSK**K**NVFIIGATNRPDIIDPAILRPGRLDQLIYIPLPDEASR**LQIFKANLRK**TPIASDVDLNFLAKTTVGFSGADLNEICQR**ACKLGIRESIEKAVVKEKERQLRAQK**GEELMEEADEDPVPELTR**KHFEEAMKFARRSVSDNDIR**KYEMFAQTLQQQR**GFGTNFKFPGGK**AAGGSAGGGPQPSAGGNDDDDLYS** |
| C**L811**C**ontig1_1_**AA | MAAKCAREECGKTVYPIEELKCLDKVWHKGCFKCTACGMTLSMKTYKGYEKKPYCEAHYPKTVPSAVIDTPEMERVRINTKNQSAVQYHEQFEKMRGTKIEVADDPEMKRLLDNTKVQSLAHYHGEHQKKVDQDAHR**SGGESPNPAAAAAAVSPTEKKPVGSIADYDPMSAEQPTK**QINASGPTGGGKAPGGKAATGGFTFKALYDCAAISNTETRDRGKGQRPKPKNQNQN |
| C**L822**C**ontig1_1_**AA | LISLLLIFFLSVPQFDADQGNEEDPDEKPR**YQQYVNEQNYHIPFK**TRISEPFREYQTVHAVGKVNADPK**RVDLNFYKGGNEDDDMPLHLSVR**FDEGLFKGNVVFNTLVNKNWSEPEER**VQSPFKPDDEFDIR**VRILPGKFQVFGNRIEIGIFDQR**IPLYGVNHVSLLGDLK**SLR**VFHYGGTK**FPNPYNAIAKLLPGMRLDISAMPLGNRVNFNLYKTNNDIALHISIRYSEGIIVR**NSMISNQWGDQEIEGSLPLAK**NEIFDLTIINEQANFLILFNGKKFCKFVHRSQNFDIETLEVDGTFELHTVTINNAR |
| C**L843**C**ontig1_1_**AA | MTSRPLVSVYNEKNETTGAQIK**LPAVFHAPIRPDIVSFIHDQMR**KNKR**QAYAVSTAAGHQTSAESWGTGR**AVARIPRVRGGGTHRSGQGAFGNMCRGGRMFAPTKVYRRWHRRINIAQRRYAVVSAIAATGVPALVQAR**GHVIDQVSEVPLVIADK**IESFTKTKEAVGFLQR**ANVWADIEK**VYKSKRYRAGKGKRRNRRYKQK**LGPLIIYNNDGGIVR**AFRNIPGITLLSVKHINLLKIAPGGHLGRFTIWTESAFRKLDSIYGTWTQKSWVKKGFSLPHAK**MTNSDFAR**IIRSDEITKVIRPVRKQTKVAKIHRNPLRKHGLMVKLNPYASVLRRAAILASKKGEEKKNAKGAEVKQTA |
| C**L846**C**ontig1_1_**AA | MTLVVKHPSILWAQRAGQIFLTIEDGGLKIDELCCEDDKFKLKGEKGGNKYEADLVLYGKLKGAERRKIETDRRIEFVIPKETEEWWPRLLKVSGKVPWIKIDFDKWKDQDEDDKDDMRDMDFSSFGLPGGGGKGYDDLDLGDDDHDYDDEMGDLEDVDDENEGIPKEGKKAEKDGKDEGK**DSATNGELVDDGKEK**KVVEEAKE |
| C**L860**C**ontig1_1_**AA | MGNQQFQQQKPGSGDKQGEGDKKRRFGPPIPTRFGKRKKGSKGPEASNKMPNVTPVTRCKLRLLKYERIKDYLLMEEEFIRNQERHKPQEERQEEERTKVDEMRGSPMAVGTLEEIIDDQHAVVSTNVGSEHYVNILSFVDKEQLEPGCAVLLNHKTHAVVGVLADDTDPMVSVMKLEK**APTETYADVGGLEQQIQEIK**EAVELPLTHPEYYEEIGIKPPKGVILYGPPGTGKTLLAKAVANQTSATFLRVVGSELIQKYLGDGPKMVRELFR**VAEEHAPSIVFIDEIDAIGTK**RYESNSGGEREIQR**TMLELLNQLDGFDSR**GDVKVLMATNRIDSLDPALIRPGRIDRKIEFPLPDEKTKRRIFGIHTARMQLENVNLEEFIEAK**DDLSGADVK**AVCTEAGLLALRDRRMRVTMEDMRKAKENVLYRKKDGAPESMYL |
| C**L886**C**ontig1_1_**AA | MSGSSKISRETLNECVAELLKKSTEKKRKFRETVELQIGLKNYDPKKDKRFSGTVRLKYIPRPHVKVCILGDQKHCDEANANGLPCMTADDLKKLNKDAKLIKGLCKRYNAFLASESLIKQIPRILGPGLNK**AGKFPSVVTHNDNLTAK**VDEIKATVKFQMKKVICLAVAIGHVEMTQEELVSNISLGINFLVSLLKKNWQNVRSLHIKSTMGPPQRLY |
| C**L895**C**ontig1_1_**AA | MRGDISK**ADSIPADAQAEAVSVEK**QQELLLMNKLASSGKLPPKSAGSFLQKRLQQRKFFDSGDYNMNKDKKTTTAVVGQQQPVPATVVSQAPPISVAAAPKLAATIEENLPKIHQQDELMPTIRPPAGSFSGCSMDAGDEDTDEHLQIPRPDTVPQRKSSILHPSAHSKLSPQPHIHHEHTDESLSMPPQXKKKNLSEWKVFFDDLKMIG |
| C**L902**C**ontig1_1_**AA | TLCLNPDVGFICWLSAVNRLPECRFYR**FVHGNEPGSLPTAIYTHR**IGVQIVSPSPSICSTSSWGLPYLHRAPSSIFKHKFCEFESTTSKQRHFALALWPCEEIESSKKFPIIHSVYGGPSIQIVRNSWHSISQFLKFLAYGYAVVIVDGRGSANRGVSFEGHIKEAMGSVEMEDQVEGLKQAIDQTGNILDGERVVCIGWSYGGYMSIQLIAEYPNIYKAAVVGGAVTDWQLYDTAYTERYMGTPQQNAEASKIFSIISKVDMLPNQEGRILFVHGMIDENVHFQHTILLINSLIKAFKPHQLLLFPTESHVFRTGESVEYFHASVLFFFRRALGLGIFVKKENI |
| C**L928**C**ontig1_1_**AA | MADNKENGDTKTAVEVK**EEVKDVVDSTISSPPTNGK**QQKEQKKIVAERVTGIVKWFNVMNGYGFICRDDTNEDIFVHNSAISKNNPEKAQRSLGDGEK**VLFDVVEGTKGPEASNVTGPEGQNVVGSK**YAADASQQRRYPRRNFYRVRNRPRGNRLSAGAPTGENGGTGEGKDTAPGEXVIDANGGGGDGQPRKEQ |
| C**L929**C**ontig1_1_**AA | MFFLKYFPISIFIFFVVLSFKDDATIVTAHPTHLSQPGLQMLLITGQQQDKPHRIVKR**FSCSGQDCYINCVNQEK**NEKDKEKAK**NDCAGQCDCNLSSK**KN |
| C**L930**C**ontig1_1_**AA | TMK**LNVAYPATGAQKTFELEDEKK**YRIFFEKR**MAQEVEADPLGDEWK**GYILRITGGNDKQGFPMKQGVITNGRVRLLLSKGQSCYRQRRKGERKRKSVRGCIVDANLSALACIIVR**KGDNEIEGLTDK**IVPRRLGPKRATKIRRLFNLTKEDDVRKYVIRRTLPAKEGKKPRVKMPKIQRLVTPITLQRKRRRIALKNKRRQKRREESAIYHQTLAKYAKEKIAEKAARRHSSASQSKASESEPRHSFTTVKKSKK |
| C**L983**C**ontig1_1_**AA | LKQFVEAGAMAVRRCAKNDLKRIAKGTGATLTSSLATLEGDELFESSLLGNAEEVVQERVSDDELILIKGTKAKSCASIILRGANDVMLDEMERSVHDALCVVKRVLEGKKLVVGGGAVETALDVYLENFALNLTSR**EQLAVAAFADALLVIPKTLATNAAQDAISLLAK**LRAFHNKSQNDKKFEDLK**WSGLDLVEGIVR**QNK**DAGIFEPLISK**VKSLKFATEAAITILRIDDLIKLNKVDKKGRDEDECE |
| C**L98**C**ontig1_1_**AA | LIRFKKFQKKSEISKKMSKTFLILTFTIWLLSSTTATKNK**SNGLLGELLDLELAK**KLIKEINVK**AAGIWTASVNELSR**LPLAKQKILCGVKLSAELKINKTEATPPKFDTKPGCECFPKVDFDARTKWSGCSQIIGRIQHQGQCGSCWAVSTASAYTDRHCIARAKKGQNSPANDAGYQFSAFDVLTCSMQGDGCR**GGWPYSAWQWIQTKGVCTGTDYTWRSGCKPYPFSPNQAGPAPPCK**SSCTASWRTAYPQDK**HMGVSATQLNGGQATVAAIQREIQTNGPVVAIFAVYSDFMSYR**SGVYFR**TSNQLVGYHAVR**IIGWGTQTCFNSNQKVDFWIGANSWGTGWGEYGFFKIRRGVNEVGFEQSGISFGIPKV |
| C**L999**C**ontig1_1_**AA | MADFNGLLNGASFTEEQFKQLNEHLATRGYLR**GFLPSSDDSYVFGVLK**SSTISAQYPHVARWFKHMQSFSDAEETGWPKSASGGSGPAPKATTTPKEAPKEAPKPAAADDFDLFGSDESDDEEKKKLTEQRLKAYAEKKAKKPGVIAK**SNVIYDVKPWDDTIDLK**EMEKNVR**SIATDGLVWGASKIEPVAFGINK**LQICCCVEDEK**VSTDWLEEEIR**GFEDLVQSVDVVAFNXGLNXKKLNERDDEGKLKSIFLLAKVSLNLIFIVFWCVV |
| C**L99**C**ontig1_1_**AA | MYQRDRDRMARVRDQERDNTRRDRRYRDEGTQNGGARSRSPRFGLRNGVGGRYPQSPDRSISHNRRPEEDRMFERRNENDRVRRRDDERPAERRGVGRRSQERDIETSKRAVDESRVFGRWADNRDENEGMPRRRFEEEKRLSPSRHQERERRDYGRRNERRRSVERNNKTSEIERERELARRLHRRYEEEEKRHQFDEYKNELHVKSSKSSSKQEREIQYEDEGVSIEDGEVSPEKKILVRRQRSEEKEDDEETNVPLPNSTLSGFLSHLRGSKDNKNKQDKETSSTPAKHEKGSKSFDEKIPKEREESKGEKSISLERPILLEKVKNEQLTPTKTPKKNEATNKKTPTTEGLPPKNVSPAKEKDEETLKDSDLKDIDRQHANFNGRERDEDDFGDDDKVNEREEYCGRTHDEVDINMESNIANVETQEGLQFTSQDHDESSKDVDKSCDHFDEDLIKSSYFVEEK**EVTNENIDEPEDKGDK**MEGSIKEEKIEQITSPVKS |
