# Supplementary material for: Direct Identification of the Meloidogyne incognita Secretome Reveals Proteins with Host Cell Reprogramming Potential
Source: PLoS Pathog. 2008 Oct 31;4(10):e1000192. doi: 10.1371/journal.ppat.1000192 (PMC2568823; doi:10.1371/journal.ppat.1000192)
Supplement: Table S5 — Correspondence between our observations and previous reports of secreted proteins from parasitic nematodes. Proteins are ranked by alphabetical order according to nematode species and then by number of unique peptides per protein. (0.31 MB DOC) [file ppat.1000192.s005.doc]

**Supplementary Table S5: Correspondence between our observations and previous reports of secreted proteins from parasitic nematodes.**

| Protein number | number unique peptides | Ratio Sec/Whole | protein name | reference | Species |
| --- | --- | --- | --- | --- | --- |
| 1 | 21 | 2.34 | Heat shock protein 70 kDa | [1] | *Brugia malayi* |
| 2 | 20 | 0.96 | Actin |
| 3 | 18 | 9.83 | Enolase |
| 7 | 16 | 2.92 | Protein disulphide isomerase |
| 17 | 10 | 10.35 | Triose phosphate isomerase |
| 18 | 10 | 2.01 | Translationally controlled tumor protein |
| 21 | 9 | 14.32 | Calmodulin |
| 35 | 7 | 2.09 | Nematode polyprotein allergen (gp15/400), NPA-1 |
| 61 | 5 | 1.90 | FKBP-12 |
| 85 | 4 | 0.98 | Cyclophilin-2 cyp-2 |
| 86 | 4 | 1.35 | Galectin Bm-GAL-1 |
| 90 | 4 | 0.59 | Embryonic fatty acid-binding protein Bm-FAB-1 | [2,3] | *Brugia malayi/ Ascaris suum* |
| 160 | 3 | 5.35 | Inorganic pyrophosphatase | [1] | *Brugia malayi* |
| 185 | 2 | 7.33 | Cystatin CPI-2 |
| 188 | 2 | 0.83 | Cyclophilin-5 cyp-5 |
| 322 | 1 | 2.29 | Transthyretin-like family protein |
| 376 | 1 | 2.29 | High mobility group protein |
| 380 | 1 | 3.05 | Ubiquitin-like protein SMT3 |
| 385 | 1 | 0.38 | Transthyretin-like family protein |
| 404 | 2 | 20.16 | Actin 1 |
| 28 | 8 | 7.64 | Cytoplasmic Cu/Zn-superoxide dismutase | [4,1] | *Brugia pahangi/ Brugia malayi* |
| 139 | 3 | 0.78 | Annexin-2 (gp-nex) | [5] | *Globodera pallid* |
| 199 | 2 | 0.63 | Secreted glutathione peroxidase | [6,1] | *Globodera Rostochiensis/ Brugia malayi* |
| 99 | 4 | 1.83 | vap-1 | [7] | *Heterodera glycines* |
| 311 | 1 | 1.53 | vap-1 | [7] | *Heterodera glycines* |
| 81 | 4 | 4.01 | Ubiquitin extension protein (Hs-UBI1) | [8] | *Heterodera schachtii* |
| 4 | 17 | 5.07 | 14-3-3b protein | [9,1] | *Meloidogyne incognita/ Brugia malayi* |
| 8 | 15 | 0.46 | Tropomyosin | [10,1] | *Meloidogyne incognita/ Brugia malayi* |
| 10 | 14 | 9.02 | Calreticulin | [11,1] | *Meloidogyne incognita/ Brugia malayi* |
| 26 | 8 | 1.09 | Pectate lyase 3 | Jaubert,S., Ledger,T.N., Abad,P. and Rosso,M.-N., Unpublished. | *Meloidogyne incognita* |
| 40 | 6 | 15.28 | Glutathione S-transferase-1 | [12] | *Meloidogyne incognita* |
| 42 | 6 | 1.49 | Cellulase/beta-1,4-endoglucanase (M-ENG1) | [13] | *Meloidogyne incognita* |
| 54 | 5 | 1.13 | ATP synthase subunit family member (atp-2) | [10] | *Meloidogyne incognita* |
| 67 | 5 | 4.01 | Glutathione S-transferase-1 | [12] | *Meloidogyne incognita* |
| 118 | 3 | 4.01 | Troponin C-like protein | [10] | *Meloidogyne incognita* |
| 157 | 3 | 2.29 | Beta-1,4-endoglucanase | [14] | *Meloidogyne incognita* |
| 178 | 2 | 5.24 | Cellulose binding protein precursor | [15] | *Meloidogyne incognita* |
| 181 | 2 | 1.26 | Venom allergen-like protein (Mi-vap-2) | [16] | *Meloidogyne incognita* |
| 290 | 1 | 0.04 | Myosin regulatory light chain | [10] | *Meloidogyne incognita* |
| 291 | 1 | 0.56 | Polygalacturonase | [17] | *Meloidogyne incognita* |
| 323 | 1 | 1.02 | Beta-1,4-endoglucanase | [14] | *Meloidogyne incognita* |
| 413 | 2 | 36669.00 | 14-3-3b protein | [9] | *Meloidogyne incognita* |
| 167 | 3 | 2.08 | Putative amphid protein | [18,19] | *Setaria digitata/Globodera rostochiensis* |

1. Hewitson JP, Harcus YM, Curwen RS, Dowle AA, Atmadja AK et al. (2008) The secretome of the filarial parasite, Brugia malayi: Proteomic profile of adult excretory-secretory products. Molecular and biochemical parasitology 160(1): 8-21.

2. Mei B, Kennedy MW, Beauchamp J, Komuniecki PR, Komuniecki R (1997) Secretion of a novel, developmentally regulated fatty acid-binding protein into the perivitelline fluid of the parasitic nematode, Ascaris suum. The Journal of biological chemistry 272(15): 9933-9941.

3. Michalski ML, Monsey JD, Cistola DP, Weil GJ (2002) An embryo-associated fatty acid-binding protein in the filarial nematode Brugia malayi. Molecular and biochemical parasitology 124(1-2): 1-10.

4. Tang L, Ou X, Henkleduhrsen K, Selkirk ME (1994) Extracellular and cytoplasmic CuZn superoxide dismutases from Brugia lymphatic filarial nematode parasites. Infection and immunity 62(3): 961-967.

5. Fioretti L, Warry A, Porter A, Haydock P, Curtis R (2001) Isolation and localisation of an annexin gene (gp-nex) from the potato cyst nematode, Globodera pallida. Nematology 3: 45-54.

6. Jones JT, Reavy B, Smant G, Prior AE (2004) Glutathione peroxidases of the potato cyst nematode Globodera Rostochiensis. Gene 324: 47-54.

7. Gao B, Allen R, Maier T, Davis EL, Baum TJ et al. (2001) Molecular characterisation and expression of two venom allergen-like protein genes in Heterodera glycines. International journal for parasitology 31(14): 1617-1625.

8. Tytgat T, Vanholme B, De Meutter J, Claeys M, Couvreur M et al. (2004) A new class of ubiquitin extension proteins secreted by the dorsal pharyngeal gland in plant parasitic cyst nematodes. Mol Plant Microbe Interact 17(8): 846-852.

9. Jaubert S, Laffaire JB, Ledger TN, Escoubas P, Amri EZ et al. (2004) Comparative analysis of two 14-3-3 homologues and their expression pattern in the root-knot nematode Meloidogyne incognita. International journal for parasitology 34(7): 873-880.

10. Jaubert S, Ledger TN, Laffaire JB, Piotte C, Abad P et al. (2002) Direct identification of stylet secreted proteins from root-knot nematodes by a proteomic approach. Molecular and biochemical parasitology 121(2): 205-211.

11. Jaubert S, Milac AL, Petrescu AJ, de Almeida-Engler J, Abad P et al. (2005) In planta secretion of a calreticulin by migratory and sedentary stages of root-knot nematode. Mol Plant Microbe Interact 18(12): 1277-1284.

12. Dubreuil G, Magliano M, Deleury E, Abad P, Rosso MN (2007) Transcriptome analysis of root-knot nematode functions induced in the early stages of parasitism. New Phytologist 176(2): 426-436.

13. Wang X, Meyers D, Yan Y, Baum T, Smant G et al. (1999) In planta localization of a beta-1,4-endoglucanase secreted by Heterodera glycines. Mol Plant Microbe Interact 12(1): 64-67.

14. Ledger TN, Jaubert S, Bosselut N, Abad P, Rosso MN (2006) Characterization of a new beta-1,4-endoglucanase gene from the root-knot nematode Meloidogyne incognita and evolutionary scheme for phytonematode family 5 glycosyl hydrolases. Gene 382: 121-128.

15. Ding X, Shields J, Allen R, Hussey RS (1998) A secretory cellulose-binding protein cDNA cloned from the root-knot nematode (Meloidogyne incognita). Mol Plant Microbe Interact 11(10): 952-959.

16. Wang X, Li H, Hu Y, Fu P, Xu J (2007) Molecular cloning and analysis of a new venom allergen-like protein gene from the root-knot nematode Meloidogyne incognita. Experimental parasitology 117(2): 133-140.

17. Jaubert S, Laffaire JB, Abad P, Rosso MN (2002) A polygalacturonase of animal origin isolated from the root-knot nematode Meloidogyne incognita. Febs Letters 522(1-3): 109-112.

18. Jones JT, Smant G, Blok V (2000) SXP/RAL-2 proteins of the potato cyst nematode Globodera rostochiensis: secreted proteins of the hypodermis and amphids. Nematology 2(8): 887-893.

19. Sasisekhar B, Suba N, Sindhuja S, Sofi GMA, Narayanan RB (2005) Setaria digitata: Identification and characterization of a hypodermally expressed SXP/RAL2 protein. Experimental parasitology 111(2): 121-125.
