# Supplementary material for: Direct Identification of the Meloidogyne incognita Secretome Reveals Proteins with Host Cell Reprogramming Potential
Source: PLoS Pathog. 2008 Oct 31;4(10):e1000192. doi: 10.1371/journal.ppat.1000192 (PMC2568823; doi:10.1371/journal.ppat.1000192)
Supplement: Table S6 — Functional classification of secreted proteins. The secretome was classified into 9 subfamilies (Protein class) using KOG and BLASTP. (0.07 MB DOC) [file ppat.1000192.s006.doc]

**Supplementary Table S6: Functional classification of secreted proteins.**

| Protein class | Category | Number of identified proteins | Example |
| --- | --- | --- | --- |
| **1** | Cell shape | 33 | Actin |
| **2** | Nucleic acid binding factors | 48 | Nucleosome assembly protein NAP-1 |
| **3** | Post-translational modification, protein turnover, chaperone functions | 103 | SUMO; Protease |
| **4** | Metabolism | 88 | Aldehyde dehydrogenase |
| **5** | Signal transduction | 25 | cAMP-dependent protein kinase |
| **6** | Protein synthesis and secretion | 93 | Ribosome; Vesicle coat complex COPI |
| **7** | Detoxification | 17 | SOD; Glutathione peroxidase |
| **8** | Cell wall modifying enzymes | 8 | cellulase |
| **9** | Not classified | 94 | SEC2 |
