# Supplementary material for: Direct Identification of the Meloidogyne incognita Secretome Reveals Proteins with Host Cell Reprogramming Potential
Source: PLoS Pathog. 2008 Oct 31;4(10):e1000192. doi: 10.1371/journal.ppat.1000192 (PMC2568823; doi:10.1371/journal.ppat.1000192)
Supplement: Table S7 — Meloidogyne incognita proteins potentially targeted to the plant nucleus. Three sets of proteins were recognized. The first set contains both an NLS and a DNA binding domain, the second set contains only an NLS, and the third contains only a DNA binding domain. Proteins are listed according to their number of unique peptides. All proteins were identified by at least two mass spectra using a filtering criterion of 0.1% FDR at the peptide level. (0.19 MB DOC) [file ppat.1000192.s007.doc]

**Supplementary Table S7:** ***Meloidogyne incognita* proteins potentially targeted to the plant nucleus.**

| **protein number** | **Contig or Accession number** | **Protein name** |
| --- | --- | --- |
| **NLS and DNA binding activity** | | |
| 109 | CL765Contig1_1_AA | 42 kDa containing proliferation-associated 2G4 |
| 112 | MI00853 | Histone H1 |
| 171 | 223g03c1.1_1_AA | Sr protein |
| 241 | MI01375 | Histone H2B |
| 295 | 208a01r1.1_1_AA | Putative u4/u6 small nuclear ribonucleoprotein |
| 389 | 58419220 | RNA polymerase II, large subunit |
| 449 | 14992.m11057 | Poly(A) polymerase and related nucleotidyltransferases |
| 483 | 1708107 | Histone H2B |
| **Only NLS** | | |
| 16 | CL1191Contig1_1_AA | Cell Division Cycle related family member (cdc-48.1) |
| 22 | CL349Contig1_1_AA | TPR Domain containing protein/ similar to suppression of tumorigenicity 13 |
| 33 | CL333Contig1_1_AA | 60S ribosomal protein L7, putative |
| 48 | CL578Contig1_1_AA | 40S ribosomal protein S8, putative |
| 49 | CL2889Contig1_1_AA | 60S ribosomal protein L6, putative |
| 58 | CL36Contig1_1_AA | Troponin family protein |
| 68 | CL19Contig2_1_AA | cAMP-dependent protein kinase |
| 72 | CL930Contig1_1_AA | 40S ribosomal protein S6, putative |
| 82 | CL860Contig1_1_AA | 26S protease regulatory subunit |
| 135 | 218p13c1.1_1_AA | 60S ribosomal protein L27a |
| 177 | CL2Contig17_1_AA | TropoNin T family member (tnt-2) |
| 206 | CL886Contig1_1_AA | 60S ribosomal protein L10a, putative |
| 215 | CL32Contig1_1_AA | putative heme d1 biosynthesis protein |
| 287 | CL26Contig3_1_AA | Protein kinase domain containing protein |
| 376 | MI09158 | Ubiquitin-like proteins |
| 409 | 17569439 | Sex determination and Dosage Compensation defect family member (sdc-2) |
| 414 | 14992.m11170 | Serine/threonine protein phosphatase 2A, regulatory subunit |
| 416 | 17570289 | Glutamate synthase |
| **Only DNA binding activity** | | |
| 47 | 219i04r1.1_1_AA | Histones H3 and H4 |
| 55 | CL999Contig1_1_AA | Elongation factor 1 beta/delta chain |
| 73 | CL613Contig1_1_AA | RNA polymerase II general transcription factor BTF3 and related proteins |
| 108 | CL145Contig1_1_AA | Transcriptional regulator of the PUR family, single-stranded-DNA-binding |
| 127 | MJ00541 | RNA polymerase I-associated factor - PAF67 |
| 162 | MI00295 | helicase domain protein |
| 203 | CL15Contig1_1_AA | Polyadenylate-binding protein (RRM superfamily) |
| 204 | CL712Contig1_1_AA | PolyC-binding hnRNP-K protein HRB57A/hnRNP, contains KH domain |
| 239 | MJ00832 | mRNA cleavage and polyadenylation factor I complex, subunit RNA15 |
| 249 | MA00426 | DNA replication factor/protein phosphatase inhibitor SET/SPR-2 |
| 251 | MJ00163 | Small Nuclear ribonucleoprotein G |
| 258 | MH01721 | H/ACA snoRNP complex, subunit NOP10 |
| 259 | MI06351 | Splicing factor RNPS1, SR protein superfamily |
| 262 | MI01658 | Elongation factor 1 beta/delta chain |
| 277 | CL1630Contig1_1_AA | Transcriptional coactivator p100 |
| 285 | CL2965Contig1_1_AA | U1 snRNP component |
| 293 | CL2931Contig1_1_AA | Spliceosomal protein snRNP-U1A/U2B |
| 294 | CL6Contig6_1_AA | Splicing factor U2AF, large subunit (RRM superfamily) |
| 295 | 208a01r1.1_1_AA | Putative u4/u6 small nuclear ribonucleoprotein |
| 296 | 223a18r1.1_1_AA | RNA-binding protein La |
| 324 | 210e11c1.1_1_AA | Putative DEAD-box RNA helicase DDX1 |
| 349 | MI07344 | mRNA cleavage factor I subunit/CPSF subunit |
| 352 | MC05730 | Putative transcriptional regulator DJ-1 |
| 359 | MI08326 | RNA-binding protein RBM8/Tsunagi (RRM superfamily) |
| 373 | MI01515 | Transcription factor containing NAC and TS-N domains |
| 374 | MI07562 | Nucleosome-binding factor SPN, POB3 subunit |
| 383 | MP01497 | RNA polymerase II transcription elongation factor Elongin/SIII, subunit elongin C |
| 387 | 115534393 | Histone H4 |
| 388 | 17561988 | Histone H2 |
| 400 | 121998 | Histone 2A |
| 407 | PP00571 | Histone 2A |
| 420 | MA02593 | Nucleotide excision repair factor NEF2, RAD23 component |
| 426 | MC01039 | Histone 2A |
| 430 | MJ03828 | Predicted helicase, DEAD-box superfamily |
| 440 | MC05088 | DNA polymerase delta processivity factor (proliferating cell nuclear antigen) |
| 456 | MJ00925 | Q subcomponent binding protein/mRNA splicing factor SF2, subunit P32 |
| 462 | 14459.m00240 | RNA-binding protein NOVA1/PASILLA and related KH domain proteins |
| 470 | MP01239 | Nucleosome assembly protein NAP-1 |
| 471 | MH02369 | mRNA splicing factor |
| 485 | 27311795 | Histone H2A |
