# Supplementary material for: Direct Identification of the Meloidogyne incognita Secretome Reveals Proteins with Host Cell Reprogramming Potential
Source: PLoS Pathog. 2008 Oct 31;4(10):e1000192. doi: 10.1371/journal.ppat.1000192 (PMC2568823; doi:10.1371/journal.ppat.1000192)
Supplement: Table S8 — Plant protein contaminants of the nematode secretome. Plant proteins were identified from root exudates that diffused across the molecular cut-off membrane into the medium for treating nematodes. No nematodes were present. All proteins were identified by at least two mass spectra using a filtering criteria of 0.1% FDR at the peptide level. (0.04 MB DOC) [file ppat.1000192.s008.doc]

**Supplementary Table S8:** **Plant protein contaminants of the nematode secretome.**

| Number unique peptides | Species | NCBI nr Accession number | Protein name |
| --- | --- | --- | --- |
| 2 | Solanum tuberosum | 8439545 | Methionine synthase |
| 1 | Lycopersicon esculentum | 4731573 | Remorin 1 |
| 1 | Arabidopsis thaliana | 6403494 | Reversibly glycosylated polypeptide |
