# Supplementary material for: Direct Identification of the Meloidogyne incognita Secretome Reveals Proteins with Host Cell Reprogramming Potential
Source: PLoS Pathog. 2008 Oct 31;4(10):e1000192. doi: 10.1371/journal.ppat.1000192 (PMC2568823; doi:10.1371/journal.ppat.1000192)
Supplement: Table S9 — Oligonucleotide primers that were used to prepare probes for in situ hybridization. Two of the probes (calreticulin [1] and β-1,4-endoglucanase [2]) were made based on previous work to serve as an internal control. (0.08 MB DOC) [file ppat.1000192.s009.doc]

**Supplementary Table S9:** **Oligonucleotide primers that were used to prepare probes for *in situ* hybridization.**

| **Protein number** | **Contig or Genbank accession number** | **Protein homology** | **Forward primer (5'-3')** | **Reverse primer (5'-3')** |
| --- | --- | --- | --- | --- |
| **10** | **AF402771** | **Calreticulin** | **GATAAGAAACCCGAAGATTG** | **ATTGTCGAAGATTGTACCAGA** |
| **11** | **CL5Contig2_1** | **SEC-2** | **CGGCGAGCAATGATGTCC** | **ATGATAAACTGGCTGATAG** |
| **13** | **CL312Contig1_1** | **Protein with unknown function** | **GTTTCTGGTGCGCAAGTG** | **TCCTCTTCTGCTTTATCTAATCC** |
| **16** | **CL1191Contig1_1** | **CDC-48.1** | **GAAGATGAGGAAGGAAATCTC** | **TCACGTTTCGGAGCAATAGC** |
| **17** | **CL480Contig2_1** | **Triosephosphate isomerase homolog** | **CATCAACATCTGGTTGTTTTGC** | **CCAAATATCGACTGGAAAAAGATTG** |
| **18** | **CL321Contig1_1** | **TCTP** | **GAAGGTTATTGAGCATATGCAG** | **ACTTCACTTCTTCCAAAGCTTC** |
| **42** | **AF100549** | **β-1,4-endoglucanase** | **ACTTTCACGACCACAATGCTC** | **GCAAAGGTTTGTGCCGCTTAC** |
| **322** | **CL2552Contig1_1** | **Transthyretin-like protein** | **TGAGAAAACAAGGAGTAGCCATTAGC** | **TGCGATGACAAGGAGCTTCC** |

1. Jaubert S, Ledger TN, Laffaire JB, Piotte C, Abad P et al. (2002) Direct identification of stylet secreted proteins from root-knot nematodes by a proteomic approach. Molecular and biochemical parasitology 121(2): 205-211.

2. Rosso MN, Favery B, Piotte C, Arthaud L, De Boer JM et al. (1999) Isolation of a cDNA encoding a beta-1,4-endoglucanase in the root-knot nematode Meloidogyne incognita and expression analysis during plant parasitism. Molecular Plant-Microbe Interactions 12(7): 585-591.
